# Consistent dissection of the protein interaction network by combining global and local metrics

**DOI:** 10.1186/gb-2007-8-12-r271

**Published:** 2007-12-21

**Authors:** Chunlin Wang, Chris Ding, Qiaofeng Yang, Stephen R Holbrook

**Affiliations:** 1 Physical Biosciences Division, Lawrence Berkeley National Laboratory, Berkeley, CA 94720, USA; 2 Division of Infectious Diseases, School of Medicine, Stanford University, Stanford, CA 94035, USA; 3 Computational Research Division, Lawrence Berkeley National Laboratory, Berkeley, CA 94720, USA

## Abstract

We propose a new network decomposition method to systematically identify protein interaction modules in the protein interaction network. Our method incorporates both a global metric and a local metric for balance and consistency. We have compared the performance of our method with several earlier approaches on both simulated and real datasets using different criteria, and show that our method is more robust to network alterations and more effective at discovering functional protein modules.

## Abstract 2

A new network decomposition method is proposed that uses both a global metric and a local metric to identify protein interaction modules in the protein interaction network.

## Background

Protein complexes are building blocks of cellular components and pathways. A comprehensive understanding of a biological system requires knowledge about how protein complexes are assembled, regulated, and organized to form cellular components and perform cellular functions. The emergence of a variety of genomic and proteomic techniques to systematically obtain such information has generated an enormous amount of data [1-11]. However, interpretation and analysis of such data in terms of biological function has not kept pace with data acquisition, mainly due to the complexity of the problem and the limitation of current techniques to handle the data.

In this paper, we address the issue of constructing protein interaction modules from the protein interaction data. Highly connected protein modules are mostly found to be protein complexes performing a specific biological function. The concept of protein interaction modules as fundamental functional units was first outlined by Hartwell *et al*. [12]. Protein interaction modules are composed of a variable number of proteins, with discrete functions arising from their individual constituents and their synergistic interactions. A multi-protein complex, such as the ribosome, is one common form of interaction module; other examples of protein functional modules include proteins working collectively in a pathway, such as signal transduction, that do not necessarily form a tightly associated, stable protein complex.

To detect protein interaction modules from protein interaction data, we use a graph theory approach. Protein interaction networks are routinely represented as graphs, with proteins as nodes and interactions as edges. In a graphical representation of a protein interaction network, a functional unit, or a group of functionally related proteins, is tightly connected as a community, while proteins from different functional units are more loosely connected. In the past few years, new algorithms have been developed to extract communities from a generic network. Girvan and Newman [13] proposed a decomposition algorithm (GN algorithm) to analyze community structure in networks. Their algorithm iteratively removes edges based on betweenness values, the number of shortest paths between all pairs of nodes in the network running through an edge, in contrast to the traditional hierarchical clustering algorithm where closely connected nodes are iteratively joined together into larger and larger communities. In a different approach, Radicchi *et al*. [14] replaced the edge betweenness metric with an edge clustering coefficient - the number of triangles to which a given edge belongs, divided by the number of triangles that might potentially include it, given the degrees of the adjacent nodes. The edge clustering coefficient is a local topology-based metric and a candidate edge with the lowest clustering coefficient is removed one at a time in the algorithm of Radicchi *et al*. (the 'edge clustering coefficient' algorithm, ECC algorithm for short).

When applied to a large network, these two algorithms give substantially different results. The reason is that an individual edge with larger betweenness does not necessarily have a lower clustering coefficient, although on average it will. Ultimately, the global metric in the GN algorithm behaves differently from the local metric in the ECC algorithm. In this paper, we propose to resolve this conflict by combining the global and local metrics to form a consistent and robust algorithm. We make three additional significant contributions: a new metric (commonality) that takes into account the effects of random edge distributions; a new definition of a protein interaction module; and a novel filtering procedure to remove false-positive interactions based on a random graph model analysis. We demonstrate that our new algorithm is more effective and robust in terms of discovering protein interaction modules in protein interaction networks than either the global or local algorithm by application to the large yeast protein interaction network.

## Results and discussion

The principal result of this paper is the development of a new algorithm for extracting protein interaction modules from a protein interaction network. We first present the new methodology developments and then compare the performance of different algorithms, including the MCL algorithm [15], on simulated networks where protein complexes were known. The MCL algorithm is a fast and scalable unsupervised cluster algorithm for graphs based on simulation of stochastic flow in graphs [15] and was found to be overall the best performing one by the Brohee and van Helden study [16]. Note that our proposed new algorithm, the GN algorithm, and the ECC algorithm are divisive partitioning-type algorithms, while the MCL algorithm is a non-partitioning algorithm. Both the modularity [17] measure and productive cuts in the following sections are not applicable to the MCL algorithm. Second, we compare the results of different algorithms on a small protein interaction network where protein complexes are largely known. Lastly, we apply our new algorithm, the GN algorithm, the ECC algorithm, and the MCL algorithm, whenever applicable, to two large yeast protein interaction networks and evaluate the performance of each algorithm based on the value of modularity [17], overlap with Munich Information Center for Protein Sequences (MIPS) complexes [18] and Gene Ontology (GO) term enrichment of each cluster.

### A new commonality metric

Consider two proteins A and B. Let *k *be the number of common interacting partners (or neighbors) between A and B. If A and B belong to the same protein complex, they likely share many common interaction partners, that is, have a large *k*. On the other hand, if A and B do not belong to the same protein complex, they likely have few common interaction partners, that is, have a small *k*. However, randomness also enters the equation. Let n, m be the number of total interacting partners for protein A and B, respectively (n and m are also called degrees of A and B). A standard model of a protein interaction type network is the fixed-degree-sequence random graph [19] where the interactions follow the hypergeometric distribution. From this model, the average number of common interacting partners between proteins A and B in a random graph is given by:



N is the total number of nodes. To offset this random effect that a large k results from large n and m, we propose a new commonality index as:

$$\frac{k+1}{\sqrt{n\cdot m}}$$

The square root of *n*·*m *makes it a scale invariant. We note that in [14], the authors define a similar metric as:

$$\frac{k+1}{\min(n-1,m-1)}.$$

### BCD algorithm

Our goal is to discover protein interaction modules. Intuitively, when two protein functional modules are sparsely connected, edges between them should have higher edge-betweenness values and lower commonality, whereas edges within a module should have high commonality and low edge-betweeness. Thus, for sparsely connected functional modules, edge-betweenness highly correlates with edge-commonality. When protein functional modules overlap, the correlation between the global metric and local metric becomes less clear. For this reason, we combine these two metrics to build a more consistent and robust metric. The new BCD (Betweenness-Commonality Decomposition) algorithm is summarized as follows: step 1, calculate the edge commonality (**C**) for each edge in the network; step 2, calculate the edge-betweenness (**B**) for each edge in the current subnetwork; step 3, remove the edge with the maximal ratio B/C; and step 4, repeat steps 2 and 3 until no edges remain.

Like the edge clustering coefficient in the ECC algorithm, the edge commonality is a static property of an edge in the context of the entire network, telling how strong the affinity is between two nodes it connects. The edge commonality is calculated only once at the beginning of a decomposition process, while the edge-betweenness is updated each time an edge is removed to achieve best results [13]. This algorithm runs with *O*(*M*^2^*N*) computational complexity, where M is the number of edges and N is the number of nodes in a network. As a practical matter, we calculate the betweenness using the fast algorithm of Brandes [20] where the edge-betweenness value can be obtained by summing pair-dependencies over all traversals [21], so that we can easily parallelize the computationally costly betweenness calculation.

### A new definition of protein interaction module

Intuitively, a protein interaction module is a subnetwork in the protein interaction network with more internal interactions than external interactions. A precise definition of the interaction module is not trivial. A number of definitions of community (or protein interaction module in terms of the protein interaction network) have been proposed with different criteria [14,17,22]. No clear consensus of module definition exists.

**Figure 1:**
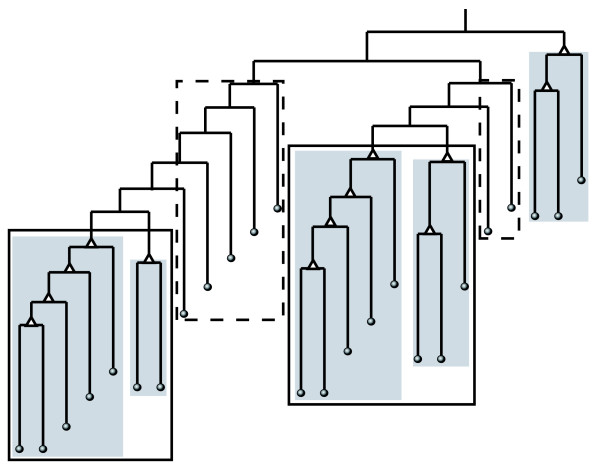
A sample decomposition tree showing protein interaction modules. Special parents are marked with triangles. Modules as defined in the text are shown as shaded subtrees. Two modules with the same parent are merged if the edge commonality between the two modules is above a threshold (shown as boxes). Dashed lines outline singletons.

**Figure 2:**
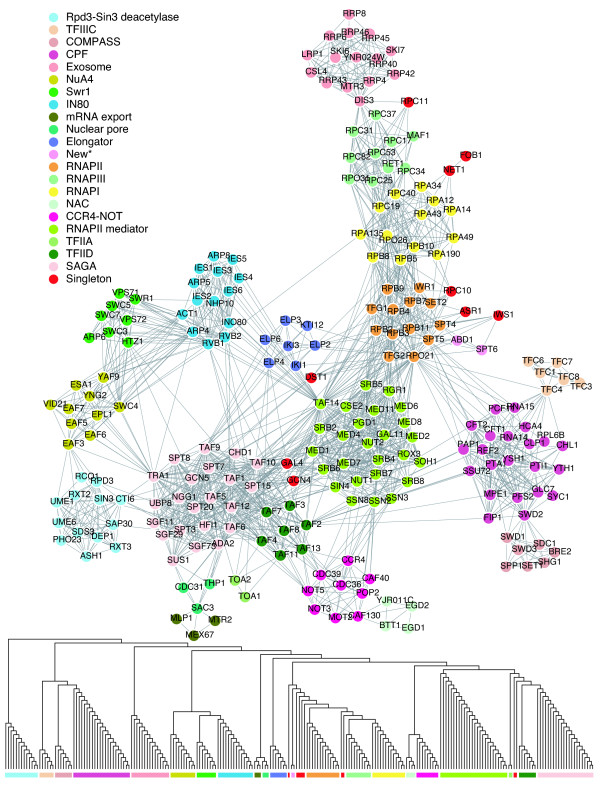
A yeast transcriptional sub-network (upper) and the decomposition tree constructed by the BCD algorithm (lower). Predicted protein modules are highlighted with colored bars (lower panel) and protein nodes in the network (upper panel) are colored accordingly. The module names in the upper panel are inferred from their members' annotation information. Singletons are colored red.

All three algorithms (BCD, GN, ECC) in this study transform a network into a decomposition tree (Figure 1). In this tree (called a dendrogram in the social sciences), the leaves are the nodes, whereas the branches join nodes or (at higher level) groups of nodes, thus identifying a hierarchical structure of communities nested within each other. When inspecting the resultant tree from either one of the tree algorithms on a small yeast transcription network with 225 proteins and 1,792 interactions, where known protein interaction modules can be inferred from the annotations of well-studied proteins, we found most, if not all, protein complexes, within which proteins are tightly grouped as subtrees in the decomposition tree with uniform structure similar to those shadowed subtrees in Figure 1. Similar results were seen in much larger networks. Based on those observations, we propose a precise definition of a protein interaction module utilizing the decomposition tree structure. We first note that on the decomposition tree, all leaf nodes are single proteins, while non-leaf nodes are collections of proteins. We define a 'special parent' as a non-leaf node with at least one child being a leaf (Figure 1). A protein interaction module is then defined as the nodes of a maximal sub-tree where all non-leaf nodes are special parents. Further, when two modules share the same parent, we merge them (Figure 1, subtrees in solid boxes) when the maximal commonality of edges connecting these two modules is larger than a pre-defined cutoff. Currently, the cutoff is set at 0.1 to avoid merging two modules with very limited connections between them. Results on actual protein interaction networks indicate that proteins within a module as defined above have very similar GO terms and perform similar functions (see Figure 2 for examples). The dangling nodes outside modules (in dashed boxes in Figure 1) are simply categorized as singletons.

### Filtering false-positive interactions

Most yeast protein interaction data were obtained from large-scale, high-throughput experiments, which generally contain false positives [23]. To minimize the number of false positive interactions, we apply a statistical test to measure the reliability of an interaction (edge). We rigorously calculate the statistical significance of each interaction between two proteins as the random probability (*P *value) that the number of common interacting partners occurs at or above the observed number. Previous work has shown that the statistical significance based on the number of common interacting partners highly correlates with the functional association of two proteins [24,25].

In a species with N proteins, the number of distinct ways in which two interacting proteins A and B with n and m interaction partners have k partners in common is given by $C_{k}^{N-2}\cdot C_{n-k-1}^{N-2-k}\cdot C_{m-k-1}^{N-n-1}$. The first factor ($C_{k}^{N-2}$) is the number of ways to choose the k common partners from all N proteins except proteins A and B. The second term ($C_{n-k-1}^{N-2-k}$) counts the number of ways of choosing dangling partners of protein A (note that the common partners and protein A, B are excluded). Similarly, the third term ($C_{m-k-1}^{N-n-1}$) is for choosing dangling partners of protein B. The total number of ways for the two interacting proteins to have n and m interaction partners, regardless of how many are in common, is given by $C_{n-1}^{N-2}\cdot C_{m-1}^{N-2}$. Therefore, the probability to randomly see two interacting proteins with n and m partners, sharing k common partners in a species with N proteins, is given by:

$$p(k|-,n,m,N)=\frac{C_{k}^{N-2}\cdot C_{n-k-1}^{N-2-k}\cdot C_{m-k-1}^{N-n-1}}{C_{n-1}^{N-2}\cdot C_{m-1}^{N-2}}$$

The statistical significance is then calculated by:

$$P=\sum_{k=k_{0}}^{\min(n-1,m-1)}p(k|-,n,m,N)$$

where k_0 _is the observed number of common partners shared by two interacting proteins. An interaction with *P *value greater than 0.01 is considered to be a 'false positive' and is discarded. We remove the edge with the highest *P *value and recalculate the *P *value for affected edges. The process is repeated until no edge has a *P *value > 0.01. We found in analysis of yeast data, this filtering always improves the quality of discovered protein interaction modules.

### Application to simulated yeast protein interaction networks

To compare the performance of our BCD algorithm, the GN algorithm, the ECC algorithm with the original edge clustering coefficient definition (ECC1), and the ECC algorithm with our commonality metric (ECC2), and the MCL algorithm [15], in which the inflation parameter was set to the optimal value 1.8 according to the study [16], we built a test graph on the basis of 198 complexes manually annotated in the MIPS database [18] in a way similar to that used in Brohee and van Helden's study [16]. Briefly, for each manually annotated MIPS complex, an edge was created between each pair of proteins within that complex. The resulting graph (referred to as test graph) contains 1,078 proteins and 9,919 interactions. To evaluate the robustness to false positives and false negatives, we derived 16 altered networks by randomly removing edges from or adding edges to the test graph in various proportions. We then assessed the quality of clustering results on each derived network by different algorithms with each annotated complex. As done in Brohee and van Helden's study [16], we computed a geometric accuracy value and a separation value to estimate the overall correspondence between a clustering result (a set of clusters) and the collection of annotated complexes, where both a high geometric accuracy value and a high separation value indicate good clustering (please see [16] for more details).

**Figure 3:**
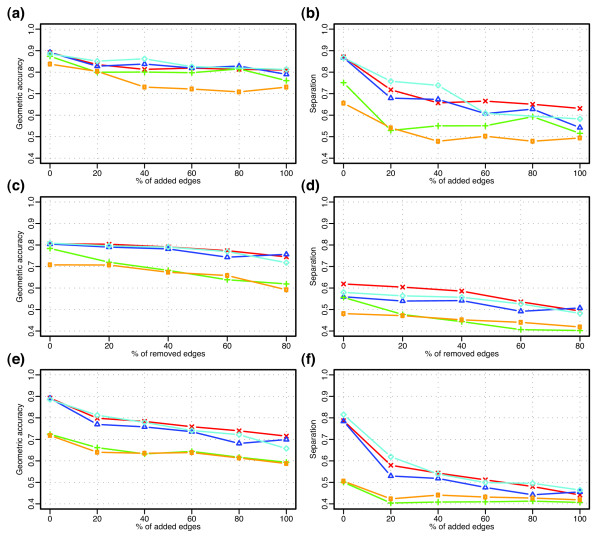
Robustness of the algorithms to random edge addition and removal. Each curve represents the value of accuracy (left panels) or separation (right panels). **(a, b) **Edge addition to the test graph. **(c, d) **Edge removal from an altered graph with 100% of randomly added edges. **(e, f) **Edge addition to an altered graph with 40% of randomly removed edges. Color code: red, BCD; blue, GN; cyan, MCL; orange, ECC with the original edge clustering coefficient; green, ECC with our commonality index.

Figure 3a displays the impact of edge addition on geometric accuracy and Figure 3b show the impact on separation. Clearly, the ECC2 algorithm with our new commonality metric greatly outperforms the ECC1 algorithm with the older edge clustering coefficient measure when the graph is altered with adding edges. In Figure 3c,d, increasing proportions (0%, 20% 40%, 60%, and 80%) of edges are randomly removed from the test graph with prior 100% edge addition. Figure 3e,f show the effect of edge addition on graphs from which 40% of the edges had previously been removed. All curves show similar trends and that BCD and MCL outperform the other three algorithms. The performance of our BCD algorithm is better than that of the MCL algorithm when the graph is more dramatically altered with both edge removal and addition (Figure 3c-f).

### Application to the yeast protein interaction network

We used the yeast protein interaction network from the BioGrid database (version 2.0.24) [26], from which we extracted 36,238 unique interactions among 5,273 yeast proteins. We applied the filtering process to the data and the resulting dataset retained 3,030 yeast proteins and 17,242 high-confidence interactions, which we call the filtered dataset. On both the original and filtered datasets, we tested five algorithms: our BCD algorithm, the GN algorithm, the ECC1 algorithm with its original edge clustering coefficient, the ECC2 algorithm with our commonality metric and the MCL algorithm whenever applicable.

#### Results on a small yeast protein interaction network

Before diving into the entire complex network, we first decomposed a small yeast transcription network with 225 proteins and 1,792 interactions, where known protein interaction modules can be inferred from the annotations of well-studied proteins (Figure 2a). Figure 2b displays a hierarchical decomposition tree by the BCD algorithm (decomposition trees constructed by the other three algorithms are provided in Additional data file 1). Note that there is no decomposition tree for the MCL algorithm.

The proposed definition of protein interaction module works well for both the GN and BCD algorithms because almost all proteins within the same computed protein module do indeed belong to the same known protein complex. Decomposition trees obtained using the ECC1 algorithm and the ECC2 algorithm with our commonality metric are shown in Additional data file 1. They produce irregularly large modules and an excess number of singletons. This suggests that the purely local metric used in the ECC algorithm is not effective. Additional data file 1 also shows good results for both the GN and BCD algorithms that combine global and local metrics. They clearly produce more consistent and robust results.

The BCD algorithm revealed 21 functional modules (Figure 2); all proteins within known protein complexes are also located within the same module, suggesting that the BCD algorithm is superior at unveiling fine structure buried in complex protein interaction networks. The MCL algorithm predicts only 11 clusters from this small yeast transcription network. Several functional modules are grouped together: the three RNA dependent RNA polymerases (A, B, C) and the RNA polymerase II mediator complex are merged into one cluster; the NuA4 histone acetyltransferase complex, the SWR1 complex, and the INO80 chromatin remodeling complex are grouped into one cluster; the TFIIA complex, the Elongator complex, the SAGA histone acetyltransferase complex, and the TFIID complex are grouped into one cluster; and the COMPASS complex and the mRNA cleavage and polyadenylation specificity complex (CPF) are grouped into one cluster. Apparently, the MCL algorithm is inefficient in discovering boundaries between functionally related protein complexes and tends to group them together. The quality of modules obtained using the GN algorithm is not as good; members of four functional modules, transcription factor IIA (TFIIA) [TOA1, TOA2], TFIID [TAF2, TAF3, TAF4, TAF7, TAF8, TAF11, TAF13], nuclear pore-associated [SAC3, CDC31, THP1], and a new one [ABD1, SPT6] predicted by the BCD algorithm, are misplaced. The ECC algorithm has the same tendency to separate peripheral members of the same known protein complex into incorrect protein modules. For instance, in the transcription network, the ECC algorithm disjoins peripheral proteins such as FOB1, RPC10, RRP8 and RPL6B in a very early phase of the decomposition process, causing those derived singletons to be separated from most functional modules. Singletons do not provide useful information for inferring the function of any module. Therefore, the number of singletons generated by an algorithm is an additional indicator of that algorithm's performance: an excess number of singletons indicates poor performance of a particular algorithm. On this small network, the ECC algorithm produces 13 singletons, while the BCD and GN algorithms produce 9 and 3 singletons, respectively. While the difference between the ECC algorithm and the BCD algorithm is only four singletons, those ECC singletons lose their connections with other modules as they are isolated at a much earlier stage of the decomposition process. Although the GN algorithm produces the least number of singletons in the example network, it is at the expense of generating mosaic modules. Similar trends are seen in following experiments of large networks.

We also note that the original ECC1 algorithm performs more poorly than the ECC2 algorithm with our commonality index (Additional data file 1). From now on, we will not discuss the original ECC1 algorithm. When we refer to the ECC algorithm, we mean the ECC algorithm using our commonality index.

#### Results on the global yeast network

In this section, we discuss the results of BCD decomposition of a specific network (yeast), the quality of computed modules, and comparison to MIPS hand-curated protein complex data.

**Figure 4:**
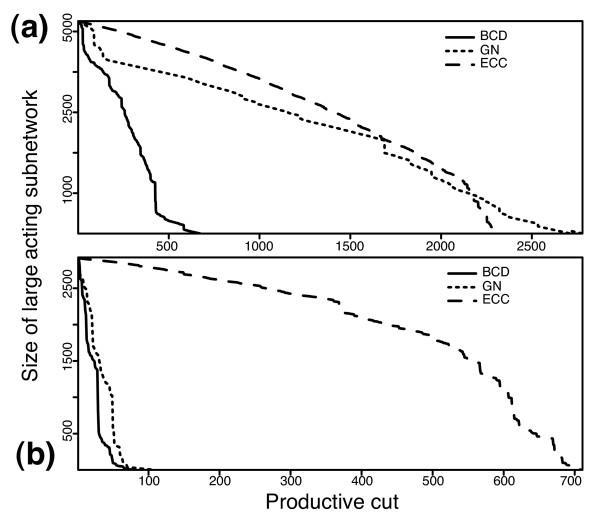
Decomposition curves for the largest sub-networks of two datasets on **(a) **unfiltered data and **(b) **filtered data by the three algorithms. During the decomposition process, the larger connected component and the larger one of its derived sub-networks are always decomposed earlier. The y-axis shows the size of the sub-network under decomposition and the x-axis shows the number of productive cuts so far. A productive cut means the removal of an edge splitting one network into two disconnected parts.

**Figure 5:**
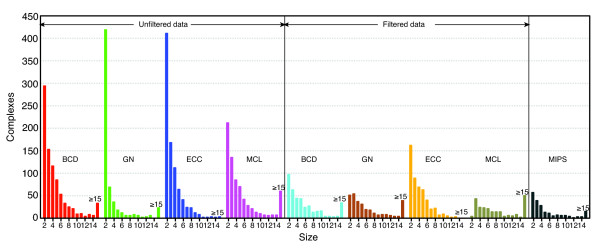
Size distribution of predicted and MIPS protein complexes.

We first studied the decomposition processes by the three algorithms as curves in Figure 4. Each curve displays the size of the current network on which an algorithm acts versus the number of productive cuts thus far. We consider the tendency of network fragmentation due to different algorithms, as measured by the number of productive cuts. Note that most module (complex) finding algorithms are typically applied on connected components of network. A productive cut is defined as a removal of an edge resulting in two separate subnetworks. On the original dataset, the BCD, GN and ECC algorithms require 674, 2,779, and 2,304 productive cuts to split the largest connected component of 5,257 nodes into smaller pieces, which means, on average, the algorithms separate 7.8, 1.9 and 2.3 nodes, respectively, from the largest connected component in each productive cut. On the filtered dataset, the respective algorithms require 80, 107 and 710 productive cuts to split the largest connected component of 2,924 nodes into smaller pieces, which means, on average, the algorithms separate 36.5, 27.3 and 4.1 nodes, respectively, from the largest connected component in each productive cut. The more productive cuts made, the more fragmented the network and the more singletons generated, as shown in Table 1. As stated earlier, a large number of singletons is an indicator of poor performance by a particular algorithm. For both datasets, the BCD algorithm produces the fewest singletons of the three partitioning-type algorithms. The size distributions of predicted protein complexes for each algorithm, including the MCL algorithm, on both datasets are shown in Figure 5. The pattern of predicted complexes generated by all three methods is similar to that of hand-curated MIPS complexes [18], suggesting that the proposed protein module definition is effective.

**Table 1:** Number of predicted complexes and singletons

	Unfiltered		Filtered	
		
Algorithm	Complex	Singleton	Complex	Singleton
BCD	850 (5.0)	991	391 (6.8)	361
GN	614 (4.6)	2,477	297 (8.9)	379
ECC	875 (3.5)	2,214	491 (4.1)	1,021
MCL	703 (7.3)	168	232 (13.0)	3

The average size of complexes is shown in parentheses.

#### Modularity

As a measure of the quality of the protein modules computed, we use modularity (Q) [17], which is a measure of a community structure in a network, measuring the difference between the number of edges falling within groups and the expected number in an equivalent network with edges placed at random. Basically, the higher the modularity, the better the separation. The best clusters are given at the point when the modularity is maximal. Previous studies stopped the decomposition process when the modularity reached its peak value and treated all resulting clusters as communities [17,21]. Applying the modularity criteria on protein interaction networks in this study, however, we found that protein modules obtained in this way tend to be dominated by several very large examples. Nonetheless, the maximal modularity is an objective measure, which is useful for comparing the performance of different algorithms. Table 2 lists the maximal modularities obtained by three algorithms on three networks of different size. The BCD algorithm has the highest Q values for both the transcription network and the unfiltered global network and is very close to the highest Q value of the GN algorithm on the filtered data, suggesting that the BCD algorithm is best in terms of maximal modularity. In particular, on the noisy original data, the maximal modularity Q value by the BCD algorithm is significantly higher than the Q values by the other two algorithms, suggesting the tolerance of data noise by the BCD algorithm is much better than the other algorithms.

**Table 2:** Comparison of modularity coefficients for network decomposition on three networks of varying sizes

		Modularity *Q*		
		
Network	Size *n*	BCD	GN	ECC
Transcription network	225	0.692	0.690	0.637
Filtered global data	3030	0.701	0.717	0.550
Unfiltered global data	5273	0.423	0.340	0.284

#### Overlap with MIPS complexes

We validated the biological significance of our predicted protein modules by comparing the hand-curated protein complexes in the MIPS [27] database with the predicted modules. For each predicted module, we found a best-matching MIPS complex using the method of Spirin and Mirny [22], which finds two complexes with the least probability of random overlap using the hypergeometric distribution:

$$P_{overlap}=\frac{\left(\begin{array}{c} n\\ k \end{array}\right)\left(\begin{array}{c} N-n\\ m-k \end{array}\right)}{\left(\begin{array}{c} N\\ m \end{array}\right)}$$

where N is the total number in the protein interaction network, n and m are the sizes of two complexes, and k is the number of common nodes. Table 3 presents the overlap (the number of common proteins divided by the number of proteins in the best-matching MIPS complexes) between predicted and MIPS complexes. In terms of the absolute number of clusters that overlap 100% with MIPS complexes, the BCD is the best one on the unfiltered dataset, while the MCL algorithm is the best on the filtered dataset. In terms of the percentage of clusters that overlap 100% with MIPS complexes, the MCL algorithm always performs better than the other three. However, we found the size of predicted clusters might affect the number. The larger a cluster is, the more likely it contains all members of an overlapping MIPS complex. From both Table 1 and Figure 5, the MCL algorithm produces a greater number of larger clusters than the other three algorithms, which was seen previously in the small yeast transcription network.

**Table 3:** Comparison of predicted protein complexes with known MIPS complexes

	BCD	GN	ECC	MCL
**Unfiltered**				
100%*	59 (6.9^†^)	27 (4.4)	56 (6.4)	53 (7.5)
>50%	65 (7.6)	51 (8.3)	56 (6.4)	63 (9.0)
>0%	125 (14.7)	92 (15.0)	122 (13.9)	153 (21.8)
No overlap	601 (70.7)	444 (72.3)	641 (73.3)	434 (61.7)
Accuracy^‡^	0.70	0.64	0.62	0.65
Separation^‡^	0.21	0.16	0.20	0.27
				
**Filtered**				
100%	53 (13.6)	45 (15.2)	50 (10.2)	67 (28.9)
>50%	46 (11.8)	38 (12.8)	49 (10.0)	24 (10.3)
>0%	83 (21.2)	66 (22.2)	120 (24.4)	50 (21.6)
No overlap	209 (53.5)	148 (49.8)	272 (55.4)	91 (39.2)
Accuracy	0.73	0.71	0.61	0.67
Separation	0.29	0.28	0.26	0.38

*The overlap is defined as the percentage of proteins in the best-matching MIPS complexes in a predicted cluster. Complexes with only one protein are excluded in this analysis. ^†^The percentage of total predicted protein complexes. ^‡^The geometric accuracy and separation according to [16].

Therefore, to estimate the overall correspondence between a resulting cluster by one approach and the collection of annotated complexes, we computed the geometric accuracy and separation as done in the described study [16]. The results are shown in Table 3. Clearly, the BCD algorithm achieves better accuracy than the other three algorithms on both unfiltered and filtered datasets. In terms of separation, it is the MCL algorithm that performs best among the four algorithms on both datasets (Table 3).

#### GO term enrichment

In addition to the MIPS protein complex dataset we also evaluated the biological significance of predicted protein modules by quantifying GO term co-occurrences using the SGD GO Term Finder [28]. The GO Term Finder calculates a *P *value that reflects the probability of observing by chance the co-occurrence of proteins with a given GO annotation in a certain complex based on a binomial distribution. The lower the *P *value of a GO term, the more statistically significant a complex is enriched in the GO term. Table 4 lists the percentage of predicted protein modules whose *P *value falls within *P *< e-15, [e-15, e-10], [e-10, e-5] and [e-5, 1]. There are more BCD complexes in terms of absolute number with *P *value less than 1e-15 on both the unfiltered and filtered datasets.

**Table 4:** Predicted protein complexes of size ≥3 enriched in GO terms

	Unfiltered				Filtered			
		
	<e-15	e-15 to e-10	e-10 to e-5	e-5 to 1	<e-15	e-15 to e-10	e-10 to e-5	e-5 to 1
BCD	58 (10.4)	41 (7.4)	118 (21.2)	339 (61.0)	62 (21.1)	38 (13.0)	86 (29.3)	108 (36.7)
GN	47 (24.1)	23 (11.8)	43 (22.1)	82 (42.1)	60 (24.4)	32 (13.0)	66 (26.8)	88 (35.8)
ECC	47 (10.1)	48 (10.3)	120 (25.9)	249 (53.7)	45 (13.7)	55 (16.7)	114 (34.7)	115 (35.0)
MCL	55 (11.2)	31 (6.3)	96 (19.6)	309 (62.9)	55 (24.1)	33 (14.5)	62 (27.2)	78 (34.2)

The number in parentheses indicates the percentage of total complexes in that category.

#### Prediction of possible novel protein complexes

**Figure 6:**
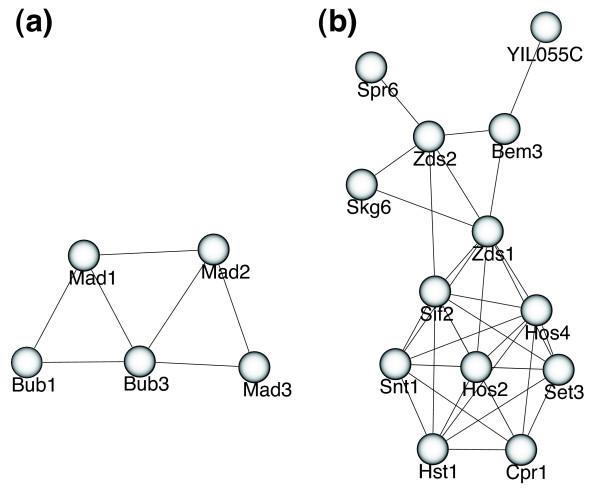
Examples of modules where the GO terms are greatly enriched. **(a) **A five-member module of the spindle-assembly checkpoint complex that is crucial in the checkpoint mechanism required to prevent cell cycle progression into anaphase in the presence of spindle damage. **(b) **A thirteen member module including members from the Set3 histone deacetylase complex (Set3, Hos2, Snt1, Hos4, Hst1, Sif2), proteins involved in telomere silencing (Zds1, Zds2 and Skg6), proteins related to sporulation (Spr6 and Bem3), and two other proteins (YIL055C and Cpr1).

The number of predicted protein complexes is larger than the number of known protein complexes compiled in the MIPS complex dataset, and many predicted protein complexes do not overlap with MIPS complexes. Among these unmatched predicted protein complexes, some are likely to be true functional protein modules because the GO terms in these complexes are greatly enriched as indicated by low *P *values. Figure 6 presents two such modules: a five-member module (*P *= 1.9e-12) of a spindle-assembly checkpoint complex that is crucial in the checkpoint mechanism required to prevent cell cycle progression into anaphase in the presence of spindle damage [29] (Figure 6a), and a thirteen-member module (*P *= 9.8e-17) including members from the Set3 histone deacetylase complex (Set3, Hos2, Snt1, Hos4, Hst1, Sif2) [30], proteins involved in telomeric silencing (Zds1, Zds2 and Skg6) [31], proteins related to sporulation (Spr6 and Bem3) [32,33] and two other proteins (YIL055C and Cpr1) (Figure 6b). A complete list of complexes and modules with functional annotation is provided in Additional data files 2 and 3.

Table 5 provides the number of predicted protein modules (4 algorithms, 2 datasets) where either the GO terms are greatly enriched (*P *< 1e-15) or they overlap with MIPS complexes (overlap = 100%). Generally, the protein modules falling within the above two categories can be viewed as functional modules. The BCD algorithm outperforms the other three algorithms in terms of identifying more functional protein modules on the unfiltered dataset. The MCL algorithm predicts more functional protein modules than our BCD algorithm does on the filtered dataset. In addition, all four algorithms predict a substantial number of complexes that do not overlap with MIPS or in which GO term co-occurrences are insignificant. However, these are potentially novel functional complexes for biologists to explore further.

**Table 5:** Predicted protein modules where either GO terms are greatly enriched (*P *< 1e-15) or all members of a best-matching MIPS complex are found (overlap = 100%)

Algorithm	Unfiltered (percentage)	Filtered (percentage)
BCD	95 (11.2*)	90 (23.0)
GN	58 (9.4)	80 (27.0)
ECC	87 (9.9)	83 (16.9)
MCL	84 (11.9)	91 (39.2)

*The percentage of total predicted protein complexes.

#### The effects of filtering false-positive interactions

In all experiments, the results on the filtered data are consistently better than the results on the original data. For example, in Table 3, the non-overlap between computed protein modules by the BCD algorithm and known protein complexes was reduced from 601 for the original data to 209 on the filtered data. In Table 4, the percentage of GO terms with probability <e-10 is always higher in the filtered data than in the original data.

## Discussion

Protein interaction networks are examples of complex systems that are difficult to understand from raw experimental data alone. Methods to organize, filter, extract significant features and display these data are critical to understanding these systems. A number of network partition algorithms have been proposed to find modular structures in protein interaction networks [22,34-39]. Our work is a further development along the network decomposition approach [13,14]. Our main contribution is to combine the global metric with a local metric in the decomposition procedure. We also resolved several critical technical issues. We propose a new commonality metric based on random graph analysis, a clear definition of protein modules utilizing the decomposition tree structure, and a noise filtering algorithm based on random graph analysis. These advances in methodology result in an effective, consistent, and robust algorithm, as demonstrated on both simulated datasets and the experimental yeast interaction data. The protein modules obtained have clear biological functions, as shown in Table 5. Our approach to recover protein interaction modules is fully self-contained, that is, it does not need other input or parameters to identify protein module boundaries. Our test experiments on yeast show that this method can effectively predict protein interaction modules from a complex interaction network. We plan to further automate this algorithm to compute protein interaction modules for a large number of organisms.

## Materials and methods

### Computing geometric accuracy and separation

We computed the geometric accuracy and separation by following the approach described in the study by Brohee and van Helden [16]. Briefly, each clustering result was compared with the annotated complexes by building a contingency table T, where row i corresponds to the i^th ^annotated complex and column j to the j^th ^cluster and the value of a cell T_ij _indicates the number of proteins found in common between complex i and cluster j. The contingency table has n rows (complexes) and m columns (clusters).

#### Accuracy

First, we define complex-wise sensitivity $Sn_{co_{i}}$ as the maximal fraction of protein of complex i that could be found in one cluster by the formula:

$$Sn_{co_{i}}=\max_{j=1}^{m}\left(T_{ij}/N_{i}\right)$$

where N_i _is the number of proteins belonging to complex i. To characterize the general sensitivity of a clustering result, we compute a clustering-wise sensitivity as the weighted average of $Sn_{co_{i}}$ over all complexes by the formula:

$$Sn=\frac{\sum_{i=1}^{n}N_{i}Sn_{co_{i}}}{\sum_{i=1}^{n}N_{i}}.$$

Second, we calculate a cluster-wise positive predictive value $PPV_{cl_{j}}$ as the maximal fraction of proteins of cluster j found in the best-matching complex by the formula:

PPVclj=max⁡i=1n(TijT.j)

where *T*_*j *_is the marginal sum of a column j by:

$$T_{.j}=\sum_{i=1}^{n}T_{ij}$$

To characterize the general PPV (positive predictive value) of a clustering result as a whole, we compute a clustering-wise PPV as the weighted average of $PPV_{cl_{j}}$ over all clusters by:

$$PPV=\frac{\sum_{j=1}^{m}T_{.j}PPV_{cl_{j}}}{\sum_{j=1}^{m}T_{.j}}$$

The geometric accuracy (Acc) indicates the tradeoff between sensitivity and predictive value. It is obtained by computing the geometric mean of the Sn and the PPV by:

$$Acc=\sqrt{Sn\cdot PPV}$$

#### Separation

From the contingency table, we derive relative frequencies with respect to the marginal sums, either per row:

$$F_{row_{ij}}=\frac{T_{ij}}{\sum_{j=1}^{m}T_{ij}}$$

or per column:

$$F_{col_{ij}}=\frac{T_{ij}}{\sum_{i=1}^{n}T_{ij}}$$

We then define the separation as the product of column-wise and row-wise frequencies by:

$$Sep_{ij}=F_{col_{ij}}\cdot F_{row_{ij}}$$

The complex-wise separation $Sep_{co_{i}}$ is calculated as the sum of separation values for a given complex i by:

$$Sep_{co_{i}}=\sum_{j=1}^{m}Sep_{ij}$$

and the cluster-wise separation $Sep_{cl_{j}}$ for cluster j by:

$$Sep_{cl_{j}}=\sum_{i=1}^{n}Sep_{ij}$$

To estimate a clustering result as a whole, complex-wise *Sep*_*co *_and clustering-wise *Sep*_*cl *_values are computed as the average of $Sep_{co_{i}}$ over all complexes, and of $Sep_{cl_{j}}$ over all clusters, respectively:

$$Sep_{co}=\frac{\sum_{i=1}^{n}Sep_{co_{i}}}{n}$$

$$Sep_{cl}=\frac{\sum_{j=1}^{m}Sep_{cl_{j}}}{m}$$

We then compute the geometric separation (Sep) as the geometric mean of *Sep*_*co *_and *Sep*_*cl *_by:

$$Sep=\sqrt{Sep_{co}\cdot Sep_{cl}}$$

## Abbreviations

BCD, Betweenness-Commonality Decomposition; ECC, edge clustering coefficient; GN, Girvan and Newman; GO, Gene Ontology; MIPS, Munich Information Center for Protein Sequences.

## Authors' contributions

CW, CD and SH were involved in the study design. CW developed the method, carried out the study, and wrote the manuscript. QY was involved in the evaluation of the method. All authors revised the manuscript, read, and approved the final manuscript.

## Additional data files

The following additional data are available with the online version of this paper. Additional data file 1 shows hierarchical decomposition trees of a yeast transcriptional sub-network by different algorithms. Additional data file 2 lists predicted protein interaction modules by the BCD algorithm on the unfiltered dataset. Additional data file 3 lists predicted protein interaction modules by the BCD algorithm on the filtered dataset.

## Supplementary Material

Additional data file 1 Hierarchical decomposition trees of a yeast transcriptional sub-network by different algorithms. Click here for file

Additional data file 2 Predicted protein interaction modules by the BCD algorithm on the unfiltered dataset. Click here for file

Additional data file 3 Predicted protein interaction modules by the BCD algorithm on the filtered dataset. Click here for file
