# Supplementary material for: Consistent dissection of the protein interaction network by combining global and local metrics
Source: Genome Biol. 2007 Dec 21;8(12):R271. doi: 10.1186/gb-2007-8-12-r271 (PMC2246273; doi:10.1186/gb-2007-8-12-r271)
Supplement: Additional data file 1 — Hierarchical decomposition trees of a yeast transcriptional sub-network by different algorithms. [file gb-2007-8-12-r271-S1.pdf]

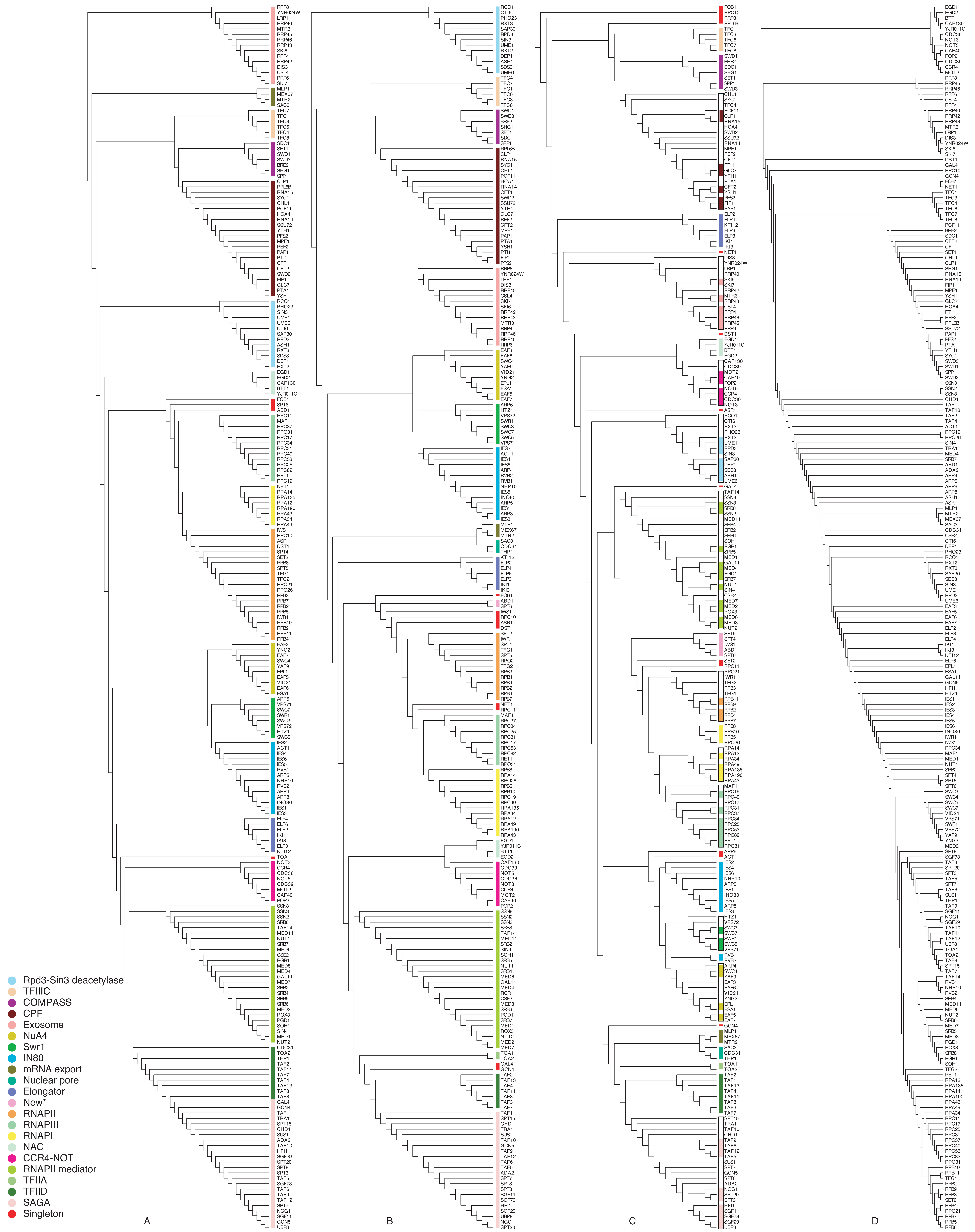

The hierarchical decomposition trees of a yeast transcriptional sub-network containing 225 proteins with 1792 interactions constructed by the GN algorithm (A), the BCD algorithm (B), the ECC algorithm with commonality (C) and the ECC algorithm with edge clustering coefficient (D). Predicted protein modules are highlighted with colored bars. The module names are inferred from their members' annotation information. Singletons are colored red. Note that the tree for the ECC algorithm with edge clustering coefficient (D) is not colored because the resulting cluters are too different from those trees by the other three algorithms.
