# Supplementary material for: Consistent dissection of the protein interaction network by combining global and local metrics
Source: Genome Biol. 2007 Dec 21;8(12):R271. doi: 10.1186/gb-2007-8-12-r271 (PMC2246273; doi:10.1186/gb-2007-8-12-r271)
Supplement: Additional data file 3 — Predicted protein interaction modules by the BCD algorithm on the filtered dataset. [file gb-2007-8-12-r271-S3.pdf]

Predicted protein interaction modules by the BCD algorithm on the filtered dataset. For each predicted module, the first row displays the module id, the enriched GO terms and P-value for that enrichment (if applicable). If the predicted module overlaps with a MIPS complex, the best-matching MIPS complex's name, id and size will be shown on the same row as well. From the second row, the name and annotation of each member of the predicted module are shown in one independent row. If a member of a predicted module is within an overlapping MIPS complex, the name of that member will be highlighted with blue color.

0001	GO_TERM:[mitochondrial outer membrane] P-Value:3.4e-05
YOL009C	[MDM12] Mitochondrial outer membrane protein, required for transmission of mitochondria to daughter cells; exists in a complex with Mmm1p and Mdm10p Null mutant is viable, temperature sensitive, and possesses abnormally large, round mitochondria that are defective for inheritance by daughter buds
YAL010C	[MDM10] Subunit of the mitochondrial sorting and assembly machinery (SAM complex); has a role in assembly of the TOM complex, which mediates protein import through the outer membrane; required for normal mitochondrial morphology and inheritance mitochondrial outer membrane protein Null mutant has short actin cables. Point mutants exhibit giant, spherical mitochondria and are defective for mitochondrial inheritance.
YLL006W	[MMM1] Mitochondrial outer membrane protein required for normal mitochondrial morphology and mtDNA stability; involved in tethering mitochondria to the actin cytoskeleton and in anchoring mtDNA nucleoids mitochondrial outer membrane protein Null mutant is viable, fails to grow on nonfermentable carbon sources, demonstrates abnormal mitochondrial morphology, fails to segregate mitochondria into daughter cells

0002	GO_TERM:[RNA polymerase I transcription factor activity] P-Value:3.9e-09 OVERLAP:[Core Factor (CF)] <510.20> SIZE:4
**YJL025W**	[RRN7] Protein involved in the transcription of 35S rRNA genes by RNA polymerase I; component of the core factor (CF) complex also composed of Rrn11p, Rrn6p and TATA-binding protein yeast Pol I core factor (CF) also composed of Rrn11p, Rrn6p and TATA-binding protein
**YBL014C**	[RRN6] Protein involved in the transcription of 35S rRNA genes by RNA polymerase I; component of the core factor (CF) complex also composed of Rrn11p, Rrn7p and TATA-binding protein yeast Pol I core factor (CF) also composed of Rrn11p, Rrn7p and TATA-binding protein
**YML043C**	[RRN11] Protein required for rDNA transcription by RNA polymerase I, component of the core factor (CF) of rDNA transcription factor, which also contains Rrn6p and Rrn7p rDNA transcription factor component

0003	GO_TERM:[mitochondrial fusion] P-Value:5.8e-10
YBR179C	[FZO1] Mitochondrial integral membrane protein involved in mitochondrial fusion and maintenance of the mitochondrial genome; contains N-terminal GTPase domain Drosophila melanogaster fuzzy onions gene homolog|integral protein of the mitochondrial outer membrane; can be isolated as part of a high molecular weight complex Null mutant is viable, exhibits a petite phenotype and fragmented mitochondrial morphology
YDR470C	[UGO1] Protein of unknown function; outer membrane component of the mitochondrial fusion machinery; Ugo1p bind directly to Fzo1p and Mgm1p and thereby link these two GTPases during mitochondrial fusion outer membrane protein Null mutant is viable but defective in mitochondrial fusion. Null mutant also exhibits fragmentation of mitochondria, loss of mtDNA, and inviability on nonfermentable carbon sources.
YOR211C	[MGM1] Mitochondrial GTPase related to dynamin, present in a complex containing Ugo1p and Fzo1p; required for normal morphology of cristae and for stability of Tim11p; homolog of human OPA1 involved in autosomal dominant optic atrophy GTP-binding domain protein related to dynamin Null mutant is viable, has a reduced number of copies of the mitochondrial chromosome per cell at each cell division, grows slowly on rich media, fails to grow on non-fermentable carbon sources

0004	GO_TERM:[NatC complex] P-Value:1.6e-10
YPR051W	[MAK3] Catalytic subunit of N-terminal acetyltransferase of the NatC type; required for replication of dsRNA virus N-acetyltransferase deficient in maintenance of killer
YCR020C-A	[MAK31] Non-catalytic subunit of N-terminal acetyltransferase of the NatC type; required for replication of dsRNA virus; member of the Sm protein family Mutant exhibits defects in the structural stability of L-A family of dsRNA-containing viral particles.
YEL053C	[MAK10] Non-catalytic subunit of N-terminal acetyltransferase of the NatC type, required for replication of dsRNA virus; expression is glucose-repressible

0005	GO_TERM:[ubiquinone metabolism] P-Value:2.2e-08
YDR204W	[COQ4] Protein with a role in ubiquinone (Coenzyme Q) biosynthesis, possibly functioning in stabilization of Coq7p; located on the matrix face of the mitochondrial inner membrane; component of a mitochondrial ubiquinone-synthesizing complex encodes component of the coenzyme Q biosynthetic pathway Unable to produce ubiquinone, hypersensitivity to polyunsaturated fatty acid treatment
YOL096C	[COQ3] O-methyltransferase, catalyzes two different O-methylation steps in ubiquinone (Coenzyme Q) biosynthesis; component of a mitochondrial ubiquinone-synthesizing complex 3,4-dihydroxy-5-hexaprenylbenzoate methyltransferase Null mutant is viable, fails to grow on H2O2; fails to grow on glycerol

YOR125C	[CAT5] Mitochondrial inner membrane protein directly involved in ubiquinone biosynthesis, essential for several other metabolic pathways including respiration and gluconeogenic gene activation may encode a protein involved in one or more monoxygenase or hydroxylase steps of ubiquinone biosynthesis Null mutant is viable, results in complete loss of glucose derepression affecting gluconeogenic key enzymes. Respiration, but not mitochondrial cytochrome c oxidase activity, are also affected; fails to synthesize ubiquinone

0006	GO_TERM:[transcription] P-Value:1.2e-01
YIL128W	[MET18] DNA repair and TFIIH regulator, required for both nucleotide excision repair (NER) and RNA polymerase II (RNAP II) transcription; involved in telomere maintenance TFIIH regulator Null mutant is viable but is temperature-sensitive, defective in ability to remove UV_induced dimers from nuclear DNA, and shows enhanced UV-induced mutations; extracts from mutant exhibit thermolabile defect in RNA Pol II transcription; methionine auxotroph
YDR267C	[CIA1] Essential protein involved in assembly of cytosolic and nuclear iron-sulfur proteins
YHR122W	

0007	GO_TERM:[ribonuclease H2 complex] P-Value:1.8e-10
YNL072W	[RNH201] Ribonuclease H2 catalytic subunit, removes RNA primers during Okazaki fragment synthesis; cooperates with Rad27p nuclease Null mutant is viable but shows 75% reduction of RNase H activity in cell extracts
YDR279W	[RNH202] Ribonuclease H2 subunit, required for RNase H2 activity
YLR154C	[RNH203] Ribonuclease H2 subunit, required for RNase H2 activity Null: viable. Other phenotypes: required for RNase H2 activity

0008	GO_TERM:[protein kinase activity] P-Value:3.2e-05
YKL126W	[YPK1] Serine/threonine protein kinase required for receptor-mediated endocytosis; involved in sphingolipid-mediated and cell integrity signaling pathways; localized to the bud neck, cytosol and plasma membrane; homolog of mammalian kinase SGK 76.5 kDa serine/threonine protein kinase|similarity to protein kinase C, is 90% identical to Ypk2p Null mutant is viable, slow growing, ypk1 ypk2 double deletion mutants are defective for vegetative growth
YDR490C	[PKH1] Serine/threonine protein kinase involved in sphingolipid-mediated signaling pathway that controls endocytosis; activates Ypk1p and Ykr2p, components of signaling cascade required for maintenance of cell wall integrity; redundant with Pkh2p Null mutant is viable; pkh1, pkh2 double mutant is lethal
YMR104C	[YPK2] Protein kinase with similarityto serine/threonine protein kinase Ypk1p; functionally redundant with YPK1 at the genetic level; participates in a signaling pathway required for optimal cell wall integrity; homolog of mammalian kinase SGK protein kinase

0009	GO_TERM:[ergosterol biosynthesis] P-Value:7.1e-07
YLR100W	[ERG27] 3-keto sterol reductase, catalyzes the last of three steps required to remove two C-4 methyl groups from an intermediate in ergosterol biosynthesis; mutants are sterol auxotrophs 3-keto sterol reductase
YER044C	[ERG28] Endoplasmic reticulum membrane protein, may facilitate protein-protein interactions between the Erg26p dehydrogenase and the Erg27p 3-ketoreductase and/or tether these enzymes to the ER, also interacts with Erg6p Null mutant is viable; random budding in diploid null mutants; null cells have an unusual sterol content.
YHR072W	[ERG7] Lanosterol synthase, an essential enzyme that catalyzes the cyclization of squalene 2,3-epoxide, a step in ergosterol biosynthesis 2,3-oxidosqualene-lanosterol cyclase

0010	GO_TERM:[mitochondrial outer membrane] P-Value:9.5e-03 OVERLAP:[Kornberg's mediator (SRB) complex] <510.40.20> SIZE:21
YPR183W	[DPM1] Dolichol phosphate mannose (Dol-P-Man) synthase of the ER membrane, catalyzes the formation of Dol-P-Man from Dol-P and GDP-Man; required for glycosyl phosphatidylinositol membrane anchoring, O mannosylation, and protein glycosylation dolichol phosphate mannose synthase
**YER179W**	[DMC1] Meiosis-specific protein required for repair of double-strand breaks and pairing between homologous chromosomes; homolog of Rad51p and the bacterial RecA protein meiosis-specific protein related to RecA and Rad51p. Dmc1p colocalizes with Rad51p to discrete subnuclear sites in nuclear spreads during mid prophase, briefly colocalizes with Zip1p, and then disappears by pachytene
YOR285W	

0011	GO_TERM:[NAD metabolism] P-Value:3.9e-04

YLR328W	[NMA1] Nicotinic acid mononucleotide adenylyltransferase, involved in NAD(+) salvage pathway nicotinamide/nicotinic acid mononucleotide adenylyltransferase Null: viable. Other phenotypes: 2 or more copies increase rDNA and telomeric silencing
YGR010W	[NMA2] Nicotinic acid mononucleotide adenylyltransferase, involved in NAD(+) salvage pathway nicotinamide/nicotinic acid mononucleotide adenylyltransferase Null: viable. Other phenotypes: 2 or more copies increase rDNA and telomeric silencing
YLR438W	[CAR2] L-ornithine transaminase (OTAse), catalyzes the second step of arginine degradation, expression is dually-regulated by allophanate induction and a specific arginine induction process; not nitrogen catabolite repression sensitive ornithine aminotransferase Catabolism of arginine defective

0012	GO_TERM:[bud site selection] P-Value:1.1e-05
YOR301W	[RAX1] Protein involved in bud site selection during bipolar budding; localization requires Rax2p; has similarity to members of the insulin-related peptide superfamily
YGR041W	[BUD9] Protein involved in bud-site selection; diploid mutants display a unipolar budding pattern instead of the wild-type bipolar pattern, and bud at the distal pole In null mutants bipolar-budding cells bud preferentially at distal pole
YLR353W	[BUD8] Protein involved in bud-site selection; diploid mutants display a unipolar budding pattern instead of the wild-type bipolar pattern, and bud at the proximal pole A bud8 bud9 double mutant buds almost exclusively from the proximal pole

0013	GO_TERM:[meiotic DNA double-strand break formation] P-Value:3.5e-08
YLR329W	[REC102] Protein involved in early stages of meiotic recombination; required for chromosome synapsis; forms a complex with Rec104p and Spo11p necessary during the initiation of recombination 23 kDa protein containing a putative leucine zipper|meiosis specific recombination protein Reduced meiotic recombination; inviable spores; mutant is rescued by spo13 and is epistatic to rad52
YHL022C	[SPO11] Meiosis-specific protein that initiates meiotic recombination by catalyzing the formation of double-strand breaks in DNA via a transesterification reaction; required for homologous chromosome pairing and synaptonemal complex formation early meiosis-specific recombination protein
YHR157W	[REC104] Protein involved in early stages of meiotic recombination; required for meiotic crossing over; forms a complex with Rec102p and Spo11p necessary during the initiation of recombination meiosis-specific protein Null mutant is viable, rec104 mutants exhibit reduced meiotic DNA recombination, executes meiosis I early; rec104 is rescued by spo13 and is epistatic to rad52 spo13

0014	GO_TERM:[mitochondrion] P-Value:3.6e-02
YML091C	[RPM2] Protein component of mitochondrial RNase P, along with the mitochondrially-encoded RNA subunit RPM1; Rnase P removes 5' extensions from tRNA precursors; Rpm2p is also involved in maturation of RPM1 and in translation of mitochondrial mRNAs mitochondrial RNase P subunit Null mutant is viable, respiratory deficient, accumulate mitochondrial tRNA precursors with 5' extensions, arrest after 25 generations on fermentable media. Spontaneous mutations that suppress arrest occur at approx 9E-6. Resultant mutants do not grow on nonfermentable carbon sources.
YJL023C	[PET130] Protein required for respiratory growth
YPR116W	

0015	GO_TERM:[endonuclease activity] P-Value:1.1e-03
YMR099C	
YJL208C	[NUC1] Major mitochondrial nuclease, has RNAse and DNA endo- and exonucleolytic activities; has a role in mitochondrial recombination nuclease
YKR079C	[TRZ1] tRNase Z, involved in RNA processing, has two putative nucleotide triphosphate-binding motifs (P-loop) and a conserved histidine motif, homolog of the human candidate prostate cancer susceptibility gene ELAC2 tRNA 3' processing endoribonuclease

0016	GO_TERM:[response to stimulus] P-Value:2.2e-02
YNL164C	[IBD2] Component of the BUB2-dependent spindle checkpoint pathway, interacts with Bfa1p and functions upstream of Bub2p and Bfa1p Null: viable, sensitive to benomyl
YKL075C	
YNL091W	[NST1] Protein of unknown function, mediates sensitivity to salt stress; interacts physically with the splicing factor Msl1p and also displays genetic interaction with MSL1

0017	GO_TERM:[response to stress] P-Value:5.0e-04
YOR043W	[WHI2] Protein required, with binding partner Psr1p, for full activation of the general stress response, possibly through Msn2p dephosphorylation; regulates growth during the diauxic shift; negative regulator of G1 cyclin expression
YLL010C	[PSR1] Plasma membrane associated protein phosphatase involved in the general stress response; required along with binding partner Whi2p for full activation of STRE-mediated gene expression, possibly through dephosphorylation of Msn2p Mutant is sensitive to sodium ions.
YLR019W	[PSR2] Functionally redundant Psr1p homolog, a plasma membrane phosphatase involved in the general stress response; required with Psr1p and Whi2p for full activation of STRE-mediated gene expression, possibly through dephosphorylation of Msn2p Mutant is sensitive to sodium ions.

0018	GO_TERM:[meiosis] P-Value:3.3e-02
YGL036W	
YGL192W	[IME4] Probable mRNA N6-adenosine methyltransferase that is required for IME1 transcript accumulation and for sporulation; expression is induced in starved MATa/MAT alpha diploid cells methyltransferase Homozygous mutant diploid cannot accumulate IME1 mRNA during early stages of meiosis and cannot sporulate
YBR057C	[MUM2] Cytoplasmic protein essential for meiotic DNA replication and sporulation; interacts with Orc2p, which is a component of the origin recognition complex Mutant is sporualtion defective and fails to perform premiotic DNA synthesis; overexpression suppresses a TOR2 allele
YCL055W	[KAR4] Transcription factor required for induction of KAR3 and CIK1 during mating, also required during meiosis; exists in two forms, a slower-migrating form more abundant during vegetative growth and a faster-migrating form induced by pheromone involved in karyogamy|transcription factor Defective in pheromone-induced expression of KAR3 and CIK1; therefore, defective in nuclear fusion because of defect in microtubule-dependent movement of nuclei; also required for meiosis

0019	GO_TERM:[transposition, RNA-mediated] P-Value:1.4e-07
YCL020W	
YCL019W	
YDR261W-A	
YDR261W-B	

0020	GO_TERM:[error-free DNA repair] P-Value:1.4e-13
YDR078C	[SHU2] Protein of unassigned function involved in mutation suppression, important for error-free repair of spontaneous and induced DNA lesions to protect the genome from mutation; associates with Shu1p, Psy3p, and Csm2p Null: MMS sensitive
YIL132C	[CSM2] Protein required for accurate chromosome segregation during meiosis Null: missegregates chromosomes in meiosis
YHL006C	[SHU1] Protein of unassigned function involved in mutation suppression, important for error-free repair of spontaneous and induced DNA lesions to protect the genome from mutation; associates with Shu2p, Psy3p, and Csm2p Null: Null mutant is viable and MMS sensitive, suppresses HU sensitivity of certain other mutations
YLR376C	[PSY3] Protein of unknown function; deletion results in a mutator phenotype suggesting a role for this protein as a mutational suppressor; deletion increases sensitivity to anticancer drugs oxaliplatin and cisplatin but not mitomycin C

0021	GO_TERM:[protein tyrosine phosphatase activity] P-Value:1.7e-04
YNL056W	[OCA2] Cytoplasmic protein required for replication of Brome mosaic virus in S. cerevisiae, which is a model system for studying replication of positive-strand RNA viruses in their natural hosts
YNL032W	[SIW14] Tyrosine phosphatase that plays a role in actin filament organization and endocytosis; localized to the cytoplasm tyrosine phosphatase Null mutant fails to show cell cycle arrest upon nutrient starvation, is sensitive to 5mM caffeine and 1M NaCL, and shows delocalized actin upon nutrient starvation; synthetically lethal with whi2, on minimal medium only
YNL099C	[OCA1] Putative protein tyrosine phosphatase, required for cell cycle arrest in response to oxidative damage of DNA
YCR095C	[YCR095C] Cytoplasmic protein required for replication of Brome mosaic virus in S. cerevisiae, which is a model system for studying replication of positive-strand RNA viruses in their natural hosts

YHL029C	[YHL029C] Cytoplasmic protein required for replication of Brome mosaic virus in S. cerevisiae, which is a model system for studying replication of positive-strand RNA viruses in their natural hosts

0022	GO_TERM:[translation regulator activity] P-Value:2.6e-11
YER153C	[PET122] Specific translational activator for the COX3 mRNA that acts together with Pet54p and Pet494p; located in the mitochondrial inner membrane translational activator of cytochrome C oxidase subunit III petite; unable to grow on non-fermentable carbon sources
YLR067C	[PET309] Specific translational activator for the COX1 mRNA, also influences stability of intron-containing COX1 primary transcripts; located in the mitochondrial inner membrane petite; unable to grow on non-fermentable carbon sources
YGR222W	[PET54] Protein required for splicing of the COX1 intron AI5 beta; also specifically required, together with Pet122p and Pet494p, for translation of the COX3 mRNA; located in the mitochondrial inner membrane petite; unable to grow on non-fermentable carbon sources
YMR257C	[PET111] Specific translational activator for the COX2 mRNA, located in the mitochondrial inner membrane translational activator of cytochrome C oxidase subunit II
YNR045W	[PET494] Specific translational activator for the COX3 mRNA that acts together with Pet54p and Pet122p; located in the mitochondrial inner membrane translational activator of cytochrome C oxidase petite; unable to grow on non-fermentable carbon sources

0023	GO_TERM:[pyridoxine metabolism] P-Value:6.9e-22
YFL060C	[SNO3] Protein of unknown function, nearly identical to Sno2p; expression is induced before the diauxic shift and also in the absence of thiamin
YNL334C	[SNO2] Protein of unknown function, nearly identical to Sno3p; expression is induced before the diauxic shift and also in the absence of thiamin
YMR095C	[SNO1] Protein of unconfirmed function, involved in pyridoxine metabolism; expression is induced during stationary phase; forms a putative glutamine amidotransferase complex with Snz1p, with Sno1p serving as the glutaminase Null mutant is viable, sensitive to 6-azauracil and methylene blue.
YMR322C	[SNO4] Possible chaperone and cysteine protease with similarity to E. coli Hsp31 and S. cerevisiae Hsp31p, Hsp32p, and Hsp33p; member of the DJ-1/ThiJ/PfpI superfamily; may have a role in pyridoxine metabolism
YNL333W	[SNZ2] Member of a stationary phase-induced gene family; transcription of SNZ2 is induced prior to diauxic shift, and also in the absence of thiamin in a Thi2p-dependent manner; forms a coregulated gene pair with SNO2; interacts with Thi11p hypersporulation
YFL059W	[SNZ3] Member of a stationary phase-induced gene family; transcription of SNZ2 is induced prior to diauxic shift, and also in the absence of thiamin in a Thi2p-dependent manner; forms a coregulated gene pair with SNO3 hypersporulation
YMR096W	[SNZ1] Protein involved in vitamin B6 biosynthesis; member of a stationary phase-induced gene family; coregulated with SNO1; interacts with Sno1p and with Yhr198p, perhaps as a multiprotein complex containing other Snz and Sno proteins highly conserved 35 kDa protein that shows increased expression after entry into stationary phase Null mutant is viable, sensitive to 6-azauracil and methylene blue.

0024	GO_TERM:[cell cycle arrest in response to pheromone] P-Value:1.4e-16 OVERLAP:[cytoplasmic ribosomal large subunit] <500.40.10> SIZE:81
YMR319C	[FET4] Low-affinity Fe(II) transporter of the plasma membrane low affinity Fe2+ transport protein Mutant lacks low affinity Fe(II) transport but has more active high affinity Fe(II) transport activity
YNL127W	[FAR11] Protein involved in G1 cell cycle arrest in response to pheromone, in a pathway different from the Far1p-dependent pathway; interacts with Far3p, Far7p, Far8p, Far9p, and Far10p Null: Defective for pheromone-induced G1 arrest
**YHL001W**	[RPL14B] Protein component of the large (60S) ribosomal subunit, nearly identical to Rpl14Ap and has similarity to rat L14 ribosomal protein ribosomal protein L14B
YLR238W	[FAR10] Protein involved in G1 cell cycle arrest in response to pheromone, in a pathway different from the Far1p-dependent pathway; interacts with Far3p, Far7p, Far8p, Far9p, and Far11p; potential Cdc28p substrate Null: Defective for pheromone-induced G1 arrest
YFR008W	[FAR7] Protein involved in G1 cell cycle arrest in response to pheromone, in a pathway different from the Far1p-dependent pathway; interacts with Far3p, Far8p, Far9p, Far10p, and Far11p Null: Defective for pheromone-induced G1 arrest
YDR200C	[VPS64] Cytoplasmic protein required for cytoplasm to vacuole targeting of proteins; forms a complex with Far3p, Far7p, Far10p, and Far11p that is involved in pheromone-induced cell cycle arrest; also localized to the endoplasmic reticulum membrane Null mutant secretes CPY.
YMR029C	[FAR8] Protein involved in G1 cell cycle arrest in response to pheromone, in a pathway different from the Far1p-dependent pathway; interacts with Far3p, Far7p, Far9p, Far10p, and Far11p Null: Defective for pheromone-induced G1 arrest
YMR052W	[FAR3] Protein involved in G1 cell cycle arrest in response to pheromone, in a pathway different from the Far1p-dependent pathway; interacts with Far7p, Far8p, Far9p, Far10p, and Far11p Null mutant does not arrest in G1 in response to pheromone but does have an intact signal transduction pathway leading to FAR1 transcriptional induction

0025	GO_TERM:[negative regulation of gluconeogenesis] P-Value:4.2e-19
YDL176W	
YDR255C	[RMD5] Cytosolic protein required for sporulation; also required for the ubiquitination of the gluconeogenetic enzyme fructose-1,6-bisphosphatase, which is degraded rapidly after the switch from gluconeogenesis to glycolysis
YBR105C	[VID24] Peripheral membrane protein located at Vid (vacuole import and degradation) vesicles; regulates fructose-1,6-bisphosphatase (FBPase) targeting to the vacuole; involved in proteasome-dependent catabolite degradation of FBPase peripheral vesicle membrane protein Null mutant is viable, defective in fructose-1,6-bisphosphatase dergadation
YIL017C	[VID28] Protein involved in proteasome-dependent catabolite degradation of fructose-1,6-bisphosphatase (FBPase); localized to the nucleus and the cytoplasm
YIL097W	[FYV10] Protein of unknown function, required for survival upon exposure to K1 killer toxin; involved in proteasome-dependent catabolite inactivation of fructose-1,6-bisphosphatase; contains CTLH domain Null mutant is viable but exhibits K1 killer toxin hypersensitivity.
YBL049W	[MOH1] Protein of unknown function, has homology to kinase Snf7p; not required for growth on nonfermentable carbon sources; essential for viability in stationary phase
YCL039W	[GID7] Protein of unknown function, involved in proteasome-dependent catabolite inactivation of fructose-1,6-bisphosphatase; contains six WD40 repeats; computational analysis suggests that Gid7p and Moh1p have similar functions
YGL227W	[VID30] Protein involved in proteasome-dependent catabolite degradation of fructose-1,6-bisphosphatase (FBPase); shifts the balance of nitrogen metabolism toward the production of glutamate; localized to the nucleus and the cytoplasm Null mutant is viable but exhibits vacuolar degradation of cytosolic proteins; mutants are also sensitive to starvation.
YMR135C	[GID8] Protein of unknown function, involved in proteasome-dependent catabolite inactivation of fructose-1,6-bisphosphatase; contains LisH and CTLH domains, like Vid30p; dosage-dependent regulator of START

0026	GO_TERM:[signal recognition particle (sensu Eukaryota)] P-Value:4.6e-17 OVERLAP:[Signal recognition particle (SRP)] <520.40> SIZE:6
YGR250C	
YER155C	[BEM2] Rho GTPase activating protein (RhoGAP) involved in the control of cytoskeleton organization and cellular morphogenesis; required for bud emergence rho GTPase activating protein (GAP) randomized bud-site selection at 26 degrees C and defective bud emergence and growth at 37 degrees C
YDL051W	[LHP1] RNA binding protein required for maturation of tRNA and snRNA precursors; acts as a molecular chaperone for RNAs transcribed by polymerase III; homologous to human La (SS-B) autoantigen
**YML105C**	[SEC65] Subunit of the signal recognition particle (SRP), involved in protein targeting to the ER; interacts with Srp54p; homolog of mammalian SRP19
**YKL122C**	[SRP21] Subunit of the signal recognition particle (SRP), which functions in protein targeting to the endoplasmic reticulum membrane; not found in mammalian SRP; forms a pre-SRP structure in the nucleolus that is translocated to the cytoplasm signal recognition particle component Null mutant is viable, associated with slow cell growth and inefficient protein translocation across the ER membrane
**YPL210C**	[SRP72] Core component of the signal recognition particle (SRP) ribonucleoprotein (RNP) complex that functions in targeting nascent secretory proteins to the endoplasmic reticulum (ER) membrane signal recognition particle component Null mutant is viable, associated with slow cell growth and inefficient protein translocation across the ER membrane
**YPR088C**	[SRP54] Signal recognition particle (SRP) subunit (homolog of mammalian SRP54); contains the signal sequence-binding activity of SRP, interacts with the SRP RNA, and mediates binding of SRP to signal receptor; contains GTPase domain
**YDL092W**	[SRP14] Signal recognition particle (SRP) subunit, interacts with the RNA component of SRP to form the Alu domain, which is the region of SRP responsible for arrest of nascent chain elongation during membrane targeting; homolog of mammalian SRP14
**YPL243W**	[SRP68] Core component of the signal recognition particle (SRP) ribonucleoprotein (RNP) complex that functions in targeting nascent secretory proteins to the endoplasmic reticulum (ER) membrane signal recognition particle component Null mutant is viable, associated with slow cell growth and inefficient protein translocation across the ER membrane

0027	
YAL036C	[RBG1] Member of the DRG family of GTP-binding proteins; interacts with translating ribosomes and with Tma46p
YCR038C	[BUD5] GTP/GDP exchange factor for Rsr1p (Bud1p) required for both axial and bipolar budding patterns; mutants exhibit random budding in all cell types GTP/GDP exchange factor for Rsr1 protein bud5 mutants select bud sites randomly

0028	GO_TERM:[dodecenoyl-CoA delta-isomerase activity] P-Value:1.9e-07

YLR284C	[ECI1] Peroxisomal delta3,delta2-enoyl-CoA isomerase, hexameric protein that converts 3-hexenoyl-CoA to trans-2-hexenoyl-CoA, essential for the beta-oxidation of unsaturated fatty acids, oleate-induced d3,d2-Enoyl-CoA Isomerase Null mutant is viable but fails to metabolize unsaturated fatty acids
YOR180C	[DCI1] Peroxisomal delta(3,5)-delta(2,4)-dienoyl-CoA isomerase, involved in fatty acid metabolism, contains peroxisome targeting signals at amino and carboxy termini delta(3,5)-delta(2,4)-dienoyl-CoA isomerase

0029	GO_TERM:[peroxisome] P-Value:5.1e-08
YGR239C	[PEX21] Part of a two-member peroxin family (Pex18p and Pex21p) specifically required for peroxisomal targeting of the Pex7p peroxisomal signal recognition factor and PTS2-type peroxisomal matrix proteins peroxin
YDR142C	[PEX7] Peroxisomal signal receptor for the N-terminal nonapeptide signal (PTS2) of peroxisomal matrix proteins; WD repeat protein; defects in human homolog cause lethal rhizomelic chondrodysplasia punctata (RCDP) beta-transducin-related (WD-40) protein family Mutant is defective in assembling specific proteins into peroxisomes (assembles catalase and acyl-CoA oxidase but not thiolase) and cannot utilize oleic acid
YHR160C	[PEX18] Part of a two-member peroxin family (Pex18p and Pex21p) specifically required for peroxisomal targeting of the Pex7p peroxisomal signal recognition factor and PTS2 peroxisomal matrix proteins peroxin Null mutant is viable but has reduced growth on oleate, partial impairment of peroxisome biogenesis
YIL160C	[POT1] 3-ketoacyl-CoA thiolase with broad chain length specificity, cleaves 3-ketoacyl-CoA into acyl-CoA and acetyl-CoA during beta-oxidation of fatty acids 3-oxoacyl CoA thiolase Null mutant is viable, unable to use oleic acid as a carbon source

0030	GO_TERM:[peroxisome organization and biogenesis] P-Value:5.9e-32
YAL055W	[PEX22] Putative peroxisomal membrane protein required for import of peroxisomal proteins, functionally complements a Pichia pastoris pex22 mutation Null mutant is viable and oleate minus
YGR133W	[PEX4] Peroxisomal ubiquitin conjugating enzyme required for peroxisomal matrix protein import and peroxisome biogenesis ubiquitin-conjugating protein family
YOL147C	[PEX11] Peroxisomal membrane protein required for peroxisome proliferation and medium-chain fatty acid oxidation, most abundant protein in the peroxisomal membrane, regulated by Adr1p and Pip2p-Oaf1p, promoter contains ORE and UAS1-like elements peroxin|peroxisomal membrane protein
YDR329C	[PEX3] Peroxisomal membrane protein (PMP) required to recruit Pex19p chaperone to peroxisomes; plays selective, essential, direct role in PMP import as a docking factor for Pex19p 48 kDa peroxisomal integral membrane protein Mutant lacks peroxisomes and shows cytosolic mislocalization of peroxisomal matrix enzymes
YOL044W	[PEX15] Phosphorylated tail-anchored type II integral peroxisomal membrane protein required for peroxisome biogenesis, cells lacking Pex15p mislocalize peroxisomal matrix proteins to cytosol, overexpression results in impaired peroxisome assembly 44 kDa phosphorylated integral peroxisomal membrane protein
YDL065C	[PEX19] Chaperone and import receptor for newly-synthesized class I peroxisomal membrane proteins (PMPs), binds PMPs in the cytoplasm and delivers them to the peroxisome for subsequent insertion into the peroxisomal membrane 40 kDa farnesylated protein associated with peroxisomes mutant lacks morphologically recognizable peroxisomes and shows mislocalization of peroxisomal matrix proteins
YDR265W	[PEX10] RING finger peroxisomal membrane peroxin required for peroxisomal matrix protein import, interacts with Pex12p, links ubiquitin-conjugating Pex4p to protein import machinery; mutations in human homolog cause a variety of peroxisomal disorders C3HC4 zinc-binding integral peroxisomal membrane protein|peroxin mutant lacks morphologically recognizable peroxisomes and shows cytosolic mislocalization of peroxisomal matrix proteins
YGR077C	[PEX8] Intraperoxisomal organizer of the peroxisomal import machinery, tightly associated with the lumenal face of the peroxisomal membrane, essential for peroxisome biogenesis, binds PTS1-signal receptor Pex5p peroxisome associated protein containing a PTS1 signal
YJL210W	[PEX2] RING-finger peroxin, peroxisomal membrane protein with a C-terminal zinc-binding RING domain, forms putative translocation subcomplex with Pex10p and Pex12p which functions in peroxisomal matrix protein import CH3HC4 zinc-binding integral peroxisomal membrane protein|peroxin Null mutant is viable but lacks morphologically recognizable peroxisomes and shows cytosolic mislocalization of peroxisomal matrix proteins
YMR026C	[PEX12] RING-finger peroxisomal membrane peroxin that plays an essential role in peroxisome biogenesis and peroxisomal matrix protein import, forms translocation subcomplex with Pex2p and Pex10p C3HC4 zinc-binding integral peroxisomal membrane protein mutant lacks morphologically recognizable peroxisomes and shows cytosolic mislocalization of peroxisomal matrix proteins
YLR191W	[PEX13] Integral peroxisomal membrane receptor for the PTS1 peroxisomal matrix protein signal recognition factor Pex5p, required for the translocation of peroxisomal matrix proteins, also interacts with Pex7p and Pex14p, contains a C-terminal SH3 domain contains Src homology 3 (SH3) domain Defective in peroxisome assembly
YNL214W	[PEX17] Peroxisomal membrane protein component of the peroxisomal translocation machinery, required for peroxisome biogenesis, binds Pex14p peroxin mutant lacks morphologically recognizable peroxisomes and shows mislocalization of peroxisomal matrix proteins

YDR244W	[PEX5] Peroxisomal membrane signal receptor for C-terminal tripeptide signal sequence (PTS1) of peroxisomal matrix proteins, required for peroxisomal matrix protein import, tetratricopeptide repeat protein, also involved in PTS1-independent import 69 kDa protein containing tetratricopeptide repeat (TPR)|peroxin Null mutant is viable but accumulates peroxisomal, leaflet-like membrane structures and exhibits deficient import of some peroxisomal matrix enzymes, particularly proteins with an SKL-like import signal
YGL153W	[PEX14] Peroxisomal membrane protein that is a central component of the peroxisomal protein import machinery, interacts with PTS1 (Pex5p) and PTS2 (Pex7p) peroxisomal matrix protein signal recognition factors and membrane receptor Pex13p peroxin Null mutant is viable but is unable to grow on oleate and lacks peroxisomes

0031	GO_TERM:[ubiquinol-cytochrome-c reductase activity] P-Value:2.5e-04 OVERLAP:[Cytochrome bc1 complex (Ubiquinol-cytochrome c reductase complex, complex III)] <420.30> SIZE:10
YBR018C	[GAL7] Galactose-1-phosphate uridyl transferase, synthesizes glucose-1-phosphate and UDP-galactose from UDP-D-glucose and alpha-D-galactose-1-phosphate in the second step of galactose catabolism galactose-1-phosphate uridyl transferase Null mutant is viable and cannot utilize galactose.
YPR054W	[SMK1] Middle sporulation-specific mitogen-activated protein kinase (MAPK) required for spore morphogenesis MAP kinase smk1 asci are defective in organizing spore wall assembly and display enhanced sensitivity to enzymatic digestion, heat shock, and ether
YNL177C	[MRPL22] Mitochondrial ribosomal protein of the large subunit
YMR036C	[MIH1] Protein tyrosine phosphatase involved in cell cycle control; regulates the phosphorylation state of Cdc28p; homolog of S. pombe cdc25 protein phosphatase Null mutant is viable Short G2 delay
**YBL045C**	[COR1] Core subunit of the ubiquinol-cytochrome c reductase complex (bc1 complex), which is a component of the mitochondrial inner membrane electron transport chain coenzyme QH2 cytochrome c reductase 44 kDa core protein subunit deficiency in cytochrome b; slow growth on glycerol
**YPR191W**	[QCR2] Subunit 2 of the ubiquinol cytochrome-c reductase complex, which is a component of the mitochondrial inner membrane electron transport chain; transcription is regulated by Hap1p, Hap2p/Hap3p, and heme 40 kDa ubiquinol cytochrome-c reductase core protein 2 Null mutant is viable and grows slowly on glycerol

0032	GO_TERM:[organellar large ribosomal subunit] P-Value:1.4e-56 OVERLAP:[mitochondrial ribosomal large subunit] <500.60.10> SIZE:44
**YGR076C**	[MRPL25] Mitochondrial ribosomal protein of the large subunit Null mutant is viable, cells become Pet-
**YOR150W**	[MRPL23] Mitochondrial ribosomal protein of the large subunit
**YBR122C**	[MRPL36] Mitochondrial ribosomal protein of the large subunit; overproduction suppresses mutations in the COX2 leader peptide-encoding region
**YPL173W**	[MRPL40] Mitochondrial ribosomal protein of the large subunit
**YLR312W-A**	[MRPL15] Mitochondrial ribosomal protein of the large subunit
**YKL167C**	[MRP49] Mitochondrial ribosomal protein of the large subunit, not essential for mitochondrial translation 16 kDa mitochondrial ribosomal large subunit protein Null mutant is viable, cold-sensitive, respiration deficient, defective in assembly of stable 54S ribosomal subunits
**YMR193W**	[MRPL24] Mitochondrial ribosomal protein of the large subunit
**YKR006C**	[MRPL13] Mitochondrial ribosomal protein of the large subunit, not essential for mitochondrial translation
**YKR085C**	[MRPL20] Mitochondrial ribosomal protein of the large subunit Null mutant is viable; shows loss of mitochondrial function, instability of mitochondrial DNA
**YDR116C**	[MRPL1] Mitochondrial ribosomal protein of the large subunit
**YMR024W**	[MRPL3] Mitochondrial ribosomal protein of the large subunit
**YNL185C**	[MRPL19] Mitochondrial ribosomal protein of the large subunit
**YDR405W**	[MRP20] Mitochondrial ribosomal protein of the large subunit Null mutant is viable, becomes [rho-] or [rho0]
**YCR071C**	[IMG2] Mitochondrial ribosomal protein of the small subunit Null mutant is viable but shows respiratory deficiency and loss of wild-type mtDNA: conversion to rho- and rho zero petites
**YLR439W**	[MRPL4] Mitochondrial ribosomal protein of the large subunit Null mutant is viable, fails to grow on nonfermentable carbon sources, has growth defects on fermentable carbon sources
**YNL252C**	[MRPL17] Mitochondrial ribosomal protein of the large subunit
**YML025C**	[YML6] Mitochondrial ribosomal protein of the large subunit, has similarity to E. coli L4 ribosomal protein and human mitoribosomal MRP-L4 protein; essential for viability, unlike most other mitoribosomal proteins
**YNL005C**	[MRP7] Mitochondrial ribosomal protein of the large subunit
**YDR322W**	[MRPL35] Mitochondrial ribosomal protein of the large subunit
**YDR237W**	[MRPL7] Mitochondrial ribosomal protein of the large subunit

**YDR462W**	[MRPL28] Mitochondrial ribosomal protein of the large subunit
**YNL284C**	[MRPL10] Mitochondrial ribosomal protein of the large subunit; appears as two protein spots (YmL10 and YmL18) on two-dimensional SDS gels
**YBL038W**	[MRPL16] Mitochondrial ribosomal protein of the large subunit ribosomal protein
**YGR220C**	[MRPL9] Mitochondrial ribosomal protein of the large subunit
**YCR046C**	[IMG1] Mitochondrial ribosomal protein of the small subunit, required for respiration and for maintenance of the mitochondrial genome mitochondrial ribosomal protein Null mutant is viable; respiration deficient
YDR296W	[MHR1] Protein involved in homologous recombination in mitochondria and in transcription regulation in nucleus; binds to activation domains of acidic activators; required for recombination-dependent mtDNA partitioning Temperature sensitive in the maintenance of mitochondrial DNA

0033	GO_TERM:[organellar small ribosomal subunit] P-Value:5.5e-73 OVERLAP:[mitochondrial ribosomal small subunit] <500.60.20> SIZE:31
YGR185C	[TYS1] Cytoplasmic tyrosyl-tRNA synthetase, class I aminoacyl-tRNA synthetase that aminoacylates tRNA(Tyr), required for cytoplasmic protein synthesis, interacts with positions 34 and 35 of the anticodon of tRNATyr tyrosine-tRNA ligase
**YMR158W**	[MRPS8] Mitochondrial ribosomal protein of the small subunit
**Q0140**	[VAR1] Mitochondrial ribosomal protein of the small subunit, mitochondrially-encoded; polymorphic in different strains due to variation in number of AAT (asparagine) codons; translated near the mitochondrial inner membrane mitochondrial ribosomal protein
**YPR166C**	[MRP2] Mitochondrial ribosomal protein of the small subunit 14 kDa mitochondrial ribosomal protein|similar to E. coli S14 protein defective mitochondrial protein synthesis; absence of a and b type cytochromes; reduced levels of mitochondrial 15 S rRNA; defective processing of apocytochrome b intron; convert to rho- and rho0 at high frequency
**YDL045W-A**	[MRP10] Mitochondrial ribosomal protein of the small subunit mitochondrial ribosome 37 S subunit component Null mutant is viable, defective in mitochondrial translation and shows a tendency to accumulate deletions in mitochondrial DNA
YDR494W	[RSM28] Mitochondrial ribosomal protein of the small subunit; genetic interactions suggest a possible role in promoting translation initiation Mitochondrial ribosomal small subunit protein Null: Viable, Pet+, mild H2O2 sensitivity. Other phenotypes: Dominant suppressor allele, due to internal deletion, selected by asking for increased expression of COX2 alleles with short deletions in the leader peptide coding region.
**YNR037C**	[RSM19] Mitochondrial ribosomal protein of the small subunit, has similarity to E. coli S19 ribosomal protein mitochondrial ribosome small subunit component
**YKL003C**	[MRP17] Mitochondrial ribosomal protein of the small subunit; MRP17 exhibits genetic interactions with PET122, encoding a COX3-specific translational activator ribosomal protein MRP17 petite
**YBL090W**	[MRP21] Mitochondrial ribosomal protein of the large subunit; MRP21 exhibits genetic interactions with mutations in the COX2 and COX3 mRNA 5'-untranslated leader sequences mitochondrial ribosome small subunit component Null mutant is viable, exhibits completely blocked mitochondrial gene expression; missense mutations suppress 5'-UTL mutations in at least 2 mitochondrial mRNAs
**YMR188C**	[MRPS17] Mitochondrial ribosomal protein of the small subunit
YDR124W	
YJL063C	[MRPL8] Mitochondrial ribosomal protein of the large subunit Null mutant is viable; shows loss of mitochondrial function, instability of mitochondrial DNA
YNR036C	
**YGR215W**	[RSM27] Mitochondrial ribosomal protein of the small subunit mitochondrial ribosome small subunit component
**YNL306W**	[MRPS18] Mitochondrial ribosomal protein of the small subunit; essential for viability, unlike most other mitoribosomal proteins
**YGR084C**	[MRP13] Mitochondrial ribosomal protein of the small subunit 35 kDa mitochondrial ribosomal small subunit protein Null mutant is viable, no impairment in ribosome synthesis or function
YOL027C	[MDM38] Mitochondrial inner membrane protein, required for K+/H+ exchange and for normal mitochondrial morphology and inheritance; associates with mitochondrial ribosomes; human ortholog Letm1 is implicated in Wolf-Hirschhorn syndrome
**YJR101W**	[RSM26] Mitochondrial ribosomal protein of the small subunit mitochondrial ribosome small subunit component
YPR125W	[YLH47] Mitochondrial inner membrane protein exposed to the mitochondrial matrix, associates with mitochondrial ribosomes, NOT required for respiratory growth; homolog of human Letm1, a protein implicated in Wolf-Hirschhorn syndrome
**YBR146W**	[MRPS9] Mitochondrial ribosomal protein of the small subunit ribosomal protein S9 (putative) Null mutant is viable, respiration deficient, exhibits defects in mitochondrial protein synthesis as indicated by a loss of cytochrome c oxidase activity
**YPL118W**	[MRP51] Mitochondrial ribosomal protein of the large subunit; MRP51 exhibits genetic interactions with mutations in the COX2 and COX3 mRNA 5'-untranslated leader sequences mitochondrial ribosome small subunit component Null mutant is viable, exhibits completely blocked mitochondrial gene expression; missense mutations suppress 5'-UTL mutations in at least 2 mitochondrial mRNAs

YIL093C	[RSM25] Mitochondrial ribosomal protein of the small subunit mitochondrial ribosome small subunit component Null mutant is viable, but unable to respire.
YOR158W	[PET123] Mitochondrial ribosomal protein of the small subunit; PET123 exhibits genetic interactions with PET122, which encodes a COX3 mRNA-specific translational activator mitochondrial ribosomal protein of small subunit Null mutant is viable but is rho- (with large deletions in mtDNA); pet123 mutations can suppress pet122 mutations; some pet123 alleles show synthetic phenotypes with mrp1 mutations
YIL070C	[MAM33] Acidic protein of the mitochondrial matrix involved in oxidative phosphorylation; related to the human complement receptor gC1q-R
YDR036C	[EHD3] Protein of unconfirmed function, plays an indirect role in endocytic membrane trafficking, member of a family of enoyl-CoA hydratase/isomerases
YJR113C	[RSM7] Mitochondrial ribosomal protein of the small subunit, has similarity to E. coli S7 ribosomal protein mitochondrial ribosome small subunit component
YHL004W	[MRP4] Mitochondrial ribosomal protein of the small subunit mitochondrial ribosomal protein|mitochondrial ribosome 37 S subunit component|similar to E. coli ribosomal protein S2
YDR175C	[RSM24] Mitochondrial ribosomal protein of the small subunit mitochondrial ribosome small subunit component
YGR165W	[MRPS35] Mitochondrial ribosomal protein of the small subunit
YDR347W	[MRP1] Mitochondrial ribosomal protein of the small subunit; MRP1 exhibits genetic interactions with PET122, encoding a COX3-specific translational activator, and with PET123, encoding a small subunit mitochondrial ribosomal protein 37 kDa mitochondrial ribosomal protein defective mitochondrial protein synthesis; absence of a and b type cytochromes; reduced levels of mitochondrial 15 S rRNA; defective processing of apocytochrome b intron; convert to rho- and rho0 at high frequency
YBR251W	[MRPS5] Mitochondrial ribosomal protein of the small subunit ribosomal protein S5 (putative)
YPL013C	[MRPS16] Mitochondrial ribosomal protein of the small subunit
YDR337W	[MRPS28] Mitochondrial ribosomal protein of the small subunit ribosomal protein (E. coli S15) Null mutant is viable, unable to respire, spontaneously loses portions of its mitochondrial genomes at a high frequencY
YER050C	[RSM18] Mitochondrial ribosomal protein of the small subunit, has similarity to E. coli S18 ribosomal protein mitochondrial ribosome small subunit component null is unable to grow on glycerol
YKL155C	[RSM22] Mitochondrial ribosomal protein of the small subunit mitochondrial ribosome small subunit component Null mutant is viable, unable to respire
YDR041W	[RSM10] Mitochondrial ribosomal protein of the small subunit, has similarity to E. coli S10 ribosomal protein; essential for viability, unlike most other mitoribosomal proteins mitochondrial ribosome small subunit component
YGL129C	[RSM23] Mitochondrial ribosomal protein of the small subunit, has similarity to mammalian apoptosis mediator proteins; null mutation prevents induction of apoptosis by overproduction of metacaspase Mca1p ATPase (putative)|mitochondrial ribosome small subunit component
YNL137C	[NAM9] Mitochondrial ribosomal component of the small subunit mitochondrial S4 ribosomal protein (putative) Null mutant is viable but is respiration-deficient and loses mitochondrial DNA integrity

0034	GO_TERM:[integral to membrane] P-Value:1.2e-01
YNL194C	
YPR079W	[MRL1] Membrane protein with similarity to mammalian mannose-6-phosphate receptors, possibly functions as a sorting receptor in the delivery of vacuolar hydrolases
YDR284C	[DPP1] Diacylglycerol pyrophosphate (DGPP) phosphatase, zinc-regulated vacuolar membrane-associated lipid phosphatase, dephosphorylates DGPP to phosphatidate (PA) and Pi, then PA to diacylglycerol; involved in lipid signaling and cell metabolism diacylglycerol pyrophosphate phosphatase
YKR088C	[TVP38] Integral membrane protein localized to late Golgi vesicles along with the v-SNARE Tlg2p; green fluorescent protein (GFP)-fusion protein localizes to the cytoplasm in a punctate pattern integral membrane protein Null: no notable phenotype

0035	GO_TERM:[late endosome] P-Value:3.4e-12
YKR039W	[GAP1] General amino acid permease; localization to the plasma membrane is regulated by nitrogen source general amino acid permease abolished activity of the general amino acid transport system
YKR007W	[MEH1] Component of the EGO complex, which is involved in the regulation of microautophagy; localizes to the vacuolar membrane, loss results in a defect in vacuolar acidification
YML121W	[GTR1] Cytoplasmic GTP binding protein and negative regulator of the Ran/Tc4 GTPase cycle, through its homolog and binding partner, Gtr2p; involved in phosphate transport and invasive growth; human RagA and RagB proteins are functional homologs small GTPase (putative) Null mutant is viable but grows slowly, is cold-sensitive, and has defects in phosphate uptake
YBR077C	[SLM4] Component of the EGO complex, which is involved in the regulation of microautophagy; gene exhibits synthetic genetic interaction with MSS4 encoding phosphatidylinositol 4-phosphate kinase

YGR163W	[GTR2] Cytoplasmic GTP binding protein, negative regulator of the Ran/Tc4 GTPase cycle downstream of Gtr1p; homolog of human RagC and RagD proteins; component of the EGO complex, which is involved in the regulation of microautophagy similar to Gtr1|small GTPase (putative)

0036	GO_TERM:[integral to peroxisomal membrane] P-Value:6.1e-08
YBR168W	[PEX32] Peroxisomal integral membrane protein, involved in negative regulation of peroxisome size; partially functionally redundant with Pex31p; genetic interactions suggest action at a step downstream of steps mediated by Pex28p and Pex29p
YDL089W	
YGR004W	[PEX31] Peroxisomal integral membrane protein, involved in negative regulation of peroxisome size; partially functionally redundant with Pex30p and Pex32p; probably acts at a step downstream of steps mediated by Pex28p and Pex29p
YLR324W	[PEX30] Peroxisomal integral membrane protein, involved in negative regulation of peroxisome number; partially functionally redundant with Pex31p; genetic interactions suggest action at a step downstream of steps mediated by Pex28p and Pex29p

0037	GO_TERM:[lipid raft] P-Value:5.7e-06
YBR021W	[FUR4] Uracil permease, localized to the plasma membrane; expression is tightly regulated by uracil levels and environmental cues uracil permease
YML052W	[SUR7] Integral membrane protein localized to eisosomes, large immobile protein structures at the cell cortex associated with endocytosis; sporulation and plasma membrane sphingolipid content are altered in mutants; has homologs YNL194C and FMP45 integral membrane protein Null mutant is viable, exhibits no growth defects on non-fermentable carbon sources or sensitivites to 3-AT or high salt

0038	GO_TERM:[GPI-anchor transamidase activity] P-Value:3.5e-06
YBR296C	[PHO89] Na+/Pi cotransporter, active in early growth phase; similar to phosphate transporters of Neurospora crassa; transcription regulated by inorganic phosphate concentrations and Pho4p Na+/Pi symporter (putative)
YHR188C	[GPI16] Transmembrane protein subunit of the glycosylphosphatidylinositol transamidase complex that adds GPIs to newly synthesized proteins; human PIG-Tp homolog GPI transamidase component, human PIG-T homologue
YLR088W	[GAA1] Subunit of the GPI (glycosylphosphatidylinositol):protein transamidase complex, removes the GPI-anchoring signal and attaches GPI to proteins in the ER GPI:protein transamidase component (putative)

0039	GO_TERM:[membrane] P-Value:6.6e-02
YDL210W	[UGA4] Permease that serves as a gamma-aminobutyrate (GABA) transport protein involved in the utilization of GABA as a nitrogen source; catalyzes the transport of putrescine and delta-aminolevulinic acid (ALA); localized to the vacuolar membrane GABA-specific transport protein
YNL279W	[PRM1] Pheromone-regulated multispanning membrane protein involved in membrane fusion during mating; predicted to have 5 transmembrane segments and a coiled coil domain; localizes to the shmoo tip; regulated by Ste12p Null mutant is viable but exhibits a mating defect.

0040	GO_TERM:[Sec62/Sec63 complex] P-Value:1.6e-08 OVERLAP:[Sec62-63 complex] <520.10.20> SIZE:4
YLR056W	[ERG3] C-5 sterol desaturase, catalyzes the introduction of a C-5(6) double bond into episterol, a precursor in ergosterol biosynthesis; mutants are viable, but cannot grow on non-fermentable carbon sources C-5 sterol desaturase Null mutant is inviable; suppresses syringomycin resistant mutant; sensitive to photoactivated 3-carbethoxypsoralen, UV light, radiomimetic mutagens, and oxidative stress
**YLR292C**	[SEC72] Non-essential subunit of Sec63 complex (Sec63p, Sec62p, Sec66p and Sec72p); with Sec61 complex, Kar2p/BiP and Lhs1p forms a channel competent for SRP-dependent and post-translational SRP-independent protein targeting and import into the ER
YIL162W	[SUC2] Invertase, sucrose hydrolyzing enzyme; a secreted, glycosylated form is regulated by glucose repression, and an intracellular, nonglycosylated enzyme is produced constitutively invertase (sucrose hydrolyzing enzyme) Null mutant is viable but cannot ferment sucrose
**YBR171W**	[SEC66] Non-essential subunit of Sec63 complex (Sec63p, Sec62p, Sec66p and Sec72p); with Sec61 complex, Kar2p/BiP and Lhs1p forms a channel competent for SRP-dependent and post-translational SRP-independent protein targeting and import into the ER glycoprotein complexed with Sec62p and Sec63p in the Sec63 complex, an integral endoplasmic reticulum membrane protein complex required for translocation of presecretory proteins

**YPL094C**	[SEC62] Essential subunit of Sec63 complex (Sec63p, Sec62p, Sec66p and Sec72p); with Sec61 complex, Kar2p/BiP and Lhs1p forms a channel competent for SRP-dependent and post-translational SRP-independent protein targeting and import into the ER ER protein translocation apparatus membrane component secretion deficient
0041	GO_TERM:[transporter activity] P-Value:3.2e-03
YBR293W	[VBA2] Permease of basic amino acids in the vacuolar membrane
YLR083C	[EMP70] Protein whose 24kDa cleavage product is found in endosome-enriched membrane fractions, predicted to be a transmembrane protein
0042	GO_TERM:[intracellular transport] P-Value:1.5e-01 OVERLAP:[Signal peptidase] <520.30> SIZE:3
**YIR022W**	[SEC11] 18kDa catalytic subunit of the Signal Peptidase Complex (SPC; Spc1p, Spc2p, Spc3p, and Sec11p) which cleaves the signal sequence of proteins targeted to the endoplasmic reticulum
YDR456W	[NHX1] Endosomal Na+/H+ exchanger, required for intracellular sequestration of Na+; required for osmotolerance to acute hypertonic shock Na+/H+ exchanger
YIL171W	
0043	GO_TERM:[Golgi apparatus] P-Value:6.4e-03
YAR033W	[MST28] Putative integral membrane protein, involved in vesicle formation; forms complex with Mst27p; member of DUP240 gene family; binds COPI and COPII vesicles
YKL174C	[TPO5] Protein involved in excretion of putrescine and spermidine; putative polyamine transporter in the Golgi or post-Golgi vesicles
0044	GO_TERM:[amino acid transporter activity] P-Value:9.0e-05
YBR068C	[BAP2] High-affinity leucine permease, functions as a branched-chain amino acid permease involved in the uptake of leucine, isoleucine and valine; contains 12 predicted transmembrane domains reduced uptake of leucine, isoleucine, and valine
YDR046C	[BAP3] Amino acid permease involved in the uptake of cysteine, leucine, isoleucine and valine valine transporter
0045	
YKL004W	[AUR1] Phosphatidylinositol:ceramide phosphoinositol transferase (IPC synthase), required for sphingolipid synthesis; can mutate to confer aureobasidin A resistance Null mutant is inviable; mutant exhibits dominant resistance to aureobasidin A. Wild type (sensitive) is recessive.
YCR010C	[ADY2] Acetate transporter required for normal sporulation transmembrane protein Null mutant is viable; forms predominantly asci containing 2 spores (dyads) whensporulated; required for long-term growth on YPD at 37 degrees C; defect in ammonia production in S.cerevisiae colonies
YCR061W	
0046	GO_TERM:[ER to Golgi vesicle-mediated transport] P-Value:1.1e-02
YLL056C	
YNR026C	[SEC12] Guanine nucleotide exchange factor (GEF); glycosylated integral membrane protein of the endoplasmic reticulum, important for the initiation of transport vesicle budding from the endoplasmic reticulum through activation of the GTPase Sar1p guanine nucleotide exchange factor for Sar1p Null mutant is inviable. Defective in endoplasmic reticulum to Golgi transport.
YLL023C	
YMR292W	[GOT1] Evolutionarily conserved non-essential protein present in early Golgi cisternae that may be involved in ER-Golgi transport at a step after vesicle tethering to Golgi membranes, exhibits membrane topology similar to that of Sft2p membrane protein Null mutant is viable but exhibits ER to Golgi transport defects in vitro. got1 is synthetically lethal with mutations in sft2; the got1 sft2 double mutant exhibits defects in transport to the Golgi complex.
0047	
YHR050W	[SMF2] Divalent metal ion transporter involved in manganese homeostasis; has broad specificity for di-valent and tri-valent metals; post-translationally regulated by levels of metal ions; member of the Nramp family of metal transport proteins Null mutant is viable, shows double mutant sickness with smf1 null
YJR015W	

0048	GO_TERM:[oligosaccharyl transferase activity] P-Value:2.1e-25 OVERLAP:[Oligosaccharyltransferase] <520.20> SIZE:9
YML055W	[SPC2] Subunit of signal peptidase complex (Spc1p, Spc2p, Spc3p, Sec11p), which catalyzes cleavage of N-terminal signal sequences of proteins targeted to the secretory pathway; homologous to mammalian SPC25 signal peptidase complex subunit|similar to mammalian protein SPC25 Null mutant is viable. spc1 spc2 double deletion mutants grow relatively well as compared to wild-type. spc2 sec11 double deletion mutant is inviable. Spc2p is important for cell viability and signal peptidase activity at high temperatures (42 degrees celsius).
YER019C-A	[SBH2] Ssh1p-Sss1p-Sbh2p complex component, involved in protein translocation into the endoplasmic reticulum; homologous to Sbh1p Sbh1p homolog Null mutant is viable. sbh1 sbh2 double deletion mutants exhibit synthetic temperature sensitivity and accumulation of secretory protein precursors
**YGL226C-A**	[OST5] Zeta subunit of the oligosaccharyltransferase complex of the ER lumen, which catalyzes asparagine-linked glycosylation of newly synthesized proteins oligosaccharyltransferase complex 9.5 kDa zeta subunit
YDR086C	[SSS1] Subunit of the Sec61p translocation complex (Sec61p-Sss1p-Sbh1p) that forms a channel for passage of secretory proteins through the endoplasmic reticulum membrane, and of the Ssh1p complex (Ssh1p-Sbh2p-Sss1p); interacts with Ost4p and Wbp1p ER protein|Sec61 trimeric complex component|Ssh1 trimeric complex component Null mutant is inviable. Depletion of the Sss1 protein rapidly results in accumulation of multiple secretory or membrane proteins devoid of post-translational modifications. SSS1 overexpression restores translocation in sec61 mutants.
**YMR149W**	[SWP1] Delta subunit of the oligosaccharyl transferase glycoprotein complex, which is required for N-linked glycosylation of proteins in the endoplasmic reticulum oligosaccharyl transferase glycoprotein complex, delta subunit lethal
**YEL002C**	[WBP1] Beta subunit of the oligosaccharyl transferase (OST) glycoprotein complex; required for N-linked glycosylation of proteins in the endoplasmic reticulum oligosaccharyl transferase glycoprotein complex, beta subunit lethal
YLR378C	[SEC61] Essential subunit of Sec61 complex (Sec61p, Sbh1p, and Sss1p); forms a channel for SRP-dependent protein import and retrograde transport of misfolded proteins out of the ER; with Sec63 complex allows SRP-independent protein import into ER
YER087C-B	[SBH1] Beta subunit of the Sec61p ER translocation complex (Sec61p-Sss1p-Sbh1p); involved in protein translocation into the endoplasmic reticulum; interacts with the exocyst complex; homologous to Sbh2p Sbh2p homolog Null mutant is viable. sbh1 sbh2 double deletion mutants exhibit synthetic temperature sensitivity and accumulation of secretory protein precursors
**YDL232W**	[OST4] Subunit of the oligosaccharyltransferase complex of the ER lumen, which catalyzes protein asparagine-linked glycosylation; type I membrane protein required for incorporation of Ost3p or Ost6p into the OST complex 3.6 kDa protein Null mutant is viable but is cold- and heat-sensitive; vanadate-resistant, hygromycin B-sensitive; defective in oligosaccharyltransferase activity in vivo and in vitro
**YJL002C**	[OST1] Alpha subunit of the oligosaccharyltransferase complex of the ER lumen, which catalyzes asparagine-linked glycosylation of newly synthesized proteins 64 kDa, alpha subunit of oligosaccharyltransferase complex; homologous to mammalian ribophorin I Null mutant is inviable; temperature-sensitive mutants show pleiotropic underglycosylation of soluble and membrane-bound glycoproteins
**YOR103C**	[OST2] Epsilon subunit of the oligosaccharyltransferase complex of the ER lumen, which catalyzes asparagine-linked glycosylation of newly synthesized proteins 40% identical to vertebrate DAD1 protein|oligosaccharyltransferase complex 16 kDa epsilon subunit Null mutant is inviable; overexpression of OST2 suppresses temperature-sensitivity of wbp1-2 mutant; conditional mutants show pleiotropic underglycosylation of soluble and membrane-bound glycoproteins
**YOR085W**	[OST3] Gamma subunit of the oligosaccharyltransferase complex of the ER lumen, which catalyzes asparagine-linked glycosylation of newly synthesized proteins; Ost3p is important for N-glycosylation of a subset of proteins oligosaccharyl transferase glycoprotein complex 34 kDa gamma subunit Null mutant is viable but shows underglycosylation of soluble and membrane-bound glycoproteins and contains less oligosaccharyltransferase activity in vitro
**YGL022W**	[STT3] Subunit of the oligosaccharyltransferase complex of the ER lumen, which catalyzes asparagine-linked glycosylation of newly synthesized proteins; forms a subcomplex with Ost3p and Ost4p and is directly involved in catalysis integral ER membrane protein|oligosaccharyltransferase complex subunit (putative) Null mutant is inviable. sst3 mutants are defective in protein glycosylation, sensitive to hygromycin B, and resistant to sodium orthovanadate. Depletion of the STT3 protein results in loss of oligosaccharyl transferase activity in vivo and a deficiency in the assembly of oligosaccharyl transferase complex.
**YML019W**	[OST6] Subunit of the oligosaccharyltransferase complex of the ER lumen, which catalyzes asparagine-linked glycosylation of newly synthesized proteins; similar to and partially functionally redundant with Ost3p N-oligosaccharyltransferase complex 37kDa subunit (putative)

0049	GO_TERM:[endoplasmic reticulum] P-Value:2.5e-02
YGL053W	[PRM8] Pheromone-regulated protein with 2 predicted transmembrane segments and an FF sequence, a motif involved in COPII binding; forms a complex with Prp9p in the ER; member of DUP240 gene family
YOR307C	[SLY41] Protein involved in ER-to-Golgi transport chloroplast phosphate transporter homolog

0050	GO_TERM:[sphingosine N-acyltransferase activity] P-Value:8.1e-07
YHL003C	[LAG1] Ceramide synthase component, involved in synthesis of ceramide from C26(acyl)-coenzyme A and dihydrosphingosine or phytosphingosine, functionally equivalent to Lac1p
YMR298W	[LIP1] Ceramide synthase subunit; single-span ER membrane protein associated with Lag1p and Lac1p and required for ceramide synthase activity, null mutant grows extremely slowly and is defective in ceramide synthesis

0051	GO_TERM:[Golgi apparatus part] P-Value:1.4e-02
YLR241W	
YER005W	[YND1] Apyrase with wide substrate specificity, involved in preventing the inhibition of glycosylation by hydrolyzing nucleoside tri- and diphosphates which are inhibitors of glycotransferases; partially redundant with Gda1p apyrase (NDPase/NTPase) Null mutant is viable but vanadate-resistant and hygromycin-sensitive. The double mutant ynd1 gda1 exhibits slow growth and substantial defects in protein glycosylation and cell morphology.
YJL004C	[SYS1] Integral membrane protein of the Golgi required for targeting of the Arf-like GTPase Arl3p to the Golgi; multicopy suppressor of ypt6 null mutation Null mutant is viable. sys1 ypt6 double mutant displays enhanced defects in vacuolar sorting and cell growth

0052	
YBR054W	[YRO2] Putative plasma membrane protein of unknown function, transcriptionally regulated by Haa1p; green fluorescent protein (GFP)-fusion protein localizes to the cell periphery and bud
YML132W	[COS3] Protein involved in salt resistance; interacts with sodium:hydrogen antiporter Nha1p; member of the DUP380 subfamily of conserved, often subtelomerically-encoded proteins

0053	
YAL018C	
YLR034C	[SMF3] Putative divalent metal ion transporter involved in iron homeostasis; transcriptionally regulated by metal ions; member of the Nramp family of metal transport proteins Nramp homolog|SMF1 and SMF2 homolog|metal transporter (putative)

0054	
YAR028W	
YPR128C	[ANT1] Peroxisomal adenine nucleotide transporter; involved in beta-oxidation of medium-chain fatty acid; required for peroxisome proliferation Null: growth defect on medium-chain length fatty acids.

0055	GO_TERM:[membrane part] P-Value:1.1e-01 OVERLAP:[H+-transporting ATPase, vacuolar] <220> SIZE:15
YGR105W	[VMA21] Integral membrane protein that is required for vacuolar H+-ATPase (V-ATPase) function, although not an actual component of the V-ATPase complex; functions in the assembly of the V-ATPase; localized to the yeast endoplasmic reticulum (ER) Null mutant is viable but grows slowly and exhibits increased calcium sensitivity. Null mutants also cannot grow on glycerol or at pH 7.5
**YPL234C**	[TFP3] Vacuolar ATPase V0 domain subunit c', involved in proton transport activity; hydrophobic integral membrane protein (proteolipid) containing four transmembrane segments; N and C termini are in the vacuolar lumen vacuolar ATPase V0 domain subunit c' (17 kDa)|vacuolar H(+) ATPase 17 kDa subunit C Null mutant is viable, defective in vacuolar acidification, high copy TFP3 confers resistance to trifluoperazine

0056	

YBR298C	[MAL31] Maltose permease, high-affinity maltose transporter (alpha-glucoside transporter); encoded in the MAL3 complex locus; member of the 12 transmembrane domain superfamily of sugar transporters; functional in genomic reference strain S288C maltose permease Defective maltose fermentation
YMR279C	
0057	
YFL062W	[COS4] Protein of unknown function, member of the DUP380 subfamily of conserved, often subtelomerically-encoded proteins
YGR191W	[HIP1] High-affinity histidine permease, also involved in the transport of manganese ions histidine permease requires supplementation with large amounts of histidine for growth
0058	GO_TERM:[membrane] P-Value:1.0e-30 OVERLAP:[H+-ATPase, plasma mebrane] <210> SIZE:4
YHR123W	[EPT1] sn-1,2-diacylglycerol ethanolamine- and cholinephosphotranferase; not essential for viability sn-1,2-diacylglycerol ethanolamine- and cholinephosphotranferase
YNL130C	[CPT1] Cholinephosphotransferase, required for phosphatidylcholine biosynthesis and for inositol-dependent regulation of EPT1 transcription sn-1,2-diacylglycerol cholinephosphotransferase Null mutant is viable, cpt1 ept1 double deletion mutants are viable
YIL114C	[POR2] Putative mitochondrial porin (voltage-dependent anion channel), related to Por1p but not required for mitochondrial membrane permeability or mitochondrial osmotic stability voltage dependent anion channel (YVDAC2) Null mutant is viable; omp2 por2 double null mutant shows poor growth; POR2 is a multicopy suppressor of omp2 null temperature-sensitive petite phenotype
YJL091C	[GWT1] Protein involved in the inositol acylation of glucosaminyl phosphatidylinositol (GlcN-PI) to form glucosaminyl(acyl)phosphatidylinositol (GlcN(acyl)PI), an intermediate in the biosynthesis of glycosylphosphatidylinositol (GPI) anchors overexpression confers 1-[4-butylbenzyl]isoquinoline (BIQ)-resistant growth in S. cerevisiae.
YLR004C	
YGR289C	[MAL11] Maltose permease, inducible high-affinity maltose transporter (alpha-glucoside transporter); encoded in the MAL1 complex locus; member of the 12 transmembrane domain superfamily of sugar transporters alpha-glucoside transporter|hexose transporter|maltose permease Mutant is defective in maltose fermentation.
YCL052C	[PBN1] Essential component of glycosylphosphatidylinositol-mannosyltransferase I, required for the autocatalytic post-translational processing of the protease B precursor Prb1p, localizes to ER in lumenal orientation; homolog of mammalian PIG-X protease B nonderepressible form Null mutant is inviable; overexpression of both PBN1 and LRE1 confers resistance to laminarinase, which degrades cell wall beta(1-3) glucan linkages; overexpression of either gene alone has no effect on cell wall glucans or glucan synthase activity
YAR027W	[UIP3] Putative integral membrane protein of unknown function; interacts with Ulp1p at the nuclear periphery; member of DUP240 gene family
YML123C	[PHO84] High-affinity inorganic phosphate (Pi) transporter and low-affinity manganese transporter; regulated by Pho4p and Spt7p; mutation confers resistance to arsenate; exit from the ER during maturation requires Pho86p inorganic phosphate transporter
YMR264W	[CUE1] Endoplasmic reticulum membrane protein that recruits the ubiquitin-conjugating enzyme Ubc7p to the ER where it functions in protein degradation; contains a CUE domain that binds ubiquitin to facilitate intramolecular monoubiquitination Ubc7p binding and recruitment protein Null mutant is viable and shows stabilization of ER degradation substrates
YDL054C	[MCH1] Protein with similarity to mammalian monocarboxylate permeases, which are involved in transport of monocarboxylic acids across the plasma membrane; mutant is not deficient in monocarboxylate transport
YJL214W	[HXT8] Protein of unknown function with similarity to hexose transporter family members, expression is induced by low levels of glucose and repressed by high levels of glucose hexose permease
YEL027W	[CUP5] Proteolipid subunit of the vacuolar H(+)-ATPase V0 sector (subunit c; dicyclohexylcarbodiimide binding subunit); required for vacuolar acidification and important for copper and iron metal ion homeostasis 17 kDa|VO sector subunit|dicyclohexylcarbodiimide binding subunit|proteolipid|vacuolar ATP synthase proteolipid C|vacuolar ATPase V0 domain subunit c (dicyclohexylcarbodiimide binding subunit) (17 kDa) Null mutant is viable, petite, copper sensitive
YDR414C	[ERD1] Predicted membrane protein required for the retention of lumenal endoplasmic reticulum proteins; mutants secrete the endogenous ER protein, BiP (Kar2p) disruption of the retention system for ER proteins; defects in the Golgi-dependent modification of glycoproteins|exhibits defects in N-glycosylation of proteins
**YCR024C-A**	[PMP1] Small single-membrane span proteolipid that functions as a regulatory subunit of the plasma membrane H(+)-ATPase Pma1p, forms unique helix and positively charged cytoplasmic domain that is able to specifically segregate phosphatidylserines proteolipid associated with plasma membrane H(+)-ATPase (Pma1p) Null mutant is viable; pmp1 pmp2 double mutant displays a lower Vmax for the plasma membrane H(+)-ATPase (Pma1p)
YHR007C	[ERG11] Lanosterol 14-alpha-demethylase, catalyzes the C-14 demethylation of lanosterol to form 4,4''-dimethyl cholesta-8,14,24-triene-3-beta-ol in the ergosterol biosynthesis pathway; member of the cytochrome P450 family cytochrome P450 lanosterol 14a-demethylase Null mutant is inviable, erg11 null inviability is suppressed by deletion of ERG3; erg11 mutants are ergosterol biosynthesis defective; many are also nystatin resistant
YDR276C	[PMP3] Small plasma membrane protein related to a family of plant polypeptides that are overexpressed under high salt concentration or low temperature, not essential for viability, deletion causes hyperpolarization of the plasma membrane potential hypothetical transmembrane protein Null mutant is viable and sensitive to cations such as sodium

YGL104C	[VPS73] Mitochondrial protein of unknown function involved in vacuolar protein sorting Null mutant secretes CPY.
YOL003C	[PFA4] Palmitoyltransferase with autoacylation activity; member of a family of putative palmitoyltransferases containing an Asp-His-His-Cys-cysteine rich (DHHC-CRD) domain palmitoyltransferase
YNL048W	[ALG11] Alpha-1,2-mannosyltransferase, catalyzes addition of the terminal alpha 1,2-Man to the Man5GlcNAc2-PP-dolichol intermediate during asparagine-linked glycosylation in the ER Null mutant displays poor growth and temperature-sensitive lethality
YPR198W	[SGE1] Membrane-associated multidrug transporter, acts as an extrusion permease, member of the drug-resistance protein family within the major facilitator superfamily (MFS), partial multicopy suppressor of gal11 mutations Null mutant is viable; shows decreased expression of galactose-inducible genes; shows increased sensitivity to crystal violet
YFL041W	[FET5] Multicopper oxidase, integral membrane protein with similarity to Fet3p; may have a role in iron transport multicopper oxidase|type 1 integral membrane protein overexpression of FET5 suppresses a fet3 null mutant.
YLR343W	[GAS2] Putative 1,3-beta-glucanosyltransferase, has similarity to Gas1p
YML075C	[HMG1] One of two isozymes of HMG-CoA reductase that catalyzes the conversion of HMG-CoA to mevalonate, which is a rate-limiting step in sterol biosynthesis; localizes to the nuclear envelope; overproduction induces the formation of karmellae 3-hydroxy-3-methylglutaryl-coenzyme A (HMG-CoA) reductase isozyme Null mutant is viable, sensitive to compactin, a competitive inhibitor of HMG-CoA reductase; hmg1 hmg2 double deletion mutants are inviable
YOR254C	[SEC63] Essential subunit of Sec63 complex (Sec63p, Sec62p, Sec66p and Sec72p); with Sec61 complex, Kar2p/BiP and Lhs1p forms a channel competent for SRP-dependent and post-translational SRP-independent protein targeting and import into the ER lethal
YOL132W	[GAS4] Putative 1,3-beta-glucanosyltransferase, has similarity to Gas1p; localizes to the cell wall
YHR110W	[ERP5] Protein with similarity to Emp24p and Erv25p, member of the p24 family involved in ER to Golgi transport p24 protein involved in membrane trafficking
YAL007C	[ERP2] Protein that forms a heterotrimeric complex with Erp1p, Emp24p, and Erv25p; member, along with Emp24p and Erv25p, of the p24 family involved in ER to Golgi transport and localized to COPII-coated vesicles p24 protein involved in membrane trafficking null mutant is viable; delayed transport of Gas1p
YPR192W	[AQY1] Spore-specific water channel that mediates the transport of water across cell membranes, developmentally controlled; may play a role in spore maturation, probably by allowing water outflow, may be involved in freeze tolerance aquaporin Null mutant is viable and exhibits improved viability when grown under hypo-osmolar or hyper-osmolar stress.
YPL274W	[SAM3] High-affinity S-adenosylmethionine permease, required for utilization of S-adenosylmethionine as a sulfur source; has similarity to S-methylmethionine permease Mmp1p high affinity S-adenosylmethionine permease Null mutant is viable but has inability to use S-adenosylmethionine as a sulfur source
YBR283C	[SSH1] Subunit of the Ssh1 translocon complex; Sec61p homolog involved in co-translational pathway of protein translocation; not essential Null mutant is viable, but grows slowly
YBR069C	[TAT1] Amino acid transport protein for valine, leucine, isoleucine, and tyrosine, low-affinity tryptophan and histidine transporter; overexpression confers FK506 resistance amino acid transport protein for valine, leucine, isoleucine, and tyrosine overexpression confers resistance to the volatile anesthetic isoflurane.
YGL051W	[MST27] Putative integral membrane protein, involved in vesicle formation; forms complex with Mst28p; member of DUP240 gene family; binds COPI and COPII vesicles protein with COPI and COPII bindng motifs
**YEL017C-A**	[PMP2] Proteolipid associated with plasma membrane H(+)-ATPase (Pma1p); regulates plasma membrane H(+)-ATPase activity; nearly identical to PMP1 proteolipid associated with plasma membrane H(+)-ATPase (Pma1p)
YER026C	[CHO1] Phosphatidylserine synthase, functions in phospholipid biosynthesis; catalyzes the reaction CDP-diaclyglycerol + L-serine = CMP + L-1-phosphatidylserine, transcriptionally repressed by myo-inositol and choline phosphatidylserine synthase The null mutant is viable but grows slowly on minimal medium. The growth rate of the null mutant on minimal medium can be increased by supplementing the medium with choline or other phospholipid precursors.
YPL227C	[ALG5] UDP-glucose:dolichyl-phosphate glucosyltransferase, involved in asparagine-linked glycosylation in the endoplasmic reticulum UDP-glucose:dolichyl-phosphate glucosyltransferase underglycosylation of carboxypeptidase Y
YDR307W	
YHL042W	
YCL025C	[AGP1] Low-affinity amino acid permease with broad substrate range, involved in uptake of asparagine, glutamine, and other amino acids; expression is regulated by the SPS plasma membrane amino acid sensor system (Ssy1p-Ptr3p-Ssy5p) amino acid permease Null mutant is viable; resistant to the amino acid analog gamma-hydroxyaspartate, decreased growth on asn, gln and some other amino acids in strains in which Gap1 and Gnp1 are also missing.
YGR260W	[TNA1] High affinity nicotinic acid plasma membrane permease, responsible for uptake of low levels of nicotinic acid; expression of the gene increases in the absence of extracellular nicotinic acid or para-aminobenzoate (PABA) high affinity nicotinic acid plasma membrane permease Null mutant is viable; the deletion of both YGR260W and YJR025C/BNA1 is lethal at low external nicotinic acid concentration
YPR028W	[YOP1] Membrane protein that interacts with Yip1p to mediate membrane traffic; overexpression results in cell death and accumulation of internal cell membranes; regulates vesicular traffic in stressed cells

YMR058W	[FET3] Ferro-O2-oxidoreductase required for high-affinity iron uptake and involved in mediating resistance to copper ion toxicity, belongs to class of integral membrane multicopper oxidases multicopper oxidase The null mutant is viable but defective for high affinity Fe(II) uptake. The null mutant is inviable when environmental iron is limiting.
YHR142W	[CHS7] Protein of unknown function, involved in chitin biosynthesis by regulating Chs3p export from the ER
YDL212W	[SHR3] Endoplasmic reticulum packaging chaperone, required for incorporation of amino acid permeases into COPII coated vesicles for transport to the cell surface Null mutants are viable, specifically accumulate amino acid permeases in the endoplasmic reticulum
YPR156C	[TPO3] Polyamine transport protein specific for spermine; localizes to the plasma membrane; member of the major facilitator superfamily
YHR026W	[PPA1] Subunit c" of the vacuolar ATPase, which functions in acidification of the vacuole; one of three proteolipid subunits of the V0 domain proteolipid|vacuolar ATPase V0 domain subunit c" Null mutant is inviable in some genetic backgrounds, in others, exhibits no V-ATPase activity and failure to assemble V-ATPase subunits onto the vacuolar membrane
YOR016C	[ERP4] Protein with similarity to Emp24p and Erv25p, member of the p24 family involved in ER to Golgi transport p24 protein involved in membrane trafficking
YGR284C	[ERV29] Protein localized to COPII-coated vesicles, involved in vesicle formation and incorporation of specific secretory cargo ER-Golgi transport vesicle protein
YLL028W	[TPO1] Polyamine transporter that recognizes spermine, putrescine, and spermidine; catalyzes uptake of polyamines at alkaline pH and excretion at acidic pH; phosphorylation enhances activity and sorting to the plasma membrane
YLR018C	[POM34] Integral membrane protein of the nuclear pore complex, localizes adjacent to the nuclear membrane integral membrane protein|nuclear pore complex subunit
YJR117W	[STE24] Highly conserved zinc metalloprotease that functions in two steps of a-factor maturation, C-terminal CAAX proteolysis and the first step of N-terminal proteolytic processing; contains multiple transmembrane spans zinc metallo-protease Null mutant is viable, exhibits a mating efficiency of ~5% that of a wild-type strain and an a-factor processing defect
YNL101W	[AVT4] Vacuolar transporter, exports large neutral amino acids from the vacuole; member of a family of seven S. cerevisiae genes (AVT1-7) related to vesicular GABA-glycine transporters Gln (Asn), Ile (Leu), Tyr transporter
YKL154W	[SRP102] Signal recognition particle (SRP) receptor beta subunit; involved in SRP-dependent protein targeting; anchors Srp101p to the ER membrane Null mutant is viable but exhibits slow growth and cannot grow on nonfermentable carbon sources. Temperature-sensitive alleles exhibit defects in translocation of some ER proteins at the nonpermissive temperature.
YEL063C	[CAN1] Plasma membrane arginine permease, requires phosphatidyl ethanolamine (PE) for localization, exclusively associated with lipid rafts; mutation confers canavanine resistance arginine permease Canavanine resistance
YGL200C	[EMP24] Integral membrane component of endoplasmic reticulum-derived COPII-coated vesicles, which function in ER to Golgi transport type I transmembrane protein
YHL048W	[COS8] Nuclear membrane protein, member of the DUP380 subfamily of conserved, often subtelomerically-encoded proteins; regulation suggests a potential role in the unfolded protein response
YIL016W	[SNL1] Protein of unknown function proposed to be involved in nuclear pore complex biogenesis and maintenance as well as protein folding; has similarity to the mammalian BAG-1 protein 18.3 kDa integral membrane protein Null mutant is viable; SNL1 is a high copy suppressor of nup116, gle2 and nic96 alleles
YBR036C	[CSG2] Endoplasmic reticulum membrane protein, required for mannosylation of inositolphosphorylceramide and for growth at high calcium concentrations Null mutant is viable but Ca2+-sensitive; a presumed point mutant is sensitive to Ca2+ levels greater than 10 mM (but remains insensitive to 50 mM Sr2+)
YJR010C-A	[SPC1] Subunit of the signal peptidase complex (SPC), which cleaves the signal sequence from proteins targeted to the endoplasmic reticulum (ER), homolog of the SPC12 subunit of mammalian signal peptidase complex Null mutant is viable; synthetically lethal with a conditional mutation in sec11; high copy Spc1 suppresses the conditional sec11 mutation
YKL008C	[LAC1] Ceramide synthase component, involved in synthesis of ceramide from C26(acyl)-coenzyme A and dihydrosphingosine or phytosphingosine, functionally equivalent to Lag1p LAG1 longevity gene homolog Null mutant is viable but exhibits synthetic lethality with mutations in lag1.
YPL264C	
YBR183W	[YPC1] Alkaline ceramidase that also has reverse (CoA-independent) ceramide synthase activity, catalyzes both breakdown and synthesis of phytoceramide; overexpression confers fumonisin B1 resistance alkaline ceramidase with reverse activity Null mutant is viable and two times more heat resistant than the wild-type parental strain.
YML048W	[GSF2] ER localized integral membrane protein that may promote secretion of certain hexose transporters, including Gal2p; involved in glucose-dependent repression A Tn3 insertion into this gene causes hypersensitivity to the cell surface polymer perturbing agent calcofluor white; Defective in glucose repression; mutants decrease transcriptional repression by MIG1; alter glucose-regulated subunit interactions within the Snf1 protein kinase complex; the effects of eff1 and eff2 on SUC2 repression are strongly synergistic.
YBR159W	[YBR159W] Microsomal beta-keto-reductase; contains oleate response element (ORE) sequence in the promoter region; mutants exhibit reduced VLCFA synthesis, accumulate high levels of dihydrosphingosine, phytosphingosine and medium-chain ceramides.
YBR106W	[PHO88] Probable membrane protein, involved in phosphate transport; pho88 pho86 double null mutant exhibits enhanced synthesis of repressible acid phosphatase at high inorganic phosphate concentrations Null mutant is viable; pho88 pho86 double mutant shows enhanced synthesis of repressible acid phosphatase in high phosphate media and arsenate resistance; disruption or high dosage of PHO88 results in reduced phosphate intake

YLR372W	[SUR4] Elongase, involved in fatty acid and sphingolipid biosynthesis; synthesizes very long chain 20-26-carbon fatty acids from C18-CoA primers; involved in regulation of sphingolipid biosynthesis elongase Null mutants is viable, not sensitive to UV or gamma radiation. sur4 mutants suppress rad3, rvs161 delta, and rvs167 mutations. sur4 fen1 mutants and sur4 elo2 mutants are inviable.
YHR133C	[NSG1] Protein involved in regulation of sterol biosynthesis; specifically stabilizes Hmg2p, one of two HMG-CoA isoenzymes that catalyze the rate-limiting step in sterol biosynthesis; homolog of mammalian INSIG proteins Null: none
YBR290W	[BSD2] Heavy metal ion homeostasis protein, facilitates trafficking of Smf1p and Smf2p metal transporters to the vacuole where they are degraded, controls metal ion transport, prevents metal hyperaccumulation, functions in copper detoxification
YDR297W	[SUR2] Sphinganine C4-hydroxylase, catalyses the conversion of sphinganine to phytosphingosine in sphingolipid biosyntheis sphingosine hydroxylase Null mutant has altered phospholipid levels; suppressor of rvs161 and rvs167 mutations.
YBR110W	[ALG1] Mannosyltransferase, involved in asparagine-linked glycosylation in the endoplasmic reticulum (ER); essential for viability, mutation is functionally complemented by human ortholog beta-1,4-mannosyltransferase
YCR034W	[FEN1] Fatty acid elongase, involved in sphingolipid biosynthesis; acts on fatty acids of up to 24 carbons in length; mutations have regulatory effects on 1,3-beta-glucan synthase, vacuolar ATPase, and the secretory pathway Null mutant is viable; slow growth; fenpropimorph resistant; resistant to a pneumocandin B0 analog (L-733,560); mating and sporulation defects; synthetic lethality with ELO3
YDR331W	[GPI8] ER membrane glycoprotein subunit of the glycosylphosphatidylinositol transamidase complex that adds glycosylphosphatidylinositol (GPI) anchors to newly synthesized proteins; human PIG-K protein is a functional homolog
YJL196C	[ELO1] Elongase I, medium-chain acyl elongase, catalyzes carboxy-terminal elongation of unsaturated C12-C16 fatty acyl-CoAs to C16-C18 fatty acids elongase Null mutant is viable, but shows no growth on media supplemented with less than 16-C saturated fatty acid in a fatty acid synthase minus background
YKL065C	[YET1] Endoplasmic reticulum transmembrane protein, homolog of human BAP31 protein homolog of mammalian BAP31
YJL117W	[PHO86] Endoplasmic reticulum (ER) resident protein required for ER exit of the high-affinity phosphate transporter Pho84p, specifically required for packaging of Pho84p into COPII vesicles Null mutant is viable and expresses repressible acid phosphatase in high phosphate medium; pho86 pho87 double mutant and pho86 pho88 double mutant constituvely synthesize repressible acid phosphatase and are arsenate-resistant; pho84 pho86 pho87 triple mutant grows more slowly than pho84 mutant
YDL015C	[TSC13] Enoyl reductase that catalyzes the last step in each cycle of very long chain fatty acid elongation, localizes to the ER, highly enriched in a structure marking nuclear-vacuolar junctions, coimmunoprecipitates with elongases Fen1p and Sur4p enoyl reductase
YPL076W	[GPI2] Protein involved in the synthesis of N-acetylglucosaminyl phosphatidylinositol (GlcNAc-PI), the first intermediate in the synthesis of glycosylphosphatidylinositol (GPI) anchors; homologous to the human PIG-C protein
YGR060W	[ERG25] C-4 methyl sterol oxidase, catalyzes the first of three steps required to remove two C-4 methyl groups from an intermediate in ergosterol biosynthesis; mutants accumulate the sterol intermediate 4,4-dimethylzymosterol C-4 sterol methyl oxidase
YHR140W	
YMR215W	[GAS3] Putative 1,3-beta-glucanosyltransferase, has similarity to Gas1p; localizes to the cell wall

0059	GO_TERM:[ribonuclease MRP complex] P-Value:3.0e-29 OVERLAP:[RNase P] <440.14.10> SIZE:10
**YIR015W**	[RPR2] Subunit of nuclear RNase P, which cleaves tRNA precursors to generate mature 5' ends; not shared between Rnase MRP and Rnase P, in contrast to all other Rnase P protein subunits Nuclear RNase P subunit Null mutant is inviable; transient depletions cause loss of RNase P
**YBL018C**	[POP8] Subunit of both RNase MRP, which cleaves pre-rRNA, and nuclear RNase P, which cleaves tRNA precursors to generate mature 5' ends Nuclear RNase P subunit|RNase MRP subunit Null mutant is inviable; transient depletions cause loss of RNase P and RNase MRP function
**YNL282W**	[POP3] Subunit of both RNase MRP, which cleaves pre-rRNA, and nuclear RNase P, which cleaves tRNA precursors to generate mature 5' ends Nuclear RNase P subunit|RNase MRP subunit
**YBR167C**	[POP7] Subunit of both RNase MRP, which cleaves pre-rRNA, and nuclear RNase P, which cleaves tRNA precursors to generate mature 5' ends Nuclear RNase P subunit|RNase MRP subunit Null mutant is inviable; transient depletions cause loss of RNase P and RNase MRP function
YDR478W	[SNM1] Subunit of RNase MRP, which cleaves pre-rRNA; not shared between Rnase MRP and nuclear Rnase P, in contrast to all other Rnase MRP protein subunits; binds to the NME1 RNA subunit of Rnase MRP RNase MRP subunit
**YGR030C**	[POP6] Subunit of both RNase MRP, which cleaves pre-rRNA, and nuclear RNase P, which cleaves tRNA precursors to generate mature 5' ends Nuclear RNase P subunit|RNase MRP subunit Null mutant is inviable; transient depletions cause loss of RNase P and RNase MRP function
YLR145W	[RMP1] Protein required for cell viability, component of RNase MRP, which is involved in RNA processing in mitochondria Protein component of RNase MRP complex

**YAL033W**	[POP5] Subunit of both RNase MRP, which cleaves pre-rRNA, and nuclear RNase P, which cleaves tRNA precursors to generate mature 5' ends Nuclear RNase P subunit|RNase MRP subunit Null mutant is inviable; transient depletion of Pop5p causes loss of RNase P and RNase MRP function
**YHR062C**	[RPP1] Subunit of both RNase MRP, which cleaves pre-rRNA, and nuclear RNase P, which cleaves tRNA precursors to generate mature 5' ends Nuclear RNase P subunit|RNase MRP subunit
**YBR257W**	[POP4] Subunit of both RNase MRP, which cleaves pre-rRNA, and nuclear RNase P, which cleaves tRNA precursors to generate mature 5' ends; binds to the RPR1 RNA subunit in Rnase P Nuclear RNase P subunit|RNase MRP subunit
**YNL221C**	[POP1] Subunit of both RNase MRP, which cleaves pre-rRNA, and nuclear RNase P, which cleaves tRNA precursors to generate mature 5' ends; binds to the RPR1 RNA subunit in Rnase P Nuclear RNase P subunit|RNase MRP subunit Null mutant is inviable; temperature-sensitive mutant shows defect in processing pre-tRNAs and pre-rRNA at nonpermissive temperature

0060	GO_TERM:[protein complex assembly] P-Value:2.3e-02 OVERLAP:[F0/F1 ATP synthase (complex V)] <420.50> SIZE:18
YNL313C	
**YJR121W**	[ATP2] Beta subunit of the F1 sector of mitochondrial F1F0 ATP synthase, which is a large, evolutionarily conserved enzyme complex required for ATP synthesis F(1)F(0)-ATPase complex beta subunit Mutant displays a growth defect on glycerol
YJL180C	[ATP12] Molecular chaperone, required for the assembly of alpha and beta subunits into the F1 sector of mitochondrial F1F0 ATP synthase greatly reduced ATPase activity; alpha and beta subunits of F1-ATPase accumulate in mitochondria as inactive aggregates
YNL315C	[ATP11] Molecular chaperone, required for the assembly of alpha and beta subunits into the F1 sector of mitochondrial F1F0 ATP synthase greatly reduced ATPase activity; alpha and beta subunits of F1-ATPase accumulate in mitochondria as inactive aggregates

0061	GO_TERM:[proton-transporting ATP synthase complex, coupling factor F(o) (sensu Eukaryota)] P-Value:1.7e-13 OVERLAP:[F0/F1 ATP synthase (complex V)] <420.50> SIZE:18
**YDR298C**	[ATP5] Subunit 5 of the stator stalk of mitochondrial F1F0 ATP synthase, which is a large, evolutionarily conserved enzyme complex required for ATP synthesis; homologous to bovine subunit OSCP (oligomycin sensitivity-conferring protein) ATP synthase subunit 5|oligomycin sensitivity-conferring protein null mutant is viable, but unable to grow on glycerol; exhibits high level of genetic instability
**YKL016C**	[ATP7] Subunit d of the stator stalk of mitochondrial F1F0 ATP synthase, which is a large, evolutionarily conserved enzyme complex required for ATP synthesis ATP synthase d subunit glycerol minus phenotype; mitochondria have no detectable oligomycin-sensitive ATPase activity; F1 loosely bound to the membranous portion
**YPR020W**	[ATP20] Subunit g of the mitochondrial F1F0 ATP synthase, which is a large enzyme complex required for ATP synthesis; associated only with the dimeric form of ATP synthase ATP synthase subunit g homolog Null mutant is viable but exhibits a reduced growth rate on respiratory substrates
**YDR377W**	[ATP17] Subunit f of the F0 sector of mitochondrial F1F0 ATP synthase, which is a large, evolutionarily conserved enzyme complex required for ATP synthesis ATP synthase subunit f No growth on glycerol
**YML081C-A**	[ATP18] Subunit of the mitochondrial F1F0 ATP synthase, which is a large enzyme complex required for ATP synthesis; termed subunit I or subunit j; does not correspond to known ATP synthase subunits in other organisms ATP synthase associated protein Null mutant is viable, deficient in oligomycin-sensitive ATPase activity, and is unable to grow on nonfermentable carbon sources.

0062	GO_TERM:[proton-transporting ATP synthase complex (sensu Eukaryota)] P-Value:1.4e-09 OVERLAP:[F0/F1 ATP synthase (complex V)] <420.50> SIZE:18
**YBL099W**	[ATP1] Alpha subunit of the F1 sector of mitochondrial F1F0 ATP synthase, which is a large, evolutionarily conserved enzyme complex required for ATP synthesis F1F0-ATPase alpha subunit null mutant is viable; grows slowly on fermentable carbon sources; exhibits delayed kinetics of protein import for several mitochondrial precursors
**YDR322C-A**	[TIM11] Subunit e of mitochondrial F1F0-ATPase, which is a large, evolutionarily conserved enzyme complex required for ATP synthesis; essential for the dimeric state of ATP synthase mitochondrial F1F0-ATPase subunit e
**Q0080**	[ATP8] Subunit 8 of the F0 sector of mitochondrial inner membrane F1-F0 ATP synthase, encoded on the mitochondrial genome ATP synthase subunit 8 Loss of respiratory function
**YPL078C**	[ATP4] Subunit b of the stator stalk of mitochondrial F1F0 ATP synthase, which is a large, evolutionarily conserved enzyme complex required for ATP synthesis F(1)F(0)-ATPase complex subunit b Null mutant is viable but is oxidative phosphorylation deficient, is unable to grow on glycerol, shows an F1 loosely bound to mitochondrial membrane, lacks subunit 6 in F0, has a five times lower cytochrome oxidase activity, produces a high percentage of sponteneous rho- mutants, and is oligomycin-insensitive

0063	GO_TERM:[TRAPP complex] P-Value:1.8e-30 OVERLAP:[TRAPP (Transport Protein Particle) complex] <260.60> SIZE:10
YJL044C	[GYP6] GTPase-activating protein (GAP) for the yeast Rab family member, Ypt6p; involved in vesicle mediated protein transport GTPase activating protein (GAP) for Ypt6
**YMR218C**	[TRS130] One of 10 subunits of the transport protein particle (TRAPP) complex of the cis-Golgi which mediates vesicle docking and fusion; involved in ER to Golgi membrane traffic; mutation activates transcription of OCH1
**YML077W**	[BET5] Component of the TRAPP (transport protein particle) complex, which plays an essential role in the vesicular transport from endoplasmic reticulum to Golgi TRAPP 18kDa component
**YGR166W**	[KRE11] Protein involved in biosynthesis of cell wall beta-glucans; subunit of the TRAPP (transport protein particle) complex, which is involved in the late steps of endoplasmic reticulum to Golgi transport Null mutant is viable; killer toxin resistant; reduced levels of 1,6-beta-glucan in cell wall
**YDR108W**	[GSG1] Subunit of TRAPP (transport protein particle), a multi-subunit complex involved in targeting and/or fusion of ER-to-Golgi transport vesicles with their acceptor compartment; protein has late meiotic role, following DNA replication
**YDR407C**	[TRS120] One of 10 subunits of the transport protein particle (TRAPP) complex of the cis-Golgi which mediates vesicle docking and fusion; involved in endoplasmic reticulum (ER) to Golgi membrane traffic
**YDR246W**	[TRS23] One of 10 subunits of the transport protein particle (TRAPP) complex of the cis-Golgi which mediates vesicle docking and fusion; involved in endoplasmic reticulum (ER) to Golgi membrane traffic; human homolog is TRAPPC4
**YOR115C**	[TRS33] One of 10 subunits of the transport protein particle (TRAPP) complex of the cis-Golgi which mediates vesicle docking and fusion; involved in endoplasmic reticulum (ER) to Golgi membrane traffic
**YDR472W**	[TRS31] One of 10 subunits of the transport protein particle (TRAPP) complex of the cis-Golgi which mediates vesicle docking and fusion; involved in endoplasmic reticulum (ER) to Golgi membrane traffic
**YBR254C**	[TRS20] One of 10 subunits of the transport protein particle (TRAPP) complex of the cis-Golgi which mediates vesicle docking and fusion; mutations in the human homolog cause the spondyloepiphyseal dysplasia tarda (SEDL) disorder
**YKR068C**	[BET3] Hydrophilic protein that acts in conjunction with SNARE proteins in targeting and fusion of ER to Golgi transport vesicles; component of the TRAPP (transport protein particle) complex transport protein particle (TRAPP) component

0064	GO_TERM:[regulation of actin polymerization and/or depolymerization] P-Value:2.2e-08 OVERLAP:[Actin-associated proteins] <140.20.20> SIZE:25
YBR264C	[YPT10] GTP binding protein that contains the PEST signal sequence specific for proteolytic enzymes; may be involved in vesicular transport; overexpression leads to accumulation of Golgi-like cisternae with budding vesicles
YER071C	
YFR016C	
**YGR080W**	[TWF1] Twinfilin, highly conserved actin monomer-sequestering protein involved in regulation of the cortical actin cytoskeleton, composed of two cofilin-like regions, localizes actin monomers to sites of rapid filament assembly twinfilin A, an actin monomer sequestering protein Null mutant is viable, twf1 null cof1-22 mutants exhibit synthetic lethality
YIR003W	
**YIL034C**	[CAP2] Beta subunit of the capping protein (CP) heterodimer (Cap1p and Cap2p) which binds to the barbed ends of actin filaments preventing further polymerization; localized predominantly to cortical actin patches capping protein beta subunit Null mutant is viable, exhibits abnormal actin distribution (including loss of actin cables); round, large cells with heterogeneous size distribution; slower growing; chitin found over entire mother cell surface rather than restricted to the mother-bud junction
**YKL007W**	[CAP1] Alpha subunit of the capping protein (CP) heterodimer (Cap1p and Cap2p) which binds to the barbed ends of actin filaments preventing further polymerization; localized predominantly to cortical actin patches capping protein Null mutant is viable; severe deficit of actin cables and increased number of actin spots in the mother; round, relatively large cells

0065	GO_TERM:[endosome] P-Value:6.2e-06
YPR185W	[ATG13] Phosphorylated protein that interacts with Vac8p, required for the cytoplasm-to-vacuole targeting (Cvt) pathway and autophagy Defective in autophagy
YNL086W	
YKL061W	

YDR357C	
YGL079W	
YEL005C	[VAB2] Protein with a potential role in vacuolar function, as suggested by its ability to bind Vac8p; green fluorescent protein (GFP)-fusion protein localizes to the cytoplasm in a punctate pattern
YLR408C	

0066	GO_TERM:[alpha-1,6-mannosyltransferase complex] P-Value:3.2e-13 OVERLAP:[m-AAA protease complex] <350.10.10> SIZE:2
YGR231C	[PHB2] Subunit of the prohibitin complex (Phb1p-Phb2p), a 1.2 MDa ring-shaped inner mitochondrial membrane chaperone that stabilizes newly synthesized proteins; determinant of replicative life span; involved in mitochondrial segregation mammalian BAP37 and S. cerevisiae Phb1p homolog|prohibitin homolog
YGR132C	[PHB1] Subunit of the prohibitin complex (Phb1p-Phb2p), a 1.2 MDa ring-shaped inner mitochondrial membrane chaperone that stabilizes newly synthesized proteins; determinant of replicative life span; involved in mitochondrial segregation Phb2p homolog|mitochondrial protein
**YER017C**	[AFG3] Component, with Yta12p, of the mitochondrial inner membrane m-AAA protease that mediates degradation of misfolded or unassembled proteins and is also required for correct assembly of mitochondrial enzyme complexes ATP dependent metalloprotease nuclear petite phenotype; loss of repspiratory competence
**YMR089C**	[YTA12] Component, with Afg3p, of the mitochondrial inner membrane m-AAA protease that mediates degradation of misfolded or unassembled proteins and is also required for correct assembly of mitochondrial enzyme complexes ATPase|CDC48/PAS1/SEC18 (AAA) family Null mutant is viable, petite grossly deficient in mitochondrial respiratory and ATPase complexes, yet synthesizes all proteins encoded by mitochondrial DNA
YGL167C	[PMR1] High affinity Ca2+/Mn2+ P-type ATPase required for Ca2+ and Mn2+ transport into Golgi; involved in Ca2+ dependent protein sorting and processing; mutations in human homolog ATP2C1 cause acantholytic skin condition Hailey-Hailey disease Ca2+ ATPase pmr1 null mutants suppress ypt1-1
YEL036C	[ANP1] Subunit of the alpha-1,6 mannosyltransferase complex; type II membrane protein; has a role in retention of glycosyltransferases in the Golgi; involved in osmotic sensitivity and resistance to aminonitrophenyl propanediol Null mutant has altered mannoprotein glycosylation and a defect in N-linked outerchain glycan mannosylation; other mutant phenotypes include aminonitrophenyl propanediol resistance, vanadate resistance, hygromycin B sensitive and a clumpy growth morphology.
YPL050C	[MNN9] Subunit of Golgi mannosyltransferase complex also containing Anp1p, Mnn10p, Mnn11p, and Hoc1p that mediates elongation of the polysaccharide mannan backbone; forms a separate complex with Van1p that is also involved in backbone elongation required for complex glycosylation mnn9 is lethal in combination with chs3.
YJR075W	[HOC1] Alpha-1,6-mannosyltransferase involved in cell wall mannan biosynthesis; subunit of a Golgi-localized complex that also contains Anp1p, Mnn9p, Mnn11p, and Mnn10p; identified as a suppressor of a cell lysis sensitive pkc1-371 allele mannosyltransferase (putative) Null mutant is viable but is hypersensitive to calcofluor white and hygromycin B and has lowered restrictive temperature in a pkc1-371 background; high copy suppressor of pkc1-371
YDR245W	[MNN10] Subunit of a Golgi mannosyltransferase complex also containing Anp1p, Mnn9p, Mnn11p, and Hoc1p that mediates elongation of the polysaccharide mannan backbone; membrane protein of the mannosyltransferase family galactosyltransferase Null mutant is viable, is larger than wild-type cells, is deficient in bud emergence, and depends upon an intact morphogenesis checkpoint control to survive
YJL183W	[MNN11] Subunit of a Golgi mannosyltransferase complex that also contains Anp1p, Mnn9p, Mnn10p, and Hoc1p, and mediates elongation of the polysaccharide mannan backbone; has homology to Mnn10p mannosyltransferase complex component Null mutant is viable, exhibits defects in mannan synthesis

0067	GO_TERM:[retromer complex] P-Value:6.0e-16 OVERLAP:[Vps35/Vps29/Vps26 complex] <260.30.30.10> SIZE:3
YKL195W	[MIA40] Essential protein of the mitochondrial intermembrane space, involved in import and assembly of intermembrane space proteins
YOR132W	[VPS17] Subunit of the membrane-associated retromer complex essential for endosome-to-Golgi retrograde protein transport; peripheral membrane protein that assembles onto the membrane with Vps5p to promote vesicle formation Null mutant is viable, exhibits defect in vacuolar morphology and protein sorting
**YHR012W**	[VPS29] Endosomal protein that is a subunit of the membrane-associated retromer complex essential for endosome-to-Golgi retrograde transport; forms a subcomplex with Vps35p and Vps26p that selects cargo proteins for endosome-to-Golgi retrieval Defective for sorting of soluble hydrolases to the vacuole. Mislocalisation of the vacuolar hydrolase sorting receptor Vps10p.
YOR069W	[VPS5] Nexin-1 homolog required for localizing membrane proteins from a prevacuolar/late endosomal compartment back to the late Golgi apparatus; structural component of the retromer membrane coat complex; forms a retromer subcomplex with Vps17p simialr to sorting nexin I Null mutant missorts and secretes soluble vacuolar proteins, contains fragmented vacuoles, and mislocalizes carboxypeptidase and Vps10p.

**YJL053W**	[PEP8] Vacuolar protein sorting protein that forms part of the multimeric membrane-associated retromer complex along with Vps35p, Vps29p, Vps17p, and Vps5p; essential for endosome-to-Golgi retrograde protein transport vacuolar protein similar to mouse gene H<beta>58 Null mutant is viable but is defective in processing of soluble vacuole proteases due to inability of soluble vacuolar hydrolase to reach the vacuole
**YJL154C**	[VPS35] Endosomal protein that is a subunit of the membrane-associated retromer complex essential for endosome-to-Golgi retrograde transport; forms a subcomplex with Vps26p and Vps29p that selects cargo proteins for endosome-to-Golgi retrieval retromer complex component Null mutant is viable, exhibits defects in sorting of vacuolar carboxypeptidase Y, proteinase A, proteinase B, and alkaline phosphatase

0068	GO_TERM:[Rab-protein geranylgeranyltransferase complex] P-Value:3.5e-07 OVERLAP:[Geranylgeranyltransferase II (GGTase II)] <180.30> SIZE:3
**YJL031C**	[BET4] Alpha subunit of Type II geranylgeranyltransferase required for vesicular transport between the endoplasmic reticulum and the Golgi; provides a membrane attachment moiety to Rab-like proteins Ypt1p and Sec4p geranylgeranyltransferase type II alpha subunit (PGGTase-II, alpha subunit)
**YOR370C**	[MRS6] Rab escort protein, forms a complex with the Ras-like small GTPase Ypt1p that is required for the prenylation of Ypt1p by protein geranylgeranyltransferase type II (Bet2p-Bet4p) rab geranylgeranyltransferase regulatory subunit Null mutant is inviable; multicopy MRS6 causes a mild pet- phenotype; multicopy MRS6 suppresses the pet- phenotype of mrs2-1 mutants; suppresses ts ypt1
**YPR176C**	[BET2] Beta subunit of Type II geranylgeranyltransferase required for vesicular transport between the endoplasmic reticulum and the Golgi; provides a membrane attachment moiety to Rab-like proteins Ypt1p and Sec4p geranylgeranyltransferase type II beta subunit

0069	GO_TERM:[pre-autophagosomal structure] P-Value:5.7e-06
YDL113C	[ATG20] Protein required for transport of aminopeptidase I (Lap4p) through the cytoplasm-to-vacuole targeting pathway; binds phosphatidylinositol-3-phosphate, involved in localization of membranes to the preautophagosome, potential Cdc28p substrate PX domain-containing protein that binds Apg17 and Cvt13, and is required for import of precursor Ape1. Null: The cvt20 mutant accumulates precursor Ape1 but is normal for autophagy.. Other phenotypes: A mutation of a conserved tyrosine to alanine in the PX domain abolishes binding to PtdIns(3)P.
YJL036W	[SNX4] Sorting nexin, involved in the retrieval of late-Golgi SNAREs from the post-Golgi endosome to the trans-Golgi network and in cytoplasm to vacuole transport; contains a PX domain; forms complex with Snx41p and Atg20p Defective in maturation of the vacuolar protein, aminopeptidase I

0070	GO_TERM:[integral to Golgi membrane] P-Value:1.0e-03
YDR084C	[TVP23] Integral membrane protein localized to late Golgi vesicles along with the v-SNARE Tlg2p; green fluorescent protein (GFP)-fusion protein localizes to the cytoplasm in a punctate pattern integral membrane protein Null: no notable phenotype
YHR105W	[YPT35] Endosomal protein of unknown function that contains a phox (PX) homology domain and binds to both phosphatidylinositol-3-phosphate (PtdIns(3)P) and proteins involved in ER-Golgi or vesicular transport
YDR100W	[TVP15] Integral membrane protein localized to late Golgi vesicles along with the v-SNARE Tlg2p integral membrane protein Null: no notable phenotype
YGL161C	[YIP5] Protein that interacts with Rab GTPases; computational analysis of large-scale protein-protein interaction data suggests a possible role in vesicle-mediated transport
YPL095C	[EEB1] Acyl-coenzymeA:ethanol O-acyltransferase responsible for the major part of short-chain fatty acid ethyl ester production during fermentation Acyl-coenzymeA:ethanol O-acyltransferase

0071	GO_TERM:[late endosome] P-Value:2.6e-07
YNL093W	[YPT53] GTPase, similar to Ypt51p and Ypt52p and to mammalian rab5; required for vacuolar protein sorting and endocytosis GTP-binding protein|rab family
YKR014C	[YPT52] GTPase, similar to Ypt51p and Ypt53p and to mammalian rab5; required for vacuolar protein sorting and endocytosis Null mutant is viable; ypt51 ypt52 double deletion exacerbates the temperature sensitivity and vacuolar protein sorting defects of ypt51 deletion
YOR089C	[VPS21] GTPase required for transport during endocytosis and for correct sorting of vacuolar hydrolases; localized in endocytic intermediates; detected in mitochondria; geranylgeranylation required for membrane association; mammalian Rab5 homolog small GTP-binding protein Null mutant is viable, temperature-sensitive, missorts multiple vacuolar proteins, accumulate 40-50 nm vesicles, and contain a large vacuole

0072	GO_TERM:[GTPase activity] P-Value:3.9e-04

YER031C	[YPT31] GTPase of the Ypt/Rab family, very similar to Ypt32p; involved in the exocytic pathway; mediates intra-Golgi traffic or the budding of post-Golgi vesicles from the trans-Golgi GTPase|YPT32 homolog|ras homolog ypt31 ypt32 double deletion mutants are inviable
YFL038C	[YPT1] Ras-like small GTPase, involved in the ER-to-Golgi step of the secretory pathway; complex formation with the Rab escort protein Mrs6p is required for prenylation of Ypt1p by protein geranylgeranyltransferase type II (Bet2p-Bet4p) GTP-binding protein|ras homolog|similar to mammalian Rab1A protein Null mutant is inviable, at non-permissive temp, ts and cs mutants accumulate ER membranes and small vesicles, fail to process invertase and other secreted proteins, and show cytoskeletal defects; ypt1 causes lethality during nitrogen starvation

0073	GO_TERM:[transport vesicle] P-Value:1.5e-07
YFL005W	[SEC4] Secretory vesicle-associated Rab GTPase essential for exocytosis; associates with the exocyst component Sec15p and may regulate polarized delivery of transport vesicles to the exocyst at the plasma membrane ras homolog|small GTP binding protein null is inviable; conditional mutants show defects in secretion and accumulation of post-Golgi vesicles under non-permissive conditions|accumulates secretory vesicles
YNL044W	[YIP3] Protein localized to COPII vesicles, proposed to be involved in ER to Golgi transport; interacts with members of the Rab GTPase family and Yip1p; also interacts with Rtn1p
YGL198W	[YIP4] Protein that interacts with Rab GTPases; computational analysis of large-scale protein-protein interaction data suggests a possible role in vesicle-mediated transport
YGR172C	[YIP1] Integral membrane protein required for the biogenesis of ER-derived COPII transport vesicles; interacts with Yif1p and Yos1p; localizes to the Golgi, the ER, and COPII vesicles
YNL263C	[YIF1] Integral membrane protein required for the fusion of ER-derived COPII transport vesicles with the Golgi; interacts with Yip1p and Yos1p; localizes to the Golgi, the ER, and COPII vesicles

0074	GO_TERM:[AP-3 adaptor complex] P-Value:1.2e-13 OVERLAP:[AP-3 complex] <260.20.30> SIZE:4
**YBR288C**	[APM3] Mu3-like subunit of the clathrin associated protein complex (AP-3); functions in transport of alkaline phosphatase to the vacuole via the alternate pathway clathrin associated protein complex medium subunit Null mutant is viable, even combined with apm1 and apm2
**YJL024C**	[APS3] Small subunit of the clathrin-associated adaptor complex AP-3, which is involved in vacuolar protein sorting; related to the sigma subunit of the mammalian clathrin AP-3 complex; suppressor of loss of casein kinase 1 function Null mutant is viable, rescues yck1,yck2 double mutant
**YGR261C**	[APL6] Beta3-like subunit of the yeast AP-3 complex; functions in transport of alkaline phosphatase to the vacuole via the alternate pathway; exists in both cytosolic and peripherally associated membrane-bound pools clathrin assembly complex beta adaptin component (putative) Null mutant is viable, null rescues yck1 yck2 double mutant
**YPL195W**	[APL5] Delta adaptin-like subunit of the clathrin associated protein complex (AP-3); functions in transport of alkaline phosphatase to the vacuole via the alternate pathway, suppressor of loss of casein kinase 1 function clathrin assembly complex AP-3 adaptin component delta-like subunit Null mutant is viable, rescues yck1,yck2 double mutant

0075	GO_TERM:[C-terminal protein lipidation] P-Value:9.5e-09 OVERLAP:[Aut2p/Aut7p complex] <260.100> SIZE:2
**YBL078C**	[ATG8] Protein required for autophagy; modified by the serial action of Atg4p, Atg7p, and Atg3p, and conjugated at the C terminus with phosphatidylethanolamine, to become the form essential for generation of autophagosomes similar to LC3, a microtubule-associated protein from rat Null mutant is viable but lacks autophagocytosis and is unable to sporulate. AUT7 is a suppressor of mutant phenotypes of aut2-1 cells. Uptake of precursor Aminopeptidase I into the vacuole depends on Aut2p and Aut7p.
YLL042C	[ATG10] E2-like conjugating enzyme that mediates formation of the Atg12p-Atg5p conjugate, which is a critical step in autophagy protein-conjugating enzyme Defective autophagy, apg10-1 allele shows reduced viablility under starvation conditions
YHR171W	[ATG7] Autophagy-related protein and dual specificity member of the E1 family of ubiquitin-activating enzymes; mediates the conjugation of Atg12p with Atg5p and Atg8p with phosphatidylethanolamine, required steps in autophagosome formation. Null mutant is viable, defective in autophagy
YNR007C	[ATG3] Protein involved in autophagy; E2-like enzyme that plays a role in formation of Atg8p-phosphatidylethanolamine conjugates, which are involved in membrane dynamics during autophagy Null mutant is viable, defective in starvation-induced bulk flow transport of cytoplasmic proteins to the vacuole, exhibits decreased survival rates during starvation, defective in protein degradation in the vacuoles induced by nitrogen starvation, homozygous diploids fail to sporulate

0076	GO_TERM:[biopolymer catabolism] P-Value:1.5e-02
YDL227C	[HO] Site-specific endonuclease required for gene conversion at the MAT locus (homothallic switching) through the generation of a ds DNA break; expression restricted to mother cells in late G1 as controlled by Swi4p-Swi6p, Swi5p and Ash1p homothallic switching endonuclease Null mutant is viable and cannot undergo mating type switching
YML088W	[UFO1] F-box receptor protein, subunit of the Skp1-Cdc53-F-box receptor (SCF) E3 ubiquitin ligase complex; binds to phosphorylated Ho endonuclease, allowing its ubiquitylation by SCF and subsequent degradation F-box protein Null mutant is viable and UV sensitive

0077	GO_TERM:[Golgi apparatus] P-Value:2.4e-11
YGR142W	[BTN2] Cytosolic coiled-coil protein that modulates arginine uptake, interacts with Rhb1p, possible role in mediating pH homeostasis between the vacuole and plasma membrane H(+)-ATPase, may have a role in intracellular protein trafficking
YOL018C	[TLG2] Syntaxin-like t-SNARE that forms a complex with Tlg1p and Vti1p and mediates fusion of endosome-derived vesicles with the late Golgi; binds Vps45p, which prevents Tlg2p degradation and also facilitates t-SNARE complex formation tSNARE that affects a late Golgi compartment Null mutant is viable in SEY6210, exhibits endocytosis defect and loss of Kex2p
YBR164C	[ARL1] Soluble GTPase with a role in regulation of membrane traffic; regulates potassium influx; G protein of the Ras superfamily, similar to ADP-ribosylation factor ADP-ribosylation factor-like protein 1
YBL017C	[PEP1] Type I transmembrane sorting receptor for multiple vacuolar hydrolases; cycles between the late-Golgi and prevacuolar endosome-like compartments Type I integral membrane protein 166aa cytoplasmic tail, 1300 aa lumenal domain proteinase deficient
YDR468C	[TLG1] Essential t-SNARE that forms a complex with Tlg2p and Vti1p and mediates fusion of endosome-derived vesicles with the late Golgi; binds the docking complex VFT (Vps fifty-three) through interaction with Vps51p tSNARE that affects a late Golgi compartment Endocytosis defect and loss of Kex2p in SEY6210 background; Deletion may be lethal in some genetic backgrounds
YDR027C	[VPS54] Component of the GARP (Golgi-associated retrograde protein) complex, Vps51p-Vps52p-Vps53p-Vps54p, which is required for the recycling of proteins from endosomes to the late Golgi; potentially phosphorylated by Cdc28p Null mutant exhibits disrupted vacuole and conditional defects in microtubule assembly and cell growth; accumulates 20-50 acidic vesicles per cell that contain CPY, ALP and VMA subunits; sensitive to Mn, Zn, Cu, high pH, hygromycin, Cd, high temp (37C), low temp (14C) and FK506.
YKR020W	[VPS51] Component of the GARP (Golgi-associated retrograde protein) complex, Vps51p-Vps52p-Vps53p-Vps54p, which is required for the recycling of proteins from endosomes to the late Golgi; links the (VFT/GARP) complex to the SNARE Tlg1p function unknown Null: small critical cell size
YDR484W	[VPS52] Component of the GARP (Golgi-associated retrograde protein) complex, Vps51p-Vps52p-Vps53p-Vps54p, which is required for the recycling of proteins from endosomes to the late Golgi; involved in localization of actin and chitin Null mutant is viable, cold-sensitive growth phenotype, suppressor of actin mutation; aberrant organization of intracellular actin and deposition of chitin at the cell surface
YJL029C	[VPS53] Component of the GARP (Golgi-associated retrograde protein) complex, Vps51p-Vps52p-Vps53p-Vps54p, which is required for the recycling of proteins from endosomes to the late Golgi; required for vacuolar protein sorting hydrophilic protein that is peripherally associated with the late Golgi and forms a stable complex with Vps52p and Vps54p Null mutant is viable but is defective for growth at 37^*C. vps53 null mutants have fragmented vacuoles, missort and secrete CPY, and mislocalize late Golgi membrane proteins to the vacuole.

0078	GO_TERM:[SNAP receptor activity] P-Value:1.2e-12 OVERLAP:[v-SNAREs] <260.50.20> SIZE:8
YMR017W	[SPO20] Meiosis-specific subunit of the t-SNARE complex, required for prospore membrane formation during sporulation; similar to but not functionally redundant with Sec9p; SNAP-25 homolog SNAP 25 homolog Null mutant is viable, other mutant fails to form spores
YER143W	[DDI1] DNA damage-inducible v-SNARE binding protein, contains a ubiquitin-associated (UBA) domain, may act as a negative regulator of constitutive exocytosis, may play a role in S-phase checkpoint control
YPR032W	[SRO7] Protein with roles in exocytosis and cation homeostasis; functions in docking and fusion of post-Golgi vesicles with plasma membrane; homolog of Sro77p and Drosophila lethal giant larvae tumor suppressor; interacts with SNARE protein Sec9p yeast homolog of the Drosophila tumor suppressor, lethal giant larvae Null mutant is viable but is cs- in combination with sni2(YBL106c) null; sni1 sni2 double mutant has exocytic defect, accumulating post-Golgi vesicles. Acts as a multicopy suppressor of rho3.
**YOR327C**	[SNC2] Vesicle membrane receptor protein (v-SNARE) involved in the fusion between Golgi-derived secretory vesicles with the plasma membrane; member of the synaptobrevin/VAMP family of R-type v-SNARE proteins vesicle-associated membrane protein (synaptobrevin) homolog Null mutant is viable, snc1 snc2 double mutants are deficient in normal bulk secretion, accumulate large numbers of post-Golgi vesicles, and display a variety of conditional lethal phenotypes
**YAL030W**	[SNC1] Vesicle membrane receptor protein (v-SNARE) involved in the fusion between Golgi-derived secretory vesicles with the plasma membrane; proposed to be involved in endocytosis; member of the synaptobrevin/VAMP family of R-type v-SNARE proteins Snc2p homolog|synaptobrevin homolog Null mutant is viable; snc1 snc2 mutants are deficient in normal bulk secretion, accumulate large numbers of post-Golgi vesicles, and display a variety of conditional lethal phenotypes; snc1 mutations suppress loss of cap in strains possessing an activated ras2 allele

YDR164C	[SEC1] Sm-like protein involved in docking and fusion of exocytic vesicles through binding to assembled SNARE complexes at the membrane; localization to sites of secretion (bud neck and bud tip) is dependent on SNARE function SNARE docking complex subunit (putative) accumulates secretory vesicles
YPL232W	[SSO1] Plasma membrane t-SNARE involved in fusion of secretory vesicles at the plasma membrane; forms a complex, with t-SNARE Sec9p, that binds v-SNARE Snc2p; also required for sporulation; syntaxin homolog that is functionally redundant with Sso2p t-SNARE SSO1, SSO2 double null mutant is inviable; high copy number of either SSO1 or SSO2 suppresses mutations in late-acting sec genes (sec1,3,5,9,15)
YGR009C	[SEC9] t-SNARE protein important for fusion of secretory vesicles with the plasma membrane; similar to but not functionally redundant with Spo20p; SNAP-25 homolog t-SNARE (putative) accumulates secretory vesicles|An uncharacterized allele accumulates 100nm secretory vesicles and berkeley bodies and is defective in proteint transport to the cell surface. The sec9-4 allele has diploid-specific bud site selection defects.
YMR183C	[SSO2] Plasma membrane t-SNARE involved in fusion of secretory vesicles at the plasma membrane; syntaxin homolog that is functionally redundant with Sso1p t-SNARE SSO1, SSO2 double null mutant is inviable; high copy number of either SSO1 or SSO2 suppresses mutations in late-acting sec genes (sec1,3,5,9,15)
YNR049C	[MSO1] Probable component of the secretory vesicle docking complex, acts at a late step in secretion; shows genetic and physical interactions with Sec1p and is enriched in microsomal membrane fractions; required for sporulation Null mutant is viable, exhibits accumulation of secretory vesicles in the bud; mso1 null mutants exhibit double mutant inviability in combinaiton with sec1, sec2, and sec4 mutants

0079	GO_TERM:[membrane fusion] P-Value:1.2e-41 OVERLAP:[Exocyst complex] <160> SIZE:7
YDR233C	[RTN1] ER membrane protein that interacts with exocyst subunit Sec6p and with Yip3p; null mutant has an altered (mostly cisternal) ER morphology; has similarity to mammalian reticulon proteins and member of the RTNLA (reticulon-like A) subfamily
YBR200W	[BEM1] Protein containing SH3-domains, involved in establishing cell polarity and morphogenesis; functions as a scaffold protein for complexes that include Cdc24p, Ste5p, Ste20p, and Rsr1p Null mutant is viable; exhibits a defect in polarization in vegetative cells, exhibits decreased expression of FUS1
**YLR166C**	[SEC10] Essential 100kDa subunit of the exocyst complex (Sec3p, Sec5p, Sec6p, Sec8p, Sec10p, Sec15p, Exo70p, and Exo84p), which has the essential function of mediating polarized targeting of secretory vesicles to active sites of exocytosis exocyst complex component accumulates secretory vesicles
**YGL233W**	[SEC15] Essential 113kDa subunit of the exocyst complex (Sec3p, Sec5p, Sec6p, Sec8p, Sec10p, Sec15p, Exo70p, and Exo84p), which mediates polarized targeting of vesicles to active sites of exocytosis; Sec15p associates with Sec4p and vesicles exocyst complex component accumulates secretory vesicles|The sec15-1 allele exhibits temperature-sensitive growth and defects in the secretory pathway.
YBR102C	[EXO84] Essential protein with dual roles in spliceosome assembly and exocytosis; the exocyst complex (Sec3p, Sec5p, Sec6p, Sec8p, Sec10p, Sec15p, Exo70p, and Exo84p) mediates polarized targeting of secretory vesicles to active sites of exocytosis exocyst complex component|spliceosome assembly protein Null mutant is inviable, defective in secretion
**YPR055W**	[SEC8] Essential 121kDa subunit of the exocyst complex (Sec3p, Sec5p, Sec6p, Sec8p, Sec10p, Sec15p, Exo70p, and Exo84p), which has the essential function of mediating polarized targeting of secretory vesicles to active sites of exocytosis exocyst complex component secretion deficient|accumulates secretory vesicles
**YER008C**	[SEC3] Non-essential subunit of the exocyst complex (Sec3p, Sec5p, Sec6p, Sec8p, Sec10p, Sec15p, Exo70p, Exo84p) which mediates targeting of post-Golgi vesicles to sites of active exocytosis; Sec3p specifically is a spatial landmark for secretion exocyst complex component accumulates secretory vesicles
**YIL068C**	[SEC6] Essential 88kDa subunit of the exocyst complex (Sec3p, Sec5p, Sec6p, Sec8p, Sec10p, Sec15p, Exo70p, and Exo84p), which has the essential function of mediating polarized targeting of secretory vesicles to active sites of exocytosis exocyst complex component lethal|accumulates secretory vesicles
**YDR166C**	[SEC5] Essential 107kDa subunit of the exocyst complex (Sec3p, Sec5p, Sec6p, Sec8p, Sec10p, Sec15p, Exo70p, and Exo84p), which has the essential function of mediating polarized targeting of secretory vesicles to active sites of exocytosis exocyst complex component secretion deficient; Null is inviable|accumulates secretory vesicles
**YJL085W**	[EXO70] Essential 70kDa subunit of the exocyst complex (Sec3p, Sec5p, Sec6p, Sec8p, Sec10p, Sec15p, Exo70p, and Exo84p), which has the essential function of mediating polarized targeting of secretory vesicles to active sites of exocytosis exocyst complex component
YBR080C	[SEC18] ATPase required for the release of Sec17p during the 'priming' step in homotypic vacuole fusion and for ER to Golgi transport; homolog of the mammalian NSF ATPase|NSF|protein involved in protein transport between ER and Golgi
YDR323C	[PEP7] Multivalent adaptor protein that facilitates vesicle-mediated vacuolar protein sorting by ensuring high-fidelity vesicle docking and fusion, which are essential for targeting of vesicles to the endosome; required for vacuole inheritance three zinc fingers; cysteine rich regions of amino acids are essential for function Null mutant is viable but grows more slowly and is temperature-sensitive; defective in vacuole segregation; mislocalizes carboxypeptidase Y and other vacuolar proteins; shows loss of vacuolar acidity and defects in vacuolar morphology
YAL014C	[SYN8] Endosomal SNARE related to mammalian syntaxin 8 syntaxin family
YDR264C	[AKR1] Palmitoyl transferase involved in protein palmitoylation; acts as a negative regulator of pheromone response pathway; required for endocytosis of pheromone receptors; involved in cell shape control; contains ankyrin repeats ankyrin repeat-containing protein Null mutant is viable, exhibits slow growth, abnormal morphology, and partial activation of pheromone response; defective for endocytosis of Ste2p and Ste3p

YGL095C	[VPS45] Protein of the Sec1p/Munc-18 family, essential for vacuolar protein sorting; required for the function of Pep12p and the early endosome/late Golgi SNARE Tlg2p; essential for fusion of Golgi-derived vesicles with the prevacuolar compartment Null mutant is viable, defective in the segregation of vacuolar material into the developing daughter cell, has large central vacuoles
YOR036W	[PEP12] Target membrane receptor (t-SNARE) for vesicular intermediates traveling between the Golgi apparatus and the vacuole; controls entry of biosynthetic, endocytic, and retrograde traffic into the prevacuolar compartment; syntaxin c-terminal TMD|integral membrane protein proteinase deficient
YEL013W	[VAC8] Phosphorylated vacuolar membrane protein that interacts with Atg13p, required for the cytoplasm-to-vacuole targeting (Cvt) pathway; interacts with Nvj1p to form nucleus-vacuole junctions Defective in vacuole inheritance and aminopeptidase I targeting to the vacuole
YBL050W	[SEC17] Peripheral membrane protein required for vesicular transport between ER and Golgi and for the 'priming' step in homotypic vacuole fusion, part of the cis-SNARE complex; has similarity to alpha-SNAP secretion deficient
YBR131W	[CCZ1] Protein involved in vacuolar assembly, essential for autophagy and the cytoplasm-to-vacuole pathway Null mutant is viable, but is sensitive to caffeine, calcium and zinc; no sporulation in homozygous null diploids
YAL002W	[VPS8] Membrane-associated hydrophilic protein that interacts with the small GTPase, Vps21p, to facilitate soluble vacuolar protein localization; required for localization and trafficking of the CPY sorting receptor; contains a RING finger motif membrane-associated hydrophilic protein which contains a C-terminal cysteine-rich region that conforms to the H2 variant of the RING finger Zn2+ binding motif Null mutant is viable, missorts and secretes vacuolar hydrolases, overexpression of VPS21 partially suppresses vps8 null
YGL124C	[MON1] Protein required for fusion of cvt-vesicles and autophagosomes with the vacuole; associates, as a complex with Ccz1p, with a perivacuolar compartment; potential Cdc28p substrate null mutant is sensitive to monensin and brefeldin A
YKL196C	[YKT6] Vesicle membrane protein (v-SNARE) with acyltransferase activity; involved in trafficking to and within the Golgi, endocytic trafficking to the vacuole, and vacuolar fusion; membrane localization due to prenylation at the carboxy-terminus v-SNARE Null mutant is inviable. Depletion of Ykt6p results in the accumulation of the p1 precursor (endoplasmic reticulum form) of the vacuolar enzyme carboxypeptidase Y and morphological abnormalities consistent with a defect in secretion.
YLR093C	[NYV1] v-SNARE component of the vacuolar SNARE complex involved in vesicle fusion; inhibits ATP-dependent Ca(2+) transport activity of Pmc1p in the vacuolar membrane vacuolar v-SNARE
YML001W	[YPT7] GTPase; GTP-binding protein of the rab family; required for homotypic fusion event in vacuole inheritance, for endosome-endosome fusion, similar to mammalian Rab7 GTP-binding protein|rab family Null mutant is viable, characterized by highly fragmented vacuoles and differential defects of vacuolar transport and maturation
YMR197C	[VTI1] Protein involved in cis-Golgi membrane traffic; v-SNARE that interacts with two t-SNARES, Sed5p and Pep12p; required for multiple vacuolar sorting pathways interacts with two t-SNARES, Sed5p and Pep12p|v-SNARE
YGL212W	[VAM7] Component of the vacuole SNARE complex involved in vacuolar morphogenesis; SNAP-25 homolog; functions with a syntaxin homolog Vam3p in vacuolar protein trafficking heptad repeat motif|hydrophilic protein Null mutant is viable, exhibits prominent large vacuoles
YOR106W	[VAM3] Syntaxin-related protein required for vacuolar assembly; functions with Vam7p in vacuolar protein trafficking; member of the syntaxin family of proteins syntaxin family Null mutant is viable, defective in processing of vacuolar hydrolases.
YDR080W	[VPS41] Vacuolar membrane protein that is a subunit of the homotypic vacuole fusion and vacuole protein sorting (HOPS) complex; essential for membrane docking and fusion at the Golgi-to-endosome and endosome-to-vacuole stages of protein transport Null mutant is viable, associated with fragmented vacuoles, exhibits defective high affinity transport due to impaired Fet3p activity and also exhibits defects in the processing and sorting of multiple vacuolar hydrolases
YDL077C	[VAM6] Vacuolar protein that plays a critical role in the tethering steps of vacuolar membrane fusion by facilitating guanine nucleotide exchange on small guanosine triphosphatase Ypt7p Null mutant is viable but exhibits defects in processing vacuolar proteases and in maturation of vacuolar alkaline phosphatase. Mutants also exhibit a defective vacuolar morphology; they contain several small vesicles that stain with vacuolar markers.
YLR396C	[VPS33] ATP-binding protein that is a subunit of the homotypic vacuole fusion and vacuole protein sorting (HOPS) complex; essential for membrane docking and fusion at both the Golgi-to-endosome and endosome-to-vacuole stages of protein transport temperature sensitive, defective vacuolar morphology and protein localization, methionine auxotroph
YPL045W	[VPS16] Subunit of the homotypic vacuole fusion and vacuole protein sorting (HOPS) complex; part of the Class C Vps complex essential for membrane docking and fusion at both the Golgi-to-endosome and endosome-to-vacuole stages of protein transport Null mutant is viable, has a severe defect in vacuolar protein sorting, is temperature sensitive for growth, displays grossly abnormal vacuolar morphology, and possesses a defect in alpha-factor processing
YLR148W	[PEP3] Vacuolar peripheral membrane protein that promotes vesicular docking/fusion reactions in conjunction with SNARE proteins, required for vacuolar biogenesis, forms complex with Pep5p that mediates protein transport to the vacuole vacuolar membrane protein Null mutant is viable, exhibits growth defects at 37 degrees celsius, exhibits vacuolar protein sorting and processing and defects, exhibits decreased levels of protease A, protease B, and carboxylpeptidase Y antigens; decreased repressible alkaline phosphatase activity; null mutants contain very few normal vacuolelike organelles; homozygous null mutants are sporulation defective
YMR231W	[PEP5] Peripheral vacuolar membrane protein required for protein trafficking and vacuole biogenesis; forms complex with Pep3p that promotes vesicular docking/fusion reactions in conjunction with SNARE proteins, also interacts with Pep7p Zn-finger protein (putative)

0080	GO_TERM:[Golgi transport complex] P-Value:1.0e-24 OVERLAP:[Golgi transport complex] <260.20.40> SIZE:8
YGL038C	[OCH1] Mannosyltransferase of the cis-Golgi apparatus, initiates the polymannose outer chain elongation of N-linked oligosaccharides of glycoproteins alpha-1,6-mannosyltransferase Null mutant is viable, temperature sensitive, lacks mannose outer chains
**YNL041C**	[COG6] Component of the conserved oligomeric Golgi complex (Cog1p through Cog8p), a cytosolic tethering complex that functions in protein trafficking to mediate fusion of transport vesicles to Golgi compartments
**YER157W**	[COG3] Essential component of the conserved oligomeric Golgi complex (Cog1p through Cog8p), a cytosolic tethering complex that functions in protein trafficking to mediate fusion of transport vesicles to Golgi compartments Strains carrying the null allele are extremely slow growing; they display a severe growth defect at 25 or 30 degrees, and fail to grow at 14 or 37 degrees. In addition to defects in protein localization and sorting, sec34 mutants exhibit defects in polarization of filamentous actin.
**YGR120C**	[COG2] Essential component of the conserved oligomeric Golgi complex (Cog1p through Cog8p), a cytosolic tethering complex that functions in protein trafficking to mediate fusion of transport vesicles to Golgi compartments Null mutant shows severe growth defect at 30 degrees and is inviable at 21 degrees; sec35-1 allele is temperature-sensitive for growth
**YGL005C**	[COG7] Component of the conserved oligomeric Golgi complex (Cog1p through Cog8p), a cytosolic tethering complex that functions in protein trafficking to mediate fusion of transport vesicles to Golgi compartments
**YGL223C**	[COG1] Essential component of the conserved oligomeric Golgi complex (Cog1p through Cog8p), a cytosolic tethering complex that functions in protein trafficking to mediate fusion of transport vesicles to Golgi compartments
**YPR105C**	[COG4] Essential component of the conserved oligomeric Golgi complex (Cog1p through Cog8p), a cytosolic tethering complex that functions in protein trafficking to mediate fusion of transport vesicles to Golgi compartments
**YML071C**	[COG8] Component of the conserved oligomeric Golgi complex (Cog1p through Cog8p), a cytosolic tethering complex that functions in protein trafficking to mediate fusion of transport vesicles to Golgi compartments
**YNL051W**	[COG5] Component of the conserved oligomeric Golgi complex (Cog1p through Cog8p), a cytosolic tethering complex that functions in protein trafficking to mediate fusion of transport vesicles to Golgi compartments

0081	GO_TERM:[cell wall chitin metabolism] P-Value:1.6e-14 OVERLAP:[COPI] <260.30.10> SIZE:8
YDL137W	[ARF2] ADP-ribosylation factor, GTPase of the Ras superfamily involved in regulation of coated formation vesicles in intracellular trafficking within the Golgi; functionally interchangeable with Arf1p ADP-ribosylation factor 2
YDR358W	[GGA1] Golgi-localized protein with homology to gamma-adaptin, interacts with and regulates Arf1p and Arf2p in a GTP-dependent manner in order to facilitate traffic through the late Golgi ARF-binding protein Single and double knockouts are viable at both 30 C and 37 C. Cells lacking GGA1, GGA2 exhibit defects in invertase processing, vacuolar morphology, maturation of alpha-factor, and sorting of CPY, proteinase A to the vacuole, but not endocytosis.
**YDL192W**	[ARF1] ADP-ribosylation factor, GTPase of the Ras superfamily involved in regulation of coated formation vesicles in intracellular trafficking within the Golgi; functionally interchangeable with Arf2p ADP-ribosylation factor Null mutant is viable and shows slow growth, cold sensitivity and sensitivity to normally sublethal concentrations of fluoride ion in the medium.
YLR330W	[CHS5] Protein of unknown function, involved in chitin biosynthesis by regulating Chs3p localization, also involved in cell fusion during mating
YMR237W	[BCH1] Protein that colocalizes with clathrin-coated vesicles; involved in transport at the trans-Golgi
YBR023C	[CHS3] Chitin synthase III, catalyzes the transfer of N-acetylglucosamine (GlcNAc) to chitin; required for synthesis of the majority of cell wall chitin, the chitin ring during bud emergence, and spore wall chitosan chitin synthase 3
YKR027W	[BCH2] The authentic, non-tagged protein was localized to the mitochondria
YJL099W	[CHS6] Protein of unknown function, involved in chitin biosynthesis by regulating Chs3p localization
YOR299W	[BUD7] Protein involved in bud-site selection; diploid mutants display an axial-like budding pattern Diploid-specific heterogenous bud site selection

0082	GO_TERM:[retrograde vesicle-mediated transport, Golgi to ER] P-Value:2.8e-10
YOL086C	[ADH1] Alcohol dehydrogenase, fermentative isozyme active as homo- or heterotetramers; required for the reduction of acetaldehyde to ethanol, the last step in the glycolytic pathway alcohol dehydrogenase Null mutant is viable and sensitive to formaldehyde.

YDR189W	[SLY1] Hydrophilic protein involved in vesicle trafficking between the ER and Golgi; SM (Sec1/Munc-18) family protein that binds the tSNARE Sed5p and stimulates its assembly into a trans-SNARE membrane-protein complex t-SNARE-interacting protein that functions in ER-to-Golgi traffic Null mutant is inviable; SLY1-20, which differs from wild-type SLY1 by a single amino acid, is a single copy suppressor of loss of YPT1
YNL258C	[DSL1] Endoplasmic reticulum (ER)-localized peripheral membrane protein required for Golgi-to-ER retrograde traffic; component of the ER target site that interacts with coatomer, the major component of the COPI vesicle protein coat
YLR440C	[SEC39] Protein of unknown function proposed to be involved in protein secretion
YGL098W	[USE1] Essential SNARE protein localized to the ER, involved in retrograde traffic from the Golgi to the ER; forms a complex with the SNAREs Sec22p, Sec20p and Ufe1p
YGL145W	[TIP20] Peripheral membrane protein required for fusion of COPI vesicles with the ER, prohibits back-fusion of COPII vesicles with the ER, may act as a sensor for vesicles at the ER membrane transport protein that interacts with Sec20p; required for protein transport from the endoplasmic reticulum to the golgi apparatus
YDR498C	[SEC20] Membrane glycoprotein v-SNARE involved in retrograde transport from the Golgi to the ER; required for N- and O-glycosylation in the Golgi but not in the ER; forms a complex with the cytosolic Tip20p v-SNARE secretion deficient
YOR075W	[UFE1] t-SNARE required for ER membrane fusion and vesicular traffic, integral membrane protein that constitutes with Sec20p and Use1p the trimeric acceptor for R/v-SNAREs on Golgi-derived vesicles at the ER; part of Dsl1p complex t-SNARE (ER)

0083	GO_TERM:[ER to Golgi transport vesicle] P-Value:1.4e-20 OVERLAP:[COPII] <260.30.20> SIZE:11
**YAR002C-A**	[ERP1] Protein that forms a heterotrimeric complex with Erp2p, Emp24p, and Erv25p; member, along with Emp24p and Erv25p, of the p24 family involved in ER to Golgi transport and localized to COPII-coated vesicles p24 protein involved in membrane trafficking null mutant is viable; delayed transport of Gas1p and invertase
YGL054C	[ERV14] Protein localized to COPII-coated vesicles, involved in vesicle formation and incorporation of specific secretory cargo; required for the delivery of bud-site selection protein Axl2p to cell surface; related to Drosophila cornichon 14 kDa protein found on ER-derived vesicles Null mutant is viable but exhibits defects in sporulation (diploids) and bud site selection (haploids). Null mutants also retain the bud site selection marker, Axl2p, in the ER and exhibit slow recovery from selective to rich media.
YDL018C	[ERP3] Protein with similarity to Emp24p and Erv25p, member of the p24 family involved in ER to Golgi transport p24 protein involved in membrane trafficking
YNL049C	[SFB2] Probable component of COPII coated vesicles that binds to Sec23p; similar to and functionally redundant with Sec24p, but expressed at low levels; involved in ER to Golgi transport and in autophagy zinc finger protein (putative)
**YML012W**	[ERV25] Protein that forms a heterotrimeric complex with Erp1, Erp2p, and Emp24, member of the p24 family involved in endoplasmic reticulum to Golgi transport vesicle coat component Null mutant is viable, displays a selective defect in transport of secretory proteins from the ER to Golgi complex.
**YPL218W**	[SAR1] GTPase, GTP-binding protein of the ARF family, component of COPII coat of vesicles; required for transport vesicle formation during ER to Golgi protein transport ARF family|GTP-binding protein Null mutant is inviable. When overexpressed, wild-type SAR1 suppresses a sec12 mutation.
**YPL085W**	[SEC16] COPII vesicle coat protein required for ER transport vesicle budding and autophagosome formation; Sec16p is bound to the periphery of ER membranes and may act to stabilize initial COPII complexes; interacts with Sec23p, Sec24p and Sec31p vesicle coat component Null mutant is inviable; temperature-sensitive mutants accumulate Kar2 (BiP) and PDI at the nonpermissive temperature.
**YDL195W**	[SEC31] Essential phosphoprotein component (p150) of the COPII coat of secretory pathway vesicles, in complex with Sec13p; required for ER-derived transport vesicle formation COPII coat of secretory pathway vesicles component (p150)
**YIL109C**	[SEC24] Component of the Sec23p-Sec24p heterodimeric complex of the COPII vesicle coat; involved in ER to Golgi transport, cargo selection and autophagy; required for the binding of the Sec13 complex to ER membranes; homologous to Lst1p and Lss1p vesicle coat component
**YPR181C**	[SEC23] GTPase-activating protein; component of the Sec23p-Sec24p heterodimeric complex of the COPII vesicle coat, involved in ER to Golgi transport and autophagy; stimulates the GDP-bound form of Sar1p GTPase activating protein (GAP) Defective for ER to Golgi transport

0084	GO_TERM:[endoplasmic reticulum] P-Value:1.5e-03 OVERLAP:[COPII] <260.30.20> SIZE:11
YML130C	[ERO1] Glycoprotein required for oxidative protein folding in the endoplasmic reticulum Null mutant is inviable; in ero1-1(ts) mutants newly synthesized carboxypeptidase Y is retained in the ER and lacks disulfide bonds; ero1 mutants are hypersensitive to to the reductant DTT, whereas overexpression of ERO1 confers resistance to DTT, the oxidant diamide can restore growth and secretion in ero1 mutants
**YAL042W**	[ERV46] Protein localized to COPII-coated vesicles, forms a complex with Erv41p; involved in the membrane fusion stage of transport ER-Golgi transport vesicle protein
YBR229C	[ROT2] Glucosidase II catalytic subunit required for normal cell wall synthesis; mutations in rot2 suppress tor2 mutations, and are synthetically lethal with rot1 mutations glucosidase II Null mutant is inviable; rot2 mutations can suppress tor2 mutations; synthetically lethal with rot1

0085	GO_TERM:[ER to Golgi transport vesicle] P-Value:3.0e-04

YFL048C	[EMP47] Integral membrane component of endoplasmic reticulum-derived COPII-coated vesicles, which function in ER to Golgi transport 47 kDa type I transmembrane protein localized to the Golgi
YLR080W	[EMP46] Integral membrane component of endoplasmic reticulum-derived COPII-coated vesicles, which function in ER to Golgi transport homolog of the Golgi protein Emp47p

0086	GO_TERM:[SNAP receptor activity] P-Value:5.7e-09 OVERLAP:[v-SNAREs] <260.50.20> SIZE:8
YBR205W	[KTR3] Putative alpha-1,2-mannosyltransferase involved in O- and N-linked protein glycosylation; member of the KRE2/MNT1 mannosyltransferase family alpha-1,2-mannosyltransferase (putative)
YHR181W	[SVP26] Integral membrane protein of the early Golgi apparatus, may function to promote retention of proteins in the early Golgi compartment; mutation affects protein N-glycosylation and cell wall integrity integral membrane protein
YLR026C	[SED5] cis-Golgi t-SNARE syntaxin required for vesicular transport between the ER and the Golgi complex, binds at least 9 SNARE proteins syntaxin family Null mutant is inviable; cells depleted of Sed5p are unable to transport carboxypeptidase Y to the Golgi complex, and stop growing after a dramatic accumulation of ER
**YLR078C**	[BOS1] v-SNARE (vesicle specific SNAP receptor), localized to the endoplasmic reticulum membrane and necessary for vesicular transport from the ER to the Golgi v-SNARE
**YHL031C**	[GOS1] v-SNARE protein involved in Golgi transport, homolog of the mammalian protein GOS-28/GS28
YKL006C-A	[SFT1] Intra-Golgi v-SNARE, required for transport of proteins between an early and a later Golgi compartment v-SNARE

0087	GO_TERM:[COPI vesicle coat] P-Value:1.2e-22 OVERLAP:[COPI] <260.30.10> SIZE:8
YKR067W	[GPT2] Glycerol-3-phosphate acyltransferase located in both lipid particles and the ER; involved in the stepwise acylation of glycerol-3-phosphate and dihydroxyacetone, which are intermediate steps in lipid biosynthesis glycerol 3-phosphate/dihydroxyacetone phosphate dual substrate-specific sn-1 acyltransferase
YML067C	[ERV41] Protein localized to COPII-coated vesicles, forms a complex with Erv46p; involved in the membrane fusion stage of transport
YER122C	[GLO3] ADP-ribosylation factor GTPase activating protein (ARF GAP), involved in ER-Golgi transport; shares functional similarity with Gcs1p similar to Gcs1p and Sps18p|zinc finger protein
YLR268W	[SEC22] R-SNARE protein; assembles into SNARE complex with Bet1p, Bos1p and Sed5p; cycles between the ER and Golgi complex; involved in anterograde and retrograde transport between the ER and Golgi; synaptobrevin homolog null mutant is cold and heat sensitive. Defective in ER to Golgi transport.
YIL004C	[BET1] Type II membrane protein required for vesicular transport between the endoplasmic reticulum and Golgi complex; v-SNARE with similarity to synaptobrevins
**YNL287W**	[SEC21] Gamma subunit of coatomer, a heptameric protein complex that together with Arf1p forms the COPI coat; involved in ER to Golgi transport of selective cargo PEST sequence-containing protein|non-clathrin coat protein
**YGL137W**	[SEC27] Essential beta'-coat protein of the COPI coatomer, involved in ER-to-Golgi and Golgi-to-ER transport; contains WD40 domains that mediate cargo selective interactions; 45% sequence identity to mammalian beta'-COP yeast coatomer beta'-subunit
**YDL145C**	[COP1] Alpha subunit of COPI vesicle coatomer complex, which surrounds transport vesicles in the early secretory pathway coatomer complex gamma-alpha-COP alpha subunit Null mutant is inviable; other cop1 alleles show secretion and protein sorting defects
**YDR238C**	[SEC26] Essential beta-coat protein of the COPI coatomer, involved in ER-to-Golgi protein trafficking and maintenance of normal ER morphology; shares 43% sequence identity with mammalian beta-coat protein (beta-COP) yeast coatomer subunit
**YIL076W**	[SEC28] Epsilon-COP subunit of the coatomer; regulates retrograde Golgi-to-ER protein traffic; stabilizes Cop1p, the alpha-COP and the coatomer complex; non-essential for cell growth epsilon-COP coatomer subunit
**YFR051C**	[RET2] Delta subunit of the coatomer complex (COPI), which coats Golgi-derived transport vesicles; involved in retrograde transport between Golgi and ER ret2-1 mutant is thermosensitive and shows defects in retrieval of dilysine-tagged proteins from the Golgi back to the ER and, at the non-permissive temperature, in forward ER-to-Golgi transport
**YPL010W**	[RET3] Zeta subunit of the coatomer complex (COPI), which coats Golgi-derived transport vesicles; involved in retrograde transport between Golgi and ER vesicle coat component ret3-1 mutant is thermosensitive and shows defects in retrieval of dilysine-tagged proteins from the Golgi back to the ER

0088	GO_TERM:[mitochondrial inner membrane protein insertion complex] P-Value:1.3e-12 OVERLAP:[Tim22p-complex] <290.20.10> SIZE:5
**YHR005C-A**	[MRS11] Essential protein of the mitochondrial intermembrane space, forms a complex with Tim9p (TIM10 complex) that mediates insertion of hydrophobic proteins at the inner membrane, has homology to Mrs5p, which is also involved in this process Null mutant is inviable; depletion of Mrs11p results in accumulation of the precursor form of mitochondrial hsp60, inability to form spectrophotometrically detectable amounts of cytochromes and changes in the mitochondrial morphology; when overexpressed, restores respiration competence to yeast defective in the splicing of mitochondrial group II introns

YOR297C	[TIM18] Component of the mitochondrial Tim54p-Tim22p complex involved in insertion of polytopic proteins into the inner membrane; may function to stabilize the complex translocase
**YJL054W**	[TIM54] Component of the mitochondrial Tim54p-Tim22p complex involved in insertion of polytopic proteins into the inner membrane Null mutant is inviable; the tim54-1 allele is temperature-sensitive and at the nonpermissive temperature is defective in the insertion of proteins into the mitochondrial inner membrane.
**YBR091C**	[MRS5] Essential protein of the inner mitochondrial membrane, peripherally localized; component of the TIM22 complex, which is a twin-pore translocase that mediates insertion of numerous multispanning inner membrane proteins Null mutant is inviable. Mrs5p depletion causes accumulation of unprocessed precursors of the mitochondrial hsp60 protein and defects in all cytochrome complexes
**YDL217C**	[TIM22] Component of the mitochondrial Tim54p-Tim22p complex involved in insertion of polytopic proteins into the inner membrane
0089	GO_TERM:[mitochondrial outer membrane translocase complex] P-Value:6.9e-14 OVERLAP:[TOM - transport across the outer membrane] <290.10> SIZE:9
YDR375C	[BCS1] Protein of the mitochondrial inner membrane that functions as an ATP-dependent chaperone, required for the assembly of the cytochrome bc(1) complex from the Rip1p and Qcr10p proteins; member of the CDC48/PAS1/SEC18 ATPase family ATPase (AAA family) Gross reduction in the Rieske iron-sulfur subunit
**YPR133W-A**	[TOM5] Small mitochondrial outer membrane protein crucial to a binding relay for the import of proteins into mitochondria; subunit on the outer mouth of the TOM channel that accepts precursors from the receptors Tom20p and Tom22p Null mutant is viable but is temperature-sensitive and shows defects in import of mitochondrial preproteins; synthetically lethal with tom6, tom7, tom20, tom37, and tom70
**YNL131W**	[TOM22] Component of the TOM (translocase of outer membrane) complex responsible for initial import of mitochondrially directed proteins; acts as a receptor for precursor proteins and mediates interaction between the TOM and TIM complexes mitochondrial import receptor protein
**YGR082W**	[TOM20] Component of the TOM (translocase of outer membrane) complex responsible for recognition and initial import steps for all mitochondrially directed proteins; acts as a receptor for incoming precursor proteins 20 kDa mitochondrial outer membrane protein import receptor
**YMR203W**	[TOM40] Component of the TOM (translocase of outer membrane) complex responsible for recognition and initial import steps for all mitochondrially directed proteins; constitutes the core element of the protein conducting pore mitochondrial outer membrane protein Null mutant is inviable; cells accumulate uncleaved mitochondrial precursor proteins
**YNL121C**	[TOM70] Component of the TOM (translocase of outer membrane) complex responsible for recognition and initial import steps for all mitochondrially directed proteins; acts as a receptor for incoming precursor proteins 70 kDa mitochondrial specialized import receptor of the outer membrane Null mutant is viable but exhibits defects in mitochondrial import
0090	GO_TERM:[mitochondrial intermembrane space protein transporter complex] P-Value:5.3e-06
YGR181W	[TIM13] Mitochondrial intermembrane space protein, forms a complex with TIm8p that mediates import and insertion of a subset of polytopic inner membrane proteins; may prevent aggregation of incoming proteins in a chaperone-like manner
YJR135W-A	[TIM8] Mitochondrial intermembrane space protein mediating import and insertion of polytopic inner membrane proteins; homolog of human DDP1 (deafness dystonia peptide 1) which is mutated in the X-linked Mohr-Tranebjaerg syndrome mitochondrial protein import machinery subunit
0091	GO_TERM:[mitochondrial inner membrane presequence translocase complex] P-Value:2.2e-28 OVERLAP:[Tim17p-complex] <290.20.20> SIZE:2
YFL016C	[MDJ1] Protein involved in folding of mitochondrially synthesized proteins in the mitochondrial matrix; localizes to the mitochondrial inner membrane; member of the DnaJ family of molecular chaperones DnaJ homolog|involved in mitochondrial biogenesis and protein folding Null mutant is viable, displays a petite phenotype, loss of mitochondrial DNA, and inviability at 37 degrees C
YLR369W	[SSQ1] Mitochondrial hsp70-type molecular chaperone, required for assembly of iron/sulfur clusters into proteins at a step after cluster synthesis, and for maturation of Yfh1p, which is a homolog of human frataxin implicated in Friedreich's ataxia HSP70 family chaperone Null mutant is viable, cold-sensitive, osmotically sensitive; ssq1 mutations can suppress the endogenous oxygen toxicity (methionine and lysine auxotrophies) of sod1 null mutants and exhibit diminished rates of respiratory oxygen consumption and reduced mitochondrial aconitase and succinate dehydrogenase activities
YJR045C	[SSC1] Mitochondrial matrix ATPase that is a subunit of the presequence translocase-associated protein import motor (PAM); involved in protein translocation into the matrix and protein folding; member of the heat shock protein 70 (HSP70) family Endo.SceI endonuclease subunit|mitochondrial matrix protein involved in protein import Null mutant is inviable; some alleles demonstrate effects in sporulation and germination
YOR232W	[MGE1] Protein of the mitochondrial matrix involved in protein import into mitochondria; acts as a cochaperone and a nucleotide release factor for Ssc1p; homolog of E. coli GrpE GrpE homolog
YNL328C	[MDJ2] Constituent of the mitochondrial import motor associated with the presequence translocase; function overlaps with that of Pam18p; stimulates the ATPase activity of Ssc1p to drive mitochondrial import; contains a J domain chaperonin Null mutant is viable, mdj1 mdj2 double mutants display severe grwoth defects at high temperature

**YNR017W**	[MAS6] Essential protein of the mitochondrial inner membrane, component of the mitochondrial import system 23 kDa mitochondrial inner membrane protein Null mutant is inviable; conditional mutants accumulate mitochondrial precursor proteins at restrictive temperature
YPL063W	[TIM50] Constituent of the mitochondrial inner membrane presequence translocase (TIM23 complex); may promote binding of incoming precursor proteins to the intermembrane space domain of Tom22p during translocation
YGR033C	[TIM21] Constituent of the mitochondrial inner membrane presequence translocase (TIM23 complex); may regulate protein import by binding to both the translocase of the outer membrane (TOM) and presequence-associated motor (PAM) complexes
YLR008C	[PAM18] Constituent of the mitochondrial import motor associated with the presequence translocase, along with Ssc1p, Tim44p, Mge1p, and Pam16p; stimulates the ATPase activity of Ssc1p to drive mitochondrial import; contains a J domain DnaJ-like protein, cochaperone
YIL022W	[TIM44] Peripheral mitochondrial membrane protein involved in mitochondrial protein import, tethers essential chaperone Ssc1p to the translocon channel at the matrix side of the inner membrane
YJL104W	[PAM16] Constituent of the mitochondrial import motor associated with the presequence translocase, along with Ssc1p, Tim44p, Mge1p, and Pam18p; has similarity to J-domain containing proteins Null mutant is inviable; MIA1 is required for respiration
**YJL143W**	[TIM17] Essential constituent of the mitochondrial inner membrane presequence translocase; interacts with Pam18p to recruit the presequence translocase-associated motor (PAM complex) and also required for protein sorting during import 16.5 kDa inner membrane protein required for import of mitochondrial precursor proteins

0092	GO_TERM:[calcium-dependent protein serine/threonine phosphatase activity] P-Value:5.1e-10 OVERLAP:[Calcineurin B] <100> SIZE:3
YOR324C	[FRT1] Tail-anchored endoplasmic reticulum membrane protein that is a substrate of the phosphatase calcineurin, interacts with homolog Frt2p, promotes cell growth in conditions of high Na+, alkaline pH, and cell wall stress
**YKL190W**	[CNB1] Calcineurin B; the regulatory subunit of calcineurin, a Ca++/calmodulin-regulated protein phosphatase which regulates Crz1p (a stress-response transcription factor), the other calcineurin subunit is encoded by CNA1 and/or CMP1 calcineurin regulatory B subunit|type 2B protein phosphatase Null mutant is viable, Li+ and Na+ sensitive, cnb1 fks1 and cnb1 vma3 double mutants are inviable
**YLR433C**	[CNA1] Calcineurin A; one isoform (the other is CMP2) of the catalytic subunit of calcineurin, a Ca++/calmodulin-regulated protein phosphatase which regulates Crz1p (a stress-response transcription factor), the other calcineurin subunit is CNB1 calcineurin subunit A
**YML057W**	[CMP2] Calcineurin A; one isoform (the other is CNA1) of the catalytic subunit of calcineurin, a Ca++/calmodulin-regulated protein phosphatase which regulates Crz1p (a stress-response transcription factor), the other calcineurin subunit is CNB1 calcineurin subunit A

0093	GO_TERM:[regulation of nitrogen utilization] P-Value:2.6e-04
YNL021W	[HDA1] Putative catalytic subunit of a class II histone deacetylase complex that also contains Hda2p and Hda3p; Hda1p interacts with the Hda2p-Hda3p subcomplex to form an active tetramer; deletion increases histone H2B, H3 and H4 acetylation histone deacetylase|shares sequence similarity with Rpd3p, Hos1p, Hos2p, and Hos3p
YPR179C	[HDA3] Subunit of a possibly tetrameric trichostatin A-sensitive class II histone deacetylase complex that contains an Hda1p homodimer and an Hda2p-Hda3p heterodimer; required for the activity of the complex; has similarity to Hda2p
YJL110C	[GZF3] GATA zinc finger protein and Dal80p homolog that negatively regulates nitrogen catabolic gene expression by competing with Gat1p for GATA site binding; function requires a repressive carbon source; dimerizes with Dal80p and binds to Tor1p GATA zinc finger protein 3 homologous to Dal80 in structure and function Null mutant is partially NCR-insensitive
YKR034W	[DAL80] Negative regulator of genes in multiple nitrogen degradation pathways; expression is regulated by nitrogen levels and by Gln3p; member of the GATA-binding family, forms homodimers and heterodimers with Deh1p GATA family transcriptional repressor Null mutant is viable, deficient in allantoin degradation

0094	GO_TERM:[transcription regulator activity] P-Value:2.1e-03
YIR023W	[DAL81] Positive regulator of genes in multiple nitrogen degradation pathways; contains DNA binding domain but does not appear to bind the dodecanucleotide sequence present in the promoter region of many genes involved in allantoin catabolism transcriptional activator for allantoin and GABA catabolic genes, contains a Zn[2]-Cys[6] fungal-type binuclear cluster domain in the N-terminal region Null mutant is viable, unable to degrade allantoin
YNL314W	[DAL82] Positive regulator of allophanate inducible genes; binds a dodecanucleotide sequence upstream of all genes that are induced by allophanate; contains an UISALL DNA-binding, a transcriptional activation, and a coiled-coil domain positive transcriptional regulator loss of induction for allantoin degradation pathways

0095	GO_TERM:[TORC 1 complex] P-Value:5.0e-08
YHR186C	[KOG1] Subunit of TORC1, a rapamycin-sensitive complex involved in growth control that contains Tor1p or Tor2p, Lst8p and Tco89p; contains four HEAT repeats and seven WD-40 repeats; may act as a scaffold protein to couple TOR and its effectors
YPL180W	[TCO89] Subunit of TORC1 (Tor1p or Tor2p-Kog1p-Lst8p-Tco89p), a complex that regulates growth in response to nutrient availability; cooperates with Ssd1p in the maintenance of cellular integrity; deletion strains are hypersensitive to rapamycin Null: Caffeine Sensitivity.
YJR066W	[TOR1] PIK-related protein kinase and rapamycin target; subunit of TORC1, a complex that controls growth in response to nutrients by regulating translation, transcription, ribosome biogenesis, nutrient transport and autophagy; involved in meiosis phosphatidylinositol kinase homolog Null mutant is viable, grows slowly; rapamycin resistance, tor1 tor2 double mutant is inviable
YER040W	[GLN3] Transcriptional activator of genes regulated by nitrogen catabolite repression (NCR), localization and activity regulated by quality of nitrogen source transcriptional activator of nitrogen-regulated genes
YNL229C	[URE2] Nitrogen catabolite repression regulator that acts by inhibition of GLN3 transcription in good nitrogen source; altered form of Ure2p creates [URE3] prion prion|transcriptional regulator Null mutant is viable but exhibits defects in nitrogen catabolite repression (NCR), and null mutant diploids are defective in pseudohyphal growth and display an increased incidence of random bud patterns.

0096	GO_TERM:[TORC 2 complex] P-Value:6.3e-24
YBR270C	[YBR270C] Hypothetical protein
YPL059W	[GRX5] Hydroperoxide and superoxide-radical responsive glutathione-dependent oxidoreductase; mitochondrial matrix protein involved in the synthesis/assembly of iron-sulfur centers; monothiol glutaredoxin subfamily member along with Grx3p and Grx4p glutaredoxin Null mutant is viable and shows high sensitivity to oxidative stress and increased sensitivity to osmotic stress, and increased oxidation levels of cell proteins; grx5 is synthetically lethal with grx2.
YNL047C	[SLM2] Phosphoinositide PI4,5P(2) binding protein, forms a complex with Slm1p; acts downstream of Mss4p in a pathway regulating actin cytoskeleton organization in response to stress; subunit of and phosphorylated by the TORC2 complex
YIL105C	[SLM1] Phosphoinositide PI4,5P(2) binding protein, forms a complex with Slm2p; acts downstream of Mss4p in a pathway regulating actin cytoskeleton organization in response to stress; subunit of and phosphorylated by the TORC2 complex
YJL058C	[BIT61] Subunit of TORC2 (Tor2p-Lst8p-Avo1-Avo2-Tsc11p-Bit61p-Slm1p-Slm2p), a membrane-associated complex that regulates cell cycle-dependent actin cytoskeletal dynamics during polarized growth and cell wall integrity
YNL006W	[LST8] Protein required for the transport of amino acid permease Gap1p from the Golgi to the cell surface; component of the TOR signaling pathway; associates with both Tor1p and Tor2p; contains a WD-repeat Reduced activity of a broad set of amino acid permeases
YKL203C	[TOR2] PIK-related protein kinase and rapamycin target; subunit of TORC1, a complex that regulates growth in response to nutrients and TORC2, a complex that regulates cell-cycle dependent polarization of the actin cytoskeleton; involved in meiosis Null mutant is inviable, exhibits disruption of the polarized distribution of the actin cytoskeleton during the cell cycle; tor2-1 allele confers rapamycin resistance
YER093C	[TSC11] Subunit of TORC2 (Tor2p-Lst8p-Avo1-Avo2-Tsc11p-Bit61p), a membrane-associated complex that regulates actin cytoskeletal dynamics during polarized growth and cell wall integrity; involved in sphingolipid metabolism; contains a RasGEFN domain
YMR068W	[AVO2] Component of a complex containing the Tor2p kinase and other proteins, which may have a role in regulation of cell growth
YOL078W	[AVO1] Component of a membrane-bound complex containing the Tor2p kinase and other proteins, which may have a role in regulation of cell growth

0097	GO_TERM:[thioredoxin peroxidase activity] P-Value:2.5e-05 OVERLAP:[Cytochrome c oxidase (complex IV)] <420.40> SIZE:11
**Q0250**	[COX2] Subunit II of cytochrome c oxidase, which is the terminal member of the mitochondrial inner membrane electron transport chain; one of three mitochondrially-encoded subunits cytochrome c oxidase subunit II
YBR024W	[SCO2] Protein anchored to the mitochondrial inner membrane, similar to Sco1p and may have a redundant function with Sco1p in delivery of copper to cytochrome c oxidase; interacts with Cox2p
YBR037C	[SCO1] Copper-binding protein of the mitochondrial inner membrane, required for cytochrome c oxidase activity and respiration; may function to deliver copper to cytochrome c oxidase; has similarity to thioredoxins required for accumulation of mitochondrial cytochrome c oxidase subunits I and II

0098	GO_TERM:[extrinsic to membrane] P-Value:1.1e-03
YLR203C	[MSS51] Nuclear encoded protein required for translation of COX1 mRNA; binds to Cox1 protein necessary for cox1 pre-mRNA processing and translation
YML129C	[COX14] Mitochondrial membrane protein, required for assembly of cytochrome c oxidase mitochondrial membrane protein Nuclear respiration deficient, lack cytochromes a and a3 and detectable cytochrome oxidase activity

0099	GO_TERM:[mitochondrial electron transport, cytochrome c to oxygen] P-Value:1.6e-13 OVERLAP:[Cytochrome c oxidase (complex IV)] <420.40> SIZE:11
**Q0045**	[COX1] Subunit I of cytochrome c oxidase, which is the terminal member of the mitochondrial inner membrane electron transport chain; one of three mitochondrially-encoded subunits cytochrome c oxidase subunit I
**Q0275**	[COX3] Subunit III of cytochrome c oxidase, which is the terminal member of the mitochondrial inner membrane electron transport chain; one of three mitochondrially-encoded subunits cytochrome c oxidase subunit III
YER154W	[OXA1] Translocase of the mitochondrial inner membrane, mediates the insertion of both mitochondrial- and nuclear-encoded proteins from the matrix into the inner membrane, interacts with mitochondrial ribosomes; null is respiratory deficient
**YDL067C**	[COX9] Subunit VIIa of cytochrome c oxidase, which is the terminal member of the mitochondrial inner membrane electron transport chain cytochrome c oxidase subunit VIIa Lacks functional cytochrome c oxidase holoenzyme
**YGL187C**	[COX4] Subunit IV of cytochrome c oxidase, which is the terminal member of the mitochondrial inner membrane electron transport chain; N-terminal 25 residues of precursor are cleaved during mitochondrial import cytochrome c oxidase subunit IV
**YNL052W**	[COX5A] Subunit Va of cytochrome c oxidase, which is the terminal member of the mitochondrial inner membrane electron transport chain; predominantly expressed during aerobic growth while its isoform Vb (Cox5Bp) is expressed during anaerobic growth cytochrome c oxidase subunit Va Null mutant is viable, respires at 10-15% of the wild-type rate due to the presence of COX5B; cox5a cox5b double deletion mutants are completely non-respiratory

0100	GO_TERM:[septin ring assembly] P-Value:1.5e-04
YOL103W-A	
YBR042C	
YHR115C	[DMA1] Protein involved in regulating spindle position and orientation, functionally redundant with Dma2p; homolog of S. pombe Dma1 and H. sapiens Chfr
YLR215C	[CDC123] Protein involved in nutritional control of the cell cycle; regulates abundance of the translation initiation factor eIF2; ortholog of human D123 protein
YNL116W	[DMA2] Protein involved in regulating spindle position and orientation, functionally redundant with Dma1p; homolog of S. pombe Dma1 and H. sapiens Chfr

0101	GO_TERM:[glycogen metabolism] P-Value:3.0e-05 OVERLAP:[Pho85p complexes] <133.20> SIZE:6
YER059W	[PCL6] Pho85p cyclin of the Pho80p subfamily; forms the major Glc8p kinase together with Pcl7p and Pho85p; involved in the control of glycogen storage by Pho85p; stabilized by Elongin C binding Null mutant is viable. A Ty insertion mutant exhibits slow growth.
YLR190W	[MMR1] Phosphorylated protein of the mitochondrial outer membrane, localizes only to mitochondria of the bud; interacts with Myo2p to mediate mitochondrial distribution to buds; mRNA is targeted to the bud via the transport system involving She2p
**YPL031C**	[PHO85] Cyclin-dependent kinase, with ten cyclin partners; involved in environmental stress response; in phosphate-rich conditions, Pho85p-Pho80p complex phosphorylates Pho4p which in turn represses PHO5 cyclin-dependent protein kinase
YIL050W	[PCL7] Pho85p cyclin of the Pho80p subfamily, forms a functional kinase complex with Pho85p which phosphorylates Mmr1p and is regulated by Pho81p; involved in glycogen metabolism, expression is cell-cycle regulated cyclin
YJL084C	

0102	GO_TERM:[response to pheromone] P-Value:1.7e-02
YNR047W	
YNL045W	

YOL104C	[NDJ1] Meiosis-specific telomere protein, required for bouquet formation, effective homolog pairing, ordered cross-over distribution (interference), sister chromatid cohesion at meiotic telomeres, and segregation of small chromosomes Null allele exhibits errors in meiotic chromosome segregation about 10-fold higher than the wild-type error rate. Spore viability of homozygous diploids with the null allele is approximately 50% of wild-type. Mutant also shows delayed meiotic chromosome synapsis, disrupted crossover interference and increased frequency of nonexchange chromosomes leading to meiosis I nondisjunction and disruption of distributive disjunction
YCR091W	[KIN82] Putative serine/threonine protein kinase, most similar to cyclic nucleotide-dependent protein kinase subfamily and the protein kinase C subfamily serine/threonine kinase (putative)|similar to cyclic nucleotide-dependent protein kinase subfamily and the protein kinase C subfamily
YML083C	

0103	GO_TERM:[transposition, RNA-mediated] P-Value:1.2e-07 OVERLAP:[Pho85p complexes] <133.20> SIZE:6
YDR034C-C	
YPR137C-A	
YMR316W	[DIA1] Protein of unknown function, involved in invasive and pseudohyphal growth; green fluorescent protein (GFP)-fusion protein localizes to the cytoplasm in a punctate pattern Null mutant is viable and causes invasive growth in haploids and pseudohyphal growth in diploids
YGR027W-A	
YML045W-A	
YKL197C	[PEX1] AAA-family ATPase peroxin required for peroxisome biogenesis, contains two 230 amino acid ATP-binding AAA cassettes, upregulated in anaerobiosis; Pex1p and Pex6p interact via their N-terminal AAA-cassettes AAA ATPase|peroxin
**YNL289W**	[PCL1] Pho85 cyclin of the Pcl1,2-like subfamily, involved in entry into the mitotic cell cycle and regulation of morphogenesis, localizes to sites of polarized cell growth G1 cyclin|associates with PHO85 Required for passage through G(sub)1 in diploid cells lacking CLN1
YPR158W-A	

0104	GO_TERM:[protein kinase activity] P-Value:9.2e-04 OVERLAP:[Casein kinase I] <120.10> SIZE:4
YDR170W-A	
YBR233W	[PBP2] RNA binding protein with similarity to mammalian heterogeneous nuclear RNP K protein, involved in the regulation of telomere position effect and telomere length
YNL054W-A	
YNL001W	[DOM34] Probable RNA-binding protein, functions in protein translation to promote G1 progression and differentiation, required for meiotic cell division
YGR094W	[VAS1] Mitochondrial and cytoplasmic valyl-tRNA synthetase valine-tRNA ligase
YMR295C	
YOR196C	[LIP5] Protein involved in biosynthesis of the coenzyme lipoic acid, has similarity to E. coli lipoic acid synthase lipoic acid synthase Null mutant is viable; cannot synthesize lipoic acid; grows slowly on ethanol-rich media; barely grows on glyerol-rich media; undergoes a high frequency of mitochondrial DNA deletions
YNL022C	
YJL095W	[BCK1] Mitogen-activated protein (MAP) kinase kinase kinase acting in the protein kinase C signaling pathway, which controls cell integrity; upon activation by Pkc1p phosphorylates downstream kinases Mkk1p and Mkk2p MEKK Null mutants are temperature-sensitive and exhibit cell lysis, which can be rescued by 1M sorbitol; null mutants grow very poorly even at the permissive temperature. Some dominant alleles suppress a pkc1 null mutant.
YKL168C	[KKQ8] Putative serine/threonine protein kinase with unknown cellular role
YNL073W	[MSK1] Mitochondrial lysine-tRNA synthetase, required for import of both aminoacylated and deacylated forms of tRNA(Lys) into mitochondria lysine-tRNA ligase An uncharacterized allele is respiratory deficient.
YMR285C	[NGL2] Protein involved in 5.8S rRNA processing; Ccr4p-like RNase required for correct 3'-end formation of 5.8S rRNA at site E; similar to Ngl1p and Ngl3p RNase
YBR086C	[IST2] Plasma membrane protein that may be involved in osmotolerance, localizes to the mother cell in small-budded cells and to the bud in medium- and large-budded cells; mRNA is transported to the bud tip by an actomyosin-driven process
YDL173W	
YBL057C	[PTH2] One of two (see also PTH1) mitochondrially-localized peptidyl-tRNA hydrolases; dispensable for cell growth
YBL010C	

YBL064C	[PRX1] Mitochondrial peroxiredoxin (1-Cys Prx) with thioredoxin peroxidase activity, has a role in reduction of hydroperoxides; induced during respiratory growth and under conditions of oxidative stress peroxiredoxin
YDL201W	[TRM8] Subunit of a tRNA methyltransferase complex composed of Trm8p and Trm82p that catalyzes 7-methylguanosine modification of tRNA
YMR291W	
YPR158C-C	
YDR266C	
YNL192W	[CHS1] Chitin synthase I, requires activation from zymogenic form in order to catalyze the transfer of N-acetylglucosamine (GlcNAc) to chitin; required for repairing the chitin septum during cytokinesis; transcription activated by mating factor chitin synthase 1
YHR082C	[KSP1] Nonessential putative serine/threonine protein kinase of unknown cellular role; overproduction causes allele-specific suppression of the prp20-10 mutation
**YNL154C**	[YCK2] Palmitoylated, plasma membrane-bound casein kinase I isoform; shares redundant functions with Yck1p in morphogenesis, proper septin assembly, endocytic trafficking; provides an essential function overlapping with that of Yck1p casein kinase I homolog Null mutant is viable; yck1 yck2 double deletion mutant is inviable

0105	GO_TERM:[glycogen biosynthesis] P-Value:2.3e-11 OVERLAP:[Synaptonemal complex (SC)] <490> SIZE:5
YJL146W	[IDS2] Protein involved in modulation of Ime2p activity during meiosis, appears to act indirectly to promote Ime2p-mediated late meiotic functions; found in growing cells and degraded during sporulation Null mutations reduce or abolish the ability of IME2p to activate expression of early, middle, and late meiotic genes. Recessive and null ids2 mutants prevent toxicity of Ime2p expression in rad52 haploids, but do not affect Ime2p polypeptide accumulation.
YOR351C	[MEK1] Meiosis-specific serine/threonine protein kinase, functions in meiotic checkpoint, phosphorylates Red1p, interacts with Hop1p; required for meiotic recombination and normal spore viability meiosis-specific serine/threonine protein kinase Null mutant is viable, however diploids homozygous for a mek1 null mutation produce only low percentages of viable spores, reduced spore viability is rescued by spo13 mutations
**YIL072W**	[HOP1] Meiosis-specific DNA binding protein that displays Red1p dependent localization to the unsynapsed axial-lateral elements of the synaptonemal complex; required for homologous chromosome synapsis and chiasma formation DNA binding protein decreased levels of meiotic crossing over and intragenic recombination between markers on homologous chromosomes
**YLR263W**	[RED1] Protein component of the axial elements of the synaptonemal complex, involved in chromosome segregation during the first meiotic division; interacts with Hop1p; required for wild-type levels of Mek1p kinase activity meiosis-specific component of synaptonemal complex axial element protein core
YGL134W	[PCL10] Pho85p cyclin; recruits, activates, and targets Pho85p cyclin-dependent protein kinase to its substrate
YKR058W	[GLG1] Self-glucosylating initiator of glycogen synthesis, also glucosylates n-dodecyl-beta-D-maltoside; similar to mammalian glycogenin glycogen synthesis initiator Null mutant is viable; disruption of both GLG1 and GLG2 renders cells unable to synthesize glycogen
YLR258W	[GSY2] Glycogen synthase, similar to Gsy1p; expression induced by glucose limitation, nitrogen starvation, heat shock, and stationary phase; activity regulated by cAMP-dependent, Snf1p and Pho85p kinases as well as by the Gac1p-Glc7p phosphatase glycogen synthase (UDP-glucose-starch glucosyltransferase) Null mutant is viable. Mutant lacking both GSY1 and GSY2 is viable but lacks glycogen synthase activity and glycogen deposition
YFR015C	[GSY1] Glycogen synthase with similarity to Gsy2p, the more highly expressed yeast homolog; expression induced by glucose limitation, nitrogen starvation, environmental stress, and entry into stationary phase glycogen synthase (UDP-glucose-starch glucosyltransferase) Null mutant is viable. Mutant lacking both GSY1 and GSY2 is viable but lacks glycogen synthase activity and glycogen deposition
YJL137C	[GLG2] Self-glucosylating initiator of glycogen synthesis, also glucosylates n-dodecyl-beta-D-maltoside; similar to mammalian glycogenin glycogen synthesis initiator Null mutant is viable; disruption of both GLG2 and GLG2 renders cells unable to synthesize glycogen

0106	GO_TERM:[alpha,alpha-trehalose-phosphate synthase complex (UDP-forming)] P-Value:8.1e-12
YDR074W	[TPS2] Phosphatase subunit of the trehalose-6-phosphate synthase/phosphatase complex, which synthesizes the storage carbohydrate trehalose; expression is induced by stress conditions and repressed by the Ras-cAMP pathway trehalose-6-phosphate phosphatase Null mutant is viable, exhibits complete loss of trehalose-6-phosphate phosphatase activity, measured in vitro, and accumulation of excessive amounts of trehalose-6-phosphate instead of trehalose upon heat shock or entrance into stationary phase in vivo; null mutant is temperature sensitive, tps2 (pfk3) pfk1 double mutants are glucose negative
YMR261C	[TPS3] Regulatory subunit of trehalose-6-phosphate synthase/phosphatase complex, which synthesizes the storage carbohydrate trehalose; expression is induced by stress conditions and repressed by the Ras-cAMP pathway trehalose-6-phosphate synthase/phosphatase complex 115 kDa regulatory subunit
YBR126C	[TPS1] Synthase subunit of trehalose-6-phosphate synthase/phosphatase complex, which synthesizes the storage carbohydrate trehalose; also found in a monomeric form; expression is induced by the stress response and repressed by the Ras-cAMP pathway trehalose-6-phosphate synthase/phosphatase complex 56 kDa synthase subunit null is viable, but does not grow on glucose and/or fructose,and shows lack of trehalose

YML100W	[TSL1] Large subunit of trehalose 6-phosphate synthase (Tps1p)/phosphatase (Tps2p) complex, which converts uridine-5'-diphosphoglucose and glucose 6-phosphate to trehalose, homologous to Tps3p and may share function similar to TPS3 gene product|trehalose-6-phosphate synthase/phosphatase complex 123 kDa regulatory subunit
YGR276C	[RNH70] 3' exoribonuclease, required for 5S and tRNA-Arg3 maturation ribonuclease H
YBL011W	[SCT1] Glycerol 3-phosphate/dihydroxyacetone phosphate dual substrate-specific sn-1 acyltransferase of the glycerolipid biosynthesis pathway, prefers 16-carbon fatty acids, similar to Gpt2p, gene is constitutively transcribed glycerol 3-phosphate/dihydroxyacetone phosphate dual substrate-specific sn-1 acyltransferase
YFL033C	[RIM15] Glucose-repressible protein kinase involved in signal transduction during cell proliferation in response to nutrients, specifically the establishment of stationary phase; originally identified as a regulator of IME2 trehalose-associated protein kinase related to S. pombe cek1+ Null mutant is viable, demonstrates delayed sporulation, decreased sporulation efficiency, and diminished expression of early meiotic genes; rim4, rim11 and rim15 mutants can't be suppressed by overexpression of IME1
YNL157W	[YNL157W] Hypothetical protein

0107	GO_TERM:[alpha-glucosidase activity] P-Value:2.7e-04 OVERLAP:[Nem1p-Spo7p complex] <295> SIZE:2
YOL016C	[CMK2] Calmodulin-dependent protein kinase; may play a role in stress response, many CA++/calmodulan dependent phosphorylation substrates demonstrated in vitro, amino acid sequence similar to Cmk1p and mammalian Cam Kinase II calmodulin-dependent protein kinase Null mutant is viable, exhibits slow rate of spore germination
YMR165C	[PAH1] Mg²⁺-dependent phosphatidate (PA) phosphatase, catalyzes the dephosphorylation of PA to yield diacylglycerol and P_i, responsible for de novo lipid synthesis; homologous to mammalian lipin 1 phosphatidate phosphohydrolase Null mutant is viable, respiration deficient and show increased stability of heterologous plasmids
**YAL009W**	[SPO7] Integral nuclear/ER membrane protein of unknown function, required for normal nuclear envelope morphology and sporulation Null mutant is viable, sporulation defective
**YHR004C**	[NEM1] Protein of the nuclear envelope required for the spherical shape of the nucleus; required for normal sporulation Null mutant is viable but exhibits slow growth at 37 deg. and 16 deg and has an abnormal nuclear envelope. Homozygous diploid null mutants exhibit defects in sporulation.
YHR182W	
YHR028C	[DAP2] Dipeptidyl aminopeptidase, synthesized as a glycosylated precursor; localizes to the vacuolar membrane; similar to Ste13p dipeptidyl aminopeptidase B (DPAP B)|dipeptidyl aminopeptidase yscV Null mutant is viable and lacks dipeptidyl aminopeptidase yscV activity
YDL179W	[PCL9] Cyclin, forms a functional kinase complex with Pho85p cyclin-dependent kinase (Cdk), expressed in late M/early G1 phase, activated by Swi5p
YBR299W	[MAL32] Maltase (alpha-D-glucosidase), inducible protein involved in maltose catabolism; encoded in the MAL3 complex locus; functional in genomic reference strain S288C maltase Defective maltose fermentation
YGR292W	[MAL12] Maltase (alpha-D-glucosidase), inducible protein involved in maltose catabolism; encoded in the MAL1 complex locus maltase Defective maltose fermentation

0108	GO_TERM:[methionyl glutamyl tRNA synthetase complex] P-Value:5.6e-06
YNR013C	[PHO91] Low-affinity phosphate transporter; deletion of pho84, pho87, pho89, pho90, and pho91 causes synthetic lethality; transcription independent of Pi and Pho4p activity; overexpression results in vigorous growth
YHR131C	
YGL105W	[ARC1] Protein that binds tRNA and methionyl- and glutamyl-tRNA synthetases (Mes1p and Gus1p), delivering tRNA to them, stimulating catalysis, and ensuring their localization to the cytoplasm; also binds quadruplex nucleic acids Null mutant is viable, leads to slow growth and reduced MetRS activity; arc1- mutants are synthetic lethals and are complemented by the genes for methionyl-tRNA and glutamyl-tRNA synthetase.
YGR264C	[MES1] Methionyl-tRNA synthetase, forms a complex with glutamyl-tRNA synthetase (Gus1p) and Arc1p, which increases the catalytic efficiency of both tRNA synthetases; also has a role in nuclear export of tRNAs methionine-tRNA ligase no growth at 36 degrees C

0109	GO_TERM:[alpha,alpha-trehalase activity] P-Value:2.4e-05
YBR001C	[NTH2] Putative neutral trehalase, required for thermotolerance and may mediate resistance to other cellular stresses neutral trehalase Null mutant is viable but lacks thermotolerance
YDR001C	[NTH1] Neutral trehalase, degrades trehalose; required for thermotolerance and may mediate resistance to other cellular stresses; may be phosphorylated by Cdc28p neutral trehalase
YGR267C	[FOL2] GTP-cyclohydrolase I, catalyzes the first step in the folic acid biosynthetic pathway GTP-cyclohydrolase I Folinic acid requiring

YJL141C	[YAK1] Serine-threonine protein kinase that is part of a glucose-sensing system involved in growth control in response to glucose availability; translocates from the cytoplasm to the nucleus and phosphorylates Pop2p in response to a glucose signal viable, confers growth to strains deleted for tpk1, tpk2, tpk3 (genes encoding the catalytic subunit of the cAMP-dependent kinase)
YLR270W	[DCS1] Non-essential hydrolase involved in mRNA decapping, may function in a feedback mechanism to regulate deadenylation, contains pyrophosphatase activity and a HIT (histidine triad) motif; interacts with neutral trehalase Nth1p
YOR173W	[DCS2] Non-essential protein containing a HIT (histidine triad) motif; regulated by Msn2p, Msn4p, and the Ras-cAMP-cAPK signaling pathway, transcript accumulates under glucose limitation, similar to Dcs1p
YPL245W	

0110	GO_TERM:[catalytic activity] P-Value:7.3e-02
YOR386W	[PHR1] DNA photolyase involved in photoreactivation, repairs pyrimidine dimers in the presence of visible light; induced by DNA damage; regulated by transcriptional repressor Rph1p photolyase photoreactivation repair deficient
YPL104W	[MSD1] Mitochondrial aspartyl-tRNA synthetase, required for acylation of aspartyl-tRNA; yeast and bacterial aspartyl-, asparaginyl-, and lysyl-tRNA synthetases contain regions with high sequence similarity, suggesting a common ancestral gene aspartyl-tRNA synthetase Null mutant is viable but shows pleiotropic phenotypes consistent with a lesion in mitochondrial protein synthesis and is unable to acylate mitochondrial aspartyl-tRNA

0111	GO_TERM:[phosphotransferase activity, alcohol group as acceptor] P-Value:7.2e-04
YBR059C	[AKL1] Ser-Thr protein kinase, member (with Ark1p and Prk1p) of the Ark kinase family; involved in endocytosis and actin cytoskeleton organization
YLL018C	[DPS1] Cytoplasmic aspartyl-tRNA synthetase, homodimeric enzyme that catalyzes the specific aspartylation of tRNA(Asp); class II aminoacyl tRNA synthetase; binding to its own mRNA may confer autoregulation aspartyl-tRNA synthetase
YGR038W	[ORM1] Evolutionarily conserved protein with similarity to Orm2p, required for resistance to agents that induce the unfolded protein response; human ortholog is located in the endoplasmic reticulum
YNR039C	[ZRG17] Endoplasmic reticulum protein of unknown function, transcription is induced under conditions of zinc deficiency; mutant phenotype suggests a role in uptake of zinc
YBR157C	[ICS2] Protein of unknown function; null mutation does not confer any obvious defects in growth, spore germination, viability, or carbohydrate utilization
YDL223C	[HBT1] Substrate of the Hub1p ubiquitin-like protein that localizes to the shmoo tip (mating projection); mutants are defective for mating projection formation, thereby implicating Hbt1p in polarized cell morphogenesis
YDR070C	[YDR070C] The authentic, non-tagged protein was localized to the mitochondria
YHR207C	[SET5] Zinc-finger protein of unknown function, contains one canonical and two unusual fingers in unusual arrangements; deletion enhances replication of positive-strand RNA virus
YGR008C	[STF2] Protein involved in regulation of the mitochondrial F1F0-ATP synthase; Stf1p and Stf2p act as stabilizing factors that enhance inhibitory action of the Inh1p protein ATPase stabilizing factor
YGL205W	[POX1] Fatty-acyl coenzyme A oxidase, involved in the fatty acid beta-oxidation pathway; localized to the peroxisomal matrix fatty-acyl coenzyme A oxidase Null mutant is viable, exhibits diminished ability to use oleic acid as a carbon source
YLL038C	[ENT4] Protein of unknown function, contains an N-terminal epsin-like domain
YNL074C	[MLF3] Serine-rich protein of unknown function; overproduction suppresses the growth inhibition caused by exposure to the immunosuppressant leflunomide Null mutant is viable and hypersensitive to leflunomide
YLR195C	[NMT1] N-myristoyl transferase, catalyzes the cotranslational, covalent attachment of myristic acid to the N-terminal glycine residue of several proteins involved in cellular growth and signal transduction N-myristoyl transferase
YER078C	
YBR028C	
YKL062W	[MSN4] Transcriptional activator related to Msn2p; activated in stress conditions, which results in translocation from the cytoplasm to the nucleus; binds DNA at stress response elements of responsive genes, inducing gene expression zinc finger protein Null mutant is viable; msn2 msn4 double deletion mutants exhibit higher sensitivity to different stresses, including carbon source starvation, heat shock and severe osmotic and oxidative stresses

YMR196W	
YJR049C	[UTR1] ATP-NADH kinase; phosphorylates both NAD and NADH; active as a hexamer; enhances the activity of ferric reductase (Fre1p)
YGL059W	
YJR059W	[PTK2] Putative serine/threonine protein kinase involved in regulation of ion transport across plasma membrane; enhances spermine uptake Mutant shows reduced spermine and putrescine uptake and is resistant to toxic polyamine analogs and Li+ and Na+ ions; ptk1 ptk2 double mutant shows virtually abolished high-affinity spermidine transport

0112	GO_TERM:[macromolecule metabolism] P-Value:8.9e-01
YKL171W	
YNL027W	[CRZ1] Transcription factor that activates transcription of genes involved in stress response; nuclear localization is positively regulated by calcineurin-mediated dephosphorylation transcription factor

0113	GO_TERM:[protein serine/threonine kinase activity] P-Value:1.1e-07 OVERLAP:[cAMP-dependent protein kinase] <110> SIZE:4
YKL116C	[PRR1] Protein kinase with a possible role in MAP kinase signaling in the pheromone response pathway protein kinase
YMR184W	[ADD37] Protein of unknown function invovled in ER-associated protein degradtion
YIL107C	[PFK26] 6-phosphofructo-2-kinase, inhibited by phosphoenolpyruvate and sn-glycerol 3-phosphate, has negligible fructose-2,6-bisphosphatase activity, transcriptional regulation involves protein kinase A 6-phosphofructose-2-kinase Null mutant is viable; on pyrvuate medium, no fructose 2,6-P2 is detectable in mutant
YGR249W	[MGA1] Protein similar to heat shock transcription factor; multicopy suppressor of pseudohyphal growth defects of ammonium permease mutants similar to heat shock transcription factor
YKL038W	[RGT1] Glucose-responsive transcription factor that regulates expression of several glucose transporter (HXT) genes in response to glucose; binds to promoters and acts both as a transcriptional activator and repressor transcriptional activator|transcriptional repressor Null mutant is viable, shows consitutive expression of glucose-induced HXT geness
**YIL033C**	[BCY1] Regulatory subunit of the cyclic AMP-dependent protein kinase (PKA), a component of a signaling pathway that controls a variety of cellular processes, including metabolism, cell cycle, stress response, stationary phase, and sporulation cAMP-dependent protein kinase regulatory subunit Null mutant is viable; sra1 mutants are associated with reduction of glycogen accumulation, temperature sensitivity, reduced growth rate on maltose and sucrose, inability to grow on galactose and nonfermentable carbon sources and nitrogen starvation intolerance. Cells lacking Sra1p are constitutive for cAPK activity resulting in meiotic arrest prior to premeiotic DNA synthesis
YNL227C	[JJJ1] Protein that contains a 70 amino acid J-domain, may function as a co-chaperone to recruit Hsp70-like activity to specific sites; mutation causes defects in fluid-phase endocytosis
YMR081C	[ISF1] Serine-rich, hydrophilic protein with similarity to Mbr1p; overexpression suppresses growth defects of hap2, hap3, and hap4 mutants; expression is under glucose control; cotranscribed with NAM7 in a cyp1 mutant Null mutant is viable; overexpression suppresses defects in hap2, hap3, and hap3 mutants; isf1 mbr1 double mutant has synthetic phenotypes
YHR135C	[YCK1] Palmitoylated, plasma membrane-bound casein kinase I isoform; shares redundant functions with Yck2p in morphogenesis, proper septin assembly, endocytic trafficking; provides an essential function overlapping with that of Yck2p casein kinase I homolog Null mutant is viable; yck1 yck2 double deletion mutants are inviable; yck1 point mutants suppress defective Snf1p kinase activity in snf4 strains
YKL093W	[MBR1] Protein involved in mitochondrial functions and stress response; overexpression suppresses growth defects of hap2, hap3, and hap4 mutants Null mutant is viable, shows defective growth on glycerol
YFR017C	
YKL064W	[MNR2] Putative magnesium transporter; has similarity to Alr1p and Alr2p, which mediate influx of Mg2+ and other divalent cations overexpression overcomes manganese toxicity
YFR014C	[CMK1] Calmodulin-dependent protein kinase; may play a role in stress response, many CA++/calmodulan dependent phosphorylation substrates demonstrated in vitro, amino acid sequence similar to Cmk2p and mammalian Cam Kinase II calmodulin-dependent protein kinase
YJR001W	[AVT1] Vacuolar transporter, imports large neutral amino acids into the vacuole; member of a family of seven S. cerevisiae genes (AVT1-7) related to vesicular GABA-glycine transporters Gln (Asn), Ile (Leu), Tyr transporter
**YPL203W**	[TPK2] Subunit of cytoplasmic cAMP-dependent protein kinase, which contains redundant catalytic subunits Tpk1p, Tpk2p, and Tpk3p and regulatory subunit Bcy1p; promotes vegetative growth in response to nutrients; activates filamentous growth cAMP-dependent protein kinase catalytic subunit Null mutant haploids are defective for invasive growth; diploid homozygous null mutants are defective for pseudohyphal growth.

YJL164C	[TPK1] Subunit of cytoplasmic cAMP-dependent protein kinase, which contains redundant catalytic subunits Tpk1p, Tpk2p, and Tpk3p and regulatory subunit Bcy1p; promotes vegetative growth in response to nutrients; inhibits filamentous growth cAMP-dependent protein kinase catalytic subunit (putative) multicopy suppression of ras mutant
YKL166C	[TPK3] Subunit of cytoplasmic cAMP-dependent protein kinase, which contains redundant catalytic subunits Tpk1p, Tpk2p, and Tpk3p and regulatory subunit Bcy1p; promotes vegetative growth in response to nutrients; inhibits filamentous growth cAMP-dependent protein kinase catalytic subunit Null mutant is viable, tpk1 tpk2 tpk3 triple mutant is inviable

0114	GO_TERM:[intrinsic to vacuolar membrane] P-Value:8.1e-07
YER072W	[VTC1] Vacuolar transporter chaperon (VTC) involved in distributing V-ATPase and other membrane proteins; together with other VTC proteins, forms a heterotetrameric complex that associates with the SNARE Nyv1p and the V0 sector of the V-ATPase S. pombe Nrf1p homolog (97% identical in predicted amino acid sequence) Null mutant is viable, but exhibits both reduced V-ATPase in the vacuolar membrane and reduced H(+)-ATPase(Pma1p) in the plasma membrane
YJL012C	[VTC4] Vacuolar membrane protein involved in vacuolar polyphosphate accumulation; functions as a regulator of vacuolar H+-ATPase activity and vacuolar transporter chaperones; involved in non-autophagic vacuolar fusion polyphosphate synthetase (putative) Null mutant has been shown to be either inviable, or viable while exhibiting no polyphosphate accumulation

0115	GO_TERM:[transferase activity] P-Value:5.1e-02
YOR239W	[ABP140] Nonessential protein that binds actin filaments and localizes to actin patches and cables, has similarity to S-adenosylmethionine (AdoMet)-dependent methyltransferases actin filament binding protein
YJL106W	[IME2] Serine/threonine protein kinase involved in activation of meiosis, associates with Ime1p and mediates its stability, activates Ndt80p; IME2 expression is positively regulated by Ime1p Null mutant is viable, homozygous null mutants are sporulation defective. High copy IME2 stimulates meiotic recombination without starvation and permits meiosis in an ime1 null background
YKR100C	[SKG1] Transmembrane protein of unknown function; localizes on the inner surface of the plasma membrane at the bud and in the daughter cell; affects the cell wall polymer composition

0116	GO_TERM:[vacuolar acidification] P-Value:4.6e-35 OVERLAP:[H+-transporting ATPase, vacuolar] <220> SIZE:15
YHR060W	[VMA22] Integral membrane protein that is required for vacuolar H+-ATPase (V-ATPase) function, although not an actual component of the V-ATPase complex; functions in the assembly of the V-ATPase; localized to the yeast endoplasmic reticulum (ER) Null mutant is viable but is defective in vacuolar H(+)-ATPase activity, sensitive to calcium, cyclosporin A, and FK506, and requires calcineurin for viability
YKL119C	[VPH2] Integral membrane protein required for vacuolar H+-ATPase (V-ATPase) function, although not an actual component of the V-ATPase complex; functions in the assembly of the V-ATPase; localized to the endoplasmic reticulum (ER) 25.2 kDa protein involved in assembly of vacuolar H(+) ATPase Null mutant is viable, zinc sensitive, defective in vacuolar acidification, calcium-sensitive, respiratory deficient
YMR027W	
YGR281W	[YOR1] Plasma membrane transporter of the ATP-binding cassette (ABC) family, mediates export of many different organic anions including oligomycin ABC transporter Null mutant is viable but exhibits a slight growth defect; null mutant is hypersensitive to reveromycin A and fumonisin B1. Overexpression increases resistance to fumonisin B, sphingosine, and reveromycin A.
YPL201C	[YIG1] Protein that interacts with glycerol 3-phosphatase and plays a role in anaerobic glycerol production; localizes to the nucleus and cytosol
YLR090W	[XDJ1] Putative chaperone, homolog of E. coli DnaJ, closely related to Ydj1p Null mutant is viable, displays no detectable phenotype
YLR447C	[VMA6] Subunit D of the five-subunit V0 integral membrane domain of vacuolar H+-ATPase (V-ATPase), an electrogenic proton pump found in the endomembrane system; stabilizes VO subunits; required for V1 domain assembly on the vacuolar membrane vacuolar ATPase V0 domain subunit d (36 kDa)|vacuolar H(+) ATPase 36 kDa subunit (D subunit of VO sector) Null mutant is viable, sensitive to media buffered at neutral pH or media containing 100 mM Ca2+
YDR202C	[RAV2] Subunit of RAVE (Rav1p, Rav2p, Skp1p), a complex that associates with the V1 domain of the vacuolar membrane (H+)-ATPase (V-ATPase) and promotes assembly and reassembly of the holoenzyme

YJR033C	[RAV1] Subunit of the RAVE complex (Rav1p, Rav2p, Skp1p), which promotes assembly of the V-ATPase holoenzyme; required for transport between the early and late endosome/PVC and for localization of TGN membrane proteins; potential Cdc28p substrate
**YKL080W**	[VMA5] Subunit C of the eight-subunit V1 peripheral membrane domain of vacuolar H+-ATPase (V-ATPase), an electrogenic proton pump found throughout the endomembrane system; required for the V1 domain to assemble onto the vacuolar membrane V1 sector hydrophilic subunit C|vacuolar ATPase V1 domain subunit C (42 kDa)|vacuolar H-ATPase Null mutant is viable; certain vma5 mutations show allele-specific synthetic lethality with cdc24-ls mutants
**YOR270C**	[VPH1] Subunit of vacuolar-ATPase V0 domain, one of two isoforms (Vph1p and Stv1p); Vph1p is located in V-ATPase complexes of the vacuole while Stv1p is located in V-ATPase complexes of the Golgi and endosomes V0 sector subunit|essential for vacuolar acidification and vacuolar H-ATPase activity|vacuolar ATPase V0 domain subunit a (100 kDa)|vacuolar H-ATPase Null mutant is viable, deficient in assembly of vacuolar H(+) ATPase and acidification of the vacuole
**YBR127C**	[VMA2] Subunit B of the eight-subunit V1 peripheral membrane domain of the vacuolar H+-ATPase (V-ATPase), an electrogenic proton pump found throughout the endomembrane system; contains nucleotide binding sites; also detected in the cytoplasm vacuolar ATPase V1 domain subunit B (60 kDa) Null mutant is viable, severely defective for growth in medium buffered at neutral pH
**YDL185W**	[TFP1] Vacuolar ATPase V1 domain subunit A containing the catalytic nucleotide binding sites; protein precursor undergoes self-catalyzed splicing to yield the extein Tfp1p and the intein Vde (PI-SceI), which is a site-specific endonuclease protein with three regions (ABC) that are spliced to yield the extein AC and the intein B; AC is a 69K vacuolar (H+)-ATPase, and B is a 50K site-specfic endonuclease named VDE (PI-SceI) that is homologous to HO. Cleavage is meiosis-specific and induces ge|site-specific endonuclease VDE (PI-SceI)|vacuolar ATPase V1 domain subunit A (69 kDa) Null mutant is viable, resistant to trifluoperazine, grows slowly under non-acidic conditions and on glycerol and is cold, temperature, and cation-sensitive
**YOR332W**	[VMA4] Subunit E of the eight-subunit V1 peripheral membrane domain of the vacuolar H+-ATPase (V-ATPase), an electrogenic proton pump found throughout the endomembrane system; required for the V1 domain to assemble onto the vacuolar membrane E subunit of V1 sector|vacuolar H(+) ATPase 27 kDa subunit Null mutant is viable, slow growing, cold-sensitive, thermo-sensitive, and exhibits poor growth on glycerol; fails to grow on media supplemented with 100 mM CaCl2 or ZnCl2
**YEL051W**	[VMA8] Subunit D of the eight-subunit V1 peripheral membrane domain of the vacuolar H+-ATPase (V-ATPase), an electrogenic proton pump found throughout the endomembrane system; plays a role in the coupling of proton transport and ATP hydrolysis V1 catalytic sector D subunit|vacuolar H-ATPase Null mutant is viable, does not grow on media buffered at pH 7.5 and does not show accumulation of quinacrine into its vacuoles; grows slowly, fails to grow on non-fermentable carbon sources
**YMR054W**	[STV1] Subunit of vacuolar-ATPase V0 domain, one of two isoforms (Stv1p and Vph1p); Stv1p is located in V-ATPase complexes of the Golgi and endosomes while Vph1p is located in V-ATPase complexes of the vacuole 110 kDa subunit; not in vacuole membrane|vacuolar H-ATPase Null mutant is viable, displays additive phenotypes in combination with vph1 null mutations
**YGR020C**	[VMA7] Subunit F of the eight-subunit V1 peripheral membrane domain of vacuolar H+-ATPase (V-ATPase), an electrogenic proton pump found throughout the endomembrane system; required for the V1 domain to assemble onto the vacuolar membrane vacuolar ATPase V1 domain subunit F (14 kDa) Null mutant is viable, unable to grow on media buffered at pH 7.5, fails to accumulate quinacrine into vacuoles, other subunits of the catalytic sector are not assembled onto the vacuolar membrane
**YHR039C-A**	[VMA10] Vacuolar H+ ATPase subunit G of the catalytic (V1) sector, involved in vacuolar acidification vacuolar ATPase V1 domain subunit G (13 kDa) Null mutant is viable, fails to grow on media buffered at pH 7.5, fails to accumulate quinacrine in its vacuole
**YPR036W**	[VMA13] Subunit H of the eight-subunit V1 peripheral membrane domain of the vacuolar H+-ATPase (V-ATPase), an electrogenic proton pump found throughout the endomembrane system; serves as an activator or a structural stabilizer of the V-ATPase vacuolar H(+) ATPase V1 sector 54 kDa subunit Null mutant is viable, V-ATPase complex from null mutants is less stable than from wild-type strains

0117	GO_TERM:[mitochondrion] P-Value:2.4e-01
YEL030W	[ECM10] Heat shock protein of the Hsp70 family, localized in mitochondrial nucleoids, plays a role in protein translocation, interacts with Mge1p in an ATP-dependent manner; overexpression induces extensive mitochondrial DNA aggregations A Tn3 insertion into this gene causes hypersensitivity to the cell surface polymer perturbing agent calcofluor white.
YJR062C	[NTA1] Amidase, removes the amide group from N-terminal asparagine and glutamine residues to generate proteins with N-terminal aspartate and glutamate residues that are targets of ubiquitin-mediated degradation 52 kDa amidase specific for N-terminal asparagine and glutamine Null mutant is viable but cannot degrade N-end rule substrates that have N-terminal asparagine or glutamine

0118	GO_TERM:[response to stress] P-Value:4.0e-02

YKL210W	[UBA1] Ubiquitin activating enzyme, involved in ubiquitin-mediated protein degradation and essential for viability ubiquitin activating enzyme e1
YMR108W	[ILV2] Acetolactate synthase, catalyses the first common step in isoleucine and valine biosynthesis and is the target of several classes of inhibitors, localizes to the mitochondria; expression of the gene is under general amino acid control acetolactate synthase Isoleucine-plus-valine requiring; Sulfometuron methyl resistance
YEL060C	[PRB1] Vacuolar proteinase B (yscB), a serine protease of the subtilisin family; involved in protein degradation in the vacuole and required for full protein degradation during sporulation vacuolar protease B Null mutant is viable, protease B deficient, has smaller spores than wild-type embedded in a thick matrix
YAL015C	[NTG1] DNA N-glycosylase and apurinic/apyrimidinic (AP) lyase involved in base excision repair, localizes to the nucleus and mitochondrion DNA glycosylase Null mutant is viable but is sensitive to H202 and menadione
YDL059C	[RAD59] Protein involved in the repair of double-strand breaks in DNA during vegetative growth via recombination and single-strand annealing; anneals complementary single-stranded DNA; homologous to Rad52p the RAD59 gene product has homology to the Rad52 protein gamma-ray sensitivity, mitotic recombination defects. rad59 is epistatic to rad52 for its repair and recombination defects.

0119	GO_TERM:[membrane] P-Value:6.6e-02
YFL054C	
YML006C	[GIS4] CAAX box containing protein of unknown function, proposed to be involved in the RAS/cAMP signaling pathway CAAX box containing protein

0120	GO_TERM:[endocytosis] P-Value:3.1e-05
YJL204C	[RCY1] F-box protein involved in recycling plasma membrane proteins internalized by endocytosis; localized to sites of polarized growth Deletion leads to an early block in the endocytic pathway before the intersection with the vacuolar protein sorting pathway
YAL026C	[DRS2] Integral membrane Ca(2+)-ATPase involved in aminophospholipid translocation; required to form a specific class of secretory vesicles that accumulate upon actin cytoskeleton disruption; mutation affects maturation of the 18S rRNA P-type ATPase, potential aminophospholipid translocase Null mutant is viable, cold sensitive with perturbed late Golgi function; drs2 arf1 double mutants are inviable. drs2 dnf1 mutants grow slowly, accumulate intracellular membranes, exhibit substantial defect in transport of alkaline phosphatase to vacuole.
YCR094W	[CDC50] Endosomal protein that regulates cell polarity; similar to Ynr048wp and Lem3p Null mutant is cold-sensitive and sensitive to MMS and HU

0121	GO_TERM:[protein neddylation] P-Value:8.7e-06
YDR139C	[RUB1] Ubiquitin-like protein with similarity to mammalian NEDD8; conjugation (neddylation) substrates include the cullins Cdc53p, Rtt101p, and Cul3p; activated by Ula1p and Uba3p (E1 enzyme pair); conjugation mediated by Ubc12p (E2 enzyme) ubiquitin-like protein Null mutant is viable, with no obvious phenotypes, but is synethic lethal with cdc34(ubc3) ts mutant
YLR128W	[DCN1] Putative Nedd8 ligase; binds Nedd8; involved in cullin neddylation; not essential; similar to C.elegans DCN-1; contains UBA-like ubiquitin-binding domain and a DUF298 domain

0122	GO_TERM:[ubiquitin ligase complex] P-Value:3.5e-26 OVERLAP:[SCF-CDC4 complex] <445.10> SIZE:5
YLR097C	[HRT3] Putative nuclear ubiquitin ligase, based on computational analysis of large-scale protein-protein interaction data; has similarity to F-box proteins; identified in association with Cdc53p, Skp1p and Ubi4 in large-scale studies
YLR289W	[GUF1] Mitochondrial GTPase of unknown function, similar to E. coli elongation factor-type GTP-binding protein LepA and to LK1236.1 from Caenorhabditis elegans GTPase
YPL014W	
YBR026C	[ETR1] 2-enoyl thioester reductase, member of the medium chain dehydrogenase/reductase family; localized to in mitochondria, where it has a probable role in fatty acid synthesis 2-enoyl thioester reductase, E.C. 1.3.1.-
YNL311C	[YNL311C] F-box protein F-box protein
YBR208C	[DUR1,2] Urea amidolyase, contains both urea carboxylase and allophanate hydrolase activities, degrades urea to CO2 and NH3; expression sensitive to nitrogen catabolite repression and induced by allophanate, an intermediate in allantoin degradation urea amidolyase (contains urea carboxylase and allophanate hydrolase) Null mutant is viable; urea degradation deficient

YLR134W	[PDC5] Minor isoform of pyruvate decarboxylase, key enzyme in alcoholic fermentation, decarboxylates pyruvate to acetaldehyde, regulation is glucose- and ethanol-dependent, repressed by thiamine, involved in amino acid catabolism pyruvate decarboxylase undetectable pyruvate decarboxylase activity in pdc1pdc5 double mutants
YGR087C	[PDC6] Minor isoform of pyruvate decarboxylase, key enzyme in alcoholic fermentation, decarboxylates pyruvate to acetaldehyde, regulation is glucose- and ethanol-dependent, involved in amino acid catabolism pyruvate decarboxylase isozyme Null mutant is viable and shows reduced pyruvate decarboxylase activity only in cells grown in ethanol
YLR352W	
YPR164W	[MMS1] Protein likely involved in protection against replication-dependent DNA damage; mutants are sensitive to methyl methanesulfonate (MMS); implicated in regulation of Ty1 transposition insertion at amino acid 166 is dead on 0.005% diepoxybutane
YIL046W	[MET30] F-box protein containing five copies of the WD40 motif, controls cell cycle function, sulfur metabolism, and methionine biosynthesis as part of the ubiquitin ligase complex; interacts with and regulates Met4p, localizes within the nucleus F-box protein
YJR090C	[GRR1] F-box protein component of the SCF ubiquitin-ligase complex, required for Cln1p and Cln2p degradation; involved in carbon catabolite repression, glucose-dependent divalent cation transport, high-affinity glucose transport, and morphogenesis Null mutant is viable, resistant to high levels of divalent cations, sensitive to sulfite, and defective in high affinity glucose transport and glucose repression; null mutant also exibits an elongated cell morphology
YLR368W	[MDM30] F box protein, component of protein ubiquitin ligases; promotes ubiquitin-mediated degradation of Gal4p; required for normal mitochondrial fusion Null: high rate of petite formation
YDR306C	
YJL149W	
YDR131C	
YPL256C	[CLN2] G1 cyclin involved in regulation of the cell cycle; activates Cdc28p kinase to promote the G1 to S phase transition; late G1 specific expression depends on transcription factor complexes, MBF (Swi6p-Mbp1p) and SBF (Swi6p-Swi4p) G1 cyclin Null mutant is viable, exhibits G1 arrest; dominant mutation advances the G(sub)1- to S- phase transition and impairs ability of cells to arrest in G(sub)1 phase in response to external signals
YBR280C	
YLR224W	
**YOR057W**	[SGT1] Probable cochaperone, regulates activity of Cyr1p (adenylyl cyclase); involved in assembly of the kinetochore complex, associates with the SCF (Skp1p/Cdc53p/F box protein) ubiquitin ligase complex
**YFL009W**	[CDC4] F-box protein required for G1/S and G2/M transition, associates with Skp1p and Cdc53p to form a complex, SCFCdc4, which acts as ubiquitin-protein ligase directing ubiquitination of the phosphorylated CDK inhibitor Sic1p ubiquitin ligase subunit Null mutant is inviable. cdc4 mutants arrest in meiosis at the mononucleate stage with duplicated spindle pole bodies.
**YDR054C**	[CDC34] Ubiquitin-conjugating enzyme or E2; together with Skp1p, Rbx1p, Cdc53p, and an F-box protein, forms a ubiquitin-protein ligase called the SCF complex which regulates cell cycle progression by targeting key substrates for degradation ubiquitin-conjugating enzyme overexpression confers resistance to xenobiotics (methylmercury, mercuric chloride, and p-chloromercuribenzoate).
YOL133W	[HRT1] RING finger containing subunit of Skp1-Cullin-F-box ubiquitin protein ligases (SCF); required for Gic2p, Far1p, Sic1p and Cln2p degradation; may tether Cdc34p (a ubiquitin conjugating enzyme or E2) and Cdc53p (a cullin) subunits of SCF Skp1-Cullin-F-box ubiquitin protein ligase (SCF) subunit
**YDL132W**	[CDC53] Cullin, structural protein of SCF complexes (which also contain Skp1p, Cdc34p, and an F-box protein) involved in ubiquitination; SCF promotes the G1-S transition by targeting G1 cyclins and the Cln-CDK inhibitor Sic1p for degradation Cells arrest in G1 with active Cln kinases but no Clb-associated Cdc28p kinase activity
**YDR328C**	[SKP1] Evolutionarily conserved kinetochore protein that is part of multiple protein complexes, including the SCF ubiquitin ligase complex, the CBF3 complex that binds centromeric DNA, and the RAVE complex that regulates assembly of the V-ATPase Null mutant is inviable, temperature-sensitive mutations in SKP1 arrest in G1 or G2

0123	GO_TERM:[Smc5-Smc6 complex] P-Value:7.7e-22
YLR383W	[SMC6] Protein involved in structural maintenance of chromosomes; essential subunit of Mms21-Smc5-Smc6 complex; required for growth, DNA repair, interchromosomal and sister chromatid recombination; homologous to S. pombe rad18
YDR288W	[NSE3] Essential subunit of the Mms21-Smc5-Smc6 complex; protein of unknown function; required for DNA repair and growth
YLR007W	[NSE1] Essential subunit of the Mms21-Smc5-Smc6 complex; nuclear protein required for DNA repair and growth nse1 mutants are highly sensitive to DNA-damaging treatments and exhibit abnormal cellular morphologies.
YML023C	[NSE5] Essential subunit of the Mms21-Smc5-Smc6 complex; required for cell viability and DNA repair non-SMC element of the Smc5-Smc6 complex
YOL034W	[SMC5] Structural maintenance of chromosomes (SMC) protein; essential subunit of the Mms21-Smc5-Smc6 complex; required for growth and DNA repair; S. pombe homolog forms a heterodimer with S. pombe Rad18p that is involved in DNA repair
YDL105W	[NSE4] Nuclear protein that plays a role in the function of the Smc5p-Rhc18p complex

YEL019C	[MMS21] SUMO ligase involved in chromosomal organization and DNA repair; essential subunit of the Mms21-Smc5-Smc6 complex; mutants are sensitive to methyl methanesulfonate and show increased spontaneous mutation and mitotic recombination mms21-1 mutant is sensitive to MMS, X rays and UV and increases the rate of mitotic segregation 23-fold

0124	GO_TERM:[response to stress] P-Value:1.7e-01 OVERLAP:[Kinesin-related motorproteins] <140.30.30.10> SIZE:8
**YHR156C**	[LIN1] Non-essential component of U5 snRNP; nuclear protein; physically interacts with Irr1p of cohesin complex; may link together proteins involved in chromosome segregation, mRNA splicing and DNA replication
YDL013W	[HEX3] Ring finger protein involved in the DNA damage response with possible recombination role; genetically identified by synthetic lethality with SGS1 (DNA helicase) and TOP3 (DNA topoisomerase); sporulation role; interacts with Slx8p and Lin1p null is synthetically lethal with sgs1 null
YOR208W	[PTP2] Phosphotyrosine-specific protein phosphatase involved in the inactivation of mitogen-activated protein kinase (MAPK) during osmolarity sensing; dephosporylates Hog1p MAPK and regulates its localization; localized to the nucleus tyrosine phosphatase Null mutant is viable, grows slowly, is hypersensitive to heat; ptp2 ptc1 mutants exhibit synthetic lethality

0125	GO_TERM:[two-component signal transduction system (phosphorelay)] P-Value:5.4e-09
YMR022W	[QRI8] Ubiquitin conjugating enzyme, involved in the ER-associated protein degradation pathway; requires Cue1p for recruitment to the ER membrane; proposed to be involved in chromatin assembly ubiquitin-conjugating enzyme Overexpression confers resistance to methylmercury.
YLR006C	[SSK1] Cytoplasmic response regulator, part of a two-component signal transducer that mediates osmosensing via a phosphorelay mechanism; dephosphorylated form is degraded by the ubiquitin-proteasome system; potential Cdc28p substrate two-component signal transducer that with Sln1p regulates osmosensing MAP kinase cascade(suppressor of sensor kinase) Null mutant is viable; suppresses the lethality of sln1 or ypd1 disruption mutants
YNR031C	[SSK2] MAP kinase kinase kinase of the HOG1 mitogen-activated signaling pathway; interacts with Ssk1p, leading to autophosphorylation and activation of Ssk2p which phosphorylates Pbs2p; also mediates actin cytoskeleton recovery from osmotic stress MAP kinase kinase kinase|activator of Pbs2p Suppresses sln1 lethality. Synthetically high-osmolarity sensitive when it is combined with both ssk22 and sho1 mutations
YJR074W	[MOG1] Conserved nuclear protein that interacts with GTP-Gsp1p, which is a Ran homolog of the Ras GTPase family, and stimulates nucleotide release, involved in nuclear protein import, nucleotide release is inhibited by Yrb1p nuclear protein that interacts with GTP-Gsp1p Null mutant is viable, temperature sensitive, exhibits defects in nuclear-protein import; MOG1 overexpression supresses the temperature sensitivity of gsp1 strains; overexpression of NTF2 or GSP1 can suppress the ts phenotype of mog1
YHR206W	[SKN7] Nuclear response regulator and transcription factor, part of a branched two-component signaling system; required for optimal induction of heat-shock genes in response to oxidative stress; involved in osmoregulation
YDL235C	[YPD1] Phosphorelay intermediate protein, phosphorylated by the plasma membrane sensor Sln1p in response to osmotic stress and then in turn phosphorylates the response regulators Ssk1p in the cytosol and Skn7p in the nucleus two-component phosphorelay intermediate Null mutant is inviable due to the persistent activation of HOG1 MAP kinase cascade. The ypd1 lethality can be suppressed by overexpression of the tyrosine phosphatase gene PTP2, or by inactivation of either one of SSK1, SSK2, PBS2, or HOG1 genes.
YIL147C	[SLN1] Histidine kinase osmosensor that regulates a MAP kinase cascade; transmembrane protein with an intracellular kinase domain that signals to Ypd1p and Ssk1p, thereby forming a phosphorelay system similar to bacterial two-component regulators histidine kinase osmosensor that regulates an osmosensing MAP kinase cascade|similar to bacterial two-component regulators Null mutant is inviable owing to the constitutive activation of the HOG1 MAPK cascade; mutations in any of the four downstream genes (SSK1, SSK2, PBS2, and HOG1) suppress sln1 lethality

0126	GO_TERM:[condensin complex] P-Value:9.7e-16 OVERLAP:[SPB associated proteins] <480.20> SIZE:14
**YMR065W**	[KAR5] Protein required for nuclear membrane fusion during karyogamy, localizes to the membrane with a soluble portion in the endoplasmic reticulum lumen, may form a complex with Jem1p and Kar2p; expression of the gene is regulated by pheromone coiled-coil membrane protein Null mutant is viable, mating defective, nuclear fusion defective
YFR031C	[SMC2] Component of the condensin complex, essential SMC chromosomal ATPase family member that forms a complex with Smc4p to form the active ATPase; Smc2p/Smc4p complex binds DNA, possibly in the cleft formed by the coiled-coil of the folded dimer SMC chromosomal ATPase family member|similar to ScII (chicken), XCAPE (xenopus), and cut14 (S. pombe) Null mutant is inviable; ts mutant (smc2-6) confers a defect in chromosome segregation and causes partial chromosome decondensation in cells arrested in mitosis
YLR272C	[YCS4] Non-SMC subunit of the condensin complex (Smc2p-Smc4p-Ycs4p-Brn1p-Ycg1p); required for establishment and maintenance of chromosome condensation, chromosome segregation, chromatin binding of condensin and silencing at the mating type locus Null mutant is viable but exhibits defects in sister chromatid separation and segregation.

YLR086W	[SMC4] Subunit of the condensin complex, which reorganizes chromosomes during cell division, forms a stable complex with Smc2p that has ATP-hydrolyzing and DNA-binding activity and promotes knotting of circular DNA; potential Cdc28p substrate SMC chromosomal ATPase family member
YBL097W	[BRN1] Essential protein required for chromosome condensation, likely to function as an intrinsic component of the condensation machinery, may influence multiple aspects of chromosome transmission and dynamics
YDR325W	[YCG1] Non-SMC subunit of the condensin complex (Smc2p-Smc4p-Ycs4p-Brn1p-Ycg1p); required for establishment and maintenance of chromosome condensation, chromosome segregation and for chromatin binding of the condensin complex condensin High-copy suppressor of brn1

0127	GO_TERM:[nucleus] P-Value:6.9e-01
YDR111C	[YDR111C] Putative alanine transaminase (glutamic pyruvic transaminase)
YER147C	[SCC4] Subunit of cohesin loading factor (Scc2p-Scc4p), a complex required for the loading of cohesin complexes onto chromosomes; involved in establishing sister chromatid cohesion during double-strand break repair via phosphorylated histone H2AX

0128	GO_TERM:[nuclear cohesin complex] P-Value:1.1e-17 OVERLAP:[Sister chromatid cohesion complex] <475.05> SIZE:6
YOR195W	[SLK19] Kinetochore-associated protein required for normal segregation of chromosomes in meiosis and mitosis; component of the FEAR regulatory network, which promotes Cdc14p release from the nucleolus during anaphase; potential Cdc28p substrate leucine zipper (putative) Null mutant exibits long astral microtubules, short spindles, bypass meiosis I, partial mitotic arrest; synthetic lethal with kar3*, loss of both produces mitotic arrest
YDL074C	[BRE1] E3 ubiquitin ligase for Rad6p, required for the ubiquitination of histone H2B, recruitment of Rad6p to promoter chromatin and subsequent methylation of histone H3 (on L4 and L79), contains RING finger domain null mutant is sensitive to brefeldin A
YEL043W	
**YPR007C**	[REC8] Meiosis-specific component of sister chromatid cohesion complex; maintains cohesion between sister chromatids during meiosis I; maintains cohesion between centromeres of sister chromatids until meiosis II; homolog of S. pombe Rec8p Null mutant is viable, does not undergo meiotic division and is unable to sporulate. The null mutant also exhibits a loss of sister chromatid cohesion, an absence of the synaptonemal complex, and chaotic chromosome segregation.
**YDR180W**	[SCC2] Subunit of cohesin loading factor (Scc2p-Scc4p), a complex required for the loading of cohesin complexes onto chromosomes; involved in establishing sister chromatid cohesion during DSB repair via histone H2AX
**YJL074C**	[SMC3] Subunit of the multiprotein cohesin complex required for sister chromatid cohesion in mitotic cells; also required, with Rec8p, for cohesion and recombination during meiosis; phylogenetically conserved SMC chromosomal ATPase family member SMC chromosomal ATPase family member
**YFL008W**	[SMC1] Subunit of the multiprotein cohesin complex, essential protein involved in chromosome segregation and in double-strand DNA break repair; SMC chromosomal ATPase family member, binds DNA with a preference for DNA with secondary structure SMC chromosomal ATPase family member null is inviable; other mutants show chromosome loss and defects in nuclear division
**YDL003W**	[MCD1] Essential protein required for sister chromatid cohesion in mitosis and meiosis; subunit of the cohesin complex; expression is cell cycle regulated and peaks in S phase Null mutant is inviable; temperature sensitive mutants are defective in mitotic sister chromatid cohesion and mitotic chromosome condensation; multicopy suppressor of smc1-2 mutation
**YIL026C**	[IRR1] Subunit of the cohesin complex, which is required for sister chromatid cohesion during mitosis and meiosis and interacts with centromeres and chromosome arms, essential for viability cohesin complex subunit Null mutant is inviable; decreased transcription of mutant causes irregularity of zygotes, colonies, decreased adhesion to solid supports

0129	GO_TERM:[sulfur amino acid metabolism] P-Value:6.7e-08 OVERLAP:[Met4/Met28/Met31 complex] <510.190.160.20> SIZE:3
YJR060W	[CBF1] Helix-loop-helix protein that binds the motif CACRTG, which is present at several sites including MET gene promoters and centromere DNA element I (CDEI); required for nucleosome positioning at this motif; targets Isw1p to DNA basic helix-loop-helix protein Null mutant is viable, but grows slowly and causes partial loss of centromere function (increased chromosome loss), benomyl and thiabendazole sensitivity, methionine auxotrophy, and changes in chromatin structure at CENs and some promoters. Null mutation causes precocious sister segregation at MI, and reduced spore viability.
YMR094W	[CTF13] Subunit of the CBF3 complex, which binds to the CDE III element of centromeres, bending the DNA upon binding, and may be involved in sister chromatid cohesion during mitosis
**YIR017C**	[MET28] Transcriptional activator in the Cbf1p-Met4p-Met28p complex, participates in the regulation of sulfur metabolism transcriptional activator in the Cbf1p-Met4p-Met28p complex Null mutant is viable but is a methionine-auxotroph and resistant to toxic analogs of sulfate.

**YNL103W**	[MET4] Lecine-zipper transcriptional activator, responsible for the regulation of the sulfur amino acid pathway, requires different combinations of the auxiliary factors Cbf1p, Met28p, Met31p and Met32p leucine zipper family|transcriptional activator
**YPL038W**	[MET31] Zinc-finger DNA-binding protein, involved in regulating expression of the methionine biosynthetic genes, similar to Met32p highly homologous to Met32p|transcriptional regulator of sulfur amino acid metabolism|zinc finger protein

0130	GO_TERM:[DASH complex] P-Value:4.0e-31 OVERLAP:[Dam1 protein complex] <270.20.30> SIZE:9
**YBR233W-A**	[DAD3] Essential subunit of the DASH microtubule ring complex, couples kinetochores to the force produced by MT depolymerization thereby aiding in chromosome segregation; is transferred to the kinetochore prior to mitosis
**YGR113W**	[DAM1] Essential subunit of the DASH microtubule ring complex, couples kinetochores to the force produced by MT depolymerization thereby aiding in chromosome segregation; Ipl1p target for regulating kinetochore-MT attachments
**YDR201W**	[SPC19] Essential subunit of the DASH microtubule ring complex, couples kinetochores to the force produced by MT depolymerization thereby aiding in chromosome segregation; also localized to nuclear side of spindle pole body spindle pole component
**YKR083C**	[DAD2] Essential subunit of the DASH microtubule ring complex, couples kinetochores to the force produced by MT depolymerization thereby aiding in chromosome segregation; is transferred to the kinetochore prior to mitosis
**YDR320C-A**	[DAD4] Essential subunit of the DASH microtubule ring complex, couples kinetochores to the force produced by MT depolymerization thereby aiding in chromosome segregation; is transferred to the kinetochore prior to mitosis
**YDR016C**	[DAD1] Essential subunit of the DASH microtubule ring complex, couples kinetochores to the force produced by MT depolymerization thereby aiding in chromosome segregation; is transferred to the kinetochore prior to mitosis Null mutant is inviable; temperature-sensitive mutant arrests with large buds and a short mitotic spindle
**YKR037C**	[SPC34] Essential subunit of the DASH microtubule ring complex, couples kinetochores to the force produced by MT depolymerization thereby aiding in chromosome segregation; also localized to nuclear side of spindle pole body spindle pole component
YKL138C-A	[HSK3] Essential subunit of the DASH microtubule ring complex, couples kinetochores to the force produced by MT depolymerization thereby aiding in chromosome segregation; is transferred to the kinetochore prior to mitosis
**YGL061C**	[DUO1] Essential subunit of the DASH microtubule ring complex, couples kinetochores to the force produced by MT depolymerization thereby aiding in chromosome segregation; is transferred to the kinetochore prior to mitosis Null mutant is inviable; overexpression arrests cells at large budded stage
**YKL052C**	[ASK1] Essential subunit of the DASH microtubule ring complex, couples kinetochores to the force produced by MT depolymerization thereby aiding in chromosome segregation; phosphorylated during the cell cycle by cyclin-dependent kinases

0131	GO_TERM:[centromeric DNA binding] P-Value:4.9e-06 OVERLAP:[CBF3 protein complex] <270.10.10> SIZE:4
**YGR140W**	[CBF2] Essential kinetochore protein, component of the CBF3 multisubunit complex that binds to the CDEIII region of the centromere; Cbf2p also binds to the CDEII region possibly forming a different multimeric complex, ubiquitinated in vivo centromere binding factor CBF3 110 kDa subunit
**YMR168C**	[CEP3] Essential kinetochore protein, component of the CBF3 complex that binds the CDEIII region of the centromere; contains an N-terminal Zn2Cys6 type zinc finger domain, a C-terminal acidic domain, and a putative coiled coil dimerization domain Cbf3 kinetochore protein complex subunit b Null mutant is inviable; mutations within the zinc finger domain result in cells that exhibit a G2-M cell cycle delay and increased chromosome loss in each mitotic cell division; at nonpermissive temperature the cep3 cells arrest with an undivided nucleus and a short mitotic spindle; at permissive temperature cep3 cells are unable to support segregation of minichromosomes with mutations in the central part of element III of yeast centromere DNA

0132	GO_TERM:[microtubule motor activity] P-Value:8.3e-06 OVERLAP:[Kinesin-related motorproteins] <140.30.30.10> SIZE:8
**YMR198W**	[CIK1] Kinesin-associated protein required for both karyogamy and mitotic spindle organization, interacts stably and specifically with Kar3p and may function to target this kinesin to a specific cellular role; has similarity to Vik1p Kar3-binding protein Null mutant is viable but is defective in both karyogamy and chromosome maintenance and does not show proper localization of Kar3p to microtubule-associated structures
YGR089W	[NNF2] Protein that exhibits physical and genetic interactions with Rpb8p, which is a subunit of RNA polymerases I, II, and III; computational analysis of large-scale protein-protein interaction data suggests a role in chromosome segregation

**YPR141C**	[KAR3] Minus-end-directed microtubule motor that functions in mitosis and meiosis, localizes to the spindle pole body and localization is dependent on functional Cik1p, required for nuclear fusion during mating; potential Cdc28p substrate kinesin-like nuclear fusion protein Null mutant is viable. Mutations in KAR3 are semidominant and cause pleiotropic effects affecting both mitosis and meiosis. kar3 mutations prevent karyogamy (nuclear fusion).

0133	GO_TERM:[response to stress] P-Value:4.0e-02 OVERLAP:[MRE11/RAD50/XRS2 complex] <510.180.30.30> SIZE:3
YCR073C	[SSK22] MAP kinase kinase kinase of the HOG1 mitogen-activated signaling pathway; functionally redundant with, and homologous to, Ssk2p; interacts with and is activated by Ssk1p; phosphorylates Pbs2p
**YNL250W**	[RAD50] Subunit of MRX complex, with Mre11p and Xrs2p, involved in processing double-strand DNA breaks in vegetative cells, initiation of meiotic DSBs, telomere maintenance, and nonhomologous end joining Mre11-Rad50-Xrs2 protein complex member involved in joining double-stranded breaks and DNA recombination Null mutant is viable but defective for X-ray damage repair, sporulation, chromosome pairing, formation and processing of DS breaks, gene conversion and reciprocal recombination in non-rDNA, tripartite synaptonemal complexes and heteroduplex DNA. Exhibits blocked meiotic recombination and formation of synaptonemal complex at early stages. rad50-1 or null is rescued by spo13 and rescues rad52 spo13.

0134	GO_TERM:[organelle organization and biogenesis] P-Value:1.2e-01
YDR150W	[NUM1] Protein required for nuclear migration, localizes to the mother cell cortex and the bud tip; may mediate interactions of dynein and cytoplasmic microtubules with the cell cortex Null mutant is viable; num1-disrupted strains contain many budded cells with two nuclei in mother cell, and haploid num1 strains tend to diploidize during mitosis
YDR295C	[HDA2] Subunit of a possibly tetrameric trichostatin A-sensitive class II histone deacetylase complex containing an Hda1p homodimer and an Hda2p-Hda3p heterodimer; involved in telomere maintenance

0135	GO_TERM:[kinetochore] P-Value:2.5e-44 OVERLAP:[Ndc80 protein complex] <270.20.40> SIZE:4
**YOL069W**	[NUF2] Component of the evolutionarily conserved kinetochore-associated Ndc80 complex (Ndc80p-Nuf2p-Spc24p-Spc25p); involved in chromosome segregation, spindle checkpoint activity and kinetochore clustering Null mutant is inviable; temperature-sensitive mutants arrest with single undivided or partially divided nucleus in the bud neck, shortened mitotic spindle, and fully replicated DNA
**YIL144W**	[TID3] Component of the evolutionarily conserved kinetochore-associated Ndc80 complex (Ndc80p-Nuf2p-Spc24p-Spc25p); conserved coiled-coil protein involved in chromosome segregation, spindle checkpoint activity, kinetochore assembly and clustering
**YER018C**	[SPC25] Component of the evolutionarily conserved kinetochore-associated Ndc80 complex (Ndc80p-Nuf2p-Spc24p-Spc25p); involved in chromosome segregation, spindle checkpoint activity and kinetochore clustering spindle pole component
**YMR117C**	[SPC24] Component of the evolutionarily conserved kinetochore-associated Ndc80 complex (Ndc80p-Nuf2p-Spc24p-Spc25p); involved in chromosome segregation, spindle checkpoint activity and kinetochore clustering spindle pole component
YGL093W	[SPC105] Protein required for accurate chromosome segregation, localizes to the nuclear side of the spindle pole body; forms a complex with Ydr532cp spindle pole component
YJR112W	[NNF1] Essential component of the MIND kinetochore complex (Mtw1p Including Nnf1p-Nsl1p-Dsn1p) which joins kinetochore subunits contacting DNA to those contacting microtubules; required for accurate chromosome segregation Null mutant is inviable; cells depleted of Nnf1p or containing a temperature-sensitive nnf1 mutation have elongated microtubules and become bi- and multinucleate
YKL089W	[MIF2] Kinetochore protein with homology to human CENP-C, required for structural integrity of the spindle during anaphase spindle elongation, interacts with histones H2A, H2B, and H4, phosphorylated by Ipl1p Null mutant is inviable, temperature sensitive mutants accumulate large budded cells and broken spindles at the restricitive temperature
YKL049C	[CSE4] Centromere protein that resembles histones, required for proper kinetochore function; homolog of human CENP-A similar to histone H3 and to human centromere protein CENP-A Null mutant is inviable; cse4-1 mutant causes increased non-disjunction of chromosome with mutated CEN and t.s. arrest at G2/M boundary with 2N DNA content
YAL034W-A	[MTW1] Essential component of the MIND kinetochore complex (Mtw1p Including Nnf1p-Nsl1p-Dsn1p) which joins kinetochore subunits contacting DNA to those contacting microtubules; critical to kinetochore assembly Null mutant is inviable. ts mtw1 mutant exhibits longer metaphase spindles and unequal sister chromatid segregation
YLR315W	[NKP2] Non-essential kinetochore protein, subunit of the Ctf19 central kinetochore complex (Ctf19p-Mcm21p-Okp1p-Mcm22p-Mcm16p-Ctf3p-Chl4p-Mcm19p-Nkp1p-Nkp2p-Ame1p-Mtw1p)
YDR383C	[NKP1] Non-essential kinetochore protein, subunit of the Ctf19 central kinetochore complex (Ctf19p-Mcm21p-Okp1p-Mcm22p-Mcm16p-Ctf3p-Chl4p-Mcm19p-Nkp1p-Nkp2p-Ame1p-Mtw1p)

YPL233W	[NSL1] Essential component of the MIND kinetochore complex (Mtw1p Including Nnf1p-Nsl1p-Dsn1p) which joins kinetochore subunits contacting DNA to those contacting microtubules; required for accurate chromosome segregation Protein required for cell viability
YIR010W	[DSN1] Essential component of the MIND kinetochore complex (Mtw1p Including Nnf1p-Nsl1p-Dsn1p) which joins kinetochore subunits contacting DNA to those contacting microtubules; important for chromosome segregation Protein required for cell viability
YDR318W	[MCM21] Protein involved in minichromosome maintenance; component of the COMA complex (Ctf19p, Okp1p, Mcm21p, Ame1p) that bridges kinetochore subunits that are in contact with centromeric DNA and the subunits bound to microtubules Null mutant is viable but exhibits defects in the stability of minichromosomes. Mutants also exhibit elevated rates of chromosome loss (but not those of recombination) and are hypersensitive to the anti-mitotic drug benomyl.
YPR046W	[MCM16] Protein involved in kinetochore-microtubule mediated chromosome segregation; binds to centromere DNA Null mutant is viable, exhibits increased sensitivity to the anitmitotic drugs benomyl and thiabenzadole; exhibits a high rate of chromosome III loss without a significant increase in recombination frequency, may exhibit altered kinetochore assembly
YLR381W	[CTF3] Outer kinetochore protein that forms a complex with Mcm16p and Mcm22p; may bind the kinetochore to spindle microtubules
YBR107C	[IML3] Protein with a role in kinetochore function, localizes to the outer kinetochore in a Ctf19p-dependent manner, interacts with Chl4p and Ctf19p Null mutant is viable, but exhibits chromosome loss and abnormal chromosomal segregation
YJR135C	[MCM22] Protein involved in minichromosome maintenance; component of the kinetochore; binds to centromeric DNA in a Ctf19p-dependent manner
YBR211C	[AME1] Essential kinetochore protein associated with microtubules and spindle pole bodies; component of the kinetochore sub-complex COMA (Ctf19p, Okp1p, Mcm21p, Ame1p); involved in spindle checkpoint maintenance microtubule stability regulator Null: Null mutant is inviable; localizes to microtubules and SPB region, ame1-1 arrests in G2/M, mutant rescues benomyl sensitivity of TUB4/ tub4 heterozygote, ame1-4 mutant allele and heterozygous mutant confer benomyl resistance, interacts with APC lid protein by two-hybrid
YDR254W	[CHL4] Outer kinetochore protein required for chromosome stability, interacts with kinetochore proteins Ctf19p, Ctf3p, and Iml3p; exhibits a two-hybrid interaction with Mif2p; association with CEN DNA requires Ctf19p Null mutant is viable. Some authors report a temperature-senstive deletion allele, while others fine no temperature sensitivity in another deletion allele. Fidelity of chromosome transmission and minichromosome nondisjunction in mitosis is decreased at all temperatures.
YGR179C	[OKP1] Outer kinetochore protein, required for accurate mitotic chromosome segregation; component of the kinetochore sub-complex COMA (Ctf19p, Okp1p, Mcm21p, Ame1p) that functions as a platform for kinetochore assembly
YPL018W	[CTF19] Outer kinetochore protein, required for accurate mitotic chromosome segregation; component of the kinetochore sub-complex COMA (Ctf19p, Okp1p, Mcm21p, Ame1p) that functions as a platform for kinetochore assembly kinetochore protein

0136	GO_TERM:[microtubule cytoskeleton] P-Value:1.2e-07 OVERLAP:[Tubulin-associated proteins] <140.30.20> SIZE:14
YLR210W	[CLB4] B-type cyclin involved in cell cycle progression; activates Cdc28p to promote the G2/M transition; may be involved in DNA replication and spindle assembly; accumulates during S phase and G2, then targeted for ubiquitin-mediated degradation B-type cyclin
YPL155C	[KIP2] Kinesin-related motor protein involved in mitotic spindle positioning, stabilizes microtubules by targeting Bik1p to the plus end; Kip2p levels are controlled during the cell cycle kinesin related protein
**YLR045C**	[STU2] Microtubule-associated protein (MAP) of the XMAP215/Dis1 family; regulates microtubule dynamics during spindle orientation and metaphase chromosome alignment; interacts with spindle pole body component Spc72p
YPL269W	[KAR9] Karyogamy protein required for correct positioning of the mitotic spindle and for orienting cytoplasmic microtubules, localizes at the shmoo tip in mating cells and at the tip of the growing bud in small-budded cells through anaphase Null mutant is viable; cytoplasmic microtubule orientation defects, nuclear migration defects, benomyl sensitive
**YCL029C**	[BIK1] Microtubule-associated protein, component of the interface between microtubules and kinetochore, involved in sister chromatid separation; essential in polyploid cells but not in haploid or diploid cells; ortholog of mammalian CLIP-170 Null mutant is viable, bik1 mutants exhibit bilateral defects in karyogamy
**YER016W**	[BIM1] Microtubule-binding protein that together with Kar9p makes up the cortical microtubule capture site and delays the exit from mitosis when the spindle is oriented abnormally Null mutant is viable, causes cold sensitivity, benomyl supersensitivity, aberrant microtubule morphology. During mitosis in bim1 mutants, the nucleus fails to move to the mother-bud neck.

0137	GO_TERM:[transposition, RNA-mediated] P-Value:8.6e-04
YJL113W	
YJL114W	

0138	GO_TERM:[prefoldin complex] P-Value:6.9e-17 OVERLAP:[Gim complexes] <177> SIZE:5
YNL188W	[KAR1] Essential protein involved in karyogamy during mating and in spindle pole body duplication during mitosis, localizes to the half-bridge of the spindle pole body, interacts with Spc72p during karyogamy, also interacts with Cdc31p Null mutant is inviable, kar1 mutants are karyogamy defective; defects in KAR1 block spindle pole body duplication; the temperature sensitivity of a kar1 mutant defective for localization to the spindle pole body can be suppressed by CDC31 overexpression or by dominant-acting CDC31 alleles
YAL047C	[SPC72] Component of the cytoplasmic Tub4p (gamma-tubulin) complex, binds spindle pole bodies and links them to microtubules; has roles in astral microtubule formation and stabilization Null mutant is inviable. Cells lacking Spc72 can only generate very short (<1 micron) and unstable astral microtubules. Consequently, nuclear migration to the bud neck and orientation of the anaphase spindle along the mother-bud axis are absent in these cells.
YHR172W	[SPC97] Component of the microtubule-nucleating Tub4p (gamma-tubulin) complex; interacts with Spc110p at the spindle pole body (SPB) inner plaque and with Spc72p at the SPB outer plaque gamma-tubulin complex component Null mutant is inviable; required for microtubule organization and spindle pole body duplication
YNL126W	[SPC98] Component of the microtubule-nucleating Tub4p (gamma-tubulin) complex; interacts with Spc110p at the spindle pole body (SPB) inner plaque and with Spc72p at the SPB outer plaque gamma-tubulin complex component Null mutant is inviable; overexpression is toxic resulting in accumulation of cells with large buds, 2N DNA content, defect in microtubule structure. ts-phenotype: arrest in G2 of cell cycle with large bud, duplicated spindle pole bodies, short spindle and elongated cytoplasmic microtubules
YLR212C	[TUB4] Gamma-tubulin, involved in nucleating microtubules from both the cytoplasmic and nuclear faces of the spindle pole body gamma tubulin Null mutant is inviable. Tub4p-depleted cells arrest during nuclear division; most arrested cells contain a large bud, replicated DNA, and a single nucleus. Immunofluorescence and nuclear staining experiments indicate that cells depleted of Tub4p contain defects in the organization of both cytoplasmic and nuclear microtubule arrays; such cells exhibit nuclear migration failure, defects in spindle formation, and/or aberrantly long cytoplasmic microtubule arrays.
**YLR200W**	[YKE2] Subunit of the heterohexameric Gim/prefoldin protein complex involved in the folding of alpha-tubulin, beta-tubulin, and actin bovine NABC complex component homolog|non-native actin binding complex polypeptide 6|prefoldin complex subunit
YJL179W	[PFD1] Subunit of heterohexameric prefoldin, which binds cytosolic chaperonin and transfers target proteins to it; involved in the biogenesis of actin and of alpha- and gamma-tubulin bovine prefoldin subunit 1 homolog (putative)
**YEL003W**	[GIM4] Subunit of the heterohexameric cochaperone prefoldin complex which binds specifically to cytosolic chaperonin and transfers target proteins to it bovine prefoldin subunit 2 homolog (putative) Null mutant is viable, sensitive to anti-microtubule drugs benomyl and nocadazole; synthetically lethal with tub4-1 mutations
**YNL153C**	[GIM3] Subunit of the heterohexameric cochaperone prefoldin complex which binds specifically to cytosolic chaperonin and transfers target proteins to it bovine prefoldin subunit 4 homolog (putative)
**YGR078C**	[PAC10] Part of the heteromeric co-chaperone GimC/prefoldin complex, which promotes efficient protein folding bovine NABC complex component homolog|non-native actin binding complex polypeptide 3|prefoldin complex subunit Null mutant is viable, benomyl sensitive, cold sensitive, microtubules disassemble at 14 degrees celsius, pac10 mutants exhibit synthetic lethality with tub4-1, cin8, cin1, pac2 and rbl2 mutants
**YML094W**	[GIM5] Subunit of the heterohexameric cochaperone prefoldin complex which binds specifically to cytosolic chaperonin and transfers target proteins to it bovine prefoldin subunit 5 homolog (putative) Null mutant is viable, cold sensitive, benomyl and nocadazole sensitive and fails to grow on YPD+1.2M KCl or YPD+1.8M sorbitol ; synthetically lethal with tub4-1 mutations

0139	GO_TERM:[spindle] P-Value:4.5e-15
YIL149C	[MLP2] Myosin-like protein associated with the nuclear envelope, connects the nuclear pore complex with the nuclear interior; involved in the Tel1p pathway that controls telomere length coiled-coil protein (putative), similar to myosin and TPR
YKR095W	[MLP1] Myosin-like protein associated with the nuclear envelope, connects the nuclear pore complex with the nuclear interior; involved with Tel1p in telomere length control; involved with Pml1p and Pml39p in nuclear retention of unspliced mRNAs colied-coil protein (putative), similar to myosin and TPR
YDR356W	[SPC110] Inner plaque spindle pole body (SPB) component, ortholog of human kendrin; involved in connecting nuclear microtubules to SPB; interacts with Tub4p-complex and calmodulin; phosphorylated by Mps1p in cell cycle-dependent manner interacts with Spc42p, calmodulin, and a 35 kDa protein|spindle pole body component
YPL124W	[SPC29] Inner plaque spindle pole body (SPB) component, links the central plaque component Spc42p to the inner plaque component Spc110p; required for SPB duplication
YOR177C	[MPC54] Component of the meiotic outer plaque, a membrane-organizing center which is assembled on the cytoplasmic face of the spindle pole body during meiosis II and triggers the formation of the prospore membrane; potential Cdc28p substrate Null: viable. Other phenotypes: sporulation deficient.
YKL042W	[SPC42] Central plaque component of spindle pole body (SPB); involved in SPB duplication, may facilitate attachment of the SPB to the nuclear membrane spindle pole body component Null mutant is inviable; temperature sensitive mutations show SBP duplication
YLR227C	[ADY4] Structural component of the meiotic outer plaque, which is a membrane-organizing center that assembles on the cytoplasmic face of the spindle pole body during meiosis II and triggers the formation of the prospore membrane

YDL239C	[ADY3] Protein required for spore wall formation, thought to mediate assembly of a Don1p-containing structure at the leading edge of the prospore membrane via interaction with spindle pole body components; potentially phosphorylated by Cdc28p Null forms largely asci that contain 2 spores (dyads) when sporulated. Sporulation defect in ady3ady3 cells is due to a failure to synthesize spore wall polymers.
YNL225C	[CNM67] Component of the spindle pole body outer plaque; required for spindle orientation and mitotic nuclear migration Null mutant is viable but shows slow growth and a nuclear migration defect
YOL091W	[SPO21] Component of the meiotic outer plaque of the spindle pole body, involved in modifying the meiotic outer plaque that is required prior to prospore membrane formation meiosis proficient, fails to form spores
YOR373W	[NUD1] Component of the spindle pole body outer plaque, required for exit from mitosis

0140	GO_TERM:[regulation of mitosis] P-Value:1.7e-11
YGL229C	[SAP4] Protein required for function of the Sit4p protein phosphatase, member of a family of similar proteins that form complexes with Sit4p, including Sap155p, Sap185p, and Sap190p
YJL013C	[MAD3] Component of the spindle-assembly checkpoint complex, which delays the onset of anaphase in cells with defects in mitotic spindle assembly; interacts physically with the spindle checkpoint proteins Bub3p and Mad2p spindle checkpoint complex subunit Null mutant is viable, benomyl/nocodazole sensitive
YJL030W	[MAD2] Component of the spindle-assembly checkpoint complex, which delays the onset of anaphase in cells with defects in mitotic spindle assembly; forms a complex with Mad1p spindle checkpoint complex subunit
YGL116W	[CDC20] Cell-cycle regulated activator of anaphase-promoting complex/cyclosome (APC/C), which is required for metaphase/anaphase transition; directs ubiquitination of mitotic cyclins, Pds1p, and other anaphase inhibitors; potential Cdc28p substrate anaphase promoting complex (APC) subunit Null mutant is inviable; conditional alleles show cell cycle arrest in G2
YGR188C	[BUB1] Protein kinase that forms a complex with Mad1p and Bub3p that is crucial in the checkpoint mechanism required to prevent cell cycle progression into anaphase in the presence of spindle damage, associates with centromere DNA via Skp1p Mutants are unable to recover from transient loss of spindle function. Overexpression of BUB1 rescues the cold sensitivity of tub1-729.
YGL086W	[MAD1] Coiled-coil protein involved in the spindle-assembly checkpoint; phosphorylated by Mps1p upon checkpoint activation which leads to inhibition of the activity of the anaphase promoting complex; forms a complex with Mad2p
YOR026W	[BUB3] Kinetochore checkpoint WD40 repeat protein that localizes to kinetochores during prophase and metaphase, delays anaphase in the presence of unattached kinetochores; forms complexes with Mad1p-Bub1p and with Cdc20p, binds Mad2p and Mad3p

0141	GO_TERM:[mitotic metaphase/anaphase transition] P-Value:1.2e-39 OVERLAP:[Anaphase promoting complex (APC)] <60> SIZE:11
YMR001C	[CDC5] Polo-like kinase with similarity to Xenopus Plx1 and S. pombe Plo1p; found at bud neck, nucleus and SPBs; has multiple functions in mitosis and cytokinesis through phosphorylation of substrates; may be a Cdc28p substrate protein kinase Null mutant is inviable. cdc5(ts) mutants form synaptonemal complexes lacking central elements and arrest either at meiosis I with broken spindles or at meiosis II with short spindles. Late shifts to a restrictive temperature result in reductional dyads; each spore contains an entire meiosis II short spindle with unseparated chromatids. In some strains at semi-permissive temperature, chromosomes segregate reductionally or equationally depending upon the centromere.
YGL003C	[CDH1] Cell-cycle regulated activator of the anaphase-promoting complex/cyclosome (APC/C), which directs ubiquitination of mitotic cyclins resulting in exit from mitosis; targets the APC/C to specific substrates including CDC20, ASE1 and CIN8 required for Clb2 and Ase1 degradation Null mutant is viable but defective in Clb2p and Ase1p degradation; deletion of cdh1 causes pheromone resistance and is synthetically lethal with sic1 deletion; overexpression causes ectopic degradation of Clb2p and Ase1p
**YFR036W**	[CDC26] Subunit of the Anaphase-Promoting Complex/Cyclosome (APC/C), which is a ubiquitin-protein ligase required for degradation of anaphase inhibitors, including mitotic cyclins, during the metaphase/anaphase transition thermosensitive cell growth (lethal at high temperature)
**YNL172W**	[APC1] Largest subunit of the Anaphase-Promoting Complex/Cyclosome (APC/C), which is a ubiquitin-protein ligase required for degradation of anaphase inhibitors, including mitotic cyclins, during the metaphase/anaphase transition ubiquitin ligase subunit
**YBL084C**	[CDC27] Subunit of the Anaphase-Promoting Complex/Cyclosome (APC/C), which is a ubiquitin-protein ligase required for degradation of anaphase inhibitors, including mitotic cyclins, during the metaphase/anaphase transition anaphase promoting complex (APC) subunit Null mutant is inviable. Some conditional alleles overreplicate their DNA.
**YHR166C**	[CDC23] Subunit of the anaphase-promoting complex/cyclosome (APC/C), which is a ubiquitin-protein ligase required for degradation of anaphase inhibitors, including mitotic cyclins, during the metaphase/anaphase transition unable to complete G(sub)2/M transition

YKL022C	[CDC16] Subunit of the anaphase-promoting complex/cyclosome (APC/C), which is a ubiquitin-protein ligase required for degradation of anaphase inhibitors, including mitotic cyclins, during the metaphase/anaphase transition; required for sporulation metal-binding nucleic acid-binding protein, interacts with Cdc23p and Cdc27p to catalyze the conjugation of ubiquitin to cyclin B (putative) Null mutant is inviable; sensitive to caffeine; cdc16 mutants are unable to progress through the G(sub)2/M transition, cell division cycle blocked at 36 degrees C
YLR102C	[APC9] Subunit of the Anaphase-Promoting Complex/Cyclosome (APC/C), which is a ubiquitin-protein ligase required for degradation of anaphase inhibitors, including mitotic cyclins, during the metaphase/anaphase transition anaphase promoting complex (APC) subunit Null mutant is viable at 37 C but show delay in entry into anaphase at 37 C
YOR249C	[APC5] Subunit of the Anaphase-Promoting Complex/Cyclosome (APC/C), which is a ubiquitin-protein ligase required for degradation of anaphase inhibitors, including mitotic cyclins, during the metaphase/anaphase transition anaphase promoting complex (APC) subunit Null mutant is inviable at 25 C
YDL008W	[APC11] Catalytic core subunit of the Anaphase-Promoting Complex/Cyclosome (APC/C), which is a ubiquitin-protein ligase required for degradation of anaphase inhibitors, including mitotic cyclins, during the metaphase/anaphase transition anaphase promoting complex (APC) subunit Null mutant is inviable at 25 C
YLR127C	[APC2] Subunit of the Anaphase-Promoting Complex/Cyclosome (APC/C), which is a ubiquitin-protein ligase required for degradation of anaphase inhibitors, including mitotic cyclins, during the metaphase/anaphase transition; similar to cullin Cdc53p anaphase promoting complex (APC) subunit Null mutant is inviable at 25 deg. C; ts mutants arrest in metaphase due to defect in the degradation of Pds1; extracts from G1-arrested apc2 mutants are defective in the ubiquitination of mitotic cyclins
YDR118W	[APC4] Subunit of the Anaphase-Promoting Complex/Cyclosome (APC/C), which is a ubiquitin-protein ligase required for degradation of anaphase inhibitors, including mitotic cyclins, during the metaphase/anaphase transition anaphase promoting complex (APC) subunit Null mutant is inviable at 25 C
YGL240W	[DOC1] Processivity factor required for the ubiquitination activity of the anaphase promoting complex (APC), mediates the activity of the APC by contributing to substrate recognition; involved in cyclin proteolysis
YDR260C	[SWM1] Subunit of the anaphase-promoting complex, which is an E3 ubiquitin ligase that regulates the metaphase-anaphase transition and exit from mitosis; required for activation of the daughter-specific gene expression and spore wall maturation Null mutant completes meiotic nuclear division but does not show spore wall maturation
YIR025W	[MND2] Subunit of the anaphase-promoting complex (APC); needed for meiotic nuclear division arrests after DNA-replication but before nuclear divisions after shift to sporulation medium

0142	GO_TERM:[meiosis I] P-Value:1.0e-11 OVERLAP:[Synaptonemal complex (SC)] <490> SIZE:5
YMR224C	[MRE11] Subunit of a complex with Rad50p and Xrs2p (RMX complex) that functions in repair of DNA double-strand breaks and in telomere stability, exhibits nuclease activity that appears to be required for RMX function; widely conserved Null mutant is viable, methyl methanesulfonate-sensitive and displays hyper-recombination in mitosis. mre11 is rescued by spo13 and epistatic to rad50s, suggesting it is an early recombination function.
YDR285W	[ZIP1] Transverse filament protein of the synaptonemal complex; required for normal levels of meiotic recombination and pairing between homologous chromosome during meiosis; potential Cdc28p substrate Null mutant is viable and shows defects in meiosis
YGL249W	[ZIP2] Meiosis-specific protein involved in normal synaptonemal complex formation and pairing between homologous chromosomes during meiosis Null mutant is viable but is defective in chromosome synapsis, but not chromosome pairing, and causes meiosis I non-disjunction and reduced homologous recombination
YLR394W	[CST9] Protein required for synaptonemal complex formation, may have a role in meiotic recombination; localizes to synapsis initiation sites on meiotic chromosomes; potential Cdc28p substrate Null mutant is viable, but exhibits delayed sporulation and reduced viability; overexpression of CSTs induces chromosome loss
YDL154W	[MSH5] Protein of the MutS family, forms a dimer with Msh4p that facilitates crossovers between homologs during meiosis; msh5-Y823H mutation confers tolerance to DNA alkylating agents; homologs present in C. elegans and humans mutS homolog Null mutant is viable. Diploids lacking the MSH5 gene display decreased levels of spore viability, increased levels of meiosis I chromosome nondisjuction, and decreased levels of reciprocal exchange between, but not within, homologs. Gene conversion is not reduced. Msh5 mutants are phenotypically similar to mutants in the meiosis-specific gene MSH4. msh5 is epistatic to msh4, suggesting they act in the same pathway.
YFL003C	[MSH4] Protein involved in meiotic recombination, required for normal levels of crossing over, colocalizes with Zip2p to discrete foci on meiotic chromosomes, has homology to bacterial MutS protein meiosis specific protein, E.coli MutS protein, localizes to discrete sites on meiotic chromosomes Null mutant is viable, has no apparent defect in mismatch repair, wild-type levels of gene conversion and postmeiotic segregation

0143	GO_TERM:[site of polarized growth] P-Value:3.3e-12 OVERLAP:[Actin-associated proteins] <140.20.20> SIZE:25
YER032W	[FIR1] Protein involved in 3' mRNA processing, interacts with Ref2p; potential Cdc28p substrate participant in 3' mRNA processing (putative) Null mutant is viable, shows slow growth in all media
YNL058C	
YAL031C	[GIP4] Cytoplasmic Glc7p-interacting protein, potential Cdc28p substrate

YCL027W	[FUS1] Membrane protein localized to the shmoo tip, required for cell fusion; expression regulated by mating pheromone; proposed to coordinate signaling, fusion, and polarization events required for fusion; potential Cdc28p substrate Null mutant is viable; in fus1 x fus1 matings there is an interruption of the mating process just before cytoplasmic fusion
YGR126W	
YOR188W	[MSB1] Protein involved in positive regulation of both 1,3-beta-glucan synthesis and the Pkc1p-MAPK pathway, potential Cdc28p substrate; multicopy suppressor of temperature-sensitive mutations in CDC24 and CDC42, and of mutations in BEM4 multicopy suppressor of cdc24 and cdc42 ts mutations
YNL293W	[MSB3] GTPase-activating protein for Sec4p and several other Rab GTPases, regulates exocytosis via its action on Sec4p, also required for proper actin organization; similar to Msb4p; both Msb3p and Msb4p localize to sites of polarized growth GTPase activating protein (GAP) for Ypt6 Null mutant is viable. msb3/msb4 double mutant exhibits slow growth and disorganized actin cytoskeleton
YNL271C	[BNI1] Formin, nucleates the formation of linear actin filaments, involved in cell processes such as budding and mitotic spindle orientation which require the formation of polarized actin cables, functionally redundant with BNR1 formin, involved in spindle orientation Null mutant is viable, bni1 bnr1 double deletion mutants are temperature sensitive and are deficient in bud emergence, exhibit a random distribution of cortical actin patches and often become multinucleate at the restrictive temperature; rho1 bni1 double mutants exhibit synthetic lethality
**YLR319C**	[BUD6] Actin- and formin-interacting protein, involved in actin cable nucleation and polarized cell growth; isolated as bipolar budding mutant; potential Cdc28p substrate Null mutant is viable; mutants exhibit severe disruption of the actin cytoskeleton; deletion strains have a depolarized cytoskeleton, mitotic delay, and probable cytokinesis defects
YOL112W	[MSB4] GTPase-activating protein of the Ras superfamily that acts primarily on Sec4p, localizes to the bud site and bud tip, has similarity to Msb3p; msb3 msb4 double mutation causes defects in secretion and actin organization Null mutant is viable. msb3/msb4 double mutant exhibits slow growth and disorganized actin cytoskeleton
YER149C	[PEA2] Coiled-coil polarisome protein required for polarized morphogenesis, cell fusion, and low affinity Ca2+ influx; forms polarisome complex with Bni1p, Bud6p, and Spa2p; localizes to sites of polarized growth coiled-coil domain Null mutant is viable, exhibits defects in mating that subtly affect mating efficiency; pea2 mutants form bilobed, peanut-like shapes when treated with pheromone, such that they can mate with wild-type but not a mating-enfeebled partner
YLL021W	[SPA2] Component of the polarisome, which functions in actin cytoskeletal organization during polarized growth; acts as a scaffold for Mkk1p and Mpk1p cell wall integrity signaling components; potential Cdc28p substrate impaired ability to form schmoos, do not mate well with other spa2 mutants

0144	GO_TERM:[cytoskeleton organization and biogenesis] P-Value:4.3e-01 OVERLAP:[Phenylalaninine-tRNA-ligase] <330> SIZE:3
YNL024C	
**YPR047W**	[MSF1] Mitochondrial phenylalanyl-tRNA synthetase alpha subunit, active as a monomer, unlike the cytoplasmic subunit which is active as a dimer complexed to a beta subunit dimer; similar to the alpha subunit of E. coli phenylalanyl-tRNA synthetase phenylalanyl-tRNA synthetase alpha subunit
YDR439W	[LRS4] Protein involved in rDNA silencing; positively charged coiled-coil protein with limited similarity to myosin loses rDNA silencing
YDR389W	[SAC7] GTPase activating protein (GAP) for Rho1p, involved in signaling to the actin cytoskeleton, null mutations suppress tor2 mutations and temperature sensitive mutations in actin; potential Cdc28p substrate GTPase activating protein (GAP) for RHO1 null mutant is viable, has growth and actin assembly defects at low temperatures, displays allele-specific suppression and double mutant lethality with actin mutations, suprresses tor mutations
YJR091C	[JSN1] Member of the Puf family of RNA-binding proteins, interacts with mRNAs encoding membrane-associated proteins; overexpression suppresses a tub2-150 mutation and causes increased sensitivity to benomyl in wild-type cells Overexpression suppresses some tub2 alleles and confers greater benomyl sensitivity
YJL107C	
YOR265W	[RBL2] Protein involved in microtubule morphogenesis, required for protection from excess free beta-tubulin; proposed to be involved the folding of beta-tubulin tubulin folding cofactor A Overexpression rescues lethality caused by excess beta-tubulin

0145	GO_TERM:[bud] P-Value:9.3e-06 OVERLAP:[Kel1p/Kel2p complex] <265> SIZE:2
YOR276W	[CAF20] Phosphoprotein of the mRNA cap-binding complex involved in translational control, repressor of cap-dependent translation initiation, competes with eIF4G for binding to eIF4E 20 kDa protein|functional and limited sequence similarity to EAP1|functionally analogous to mammalian 4E-BPs
YDR348C	
YAL024C	[LTE1] Putative GDP/GTP exchange factor required for mitotic exit at low temperatures; acts as a guanine nucleotide exchange factor (GEF) for Tem1p, which is a key regulator of mitotic exit; physically associates with Ras2p-GTP lethal at low temperature (8 degrees C)
YCL040W	[GLK1] Glucokinase, catalyzes the phosphorylation of glucose at C6 in the first irreversible step of glucose metabolism; one of three glucose phosphorylating enzymes; expression regulated by non-fermentable carbon sources glucokinase
YDR516C	[EMI2] Non-essential protein of unknown function required for transcriptional induction of the early meiotic-specific transcription factor IME1, also required for sporulation

YBR169C	[SSE2] Member of the heat shock protein 70 (HSP70) family; may be involved in protein folding; localized to the cytoplasm; highly homologous to the heat shock protein Sse1p HSP70 family|SSE1 homolog
YKR090W	[PXL1] LIM domain-containing protein that localizes to sites of polarized growth, required for selection and/or maintenance of polarized growth sites, may modulate signaling by the GTPases Cdc42p and Rho1p; has similarity to metazoan paxillin
YLR096W	[KIN2] Serine/threonine protein kinase involved in regulation of exocytosis; localizes to the cytoplasmic face of the plasma membrane; closely related to Kin1p
**YGR238C**	[KEL2] Protein that functions in a complex with Kel1p to negatively regulate mitotic exit, interacts with Tem1p and Lte1p; localizes to regions of polarized growth; potential Cdc28p substrate
**YHR158C**	[KEL1] Protein required for proper cell fusion and cell morphology; functions in a complex with Kel2p to negatively regulate mitotic exit, interacts with Tem1p and Lte1p; localizes to regions of polarized growth; potential Cdc28p substrate The null mutant is viable but shows a moderate defect in cell fusion during mating.

0146	GO_TERM:[transcription regulator activity] P-Value:1.2e-06 OVERLAP:[SBF complex] <510.190.60> SIZE:2
YDL056W	[MBP1] Transcription factor involved in regulation of cell cycle progression from G1 to S phase, forms a complex with Swi6p that binds to MluI cell cycle box regulatory element in promoters of DNA synthesis genes transcription factor
YNR009W	[NRM1] Putative transcriptional repressor of MBF (MCB binding factor) target genes
**YLR182W**	[SWI6] Transcription cofactor, forms complexes with DNA-binding proteins Swi4p and Mbp1p to regulate transcription at the G1/S transition; involved in meiotic gene expression; localization regulated by phosphorylation; potential Cdc28p substrate transcription factor Null mutant is viable and deficient in homothallic switching
**YER111C**	[SWI4] DNA binding component of the SBF complex (Swi4p-Swi6p), a transcriptional activator that in concert with MBF (Mbp1-Swi6p) regulates late G1-specific transcription of targets including cyclins and genes required for DNA synthesis and repair transcription factor Null mutant is viable, deficient in homothallic switching, and temperature sensitive
YOR083W	[WHI5] Protein that regulates the critical cell size required for passage through Start and commitment to cell division; may act upstream of SCB binding factor (SBF) and MCB binding factor (MBF); periodically expressed in G1 function unknown Null: small critical cell size

0147	GO_TERM:[kinase regulator activity] P-Value:7.3e-06 OVERLAP:[Sin3 complex] <510.190.150> SIZE:4
YKR091W	[SRL3] Cytoplasmic protein that, when overexpressed, suppresses the lethality of a rad53 null mutation; potential Cdc28p substrate
**YNL309W**	[STB1] Protein with a role in regulation of MBF-specific transcription at Start, phosphorylated by Cln-Cdc28p kinases in vitro; unphosphorylated form binds Swi6p and binding is required for Stb1p function; expression is cell-cycle regulated
YGR108W	[CLB1] B-type cyclin involved in cell cycle progression; activates Cdc28p to promote the transition from G2 to M phase; accumulates during G2 and M, then targeted via a destruction box motif for ubiquitin-mediated degradation by the proteasome B-type cyclin
YBR135W	[CKS1] Subunit of the Cdc28 protein kinase, required for mitotic proteolysis, may also be involved in the proteolysis of the G1 cyclins Cdc28 protein kinase subunit Null mutant is inviable and arrests in G1.
YLR079W	[SIC1] Inhibitor of Cdc28-Clb kinase complexes that controls G1/S phase transition, preventing premature S phase and ensuring genomic integrity; phosphorylation targets Sic1p for SCF(CDC4)-dependent turnover; functional homolog of mammalian Kip1 Null mutant is viable, shows increased frequency of broken and lost chromosomes; sic1 deletion mutant rescues lethality of cln1 cln2 cln3 triple mutant.

0148	GO_TERM:[protein kinase cascade] P-Value:5.3e-04
YGR221C	[TOS2] Protein involved in localization of Cdc24p to the site of bud growth; may act as a membrane anchor; localizes to the bud neck and bud tip; potentially phosphorylated by Cdc28p
YBL105C	[PKC1] Protein serine/threonine kinase essential for cell wall remodeling during growth; localized to sites of polarized growth and the mother-daughter bud neck; homolog of the alpha, beta, and gamma isoforms of mammalian protein kinase C (PKC) The null mutant is inviable and lyses rapidly in hypotonic media. Cells lacking PKC1 arrest growth with small buds at a point after DNA replication but prior to mitosis.
YOL100W	[PKH2] Serine/threonine protein kinase involved in sphingolipid-mediated signaling pathway that controls endocytosis; activates Ypk1p and Ykr2p, components of signaling cascade required for maintenance of cell wall integrity; redundant with Pkh1p Null mutant is viable; pkh1, pkh2 double mutant is lethal

0149	GO_TERM:[nucleus] P-Value:5.3e-02 OVERLAP:[rRNA splicing] <440.30.20> SIZE:24

YLR401C	[DUS3] Dihydrouridine synthase, member of a widespread family of conserved proteins including Smm1p, Dus1p, and Dus4p; contains a consensus oleate response element (ORE) in its promoter region dihydrouridine synthase 3
YIL031W	[ULP2] Peptidase that deconjugates Smt3/SUMO-1 peptides from proteins, plays a role in chromosome cohesion at centromeric regions and recovery from checkpoint arrest induced by DNA damage or DNA replication defects; potential Cdc28p substrate
YJL092W	[HPR5] DNA helicase and DNA-dependent ATPase involved in DNA repair, required for proper timing of commitment to meiotic recombination and the transition from Meiosis I to Meiosis II; potential Cdc28p substrate DNA helicase Null mutant is viable, radiation (ultraviolet or ionizing sensitive), loss of function results in RAD52-dependent hyperrecombination suggesting recombination suppression occurs by antagonizing the Rad52 recombinational repair pathway; wild-type suppresses mitotic recombination; some mutant alleles have lower spore viability which is not rescued by spo13, suggesting they affect a late recombination function; hpr5 mutations are rad6 suppressors. Growth defects of mgs1 rad18 double mutants are suppressed by a mutation in HPR5.
**YLR223C**	[IFH1] Essential protein with a highly acidic N-terminal domain; IFH1 exhibits genetic interactions with FHL1, overexpression interferes with silencing at telomeres and HM loci; potential Cdc28p substrate Null mutant is inviable, ifh1 fhl1 double deletion mutant is viable

0150	GO_TERM:[spindle pole body duplication in nuclear envelope] P-Value:4.1e-08
YPL255W	[BBP1] Protein required for the spindle pole body (SPB) duplication, localized at the central plaque periphery; forms a complex with a nuclear envelope protein Mps2p and SPB components Spc29p and Kar1p; required for mitotic functions of Cdc5p Null mutant is inviable; cells depleted of Bbp1p are defective in nuclear segregation, bud formation, cytokinesis and nuclear spindle formation; overexpression gives ascus that contains asci instead of spores
YGL075C	[MPS2] Essential membrane protein localized at the nuclear envelope and spindle pole body (SPB), required for insertion of the newly duplicated SPB into the nuclear envelope; potentially phosphorylated by Cdc28p Null mutant is inviable, however some null spore clones can survive with abnormal ploidy; the mps2-1 mutant is incapable of proper duplication of the SPB, resulting in a defective pole that only nucleates cytoplasmic microtubules. Overexpression of MPS2 in a cim5-1 ts mutant is toxic to cells.
YLR457C	[NBP1] Spindle pole body (SPB) component, required for the insertion of the duplication plaque into the nuclear membrane during SPB duplication; essential for bipolar spindle formation; component of the Mps2p-Bbp1p complex

0151	GO_TERM:[binding] P-Value:2.8e-02
YDR501W	[PLM2] Protein required for partitioning of the 2-micron plasmid Null mutant is viable and shows 2mu-m plasmid instability
YKR078W	

0152	GO_TERM:[G2/M-specific transcription in mitotic cell cycle] P-Value:3.5e-06
YNL068C	[FKH2] Transcription factor of the forkhead family that regulates the cell cycle and pseudohyphal growth; also involved in chromatin silencing at HML and HMR; potential Cdc28p substrate forkhead protein
YOR372C	[NDD1] Transcriptional activator essential for nuclear division; localized to the nucleus; essential component of the mechanism that activates the expression of a set of late-S-phase-specific genes Null mutant is inviable and arrests prior to nuclear division but after DNA replication; cells are large budded with short mitotic spindles.

0153	GO_TERM:[G2/M transition of mitotic cell cycle] P-Value:1.0e-07 OVERLAP:[Cdc28p complexes] <133.10> SIZE:10
**YBR160W**	[CDC28] Catalytic subunit of the main cell cycle cyclin-dependent kinase (CDK); alternately associates with G1 cyclins (CLNs) and G2/M cyclins (CLBs) which direct the CDK to specific substrates cyclin-dependent protein kinase arrests at G1/S transition arrests at G2/M transition
**YPR119W**	[CLB2] B-type cyclin involved in cell cycle progression; activates Cdc28p to promote the transition from G2 to M phase; accumulates during G2 and M, then targeted via a destruction box motif for ubiquitin-mediated degradation by the proteasome B-type cyclin
YAL028W	[FRT2] Tail-anchored endoplasmic reticulum membrane protein, interacts with homolog Frt1p but is not a substrate of calcineurin (unlike Frt1p), promotes growth in conditions of high Na+, alkaline pH, or cell wall stress; potential Cdc28p substrate
YJL187C	[SWE1] Protein kinase that regulates the G2/M transition by inhibition of Cdc28p kinase activity; localizes to the nucleus and to the daughter side of the mother-bud neck; homolog of S. pombe Wee1p; potential Cdc28p substrate tyrosine kinase Null mutant is viable. Defective for morphogenesis checkpoint
YDR330W	[UBX5] UBX (ubiquitin regulatory X) domain-containing protein that interacts with Cdc48p

**YPR120C**	[CLB5] B-type cyclin involved in DNA replication during S phase; activates Cdc28p to promote initiation of DNA synthesis; functions in formation of mitotic spindles along with Clb3p and Clb4p; most abundant during late G1 phase B-type cyclin Null mutant is viable, but has an extended S phase

0154	GO_TERM:[catalytic activity] P-Value:4.1e-01
YOR018W	[ROD1] Membrane protein; overexpression confers resistance to the GST substrate o-dinitrobenzene as well as to zinc and calcium; contains 2 PY motifs, which are required for Rod1p interaction with Rsp5p, a hect-type ubiquitin ligase Null mutant is viable but is hypersensitive to o-dinitrobenzene, calcium, and zinc
YGL158W	[RCK1] Protein kinase involved in the response to oxidative stress; identified as suppressor of S. pombe cell cycle checkpoint mutations
YNL284C-A	
YNL186W	[UBP10] Ubiquitin-specific protease that deubiquitinates ubiquitin-protein moieties; may regulate silencing by acting on Sir4p; involved in posttranscriptionally regulating Gap1p and possibly other transporters; primarily located in the nucleus Null mutant is viable, exhibits decreased telomeric silencing; UBP10(DOT4) overexpression reduces silencing at the HM, rDNA, and telomeric loci
YGR251W	
YKL078W	[DHR2] Predominantly nucleolar DEAH-box RNA helicase, required for 18S rRNA synthesis Required for 18S ribosomal RNA synthesis Null: essential

0155	GO_TERM:[filamentous growth] P-Value:1.2e-09 OVERLAP:[Cdc28p complexes] <133.10> SIZE:10
**YMR199W**	[CLN1] G1 cyclin involved in regulation of the cell cycle; activates Cdc28p kinase to promote the G1 to S phase transition; late G1 specific expression depends on transcription factor complexes, MBF (Swi6p-Mbp1p) and SBF (Swi6p-Swi4p) G1 cyclin Null mutant is viable, exhibits G1 arrest
YDR480W	[DIG2] Regulatory protein of unknown function, pheromone-inducible, involved in the regulation of mating-specific genes and the invasive growth pathway, required for MAP-kinase imposed repression, inhibits pheromone-responsive transcription MAP kinase-associated protein Null mutant is viable; dig1 dig2 double mutants show constitutive mating pheromone specific gene expression and invasive growth
YPL049C	[DIG1] Regulatory protein of unknown function, constitutively-expressed, involved in the regulation of mating-specific genes and the invasive growth pathway, required for MAP-kinase imposed repression, inhibits pheromone-responsive transcription MAP kinase-associated protein Null mutant is viable, shows abnormal bud morphology; dig1 dig2 double mutants show constitutive mating defect and invasive growth; overexpression causes pheromone resistance
YHR084W	[STE12] Transcription factor that is activated by a MAP kinase signaling cascade, activates genes involved in mating or pseudohyphal/invasive growth pathways; cooperates with Tec1p transcription factor to regulate genes specific for invasive growth transcription factor Null mutant is viable but sterile; homozygous mutant diploids exhibit defects in pseudohyphal growth
YBR083W	[TEC1] Transcription factor required for full Ty1 epxression, Ty1-mediated gene activation, and haploid invasive and diploid pseudohyphal growth; TEA/ATTS DNA-binding domain family member TEA/ATTS DNA-binding domain family transcription factor
YER109C	[FLO8] Transcription factor required for flocculation, diploid filamentous growth, and haploid invasive growth; genome reference strain S288C and most laboratory strains have a mutation in this gene transcriptional activator of FLO1 (putative) Null mutant is viable; wild-type gene is required for flocculation and for pseudo-hyphal growth
YMR164C	[MSS11] Transcription factor involved in regulation of invasive growth and starch degradation; controls the activation of MUC1 and STA2 in response to nutritional signals 758 amino acid polypeptide with poly-glutamine and poly-asparagine domains Null mutant is viable, exhibits diminished transcription of STA2; multiple copies suppress repressive effect of STA10, enhance expression of STA2 in non-STA10 strains

0156	GO_TERM:[signal transduction] P-Value:2.2e-09 OVERLAP:[STE5-MAPK complex] <470.20> SIZE:5
YGL178W	[MPT5] Protein that specifically binds to mRNAs encoding chromatin modifiers and spindle pole body components; has roles in longevity, in maintenance of cell wall integrity, and in sensitivity to and recovery from pheromone arrest Null mutant is viable, temperature sensitive
YLR452C	[SST2] GTPase-activating protein for Gpa1p, regulates desensitization to alpha factor pheromone; also required to prevent receptor-independent signaling of the mating pathway; member of the RGS (regulator of G-protein signaling) family GTPase activating protein (GAP)|RGS (regulator of G-protein signalling) family Null mutants are viable and exhibit increased sensitivity to mating factors
**YGR040W**	[KSS1] Mitogen-activated protein kinase (MAPK) involved in signal transduction pathways that control filamentous growth and pheromone response MAP kinase
YLR313C	[SPH1] Protein involved in shmoo formation and bipolar bud site selection; homologous to Spa2p, localizes to sites of polarized growth in a cell cycle dependent- and Spa2p-dependent manner, interacts with MAPKKs Mkk1p, Mkk2p, and Ste7p Spa2p homolog

**YBL016W**	[FUS3] Mitogen-activated protein kinase involved in mating pheromone response; activated by phoshporylation by Ste7p; provides specificity during the mating vs. filamentous growth response by phosphorylating transcriptional and cytoplasmic targets CDC28/cdc2 related protein kinase sterile; divide continuously in the presence of pheromones; form prezygotes with wild-type cells of opposite mating type but cannot undergo cell fusion
**YDR103W**	[STE5] Scaffold protein that, in response to pheromone, shuttles from the nucleus to the plasma membrane and assembles kinases Ste11p, Ste7p, and Fus3p into a specific signaling complex; active oligomeric form interacts with Ste4p-Ste18p complex Null mutant is viable but sterile. Overexpression of STE5 suppresses the temperature sensitivity of a cdc25 allele.
**YDL159W**	[STE7] Signal transducing MAP kinase kinase involved in pheromone response, where it phosphorylates Fus3p, and in the pseudohyphal/invasive growth pathway, through phosphorylation of Kss1p; phosphorylated by Ste11p, degraded by ubiquitin pathway MAP kinase kinase (MEK)
**YLR362W**	[STE11] Signal transducing MEK kinase involved in pheromone response and pseudohyphal/invasive growth pathways, where it phosphorylates Ste7p, and the high osmolarity response pathway, via phosphorylation of Pbs2p; regulated by Ste20p and Ste50p

0157	GO_TERM:[signal transduction] P-Value:6.9e-08 OVERLAP:[H+-ATPase, plasma mebrane] <210> SIZE:4
YDL035C	[GPR1] Plasma membrane G protein coupled receptor (GPCR) that interacts with the heterotrimeric G protein alpha subunit, Gpa2p, and with Plc1p; sensor that integrates nutritional signals with the modulation of cell fate via PKA and cAMP synthesis G protein coupled receptor (GPCR)
YPL268W	[PLC1] Phosphoinositide-specific phospholipase C, hydrolyzes phosphatidylinositol 4,5-biphosphate (PIP2) to generate inositol 1,4,5-triphosphate (IP3) and 1,2-diacylglycerol (DAG); involved in kinetochore function and pseudohyphal differentiation phosphoinositide-specific phospholipase C
**YPL036W**	[PMA2] Plasma membrane H+-ATPase, isoform of Pma1p, involved in pumping protons out of the cell; regulator of cytoplasmic pH and plasma membrane potential plasma membrane ATPase
YAL056W	[GPB2] Proposed beta subunit of the heterotrimeric G protein that interacts with the receptor Grp1p, has signaling role in response to nutrients; involved in regulation of pseudohyphal growth through cAMP levels; homolog of Gpb1p Deletion causes a high PKA phenotype.
YER020W	[GPA2] Nucleotide binding alpha subunit of the heterotrimeric G protein that interacts with the receptor Gpr1p, has signaling role in response to nutrients; green fluorescent protein (GFP)-fusion protein localizes to the cell periphery nucleotide binding regulatory protein
YGL121C	[GPG1] Proposed gamma subunit of the heterotrimeric G protein that interacts with the receptor Grp1p; involved in regulation of pseudohyphal growth; requires Gpb1p or Gpb2p to interact with Gpa2p Heterotrimeric G protein gamma subunit mimic Null: A modest reduction in pseudohyphal differentiation, invasive growth, and FLO11 expression
YOR371C	[GPB1] Proposed beta subunit of the heterotrimeric G protein that interacts with the receptor Grp1p, has signaling role in response to nutrients; involved in regulation of pseudohyphal growth through cAMP levels; homolog of Gpb2p

0158	GO_TERM:[vacuolar transport] P-Value:4.9e-05
YBL056W	[PTC3] Type 2C protein phosphatase; dephosphorylates Hog1p (see also Ptc2p) to limit maximal kinase activity induced by osmotic stress; dephosphorylates T169 phosphorylated Cdc28p (see also Ptc2p); role in DNA checkpoint inactivation protein phosphatase type 2C
YBR097W	[VPS15] Myristoylated serine/threonine protein kinase involved in vacuolar protein sorting; functions as a membrane-associated complex with Vps34p; active form recruits Vps34p to the Golgi membrane; also detected in mitochondria serine/threonine protein kinase defective vacuolar protein localization
YLR240W	[VPS34] Phosphatidylinositol 3-kinase responsible for the synthesis of phosphatidylinositol 3-phosphate; forms membrane-associated signal transduction complex with Vps15p to regulate protein sorting; similar to p110 subunit of mammalian PI 3-kinase phosphatidylinositol 3-kinase temperature sensitive, defective vacuolar protein sorting
YBR128C	[ATG14] Subunit of an autophagy-specific phosphatidylinositol 3-kinase complex (with Vps34p, Vps15p, and Vps30p) required for organization of a pre-autophagosomal structure; ATG14 transcription is activated by Gln3p during nitrogen starvation Null mutant is viable but defective in autophagy.
YLR360W	[VPS38] Part of a Vps34p phosphatidylinositol 3-kinase complex that functions in carboxypeptidase Y (CPY) sorting; binds Vps30p and Vps34p to promote production of phosphatidylinositol 3-phosphate (PtdIns3P) which stimulates kinase activity
YPL120W	[VPS30] Protein that forms a membrane-associated complex with Apg14p that is essential for autophagy; involved in a retrieval step of the carboxypeptidase Y receptor, Vps10p, to the late Golgi from the endosome; involved in vacuolar protein sorting Vacuolar hydrolases sorting receptor Vps10p is mislocalized in vps30 mutants.

0159	GO_TERM:[protein kinase regulator activity] P-Value:3.1e-04 OVERLAP:[Cdc28p complexes] <133.10> SIZE:10
YJL157C	[FAR1] Cyclin-dependent kinase inhibitor that mediates cell cycle arrest in response to pheromone; also forms a complex with Cdc24p, Ste4p, and Ste18p that may specify the direction of polarized growth during mating; potential Cdc28p substrate Cdc28p kinase inhibitor

**YAL040C**	[CLN3] G1 cyclin involved in cell cycle progression; activates Cdc28p kinase to promote the G1 to S phase transition; plays a role in regulating transcription of the other G1 cyclins, CLN1 and CLN2; regulated by phosphorylation and proteolysis G1 cyclin Null mutant is viable; dominant mutation causes alpha-factor resistance and small cell size; chromosomal deletion increases cell volume
YNL197C	[WHI3] RNA binding protein that binds to and sequesters the G1 cyclin CLN3 mRNA; regulates cell fate and dose-dependently inhibits passage through Start by regulating the critical cell size requirement necessary for cell cycle progression RNA binding protein (putative) Null mutant is viable and defective in filamentous growth

0160	GO_TERM:[heterotrimeric G-protein complex] P-Value:8.4e-10 OVERLAP:[Pheromone response pathway] <470.30.10> SIZE:3
**YJR086W**	[STE18] G protein gamma subunit, forms a dimer with Ste4p to activate the mating signaling pathway, forms a heterotrimer with Gpa1p and Ste4p to dampen signaling; C-terminus is palmitoylated and farnesylated, which are required for normal signaling G protein gamma subunit|coupled to mating factor receptor The null mutant is viable but sterile. Sst1 sst2 double mutants and scg1 mutants can be suppressed by a null allele of ste18.
**YOR212W**	[STE4] G protein beta subunit, forms a dimer with Ste18p to activate the mating signaling pathway, forms a heterotrimer with Gpa1p and Ste18p to dampen signaling; may recruit Rho1p to the polarized growth site during mating; contains WD40 repeats G protein beta subunit|coupled to mating factor receptor
YFL026W	[STE2] Receptor for alpha-factor pheromone; seven transmembrane-domain GPCR that interacts with both pheromone and a heterotrimeric G protein to initiate the signaling response that leads to mating between haploid a and alpha cells G protein coupled receptor (GPCR)|alpha-factor pheromone receptor|seven-transmembrane domain protein
**YHR005C**	[GPA1] GTP-binding alpha subunit of the heterotrimeric G protein that couples to pheromone receptors; negatively regulates the mating pathway by sequestering G(beta)gamma and by triggering an adaptive response; activates the pathway via Scp160p G protein alpha subunit|coupled to mating factor receptor The null mutation is inviable in haploids but not diploids. Gpa1 mutants exhibit specific defects in the pheromone responsiveness of both a and alpha cells.

0161	GO_TERM:[poly(A)-specific ribonuclease activity] P-Value:3.9e-06
YGL094C	[PAN2] Essential subunit of the Pan2p-Pan3p poly(A)-ribonuclease complex, which acts to control poly(A) tail length and regulate the stoichiometry and activity of postreplication repair complexes poly(A) ribonuclease 135 kDa subunit Null mutant is viable but shows an increase in average length of mRNA poly(A) tails and a loss of Pab1p-stimulated poly(A) ribonuclease activity in vitro
YKL025C	[PAN3] Essential subunit of the Pan2p-Pan3p poly(A)-ribonuclease complex, which acts to control poly(A) tail length and regulate the stoichiometry and activity of postreplication repair complexes Pab1p-dependent poly(A) ribonuclease (PAN) 76 kDa subunit Null mutant is viable but lacks Pab1p-dependent poly(A) ribonuclease activity in vitro; Tn3 insertion into PAN3 causes hypersensitivity to calcofluor white
YGR178C	[PBP1] Protein interacting with poly(A)-binding protein Pab1p; likely involved in controlling the extent of mRNA polyadenylation; forms a complex with Mkt1p that may regulate HO translation; interacts with Lsm12p in a copurification assay Null mutant is viable; other mutant suppresses pab1 null mutant.
YBR249C	[ARO4] 3-deoxy-D-arabino-heptulosonate-7-phosphate (DAHP) synthase, catalyzes the first step in aromatic amino acid biosynthesis and is feedback-inhibited by tyrosine 3-deoxy-D-arabino-heptulosonate 7-phosphate (DAHP) synthase isoenzyme
YMR076C	[PDS5] Protein required for establishment and maintenance of sister chromatid condensation and cohesion, colocalizes with cohesin on chromosomes in an interdependent manner, may function as a protein-protein interaction scaffold

0162	GO_TERM:[nucleus] P-Value:2.3e-01
YDL214C	[PRR2] Protein kinase with a possible role in MAP kinase signaling in the pheromone response pathway protein kinase
YJL051W	[YJL051W] Bud tip localized protein of unknown function; mRNA is targeted to the bud by a She2p dependent transport system; mRNA is cell cycle regulated via Fkh2p, peaking in G2/M phase; null mutant displays increased levels of spontaneous Rad52 foci
YIL036W	[CST6] Basic leucine zipper (bZIP) transcription factor of the ATF/CREB family, activates transcription of genes involved in utilization of non-optimal carbon sources; involved in telomere maintenance basic leucine zipper (bZIP) transcription factor Overexpression of CSTs induces chromosome loss
YDL053C	[PBP4] Pbp1p binding protein, interacts strongly with Pab1p-binding protein 1 (Pbp1p) in the yeast two-hybrid system; also interacts with Lsm12p in a copurification assay
YHR121W	[LSM12] Protein of unknown function that interacts with Pbp1p and Pbp4p and associates with ribosomes; contains an RNA-binding LSM domain and an AD domain; may play a role in RNA processing

0163	GO_TERM:[regulation of catalytic activity] P-Value:1.5e-04 OVERLAP:[Cdc28p complexes] <133.10> SIZE:10

**YDL155W**	[CLB3] B-type cyclin involved in cell cycle progression; activates Cdc28p to promote the G2/M transition; may be involved in DNA replication and spindle assembly; accumulates during S phase and G2, then targeted for ubiquitin-mediated degradation B-type cyclin
YPL267W	[ACM1] Protein of unknown function, potential Cdc28p substrate

0164	GO_TERM:[cellular morphogenesis during vegetative growth] P-Value:5.2e-16
YIL129C	[TAO3] Protein involved in cell morphogenesis and proliferation, associated with protein kinase Cbk1p; mutants activate OCH1 transcription tao3 mutants activate OCH1 transcription and form aggregates. Null mutant is viable in the W303 background
YKL189W	[HYM1] Component of the RAM signaling network that is involved in regulation of Ace2p activity and cellular morphogenesis, interacts with Kic1p and Sog2p, localizes to sites of polarized growth during budding and during the mating response
YHR102W	[KIC1] Protein kinase of the PAK/Ste20 kinase family, required for cell integrity possibly through regulating 1,6-beta-glucan levels in the wall; physically interacts with Cdc31p (centrin), which is a component of the spindle pole body
YOR353C	[SOG2] Protein required for cell morphogenesis and cell separation after mitosis
YLR131C	[ACE2] Transcription factor that activates expression of early G1-specific genes, localizes to daughter cell nuclei after cytokinesis and delays G1 progression in daughters, localization is regulated by phosphorylation; potential Cdc28p substrate zinc finger transcription factor Null mutant is viable, exhibits decreased CUP1 mRNA expression
YIR016W	
YOL036W	
YFL034C-B	[MOB2] Component of the RAM signaling network, localizes and activates the Ace2p in the daughter cell nucleus to direct daughter cell-specific transcription of several genes involved in cell separation; Mob1p-like protein Mob1p-like protein Null is viable; other mutants have synthetic interactions with MPS1
YNL161W	[CBK1] Serine/threonine protein kinase that regulates cell morphogenesis pathways; involved in cell wall biosynthesis, apical growth, proper mating projection morphology, bipolar bud site selection in diploid cells, and cell separation serine/threonine protein kinase Null mutation is viable; shows alpha factor resistance; in liquid culture large aggregates of cells are formed

0165	GO_TERM:[telomere capping] P-Value:6.4e-09
YGL256W	[ADH4] Alcohol dehydrogenase type IV, dimeric enzyme demonstrated to be zinc-dependent despite sequence similarity to iron-activated alcohol dehydrogenases; transcription is induced in response to zinc deficiency; alcohol dehydrogenase isoenzyme IV alcohol dehydrogenase isoenzyme IV
YDL220C	[CDC13] Single stranded DNA-binding protein found at TG1-3 telomere G-tails; regulates telomere replication through recruitment of specific sub-complexes, but the essential function is telomere capping single-stranded TG1-3 telomere G-tails binding protein
YDR082W	[STN1] Telomere end-binding and capping protein, plays a key role with Pol12p in linking telomerase action with completion of lagging strand synthesis, and in a regulatory step required for telomere capping
YLR010C	[TEN1] Protein that regulates telomeric length; protects telomeric ends in a complex with Cdc13p and Stn1p

0166	GO_TERM:[protein kinase activity] P-Value:4.3e-02
YMR111C	
YJR142W	
YPL150W	
YPR091C	
YDL128W	[VCX1] Vacuolar H+/Ca2+ exchanger involved in control of cytosolic Ca2+ concentration; has similarity to sodium/calcium exchangers, including the bovine Na+/Ca2+,K+ antiporter vacuolar H+/Ca2+ exchanger Null mutant is viable, sensitive to high Ca2+ conditions
YIL135C	[VHS2] Cytoplasmic protein of unknown function; identified as a high-copy suppressor of the synthetic lethality of a sis2 sit4 double mutant, suggesting a role in G1/S phase progression; similar to Mlf3p
YLR248W	[RCK2] Protein kinase involved in the response to oxidative and osmotic stress; identified as suppressor of S. pombe cell cycle checkpoint mutations

0167	GO_TERM:[catalytic activity] P-Value:1.8e-01 OVERLAP:[cytoplasmic ribosomal large subunit] <500.40.10> SIZE:81

**YJR094W-A**	[RPL43B] Protein component of the large (60S) ribosomal subunit, identical to Rpl43Ap and has similarity to rat L37a ribosomal protein ribosomal protein L43B
YGL245W	[GUS1] Glutamyl-tRNA synthetase (GluRS), forms a complex with methionyl-tRNA synthetase (Mes1p) and Arc1p; complex formation increases the catalytic efficiency of both tRNA synthetases and ensures their correct localization to the cytoplasm
YHR111W	[UBA4] Protein that activates Urm1p before its conjugation to proteins (urmylation); one target is the thioredoxin peroxidase Ahp1p, suggesting a role of urmylation in the oxidative stress response

0168	
YBR162C	[TOS1] Covalently-bound cell wall protein of unknown function; identified as a cell cycle regulated SBF target gene; deletion mutants are highly resistant to treatment with beta-1,3-glucanase; has sequence similarity to YJL171C
YOR233W	[KIN4] Kinase that acts by inhibiting the mitotic exit network (MEN) when the spindle position checkpoint is activated; localized asymmetrically to mother cell cortex, spindle pole body and bud neck protein kinase

0169	GO_TERM:[regulation of exit from mitosis] P-Value:2.1e-06
YDR226W	[ADK1] Adenylate kinase, required for purine metabolism; localized to the cytoplasm and the mitochondria; lacks cleavable signal sequence adenylate kinase
YFR028C	[CDC14] Protein phosphatase required for mitotic exit; located in the nucleolus until liberated by the FEAR and Mitotic Exit Network in anaphase, enabling it to act on key substrates to effect a decrease in CDK/B-cyclin activity and mitotic exit protein phosphatase Null mutant is inviable; ts mutant arrests at late anaphase with phenotypes similar to cdc5 mutants
YJR053W	[BFA1] Component of the GTPase-activating Bfa1p-Bub2p complex involved in multiple cell cycle checkpoint pathways that control exit from mitosis Null mutant is viable; mutants are sensitive to microtubule inhibitors, exhibit defects in mitotic checkpoints, and exhibit moderate defects in mating efficiency
YMR055C	[BUB2] Mitotic exit network regulator, forms GTPase-activating Bfa1p-Bub2p complex that binds Tem1p and spindle pole bodies, blocks cell cycle progression before anaphase in response to spindle and kinetochore damage Reduces the cell cycle delay which accompanies activation of a conditionally dicentric chromosome

0170	GO_TERM:[spindle] P-Value:1.0e-10 OVERLAP:[CCR4 complex] <510.190.110> SIZE:13
YGR052W	[YGR052W] The authentic, non-tagged protein was localized to the mitochondria
YML037C	
**YGR092W**	[DBF2] Ser/Thr kinase involved in transcription and stress response; functions as part of a network of genes in exit from mitosis; localization is cell cycle regulated; activated by Cdc15p during the exit from mitosis Ser/Thr Kinase Null mutant is viable, dbf1 dbf20 null mutants are inviable; mutants show dumb-bell phenotype
YPR111W	[DBF20] Ser/Thr kinase involved in late nuclear division, one of the mitotic exit network (MEN) proteins; necessary for the execution of cytokinesis Ser/Thr Kinase Dumbell formation
YDL028C	[MPS1] Dual-specificity kinase required for spindle pole body (SPB) duplication and spindle checkpoint function; substrates include SPB proteins Spc42p, Spc110p, and Spc98p, mitotic exit network protein Mob1p, and checkpoint protein Mad1p Null mutant is inviable. Eliminating the expression of MPS1 causes accumulation of non-viable cells with less than a 1 N DNA content. Allele-specific suppression and synthetic lethal interactions occur between mps1 and cdc37. Overexpression of Mps1p induces modification of Mad1p and arrests wild-type yeast cells in mitosis with morphologically normal spindles. mps1 does not arrest in the absence of spindle pole body duplication and monopolar spindle formation, or nocodozole treatment. Required for sporulation.
YAR019C	[CDC15] Protein kinase of the Mitotic Exit Network that is localized to the spindle pole bodies at late anaphase; promotes mitotic exit by directly switching on the kinase activity of Dbf2p protein kinase domain Null mutant inviable, arrests in G2; buds at distal instead of axial position, undergoes autolysis when buds reach the size of mother cells; the mitotic, but not meiotic, phenotype is suppressible by overexpressing SPO12.
**YIL106W**	[MOB1] Component of the mitotic exit network; associates with and is required for the activation and Cdc15p-dependent phosphorylation of the Dbf2p kinase; required for cytokinesis and cell separation; component of the CCR4 transcriptional complex Null mutant is inviable; conditional mutants arrest in late mitosis
YJR089W	[BIR1] Essential chromosomal passenger protein involved in coordinating cell cycle events for proper chromosome segregation; C-terminal region binds Sli15p, and the middle region, upon phosphorylation, localizes Cbf2p to the spindle at anaphase
YBR156C	[SLI15] Subunit of the Ipl1p-Sli15p-Bir1p complex that regulates kinetochore-microtubule attachments, activation of the spindle tension checkpoint, and mitotic spindle disassembly; regulates the activity and localization of the Ipl1p aurora kinase Null mutant is inviable; sli15 conditional mutations are sythentically lethal with ipl1-2 alleles.
YPL209C	[IPL1] Aurora kinase involved in regulating kinetochore-microtubule attachments, associates with Sli15p, which stimulates Ipl1p kinase activity and promotes its association with the mitotic spindle, potential Cdc28p substrate protein kinase temperature-sensitive mutant lacks proper chromosome segregation at non-permissive temperature

0171	GO_TERM:[ribose phosphate diphosphokinase activity] P-Value:4.5e-09
YER099C	[PRS2] 5-phospho-ribosyl-1(alpha)-pyrophosphate synthetase, involved in nucleotide, histidine, and tryptophan biosynthesis; one of a five related enzymes, which are active as heteromultimeric complexes ribose-phosphate pyrophosphokinase
YKL181W	[PRS1] 5-phospho-ribosyl-1(alpha)-pyrophosphate synthetase, involved in nucleotide, histidine, and tryptophan biosynthesis; one of five related enzymes, which are active as heteromultimeric complexes ribose-phosphate pyrophosphokinase
YMR139W	[RIM11] Protein kinase required for signal transduction during entry into meiosis; promotes the formation of the Ime1p-Ume6p complex by phosphorylating Ime1p and Ume6p; shares similarity with mammalian glycogen synthase kinase 3-beta Null mutant is viable; some alleles are Spo+ and sporulate slowly; rim11 is epistatic to the lethality of IME1 overexpression in haploids and permits Ime1p accumulation; RIM11 is a high copy suppressor of mck1 (cs) mutants
YOL061W	[PRS5] 5-phospho-ribosyl-1(alpha)-pyrophosphate synthetase, involved in nucleotide, histidine, and tryptophan biosynthesis; one of a five related enzymes, which are active as heteromultimeric complexes phosphoribosylpyrophosphate synthetase (ribose-phosphate pyrophosphokinase) Null mutant is viable but reduces the cellular 5-phosphoribosyl-1(alpha)-pyrophosphate synthetase activity by 84%. prs5 mutations are synthetically lethal with mutations in prs1 or prs3.

0172	GO_TERM:[cell organization and biogenesis] P-Value:2.3e-01
YGR270W	[YTA7] Protein of unknown function, member of CDC48/PAS1/SEC18 family of ATPases, potentially phosphorylated by Cdc28p
YPL115C	[BEM3] Rho GTPase activating protein (RhoGAP) involved in control of the cytoskeleton organization; targets the essential Rho-GTPase Cdc42p, which controls establishment and maintenance of cell polarity, including bud-site assembly rho GTPase activating protein (GAP)

0173	GO_TERM:[axial bud site selection] P-Value:9.2e-17 OVERLAP:[Septin filaments] <140.10.20> SIZE:7
YDR168W	[CDC37] Essential Hsp90p co-chaperone; necessary for passage through the START phase of the cell cycle Null mutant is inviable; temperature-sensitive mutants arrest in G1 and form shmoo morphology at the restrictive temperature
YKL048C	[ELM1] Serine/threonine protein kinase that regulates cellular morphogenesis, septin behavior, and cytokinesis; required for the regulation of other kinases; forms part of the bud neck ring protein kinase
YJR092W	[BUD4] Protein involved in bud-site selection and required for axial budding pattern; localizes with septins to bud neck in mitosis and may constitute an axial landmark for next round of budding; potential Cdc28p substrate Null mutant is viable, haploids have dipolar budding, normally they have axial budding, no effects on diploids
YPL153C	[RAD53] Protein kinase, required for cell-cycle arrest in response to DNA damage; activated by trans autophosphorylation when interacting with hyperphosphorylated Rad9p protein kinase Null mutant is inviable, radiation sensitive
YNL166C	[BNI5] Protein involved in organization of septins at the mother-bud neck, may interact directly with the Cdc11p septin, localizes to bud neck in a septin-dependent manner Null: Null mutant is viable, interacts genetically with CDC3, CDC10, CDC11, and CDC12 (septin) genes
YCL024W	[KCC4] Protein kinase of the bud neck involved in the septin checkpoint, associates with septin proteins, negatively regulates Swe1p by phosphorylation, shows structural homology to bud neck kinases Gin4p and Hsl1p S. pombe Nim1 homolog|protein kinase Null mutant is viable. Deletion of KCC4 causes moderate defects in bud formation at stationary phase; overexpression of KCC4 inhibits cell growth.
YDR507C	[GIN4] Protein kinase involved in bud growth and assembly of the septin ring, proposed to have kinase-dependent and kinase-independent activities; undergoes autophosphorylation; similar to Kcc4p and Hsl1p serine/threonine kinase (putative) Null mutant is viable, exhibits a mild elongated bud phenotype and some cell clumping
YNL078W	[NIS1] Protein localized in the bud neck at G2/M phase; physically interacts with septins; possibly involved in a mitotic signaling network
**YDL225W**	[SHS1] One of five related septins (Cdc3p, Cdc10p, Cdc11p, Cdc12p, Shs1p) that form a cortical filamentous collar at the mother-bud neck which is necessary for normal morphogenesis and cytokinesis septin deficient for cytokinesis
**YHR107C**	[CDC12] Component of the septin ring of the mother-bud neck that is required for cytokinesis; septins recruit proteins to the neck and can act as a barrier to diffusion at the membrane, and they comprise the 10nm filaments seen with EM 10 nm filament component of mother-bud neck|septin abnormal cell-wall deposition and bud growth, inability to complete cytokinesis, failure to form the ring of 10nm filaments in the neck region of budding cells
**YJR076C**	[CDC11] Component of the septin ring of the mother-bud neck that is required for cytokinesis; septins recruit proteins to the neck and can act as a barrier to diffusion at the membrane, and they comprise the 10nm filaments seen with EM 10 nm filament component of mother-bud neck|septin abnormal cell-wall deposition and bud growth, inability to complete cytokinesis, failure to form the ring of 10nm filaments in the neck region of budding cells
**YCR002C**	[CDC10] Component of the septin ring of the mother-bud neck that is required for cytokinesis; septins recruit proteins to the neck and can act as a barrier to diffusion at the membrane, and they comprise the 10nm filaments seen with EM septin abnormal cell-wall deposition and bud growth, inability to complete cytokinesis, failure to form the ring of 10nm filaments in the neck region of budding cells

YLR314C	[CDC3] Component of the septin ring of the mother-bud neck that is required for cytokinesis; septins recruit proteins to the neck and can act as a barrier to diffusion at the membrane, and they comprise the 10nm filaments seen with EM septin Null mutant is inviable; other mutants show abnormal cell-wall deposition and bud growth, inability to complete cytokinesis, and failure to form the ring of 10nm filaments in the neck region of budding cells.

0174	GO_TERM:[site of polarized growth] P-Value:1.2e-03
YBL085W	[BOI1] Protein implicated in polar growth, functionally redundant with Boi2p; interacts with bud-emergence protein Bem1p; contains an SH3 (src homology 3) domain and a PH (pleckstrin homology) domain
YHL007C	[STE20] Signal transducing kinase of the PAK (p21-activated kinase) family, involved in pheromone response and pseudohyphal/invasive growth pathways, activated by Cdc42p; binds Ste4p at a GBB motif present in noncatalytic domains of PAK kinases

0175	GO_TERM:[establishment of cell polarity] P-Value:1.7e-17
YKL082C	[RRP14] Essential protein, constituent of 66S pre-ribosomal particles; interacts with proteins involved in ribosomal biogenesis and cell polarity; member of the SURF-6 family
YDR309C	[GIC2] Protein of unknown function involved in initiation of budding and cellular polarization, interacts with Cdc42p via the Cdc42/Rac-interactive binding (CRIB) domain Null mutant is viable and temperature sensitive at 37 degrees C; gic1 gic2 double null is temperature sensitive at 33 degrees C
YHR061C	[GIC1] Protein of unknown function involved in initiation of budding and cellular polarization, interacts with Cdc42p via the Cdc42/Rac-interactive binding (CRIB) domain Null mutant is viable; gic1 gic2 double null is temperature sensitive at 33 degrees C
YOR127W	[RGA1] GTPase-activating protein for the polarity-establishment protein Cdc42p; implicated in control of septin organization, pheromone response, and haploid invasive growth rho GTPase activating protein (GAP) Null mutant is viable but shows increased signaling in the pheromone pathway; haploid null mutants bud predominantly in a bipolar, rather than the normal axial, manner
YPL161C	[BEM4] Protein involved in establishment of cell polarity and bud emergence; interacts with the Rho1p small GTP-binding protein and with the Rho-type GTPase Cdc42p; involved in maintenance of proper telomere length
YNL298W	[CLA4] Cdc42p activated signal transducing kinase of the PAK (p21-activated kinase) family, involved in septin ring assembly and cytokinesis; directly phosphorylates septins Cdc3p and Cdc10p; other yeast PAK family members are Ste20p and Skm1p Ste20p homolog|protein kinase Null mutant is viable, possesses a cytokinesis defect; cla4 cln1 cln2 strains are inviable; cla4 ste20 double deletion mutants cannot maintain septin rings at the bud neck and and cannot undergo cytokinesis
YER114C	[BOI2] Protein implicated in polar growth, functionally redundant with Boi1p; interacts with bud-emergence protein Bem1p; contains an SH3 (src homology 3) domain and a PH (pleckstrin homology) domain Null boi1 boi2 mutants become large round cells or lysed with buds, display defects in bud formation and in the maintenance of cell polarity
YGR152C	[RSR1] GTP-binding protein of the ras superfamily required for bud site selection, morphological changes in response to mating pheromone, and efficient cell fusion; localized to the plasma membrane; significantly similar to mammalian Rap GTPases random budding pattern
YAL041W	[CDC24] Guanine nucleotide exchange factor (GEF or GDP-release factor) for Cdc42p; required for polarity establishment and maintenance, and mutants have morphological defects in bud formation and shmooing guanine nucleotide exchange factor (a.k.a. GDP-release factor) for cdc42 temperature sensitive mutation affecting bud formation and localized cell surface growth at a restrictive temperature
YLR229C	[CDC42] Small rho-like GTPase, essential for establishment and maintenance of cell polarity; mutants have defects in the organization of actin and septins Rho subfamily of Ras-like proteins Null mutant is inviable; temperature sensitive mutations unable to form buds and display delocalized cell-surface deposition at the restrictive temperature

0176	GO_TERM:[protein kinase activity] P-Value:2.9e-03
YCL043C	[PDI1] Protein disulfide isomerase, multifunctional protein resident in the endoplasmic reticulum lumen, essential for the formation of disulfide bonds in secretory and cell-surface proteins, unscrambles non-native disulfide bonds protein disulfide isomerase
YMR304W	[UBP15] Ubiquitin-specific protease that may play a role in ubiquitin precursor processing
YGL179C	[TOS3] Protein kinase, related to and functionally redundant with Elm1p and Sak1p for the phosphorylation and activation of Snf1p; functionally orthologous to LKB1, a mammalian kinase associated with Peutz-Jeghers cancer-susceptibility syndrome
YPR115W	
YMR216C	[SKY1] SR protein kinase (SRPK) involved in regulating proteins involved in mRNA metabolism and cation homeostasis; similar to human SRPK1 Slow growth; Decreased in vivo phosphorylation of npl3p
YHL009C	[YAP3] Basic leucine zipper (bZIP) transcription factor

YPL141C	
YDR023W	[SES1] Cytosolic seryl-tRNA synthetase, class II aminoacyl-tRNA synthetase that aminoacylates tRNA(Ser), displays tRNA-dependent amino acid recognition which enhances discrimination of the serine substrate, interacts with peroxin Pex21p serine-tRNA ligase
YKR071C	[DRE2] Protein of unknown function; mutation displays synthetic lethal interaction with the pol3-13 allele of CDC2

0177	GO_TERM:[protein kinase activity] P-Value:1.0e-01
YER098W	[UBP9] Ubiquitin-specific protease that cleaves ubiquitin-protein fusions ubiquitin carboxyl-terminal hydrolase
YOR335C	[ALA1] Cytoplasmic alanyl-tRNA synthetase, required for protein synthesis; point mutation (cdc64-1 allele) causes cell cycle arrest at G1; lethality of null mutation is functionally complemented by human homolog null mutant is inviable; allele cdc64-1: arrest of proliferation at the regulatory step Start, inhibition of zygote formation and successful conjugation
YJR141W	
YDR134C	
YPL257W-A	
YML035C	[AMD1] AMP deaminase, tetrameric enzyme that catalyzes the deamination of AMP to form IMP and ammonia; may be involved in regulation of intracellular adenine nucleotide pools AMP deaminase
YMR115W	[YMR115W] The authentic, non-tagged protein was localized to the mitochondria
YOL125W	
YKL101W	[HSL1] Nim1p-related protein kinase that regulates the morphogenesis and septin checkpoints; associates with the assembled septin filament; required along with Hsl7p for bud neck recruitment, phosphorylation, and degradation of Swe1p serine-threonine kinase Null mutant is viable; synthetically lethal with histone H3 mutations; G2 delay
YNL307C	[MCK1] Protein serine/threonine/tyrosine (dual-specificity) kinase involved in control of chromosome segregation and in regulating entry into meiosis; related to mammalian glycogen synthase kinases of the GSK-3 family 43.1 kDa serine/threonine/tyrosine protein kinase

0178	
YDR049W	
YKL204W	[EAP1] eIF4E-associated protein, binds eIF4E and inhibits cap-dependent translation, also functions independently of eIF4E to maintain genetic stability; plays a role in cell growth, implicated in the TOR signaling cascade Mutant is temperature sensitive and partially resistant to rapamycin

0179	GO_TERM:[sporulation] P-Value:6.8e-03
YBR273C	[UBX7] UBX (ubiquitin regulatory X) domain-containing protein that interacts with Cdc48p
YJL048C	[UBX6] UBX (ubiquitin regulatory X) domain-containing protein that interacts with Cdc48p, transcription is repressed when cells are grown in media containing inositol and choline

0180	GO_TERM:[energy derivation by oxidation of organic compounds] P-Value:2.4e-02
YNL155W	
YBL058W	[SHP1] UBX (ubiquitin regulatory X) domain-containing protein that regulates Glc7p phosphatase activity and interacts with Cdc48p; interacts with ubiquitylated proteins in vivo and is required for degradation of a ubiquitylated model substrate Null mutant is viable; sporulation defective, slow growth; is deficient in glycogen accumulation; low Glc7p specific activity
YLR377C	[FBP1] Fructose-1,6-bisphosphatase, key regulatory enzyme in the gluconeogenesis pathway, required for glucose metabolism fructose-1,6-bisphosphatase unable to grow with ethanol

0181	GO_TERM:[cellular protein catabolism] P-Value:2.8e-18

YKL020C	[SPT23] ER membrane protein involved, with its homolog Mga2p, in regulation of OLE1 transcription; inactive ER form dimerizes and one subunit is then activated by ubiquitin/proteasome-dependent processing followed by nuclear targeting Null mutant does not have an Spt-phenotype. Disruption does not significantly affect cell growth or fatty acid metabolism.
YFL044C	[OTU1] Deubiquitylation enzyme that binds to the chaperone-ATPase Cdc48p; may contribute to regulation of protein degradation by deubiquitylating substrates that have been ubiquitylated by Ufd2p; member of the Ovarian Tumor (OTU) family De-ubiquitylation enzyme (DUB) of the OTU (ovarian tumor) family
YKL213C	[DOA1] WD repeat protein required for ubiquitin-mediated protein degradation, forms complex with Cdc48p, plays a role in controlling cellular ubiquitin concentration; also promotes efficient NHEJ in postdiauxic/stationary phase regulatory component of the proteasome pathway Null mutant is viable and defective in degradation of ubiquitinated proteins; homozygous null diploid shows sporulation defect
YDL190C	[UFD2] Ubiquitin chain assembly factor (E4) that cooperates with a ubiquitin-activating enzyme (E1), a ubiquitin-conjugating enzyme (E2), and a ubiquitin protein ligase (E3) to conjugate ubiquitin to substrates; also functions as an E3 ubiquitin conjugating factor e4 Null mutant is viable but exhibits increased sensitivity to ethanol stress.
YIL030C	[SSM4] Ubiquitin-protein ligase of the ER/nuclear envelope, required for degradation of Alpha2p and other proteins containing a Deg1 degradation signal; ssm4 mutation suppresses mRNA instability caused by an rna14 mutation integral membrane protein Null mutant is viable, suppresses temperature sensitive rna14 mutations; ssm4 sls1 mutants are inviable
YMR297W	[PRC1] Vacuolar carboxypeptidase Y (proteinase C), involved in protein degradation in the vacuole and required for full protein degradation during sporulation CPY|carboxypeptidase Y (proteinase C)|carboxypeptidase yscY Null mutant is viable,proteinase C deficient
YDR057W	[YOS9] Lectin; soluble lumenal ER protein; member of the OS-9 protein family; similar to mannose-6-phosphate receptors (MPRs); serves as a receptor that recognizes misfolded N-glycosylated proteins and participates in their targeting to ERAD membrane-associated glycoprotein Accelerates Gas1 transport and processing in cells overexpressing YOS9. Gas1 processing is slowed in cells bearing a deletion in YOS9.
YLR450W	[HMG2] One of two isozymes of HMG-CoA reductase that convert HMG-CoA to mevalonate, a rate-limiting step in sterol biosynthesis; overproduction induces assembly of peripheral ER membrane arrays and short nuclear-associated membrane stacks 3-hydroxy-3-methylglutaryl-coenzyme A (HMG-CoA) reductase isozyme Null mutant is viable, sensitive to compactin, a competitive inhibitor of HMG-CoA reductase; hmg1 hmg2 double deletion mutants are inviable
YLR207W	[HRD3] Resident protein of the ER membrane that plays a central role in ER-associated protein degradation (ERAD), forms HRD complex with Hrd1p and ERAD determinants that engages in lumen to cytosol communication and coordination of ERAD events Null mutant is viable, slows degradation of Hmg2p
YML029W	[USA1] Protein that interacts in the two-hybrid system with the U1 snRNP-specific protein, Snp1p; may have a role in pre-mRNA splicing pre-mRNA splicing factor (putative)
YOL013C	[HRD1] Ubiquitin-protein ligase required for endoplasmic reticulum-associated degradation (ERAD) of misfolded proteins; genetically linked to the unfolded protein response (UPR); regulated through association with Hrd3p; contains an H2 ring finger Null mutant is viable, slows degradation of Hmg2p
YBR201W	[DER1] Endoplasmic reticulum membrane protein, required for ER-associated protein degradation, involved in the retrograde transport of misfolded or unassembled proteins; N- and C- termini protrude into the cytoplasm, has similarity to Dfm1p Null mutant is viable, but blocks ER-degradation of target proteins
YDL126C	[CDC48] ATPase in ER, nuclear membrane and cytosol with homology to mammalian p97; in a complex with Npl4p and Ufd1p participates in retrotranslocation of ubiquitinated proteins from the ER into the cytosol for degradation by the proteasome
YML013W	[SEL1] UBX (ubiquitin regulatory X) domain-containing protein that interacts with Cdc48p, has a ubiquitin-associated (UBA) domain, interacts with ubiquitylated proteins in vivo, and is required for degradation of a ubiquitylated model substrate Null: enhanced secretion
YBR170C	[NPL4] Endoplasmic reticulum and nuclear membrane protein, forms a complex with Cdc48p and Ufd1p that recognizes ubiquitinated proteins in the endoplasmic reticulum and delivers them to the proteasome for degradation Temperature-sensitive mutants accumulate nuclear-targeted proteins in the cytoplasm and poly(A)+RNA in the nucleus and show defects in nuclear membrane integrity at the nonpermissive temperature
YGR048W	[UFD1] Protein that interacts with Cdc48p and Npl4p, involved in recognition of polyubiquitinated proteins and their presentation to the 26S proteasome for degradation; involved in transporting proteins from the ER to the cytosol Homozygous ufd1-1 mutant diploids exhibit sporulation defects. loss of Ufd1 blocks ER-associated protein degradation at a post-ubiquitination but pre-proteasome step.

0182	GO_TERM:[mitochondrial processing peptidase activity] P-Value:4.1e-06 OVERLAP:[Processing peptidase] <350.30> SIZE:2
YCL028W	[RNQ1] [PIN(+)] prion, an infectious protein conformation that is generally an ordered protein aggregate transferable epigenetic modifier, forms a prion responsible for the [PIN(+)] phenotype
YML058W	[SML1] Ribonucleotide reductase inhibitor involved in regulating dNTP production; regulated by Mec1p and Rad53p during DNA damage and S phase Null mutant is viable and suppresses mec1 and rad53 lethality; suppresses mip1-1 at 37 C, suppresses dun1 DNA damage sensitivity; increased resistance to DNA damage; increased dNTP pools
YIL066C	[RNR3] Ribonucleotide-diphosphate reductase (RNR), large subunit; the RNR complex catalyzes the rate-limiting step in dNTP synthesis and is regulated by DNA replication and DNA damage checkpoint pathways via localization of the small subunits ribonucleotide reductase, large (R1) subunit

YHR120W	[MSH1] DNA-binding protein of the mitochondria involved in repair of mitochondrial DNA, has ATPase activity and binds to DNA mismatches; has homology to E. coli MutS; transcription is induced during meiosis mutS homolog Null mutant is viable, petite
**YHR024C**	[MAS2] Larger subunit of the mitochondrial processing protease, essential processing enzyme that cleaves the N-terminal targeting sequences from mitochondrially imported proteins mitochondrial processing protease 53 kDa subunit
**YLR163C**	[MAS1] Smaller subunit of the mitochondrial processing protease, essential processing enzyme that cleaves the N-terminal targeting sequences from mitochondrially imported proteins mitochondrial processing protease subunit Null mutant is inviable; Elevated mitotic recombination and chromosomal missegregation when overproduced

0183	GO_TERM:[ribonucleoside-diphosphate reductase activity] P-Value:7.7e-09 OVERLAP:[Ribonucleoside-diphosphate reductase] <430> SIZE:4
**YER070W**	[RNR1] Ribonucleotide-diphosphate reductase (RNR), large subunit; the RNR complex catalyzes the rate-limiting step in dNTP synthesis and is regulated by DNA replication and DNA damage checkpoint pathways via localization of the small subunits ribonucleotide reductase, large (R1) subunit
YOR230W	[WTM1] Transcriptional repressor involved in regulation of meiosis and silencing; contains WD repeats transcriptional modulator
YOR229W	[WTM2] Transcriptional repressor involved in regulation of meiosis and silencing; contains WD repeats transcriptional modulator
**YGR180C**	[RNR4] Ribonucleotide-diphosphate reductase (RNR), small subunit; the RNR complex catalyzes the rate-limiting step in dNTP synthesis and is regulated by DNA replication and DNA damage checkpoint pathways via localization of the small subunits ribonucleotide reductase, small (R2) subunit Null mutant is inviable in the W303 strain background, but viable and sick in another (Wang et al.[1997] Mol. Cell Biol.17:6114-6121). An rnr4 mutant is resistant to 40 ug/ml benomyl, supersensitive to hydroxyurea (HU)[dead at 0.005M HU], and cold sensitive [cells arrest at 14 deg. C. with a large bud and short mitotic spindle].
**YJL026W**	[RNR2] Ribonucleotide-diphosphate reductase (RNR), small subunit; the RNR complex catalyzes the rate-limiting step in dNTP synthesis and is regulated by DNA replication and DNA damage checkpoint pathways via localization of the small subunits ribonucleotide reductase subunit|ribonucleotide reductase, small (R2) subunit

0184	GO_TERM:[chaperonin-containing T-complex] P-Value:2.4e-12 OVERLAP:[Chaperonine containing T-complex TRiC (TCP RING Complex)] <130> SIZE:8
YDR030C	[RAD28] Protein involved in transcription-coupled repair nucleotide excision repair of UV-induced DNA lesions; homolog of human CSA protein Null mutant is viable but is hypermutable following exposure to UV light and is slightly more sensitive to UV light in the presence of mutations in rad7 or rad16
**YDR212W**	[TCP1] Alpha subunit of chaperonin-containing T-complex, which mediates protein folding in the cytosol; involved in maintenance of actin cytoskeleton; homolog to Drosophila melanogaster and mouse tailless complex polypeptide chaperonin subunit alpha
YNL212W	[VID27] Cytoplasmic protein of unknown function; possibly involved in vacuolar protein degradation; not essential for proteasome-dependent degradation of fructose-1,6-bisphosphatase (FBPase); null mutants exhibit normal growth Null mutant is viable but exhibits vacuole degradation of cytosolic proteins
**YIL142W**	[CCT2] Subunit beta of the cytosolic chaperonin Cct ring complex, related to Tcp1p, required for the assembly of actin and tubulins in vivo
YOR281C	[PLP2] Essential protein with similarity to phosducins, which are GTPase inhibitors; lethality of null mutation is functionally complemented by expression of mouse phosducin-like protein MgcPhLP
**YDL143W**	[CCT4] Subunit of the cytosolic chaperonin Cct ring complex, related to Tcp1p, required for the assembly of actin and tubulins in vivo
**YDR188W**	[CCT6] Subunit of the cytosolic chaperonin Cct ring complex, related to Tcp1p, essential protein that is required for the assembly of actin and tubulins in vivo; contains an ATP-binding motif
**YJL111W**	[CCT7] Subunit of the cytosolic chaperonin Cct ring complex, related to Tcp1p, required for the assembly of actin and tubulins in vivo chaperonin containing T-complex subunit seven component

0185	GO_TERM:[clathrin coat] P-Value:2.2e-18 OVERLAP:[AP-1 complex] <260.20.10> SIZE:4
YDR320C	[SWA2] Auxilin-like protein involved in vesicular transport; clathrin-binding protein required for uncoating of clathrin-coated vesicles auxilin-like protein Null mutant is viable but exhibits endocytosis and late Golgi defects.
YNR006W	[VPS27] Endosomal protein that forms a complex with Hse1p; required for recycling Golgi proteins, forming lumenal membranes and sorting ubiquitinated proteins destined for degradation; has Ubiquitin Interaction Motifs which bind ubiquitin (Ubi4p) cysteine rich putative zinc finger essential for function|hydrophilic protein required for membrane traffic to the vacuole
YGR167W	[CLC1] Clathrin light chain, subunit of the major coat protein involved in intracellular protein transport and endocytosis; thought to regulate clathrin function, two Clathrin heavy chains (CHC1) form the clathrin triskelion structural component clathrin light chain Null mutant is viable but slow-growing and shows defects in receptor-mediated endocytosis, maturation of alpha factor and levels of clathrin heavy chain (Chc1p); high copy suppresses the inviable double mutant chc1-delete, scd1-i-allele; elevated CHC1 expression suppresses some clc1-delete phenotypes

YHR108W	[GGA2] Golgi-localized protein with homology to gamma-adaptin, interacts with and regulates Arf1p and Arf2p in a GTP-dependent manner in order to facilitate traffic through the late Golgi ARF-binding protein Single and double knockouts are viable at both 30 C and 37 C. Cells lacking GGA1, GGA2 exhibit defects in invertase processing, vacuolar morphology, maturation of alpha-factor, and sorting of CPY, proteinase A to the vacuole, but not endocytosis.
YDR153C	[ENT5] Protein containing an N-terminal epsin-like domain involved in clathrin recruitment and traffic between the Golgi and endosomes; associates with the clathrin adaptor Gga2p, clathrin adaptor complex AP-1, and clathrin
YGL206C	[CHC1] Clathrin heavy chain, subunit of the major coat protein involved in intracellular protein transport and endocytosis; two heavy chains form the clathrin triskelion structural component; the light chain (CLC1) is thought to regulate function Clathrin heavy chain Null mutant is viable, but is slow-growing and shows defects in mating, sporulation and vesicle ultrastructure (however it shows little or no defect in secretion); null mutants easily become inviable due to second site mutations in a number of unlinked genes such as SCD1 and CDL1. Null mutants also exhibit an endocytosis defect, late Golgi protein mislocalization. chc1-5 exhibits delayed vacuolar protein transport.
YJR125C	[ENT3] Protein containing an N-terminal epsin-like domain involved in clathrin recruitment and traffic between the Golgi and endosomes; associates with the clathrin adaptor Gga2p
YJL207C	[YJL207C] Protein of unknown function, proposed to function as a large AP-1 accessory protein; colocalizes with clathrin to the late-Golgi apparatus; YJL207C is a non-essential gene
YFR043C	[YFR043C] Hypothetical protein; null mutant displays increased levels of spontaneous Rad52 foci
YBR019C	[GAL10] UDP-glucose-4-epimerase, catalyzes the interconversion of UDP-galactose and UDP-D-glucose in galactose metabolism; also catalyzes the conversion of alpha-D-glucose or alpha-D-galactose to their beta-anomers UDP-glucose 4-epimerase Null mutant is viable and cannot utilize galactose.
**YPR029C**	[APL4] Gamma-adaptin, large subunit of the clathrin-associated protein (AP-1) complex; binds clathrin; involved in vesicle mediated transport clathrin associated protein complex large subunit|gamma-adaptin
**YKL135C**	[APL2] Beta-adaptin, large subunit of the clathrin-associated protein (AP-1) complex; binds clathrin; involved in clathrin-dependent Golgi protein sorting beta-adaptin|clathrin associated protein complex large subunit
YFL034W	
YHL019C	[APM2] Protein of unknown function, homologous to the medium chain of mammalian clathrin-associated protein complex; involved in vesicular transport
**YLR170C**	[APS1] Small subunit of the clathrin-associated adaptor complex AP-1, which is involved in protein sorting at the trans-Golgi network; homolog of the sigma subunit of the mammalian clathrin AP-1 complex clathrin associated protein complex small subunit Null mutant is viable; aps1 mutants demonstrate synthetic effects with chc1 alleles

0186	GO_TERM:[late endosome to vacuole transport] P-Value:3.7e-08 OVERLAP:[Vps4p ATPase complex (Vps protein complex)] <260.70> SIZE:3
**YLR025W**	[SNF7] One of four subunits of the endosomal sorting complex required for transport III (ESCRT-III); involved in the sorting of transmembrane proteins into the multivesicular body (MVB) pathway; recruited from the cytoplasm to endosomal membranes
YDR069C	[DOA4] Ubiquitin hydrolase, required for recycling ubiquitin from proteasome-bound ubiquitinated intermediates, acts at the late endosome/prevacuolar compartment to recover ubiquitin from ubiquitinated membrane proteins en route to the vacuole ubiquitin isopeptidase Null mutant is viable, but exhibits uncoordinated DNA replication|A nonsense mutation in the doa4-10 mutant eliminates the catalytic residues of the deubiquitinating enzyme while keeping the rhodanase domain intact. At 36 degrees C, this doa4-10 mutant exhibits increased sensitivity to camptothecin (CPT), osmotic stress, and hydroxyurea, and a reversible petite phenotype.
YPL084W	[BRO1] Cytoplasmic class E vacuolar protein sorting (VPS) factor that coordinates deubiquitination in the multivesicular body (MVB) pathway by recruiting Doa4p to endosomes
YOR275C	[RIM20] Protein involved in proteolytic activation of Rim101p in response to alkaline pH; member of the PalA/AIP1/Alix family; interacts with the ESCRT-III subunits Snf7p, suggesting a relationship between the response to pH and multivesicular body formation Null: Affected in sporulation and invasive growth. Other phenotypes: Alkaline sensitivity
**YPR173C**	[VPS4] AAA-type ATPase required for efficient late endosome to vacuole transport; catalyzes the release of an endosomal membrane-associated class E VPS protein complex; cytoplasmic protein that is also associated with an endosomal compartment AAA ATPase Null mutant is viable, exhibits protein sorting and morphological defects
YKR035W-A	[DID2] Class E protein of the vacuolar protein-sorting (Vps) pathway, associates reversibly with the late endosome, has human ortholog that may be altered in breast tumors class E vacuolar-protein sorting and endocytosis factor Overexpression causes growth inhibition and G2 arrest in rad52 and cdc9 mutants; null mutants are canavanine-hypersensitive, temperature sensitive, and suppress defects associated with loss of DOA4
YLR181C	[VTA1] Multivesicular body (MVB) protein involved in endosomal protein sorting; binds to Vps20p and Vps4p; may regulate Vps4p function; bindsVps60p and may act at a late step in MVB formation; mutants show class E vacuolar-protein sorting defects Null: Class E defect in vacuolar protein sorting (accumulates FM4-64, Ste3, Vph1, Pep12 in the class E compartment)

0187	GO_TERM:[endosome membrane] P-Value:2.6e-22 OVERLAP:[Vps4p ATPase complex (Vps protein complex)] <260.70> SIZE:3

YCL008C	[STP22] Component of the ESCRT-I complex, which is involved in ubiquitin-dependent sorting of proteins into the endosome; homologous to the mouse and human Tsg101 tumor susceptibility gene; mutants exhibit a Class E Vps phenotype putative ubiquitin receptor
YGR206W	
YCR065W	[HCM1] Forkhead transcription factor involved in cell cycle specific transcription of SPC110; dosage-dependent suppressor of calmodulin mutants with specific defects in SPB assembly; involved in telomere maintenance forkhead protein Null mutant is viable; exacerbates temperature-sensitivity of a cmd1-1 (calmodulin) mutant
**YKL041W**	[VPS24] One of four subunits of the endosomal sorting complex required for transport III (ESCRT-III); forms an ESCRT-III subcomplex with Did4p; involved in the sorting of transmembrane proteins into the multivesicular body (MVB) pathway
YLR119W	[SRN2] Component of the ESCRT-I complex, which is involved in ubiquitin-dependent sorting of proteins into the endosome; suppressor of rna1-1 mutation; may be involved in RNA export from nucleus
YMR077C	[VPS20] Myristoylated subunit of ESCRTIII, the endosomal sorting complex required for transport of transmembrane proteins into the multivesicular body pathway to the lysosomal/vacuolar lumen; cytoplasmic protein recruited to endosomal membranes
YPL065W	[VPS28] Component of the ESCRT-I complex, which is involved in ubiquitin-dependent sorting of proteins into the endosome; involved in transport of precursors for soluble vacuolar hydrolases from the late endosome to the vacuole Null mutant is viable, shows moderate defects in both biosynthetic traffic and endocytic traffic destined for the vacuole
YPL002C	[SNF8] Component of the ESCRT-II complex, which is involved in ubiquitin-dependent sorting of proteins into the endosome; appears to be functionally related to SNF7; involved in glucose derepression
YJR102C	[VPS25] Component of the ESCRT-II complex, which is involved in ubiquitin-dependent sorting of proteins into the endosome Null mutant is viable but a class E vps mutant (missorts vacuolar hydrolases and accumulates late endosomal compartment vacuolar hydrolases and accumulates a late endosomal compartment).
YLR417W	[VPS36] Component of the ESCRT-II complex, which is involved in ubiquitin-dependent sorting of proteins into the endosome

0188	GO_TERM:[guanyl-nucleotide exchange factor complex] P-Value:5.1e-05
YGR209C	[TRX2] Cytoplasmic thioredoxin isoenzyme of the thioredoxin system which protects cells against both oxidative and reductive stress, forms LMA1 complex with Pbi2p, acts as a cofactor for Tsa1p, required for ER-Golgi transport and vacuole inheritance EC 1.8.4.8|thioredoxin Null mutant is viable; trx1-trx2 double mutant shows prolonged S phase, shortened G(sub)1 and methionine auxotrophy
YDR137W	[RGP1] Subunit of a Golgi membrane exchange factor (Ric1p-Rgp1p) that catalyzes nucleotide exchange on Ypt6p reduced growth
YLR039C	[RIC1] Protein involved in retrograde transport to the cis-Golgi network; forms heterodimer with Rgp1p that acts as a GTP exchange factor for Ypt6p; involved in transcription of rRNA and ribosomal protein genes defective in the transcription of both ribosomal protein genes and ribosomal RNA

0189	GO_TERM:[double-strand break repair via nonhomologous end joining] P-Value:2.7e-03 OVERLAP:[DNA ligase IV] <510.180.30.20> SIZE:2
YGR241C	[YAP1802] Protein involved in clathrin cage assembly; binds Pan1p and clathrin; homologous to Yap1801p, member of the AP180 protein family
YNL084C	[END3] EH domain-containing protein involved in endocytosis, actin cytoskeletal organization and cell wall morphogenesis; forms a complex with Sla1p and Pan1p Null mutant is viable and defective in endocytosis
YDR369C	[XRS2] Protein required for DNA repair; component of the Mre11 complex, which is involved in double strand breaks, meiotic recombination, telomere maintenance, and checkpoint signaling DNA repair protein X-ray sensitive, spores inviable, xrs2 is rescued by spo13 and is epistatic to rad52
**YGL090W**	[LIF1] Protein involved in DNA double-strand break repair; physically interacts with DNA ligase 4 (Lig4p); homologous to mammalian XRCC4 protein Null mutant is viable but is deficient in non-homologous double-strand break repair; inefficient in sporulation; LIG4 protein destabilization
YLR109W	[AHP1] Thiol-specific peroxiredoxin, reduces hydroperoxides to protect against oxidative damage; function in vivo requires covalent conjugation to Urm1p alkyl hydroperoxide reductase hypersensitive to tert-butyl hydroperoxide

0190	GO_TERM:[actin cytoskeleton] P-Value:6.3e-26 OVERLAP:[Arp2p/Arp3p complex] <260.90> SIZE:6
YIL159W	[BNR1] Formin, nucleates the formation of linear actin filaments, involved in cell processes such as budding and mitotic spindle orientation which require the formation of polarized actin cables, functionally redundant with BNI1 Null mutant is viable; bni1 bnr1 double mutant exhibits severe temperature sensitive growth
YBR260C	[RGD1] GTPase-activating protein (RhoGAP) for Rho3p and Rho4p, possibly involved in control of actin cytoskeleton organization GTPase activating protein (GAP) (putative) Null mutant is viable and exhibits slightly decreased viability during late exponential and stationary phase in minimal medium
YMR032W	[HOF1] Bud neck-localized, SH3 domain-containing protein required for cytokinesis; regulates actomyosin ring dynamics and septin localization; interacts with the formins, Bni1p and Bnr1p, and with Cyk3p, Vrp1p, and Bni5p Null mutant is defective in cytokinesis

YJL020C	[BBC1] Protein possibly involved in assembly of actin patches; interacts with an actin assembly factor Las17p and with the SH3 domains of Type I myosins Myo3p and Myo5p; localized predominantly to cortical actin patches
YLR337C	[VRP1] Proline-rich actin-associated protein involved in cytoskeletal organization and cytokinesis; related to mammalian Wiskott-Aldrich syndrome protein (WASP)-interacting protein (WIP) proline-rich protein verprolin Null mutant is viable but is both temperature and pH sensitive and cannot grow on minimal medium. Null mutant also exhibits depolarization of the actin cytoskeleton.
YKR069W	[MET1] S-adenosyl-L-methionine uroporphyrinogen III transmethylase, involved in sulfate assimilation, methionine metabolism, and siroheme biosynthesis Null mutant is viable, and is a methionine auxotroph
YOR247W	[SRL1] Mannoprotein that exhibits a tight association with the cell wall, required for cell wall stability in the absence of GPI-anchored mannoproteins; has a high serine-threonine content; expression is induced in cell wall mutants
YGL242C	
YIL156W	[UBP7] Ubiquitin-specific protease that cleaves ubiquitin-protein fusions ubiquitin-specific protease
YIR006C	[PAN1] Part of actin cytoskeleton-regulatory complex Pan1p-Sla1p-End3p, associates with actin patches on the cell cortex; promotes protein-protein interactions essential for endocytosis; previously thought to be a subunit of poly(A) ribonuclease Null mutant is inviable; conditional mutants show arrest of translation initiation, alterations in mRNA poly(A) tail lengths, and altered cellular location of Mod5p
YKL129C	[MYO3] One of two type I myosins; localizes to actin cortical patches; deletion of MYO3 has little effect on growth, but myo3 myo5 double deletion causes severe defects in growth and actin cytoskeleton organization myosin I Null mutant is viable, myo3 myo5 double deletion mutants exhibit severe defects in growth and actin cytoskeletal organization
YMR109W	[MYO5] One of two type I myosins; contains proline-rich tail homology 2 (TH2) and SH3 domains; MYO5 deletion has little effect on growth, but myo3 myo5 double deletion causes severe defects in growth and actin cytoskeleton organization myosin I Null mutant is viable; myo3 myo5 double deletion mutants exhibit loss of actin polarity, growth arrest at 37 degrees or high osmotic strength, accumulation of intracellular membranes, and loss of polarized cell surface growth; myo3 myo5 double mutants have longer doubling times and thicker cell walls
**YDL029W**	[ARP2] Essential component of the Arp2/3 complex, which is a highly conserved actin nucleation center required for the motility and integrity of actin patches; involved in endocytosis and membrane growth and polarity actin related protein cells with mutations in Arp2 and Arc15 are defective in mitochondrial movement.
**YJR065C**	[ARP3] Essential component of the Arp2/3 complex, which is a highly conserved actin nucleation center required for the motility and integrity of actin patches; involved in endocytosis and membrane growth and polarity Mutations in Arp3 lead to defects in actin-patch motility and a rearrangement of the cortical actin cytoskeleton.
YBR234C	[ARC40] Essential subunit of the ARP2/3 complex, which is required for the motility and integrity of cortical actin patches
**YNR035C**	[ARC35] Subunit of the ARP2/3 complex, which is required for the motility and integrity of cortical actin patches; required for cortical localization of calmodulin Null mutant exhibits severe growth defects; synthetic lethal with vma2.
**YKL013C**	[ARC19] Subunit of the ARP2/3 complex, which is required for the motility and integrity of cortical actin patches Null mutant is viable, but exhibits severe growth defects
**YIL062C**	[ARC15] Subunit of the ARP2/3 complex, which is required for the motility and integrity of cortical actin patches Null mutant exhibits severe growth defects. Cells with mutations in Arp2 and Arc15 are defective in mitochondrial movement.
**YLR370C**	[ARC18] Subunit of the ARP2/3 complex, which is required for the motility and integrity of cortical actin patches
0191	GO_TERM:[incipient bud site] P-Value:2.2e-03
YPR154W	[PIN3] Protein that induces appearance of [PIN+] prion when overproduced Other phenotypes: overexpression of PIN3 allows for the induction of the [PSI+] prion in strains cured of [PIN+].
YMR192W	[GYL1] Putative GTPase activating protein (GAP) that may have a role in polarized exocytosis; stimulates Gyp5p GAP activity on Ypt1p, colocalizes with Gyp5p at sites of polarized growth; interacts with Gyp5p, Rvs161p, and Rvs167p
YPL249C	[GYP5] GTPase-activating protein (GAP) for yeast Rab family members, involved in ER to Golgi trafficking; exhibits GAP activity toward Ypt1p that is stimulated by Gyl1p, also acts on Sec4p; interacts with Gyl1p, Rvs161p and Rvs167p GTPase-activating protein
0192	GO_TERM:[ubiquitin cycle] P-Value:1.2e-05
YGL144C	[ROG1] Protein with putative serine active lipase domain
YMR275C	[BUL1] Ubiquitin-binding component of the Rsp5p E3-ubiquitin ligase complex, functional homolog of Bul2p, disruption causes temperature-sensitive growth, overexpression causes missorting of amino acid permeases
YER125W	[RSP5] Ubiquitin-protein ligase involved in ubiquitin-mediated protein degradation; plays a role in heat shock element (HSE)-mediated gene expression and multivesicular body sorting; contains a hect (homologous to E6-AP carboxyl terminus) domain Null mutant is inviable; an rsp5 mutation was isolated as a suppressor of mutations in SPT3; certain rsp5 mutants also exhibit hypersensitivity to stresses such as cadmium and canavanine, and sporulation defects

YHL002W	[HSE1] Subunit of the endosomal Vps27p-Hse1p complex required for sorting of ubiquitinated membrane proteins into intralumenal vesicles prior to vacuolar degradation, as well as for recycling of Golgi proteins and formation of lumenal membranes Null: accumulates enlarged prevacuolar/endosomal compartment. Fails to sort proteins into the vacuolar lumen.. Other phenotypes: secretes CPY
YGR268C	[HUA1] Cytoplasmic protein containing a zinc finger domain with sequence similarity to that of Type I J-proteins; computational analysis of large-scale protein-protein interaction data suggests a possible role in actin patch assembly
YOR124C	[UBP2] Ubiquitin-specific protease that removes ubiquitin from ubiquitinated proteins, cleaves at the C terminus of ubiquitin fusions; capable of cleaving polyubiquitin and possesses isopeptidase activity ubiquitin-specific protease Null mutant is viable. Null yuh1 ubp1 ubp2 ubp3 quadruple mutants are viable and retain the ability to deubiquitinate ubiquitin fusions.
YOR138C	[RUP1] Protein involved in regulation of Rsp5p, which is an essential HECT ubiquitin ligase; required for binding of Rsp5p to Ubp2p; contains an UBA domain

0193	GO_TERM:[phosphoinositide metabolism] P-Value:3.2e-03
YNL106C	[INP52] Phosphatidylinositol 4,5-bisphosphate 5-phosphatase, synaptojanin-like protein with an N-terminal Sac1 domain, plays a role in endocytosis; hyperosmotic stress causes translocation to actin patches inositol polyphosphate 5-phosphatase Null mutant is viable, has abnormal vacuoles
YPR171W	[BSP1] Adapter that links synaptojanins Inp2p and Inp53p to the cortical actin cytoskeleton
YHR076W	[PTC7] Mitochondrially localized type 2C protein phosphatase; expression induced by growth on ethanol and by sustained osmotic stress; possible role in carbon source utilization in low oxygen environments type 2C Protein Phosphatase
YMR079W	[SEC14] Phosphatidylinositol/phosphatidylcholine transfer protein involved in coordinate regulation of PtdIns and PtdCho metabolism, products of which are regulators in Golgi to plasma membrane transport; functionally homologous to mammalian PITPs phosphatidylcholine transfer protein|phosphatidylinositol transfer protein Null mutant is inviable; other mutations are temperature sensitive

0194	GO_TERM:[actin cytoskeleton organization and biogenesis] P-Value:2.5e-14 OVERLAP:[Actin-associated proteins] <140.20.20> SIZE:25
YFL012W	
YPL111W	[CAR1] Arginase, responsible for arginine degradation, expression responds to both induction by arginine and nitrogen catabolite repression; disruption enhances freeze tolerance arginase Null mutant is viable but defective in arginine catabolism
YDR523C	[SPS1] Putative protein serine/threonine kinase expressed at the end of meiosis and localized to the prospore membrane, required for correct localization of enzymes involved in spore wall synthesis
YLR243W	
YOR367W	[SCP1] Component of yeast cortical actin cytoskeleton, binds and cross links actin filaments; originally identified by its homology to calponin (contains a calponin-like repeat) but the Scp1p domain structure is more similar to transgelin calponin homolog Null mutant is viable and shows no apparent phenotype
YJR083C	[ACF4] Protein of unknown function, computational analysis of large-scale protein-protein interaction data suggests a possible role in actin cytoskeleton organization; potential Cdc28p substrate
YNL020C	[ARK1] Serine/threonine protein kinase involved in regulation of the cortical actin cytoskeleton; involved in control of endocytosis serine/threonine kinase (putative) Null mutant is viable and shows slight delocalisation of actin cytoskeleton
YHR079C	[IRE1] Serine-threonine kinase and endoribonuclease; transmembrane protein that initiates the unfolded protein response signal by regulating synthesis of Hac1p through HAC1 mRNA splicing endoribonuclease|serine-threonine kinase|transmembrane protein Null mutant is viable, myo-inositol auxotroph; IRE1 is essential for viability under stress conditions that cause unfolded proteins to accumulate in the ER
YMR253C	
YLR064W	
YBR098W	[MMS4] Subunit of the structure-specific Mms4p-Mus81p endonuclease that cleaves branched DNA; involved in recombination and DNA repair null is synthetically lethal with sgs1 null
YBR108W	
YBR239C	
YHR114W	[BZZ1] SH3 domain protein implicated in the regulation of actin polymerization, able to recruit actin polymerization machinery through its SH3 domains, colocalizes with cortical actin patches and Las17p, interacts with type I myosins
**YCR088W**	[ABP1] Actin-binding protein of the cortical actin cytoskeleton, important for activation of the Arp2/3 complex that plays a key role actin in cytoskeleton organization actin binding protein

**YOR181W**	[LAS17] Actin assembly factor, activates the Arp2/3 protein complex that nucleates branched actin filaments; localizes with the Arp2/3 complex to actin patches; homolog of the human Wiskott-Aldrich syndrome protein (WASP) actin assembly factor Null mutant is viable, demonstrates impaired budding and cytokenesis and severely disrupted cortical actin; other mutants accumulate secretory vesicles in the bud
YJL151C	[SNA3] Integral membrane protein localized to vacuolar intralumenal vesicles, computational analysis of large-scale protein-protein interaction data suggests a possible role in either cell wall synthesis or protein-vacuolar targeting Null mutant is viable, excretes inositol
YKR030W	[GMH1] Golgi membrane protein of unknown function, interacts with Gea1p and Gea2p; required for localization of Gea2p; computational analysis suggests a possible role in either cell wall synthesis or protein-vacuolar targeting
YPL246C	[RBD2] Possible rhomboid protease, has similarity to eukaryotic rhomboid proteases including Pcp1p rhomboid protease
**YDR388W**	[RVS167] Actin-associated protein, subunit of a complex (Rvs161p-Rvs167p) involved in regulation of actin cytoskeleton, endocytosis, and viability following starvation or osmotic stress; homolog of mammalian amphiphysin cytoskeletal protein (putative) Null mutant is viable but exhibits reduced viability upon starvation
YNL094W	[APP1] Protein of unknown function, interacts with Rvs161p and Rvs167p; computational analysis of protein-protein interactions in large-scale studies suggests a possible role in actin filament organization Null: Viable. Other phenotypes: Unknown
**YNL243W**	[SLA2] Transmembrane actin-binding protein involved in membrane cytoskeleton assembly and cell polarization; adaptor protein that links actin to clathrin and endocytosis; present in the actin cortical patch of the emerging bud tip; dimer in vivo transmembrane protein Null mutant is viable and temperature sensitive
YOR284W	[HUA2] Cytoplasmic protein of unknown function; computational analysis of large-scale protein-protein interaction data suggests a possible role in actin patch assembly
**YBL007C**	[SLA1] Cytoskeletal protein binding protein required for assembly of the cortical actin cytoskeleton; contains 3 SH3 domains; interacts with proteins regulating actin dynamics and with proteins required for endocytosis cytoskeletal protein binding protein Null mutant is viable, temperature sensitive. sla1 mutants are synthetically lethal in combination with anc1 and abp1 mutants
YFR024C-A	[LSB3] Protein containing a C-terminal SH3 domain; binds Las17p, which is a homolog of human Wiskott-Aldrich Syndrome protein involved in actin patch assembly and actin polymerization
YHR016C	[YSC84] Protein involved in the organization of the actin cytoskeleton; contains SH3 domain similar to Rvs167p

0195	OVERLAP:[Pho85p complexes] <133.20> SIZE:6
YKR094C	[RPL40B] Fusion protein, identical to Rpl40Ap, that is cleaved to yield ubiquitin and a ribosomal protein of the large (60S) ribosomal subunit with similarity to rat L40; ubiquitin may facilitate assembly of the ribosomal protein into ribosomes also encodes a ubiquitin protein|ribosomal protein L40B
YKR018C	
**YDL127W**	[PCL2] G1 cyclin, associates with Pho85p cyclin-dependent kinase (Cdk) to contribute to entry into the mitotic cell cycle, essential for cell morphogenesis; localizes to sites of polarized cell growth G1 cyclin
YPL133C	[RDS2] Zinc cluster protein involved in conferring resistance to ketoconazole transcriptional regulator Null: sensitive to ketoconazole. Other phenotypes: inability to grow on non-fermentable carbon sources, sensitivity to calcofluor white|Null mutant is viable; cannot utilize glycerol and lactate as sole carbon source; hypersensitive to calcofluor white

0196	GO_TERM:[magnesium ion transport] P-Value:4.6e-05
YFL050C	[ALR2] Probable Mg(2+) transporter; overexpression confers increased tolerance to Al(3+) and Ga(3+) ions Null mutant is viable, overexpression increases resistance to aluminum and gallium toxicity
YOL130W	[ALR1] Plasma membrane Mg(2+) transporter, expression and turnover are regulated by Mg(2+) concentration; overexpression confers increased tolerance to Al(3+) and Ga(3+) ions Null mutant is inviable; overexpression increases resistance to aluminum and gallium toxicity
YGL180W	[ATG1] Protein serine/threonine kinase, required for autophagy and for the cytoplasm-to-vacuole targeting (Cvt) pathway protein kinase Defective in autophagy; loses viability more rapidly than wild type during nitrogen starvation; defective in vacuolar protein degradation during nitrogen starvation; defective in sporulation
YDL025C	
YHR009C	

0197	GO_TERM:[glutathione metabolism] P-Value:5.8e-05
YDR120C	[TRM1] tRNA methyltransferase, localizes to both the nucleus and mitochondrion to produce the modified base N2,N2-dimethylguanosine in tRNAs in both compartments N2,N2-dimethylguanosine-specific tRNA methyltransferase An uncharacterized allele affects a specific base modification of both cytoplasmic and mitochondrial tRNA.

YGR121C	[MEP1] Ammonium permease; belongs to a ubiquitous family of cytoplasmic membrane proteins that transport only ammonium (NH4+); expression is under the nitrogen catabolite repression regulation ammonia permease
YEL017W	[GTT3] Protein of unknown function with a possible role in glutathione metabolism, as suggested by computational analysis of large-scale protein-protein interaction data; GFP-fusion protein localizes to the nuclear periphery
YIR038C	[GTT1] ER associated glutathione S-transferase capable of homodimerization; expression induced during the diauxic shift and throughout stationary phase; functional overlap with Gtt2p, Grx1p, and Grx2p glutathione transferase heat shock sensitive at stationary phase

0198	GO_TERM:[response to DNA damage stimulus] P-Value:1.8e-10 OVERLAP:[MSH2/MSH3 complex] <510.180.50.10> SIZE:3
YPL164C	[MLH3] Protein involved in DNA mismatch repair; forms a complex with Mlh1p to promote meiotic crossing-over; mammalian homolog is implicated mammalian microsatellite instability Null mutant is viable. Null mutant exhibits reduced (70%) rate of meiotic cross over.
YMR190C	[SGS1] Nucleolar DNA helicase of the RecQ family involved in maintenance of genome integrity, regulates chromosome synapsis and meiotic crossing over; has similarity to human BLM and WRN helicases implicated in Bloom and Werner syndromes Null mutant is viable; strains lacking SGS1 exhibit elevated levels of chromosome misseggregation during both mitotic and meiotic division. sgs1 null strains suppress the slow growth of a top3 delta strain lacking topoisomerase III and show an increase in subtelomeric Y' instability due to hyperrecombination.
YLR234W	[TOP3] DNA Topoisomerase III, conserved protein that functions in a complex with Sgs1p and Rmi1p to relax single-stranded negatively-supercoiled DNA preferentially, involved in telomere stability and regulation of mitotic recombination DNA topoisomerase III Null mutant exhibits a genomic instability phenotype that includes slow growth, hyper-sensitivity to genotoxic agents, mitotic hyper-recombination, increased chromosome missegregation, and meiotic failure. top3 is RAD1-dependent hyper-Rec in mitosis, suggesting that top3 damage is channeled to the recombination repair pathway by RAD1; TOP3 is required for sporulation.
YPL024W	[RMI1] Involved in response to DNA damage; null mutants have increased rates of recombination and delayed S phase; interacts physically and genetically with Sgs1p (RecQ family member) and Top3p (topoisomerase III) Null mutant is viable and suppresses the failure of an ace2 null to activate CTS1; also grows slowly at 37 C
YLR035C	[MLH2] Protein required for DNA mismatch repair in mitosis and meiosis; involved in the repair of mutational intermediates; forms a complex with Pms1p and Msh1p to repair mismatched DNA Null mutant is viable but non-Mendelian segregation is elevated and parity is altered during meiosis.
**YNL082W**	[PMS1] ATP-binding protein required for mismatch repair in mitosis and meiosis; functions as a heterodimer with Mlh1p, binds double- and single-stranded DNA via its N-terminal domain, similar to E. coli MutL mutL homolog|similar to Mlh1p; associates with Mlh1p, forming a heterodimer that then forms a ternary complex with either Msh2p-Msh3p or Msh2p-Msh6p bound to mismatched DNA Null mutant is viable; postmeiotic segregation increased
**YOL090W**	[MSH2] Protein that forms heterodimers with Msh3p and Msh6p that bind to DNA mismatches to initiate the mismatch repair process; contains a Walker ATP-binding motif required for repair activity; Msh2p-Msh6p binds to and hydrolyzes ATP mutS homolog Haploid mutants display 85-fold increased rate of spontaneous mutation to canavanine resistance. Mutants are defective for gene conversion polarity gradients and high spore viability. Inactivation of MSH2 causes high rates of accumulation of both base-substitution and frameshift mutations.
YMR167W	[MLH1] Protein required for mismatch repair in mitosis and meiosis, postmeiotic segregation, and spore viability; forms a complex with Pms1p and Msh2p to repair mismatched DNA; human homolog is associated with hereditary non-polyposis colon cancer mutL homolog Null mutant is viable; displays a dramatic increase in the instability of simple sequence repeats, disruption of the MLH1 gene in diploid strains causes increased spore lethality; mlh1 delta pms1 delta double mutant are indistinguishable from those of the mlh1 delta and pms1 delta single mutants
YOR033C	[EXO1] 5'-3' exonuclease and flap-endonuclease involved in recombination, double-strand break repair and DNA mismatch repair; member of the Rad2p nuclease family, with conserved N and I nuclease domains exonuclease Mutants demonstrate sensitivity to cycloheximide, bleomycin, actinomycin D, 5-fluorouracil, and several other antibiotics, as well as irregular shapes and sensitivity to zymolase digestion

0199	GO_TERM:[DNA damage checkpoint] P-Value:2.1e-04
YER079W	
YDL101C	[DUN1] Cell-cycle checkpoint serine-threonine kinase required for DNA damage-induced transcription of certain target genes, phosphorylation of Rad55p and Sml1p, and transient G2/M arrest after DNA damage; also regulates postreplicative DNA repair protein kinase Null mutant is viable, defective in DNA damage repair and DNA damage-resposive induction of RNR genes, and sensitive to DNA damaging agents. dun1pan2 and dun1pan3 double mutants are hypersensitive to replicational stress.
YDR217C	[RAD9] DNA damage-dependent checkpoint protein, required for cell-cycle arrest in G1/S, intra-S, and G2/M; transmits checkpoint signal by activating Rad53p and Chk1p; hyperphosphorylated by Mec1p and Tel1p; potential Cdc28p substrate cell cycle arrest protein radiation sensitive

0200	GO_TERM:[response to DNA damage stimulus] P-Value:1.2e-15 OVERLAP:[Replication factor A complex] <410.40.20> SIZE:3
YIR002C	[MPH1] Member of the DEAH family of helicases, functions in an error-free DNA damage bypass pathway that involves homologous recombination, mutations confer a mutator phenotype
YCR028C-A	[RIM1] Single-stranded DNA-binding protein essential for mitochondrial genome maintenance; involved in mitochondrial DNA replication DNA binding protein
YLR226W	[BUR2] Cyclin for the Sgv1p (Bur1p) protein kinase; Sgv1p and Bur2p comprise a CDK-cyclin complex involved in transcriptional regulation through its phosphorylation of the carboxy-terminal domain of the largest subunit of RNA polymerase II Uncharacterized mutant allele causes increased transcription of SUC2 in the absence of its UAS; Overexpression induces chromosome loss
YMR234W	[RNH1] Ribonuclease H1, removes RNA primers during Okazaki fragment synthesis; degrades RNA attached to the 5'-end of a DNA strand ribonuclease H
YBL088C	[TEL1] Protein kinase, primarily involved in telomere length regulation; contributes to cell cycle checkpoint control in response to DNA damage; functionally redundant with Mec1p; homolog of human ataxia telangiectasia (ATM) gene
YFL014W	[HSP12] Plasma membrane localized protein that protects membranes from desiccation; induced by heat shock, oxidative stress, osmostress, stationary phase entry, glucose depletion, oleate and alcohol; regulated by the HOG and Ras-Pka pathways heat shock protein 12 Null mutant is viable, but shows induction of heat shock response under conditions normally associated with low-level HSP12 expression
YJR144W	[MGM101] Protein involved in mitochondrial genome maintenance; component of the mitochondrial nucleoid, required for the repair of oxidative mtDNA damage mitochondrial nucleoid protein Null mutant is viable. Meiotic segregants with a disrupted mgm101 allele cannot undergo more than 10 divisions on glycerol medium.
YCR092C	[MSH3] Mismatch repair protein, forms dimers with Msh2p that mediate repair of insertion or deletion mutations and removal of nonhomologous DNA ends, contains a PCNA (Pol30p) binding motif required for genome stability forms a complex with Msh2p to repair insertion-deletion mispairs; redundant with Pms3/Msh6p in repair of insertion-deletion mispairs|mutS homolog Null mutant is viable. Inactivation of MSH3 results in low rates of frameshift mutations.
YML032C	[RAD52] Protein that stimulates strand exchange by facilitating Rad51p binding to single-stranded DNA; anneals complementary single-stranded DNA; involved in the repair of double-strand breaks in DNA during vegetative growth and meiosis Null mutant is viable, radiation sensitive; rad52 rad27 double mutants are inviable, double strand break ends are excessively recessed in mutant, rad52 is rescued by rad50 spo13, but not spo13, and is classified as late recombination gene. Growth defects of mgs1 rad18 double mutants are suppressed by overexpression of Rad52.|Deletion of this homologous recombination (HR) gene decreases psoralen-induced recombination and increases mutation frequencies.
YBR136W	[MEC1] Genome integrity checkpoint protein and PI kinase superfamily member; signal transducer required for cell cycle arrest and transcriptional responses prompted by damaged or unreplicated DNA; monitors and participates in meiotic recombination Null mutant is inviable; overproduction of Rad53p rescues some esr1 alleles
YHR164C	[DNA2] Essential tripartite DNA replication factor with single-stranded DNA-dependent ATPase, ATP-dependent nuclease, and helicase activities; required for Okazaki fragment processing; involved in DNA repair pathways; potential Cdc28p substrate DNA replication helicase
YDR499W	[LCD1] Essential protein required for the DNA integrity checkpoint pathways; interacts physically with Mec1p; putative homolog of S. pombe Rad26 and human ATRIP Null mutant is inviable. Null mutant is rescued by deletion of SML1, but deletion of SML1 does not suppress the hypersensitivity to DNA damaging agents caused by the absence of DDC2.
YDL156W	
**YAR007C**	[RFA1] Subunit of heterotrimeric Replication Factor A (RF-A), which is a highly conserved single-stranded DNA binding protein involved in DNA replication, repair, and recombination RF-A|heterotrimeric RPA (RF-A) single-stranded DNA binding protein 69 kDa subunit Null mutant is inviable; cells lacking RFA1 accumulate as multiply budded cells with a single nucleus suggesting a defect in DNA replication; rfa1 repair defects are suppressed by high copy RAD52
YDR097C	[MSH6] Protein required for mismatch repair in mitosis and meiosis, forms a complex with Msh2p to repair both single-base & insertion-deletion mispairs; potentially phosphorylated by Cdc28p human GTBP protein homolog Mutations in MSH6 or MSH3 cause partial defects in MMR, with inactivation of MSH6 resulting in high rates of base-substitution mutations and low rates of frameshift mutations; msh3 msh6 double deletion mutants exhibit microsatellite instability and mutability similar to that in a msh2 mutant.
**YJL173C**	[RFA3] Subunit of heterotrimeric Replication Factor A (RF-A), which is a highly conserved single-stranded DNA binding protein involved in DNA replication, repair, and recombination replication factor-A subunit 3 Null mutant is inviable and arrests as budded and multiply budded cells
**YNL312W**	[RFA2] Subunit of heterotrimeric Replication Factor A (RF-A), which is a highly conserved single-stranded DNA binding protein involved in DNA replication, repair, and recombination 29% identical to the human p34 subunit of RF-A|replication factor RF-A subunit 2 Null mutant is inviable; arrests as budded and multiply budded cells; rfa2 (ts) cells have a mutator and a hyper-recombination phenotype and are more sensitive to hydroxyurea and methyl-methane-sulfonate than wild-type cells

0201	GO_TERM:[GET complex] P-Value:1.4e-08
YOR164C	

YBR137W	
YOL111C	[MDY2] Protein required for efficient mating; involved in shmoo formation and nuclear migration in the pre-zygote; associates with ribosomes and interacts with YOR164C; contains a ubiquitin-like (UBL) domain
YOR007C	[SGT2] Glutamine-rich cytoplasmic protein of unknown function, contains tetratricopeptide (TPR) repeats, which often mediate protein-protein interactions; conserved in human and C. elegans
YAL017W	[PSK1] One of two (see also PSK2) PAS domain containing S/T protein kinases; coordinately regulates protein synthesis and carbohydrate metabolism and storage in response to a unknown metabolite that reflects nutritional status
YDL100C	[GET3] ATPase, subunit of the GET complex; required for the retrieval of HDEL proteins from the Golgi to the ER in an ERD2 dependent fashion; involved in resistance to heat and metal stress Null: YDL100c gene disruption results in sensitivity to As(III), As(V), Co(II) and Cu(II).
YER083C	[GET2] Subunit of the GET complex; required for meiotic nuclear division and for the retrieval of HDEL proteins from the Golgi to the ER in an ERD2 dependent fashion; may be involved in cell wall function null is hypersensitive to calcofluor white suffer an increased spheroplast lysis rate
YGL020C	[GET1] Subunit of the GET complex; required for the retrieval of HDEL proteins from the Golgi to the ER in an ERD2 dependent fashion and for normal mitochondrial morphology and inheritance Null: Required for spore wall formation, but not IME1 induction or nuclear division

0202	GO_TERM:[dolichyl-phosphate-mannose-protein mannosyltransferase activity] P-Value:2.2e-04 OVERLAP:[H+-ATPase, plasma mebrane] <210> SIZE:4
YAL023C	[PMT2] Protein O-mannosyltransferase, transfers mannose residues from dolichyl phosphate-D-mannose to protein serine/threonine residues; acts in a complex with Pmt1p, can instead interact with Pmt5p in some conditions; target for new antifungals dolichyl phosphate-D-mannose:protein O-D-mannosyltransferase Null mutants are viable but show diminished in vitro and in vivo O-mannosylation activity; pmt1 pmt2 double mutant shows severe growth defect but has residual O-mannosylation activity; pmt2 pmt3 pmt4 triple mutant is inviable
YCR043C	
YDL095W	[PMT1] Protein O-mannosyltransferase, transfers mannose residues from dolichyl phosphate-D-mannose to protein serine/threonine residues; acts in a complex with Pmt2p, can instead interact with Pmt3p in some conditions; target for new antifungals dolichyl phosphate-D-mannose:protein O-D-mannosyltransferase Null mutant is viable but shows decrease by 40-50% of in vivo protein O-mannosylation; pmt1 pmt2 double mutant shows severe growth defect but residual O-mannosylation activity; the pmt1 pmt2 pmt3 pmt4 quadruple mutant is inviable
YGR266W	
**YGL008C**	[PMA1] Plasma membrane H+-ATPase, pumps protons out of the cell; major regulator of cytoplasmic pH and plasma membrane potential; part of the P2 subgroup of cation-transporting ATPases plasma membrane H+-ATPase inviable; pma1 mutants are resistant to Dio-9, ethidium bromide and guanidine derivatives
YOR153W	[PDR5] Short-lived membrane ABC (ATP-binding cassette) transporter, actively exports various drugs, expression regulated by Pdr1p; also involved in steroid transport, cation resistance, and cellular detoxification during exponential growth multidrug resistance transporter pleiotropic drug resistance

0203	GO_TERM:[small protein conjugating enzyme activity] P-Value:1.3e-08
YGL087C	[MMS2] Protein involved in error-free postreplication DNA repair; forms a heteromeric complex with Ubc13p that has a ubiquitin-conjugating activity; cooperates with chromatin-associated RING finger proteins, Rad18p and Rad5p Null mutant is viable and is sensitive to MMS and UV
YMR140W	[SIP5] Protein of unknown function; interacts with both the Reg1p/Glc7p phosphatase and the Snf1p kinase interaction between Reg1 and Snf1 is reduced threefold in a sip5Delta mutant.
YDR409W	[SIZ1] SUMO ligase that promotes the attachment of sumo (Smt3p; small ubiquitin-related modifier) to proteins; binds Ubc9p and may bind septins; specifically required for sumoylation of septins in vivo; localized to the septin ring chromatin protein; SUMO1/Smt3 ligase Null mutant is viable. SIZ1 is a dosage bypass suppressor of an SMT4 deletion. A siz1 siz2 deletion has a synthetic phenotype (slow growth). Null mutant exhibits defective Smt3-modification of septins.
YDR092W	[UBC13] Ubiquitin-conjugating enzyme involved in the error-free DNA postreplication repair pathway; interacts with Mms2p to assemble ubiquitin chains at the Ub Lys-63 residue; DNA damage triggers redistribution from the cytoplasm to the nucleus ubiquitin-conjugating enzyme Deletion results in elevated levels of the DNA damage recognition protein Rad4 and an increase in ubiquitylated Rad23.
YDL064W	[UBC9] SUMO-conjugating enzyme involved in the Smt3p conjugation pathway; nuclear protein required for S- and M-phase cyclin degradation and mitotic control; involved in proteolysis mediated by the anaphase-promoting complex cyclosome (APCC) SUMO-conjugating enzyme
YCR066W	[RAD18] Protein involved in postreplication repair; binds single-stranded DNA and has single-stranded DNA dependent ATPase activity; forms heterodimer with Rad6p; contains RING-finger motif ATPase (putative)|zinc finger protein Radiation-sensitive. mgs1 exhibits a synergistic growth defect with rad18. Growth defects of mgs1 rad18 double mutants are suppressed by a mutation in SRS2 or by overexpression of Rad52.|Deletion mutants of this post-replication repair (PRR) gene do not have any cross-link-induced mutations but show increased levels of recombination.

YLR032W	[RAD5] Single-stranded DNA-dependent ATPase, involved in postreplication repair; contains RING finger domain ATPase (putative)|DNA helicase (putative) Radiation-sensitive. mgs1 exhibits a synergistic growth defect with rad5. mgs1 rad5 double mutant has increased sensitivity to hydroxyurea and a greatly increased spontaneous mutation rate.|Deletion mutants of this post-replication repair (PRR) gene do not have any cross-link-induced mutations but show increased levels of recombination.

0204	GO_TERM:[chromatin silencing] P-Value:1.7e-12 OVERLAP:[Chromatin assembly complex (CAC)] <90.10> SIZE:3
**YBR195C**	[MSI1] Subunit of chromatin assembly factor I (CAF-I), regulates the RAS/cAMP pathway via sequestration of Npr1p kinase; localizes to the nucleus and cytoplasm; homologous to human retinoblastoma binding proteins RbAp48 and RbAp46 chromatin assembly factor-I (CAF-I) p50 subunit|negative regulator of ras-mediated cAMP induction|similar to GTP-binding protein beta subunit
**YML102W**	[CAC2] Component of the chromatin assembly complex (with Rlf2p and Msi1p) that assembles newly synthesized histones onto recently replicated DNA, required for building functional kinetochores, conserved from yeast to humans chromatin assembly factor-I (CAF-I) p60 subunit
YNL206C	[RTT106] Protein with a role in regulation of Ty1 transposition Null mutant is viable, but Ty1 retrotransposition is increased.
**YPR018W**	[RLF2] Largest subunit (p90) of the Chromatin Assembly Complex (CAF-I) with Cac2p and Msi1p that assembles newly synthesized histones onto recently replicated DNA; involved in the maintenance of transcriptionally silent chromatin chromatin assembly factor-I (CAF-I) p90 subunit
YMR127C	[SAS2] Histone acetyltransferase (HAT) catalytic subunit of the SAS complex (Sas2p-Sas4p-Sas5p), which acetylates free histones and nucleosomes and regulates transcriptional silencing; member of the MYSTacetyltransferase family zinc finger protein Null mutant is viable, suppresses temperature sensitive defects of orc2-1 and orc5-1; has opposite effects on HML and HMR
YDR181C	[SAS4] Subunit of the SAS complex (Sas2p, Sas4p, Sas5p), which acetylates free histones and nucleosomes and regulates transcriptional silencing; required for the HAT activity of Sas2p
YOR213C	[SAS5] Subunit of the SAS complex (Sas2p, Sas4p, Sas5p), which acetylates free histones and nucleosomes and regulates transcriptional silencing; stimulates Sas2p HAT activity

0205	GO_TERM:[replisome] P-Value:3.0e-18 OVERLAP:[DNA polymerase alpha (I) - primase complex] <410.40.60> SIZE:4
YDL102W	[CDC2] Catalytic subunit of DNA polymerase delta; required for chromosomal DNA replication during mitosis and meiosis, intragenic recombination, repair of double strand DNA breaks, and DNA replication during nucleotide excision repair (NER) DNA polymerase III catalytic (delta) subunit Null mutant is inviable. cdc2 mutants arrest at the mononucleate stage with duplicated spindle pole bodies and no complete spindles. mgs1 mutation suppresses the temperature sensitivity of cdc2 mutants.
YJR006W	[HYS2] DNA polymerase III (delta) subunit, essential for cell viability; involved in DNA replication and DNA repair DNA polymerase delta subunit
YJR043C	[POL32] Third subunit of DNA polymerase delta, involved in chromosomal DNA replication; required for error-prone DNA synthesis in the presence of DNA damage and processivity; interacts with Hys2p, PCNA (Pol30p), and Pol1p DNA polymerase delta subunit Null mutant is viable but is cold-sensitive, hydroxyurea-sensitive, defective for induced mutagenesis, synthetic lethal with pol3, pol30 and pol31
**YNL102W**	[POL1] Catalytic subunit of the DNA polymerase alpha-primase complex, required for the initiation of DNA replication during mitotic DNA synthesis and premeiotic DNA synthesis DNA polymerase I alpha subunit p180 Null mutant is inviable. pol1(ts) mutants show blocked cell division at 36 degrees C
**YBL035C**	[POL12] B subunit of DNA polymerase alpha-primase complex, required for initiation of DNA replication during mitotic and premeiotic DNA synthesis; also functions in telomere capping and length regulation DNA polymerase alpha-primase complex B subunit
**YIR008C**	[PRI1] Subunit of DNA primase, which is required for DNA synthesis and double-strand break repair DNA primase p48 polypeptide Null mutant is inviable; pri1 pri2 mutants are inviable
**YKL045W**	[PRI2] Subunit of DNA primase, which is required for DNA synthesis and double-strand break repair DNA primase p58 polypeptide lethal

0206	GO_TERM:[DNA replication factor C complex] P-Value:1.7e-27 OVERLAP:[Replication factor C complex] <410.40.30> SIZE:5
YDL164C	[CDC9] DNA ligase found in the nucleus and mitochondria, an essential enzyme that joins Okazaki fragments during DNA replication; also acts in nucleotide excision repair, base excision repair, and recombination DNA ligase cell division cycle blocked at 36 degrees, increased sensitivity to ultraviolet radiation and bleomycin; temperature sensitive
YKL113C	[RAD27] 5' to 3' exonuclease, 5' flap endonuclease, required for Okazaki fragment processing and maturation as well as for long-patch base-excision repair; member of the S. pombe RAD2/FEN1 family 42 kDa 5' to 3' exonuclease required for Okazaki fragment processing Null mutant demonstrates temperature-sensitive growth and sensitivity to UV light and methylmethane sulfonate. rad27 mutant cells are defective in Okazaki fragment maturation.
YGL234W	[ADE5,7] Bifunctional enzyme of the 'de novo' purine nucleotide biosynthetic pathway, contains aminoimidazole ribotide synthetase and glycinamide ribotide synthetase activities aminoimidazole ribotide synthetase|glycinamide ribotide synthetase Adenine requiring
YCL016C	[DCC1] Subunit of a complex with Ctf8p and Ctf18p that shares some components with Replication Factor C, required for sister chromatid cohesion and telomere length maintenance benomyl sensitive and defective in sister chromatid cohesion

YJL115W	[ASF1] Nucleosome assembly factor, involved in chromatin assembly after DNA replication, anti-silencing protein that causes derepression of silent loci when overexpressed
YBR088C	[POL30] Proliferating cell nuclear antigen (PCNA), functions as the sliding clamp for DNA polymerase delta; may function as a docking site for other proteins required for mitotic and meiotic chromosomal DNA replication and for DNA repair Proliferating Cell Nuclear Antigen (PCNA)
YOR144C	[ELG1] Protein required for S phase progression and telomere homeostasis, forms an alternative replication factor C complex important for DNA replication and genome integrity; mutants are sensitive to DNA damage
YHR191C	[CTF8] Subunit of a complex with Ctf18p that shares some subunits with Replication Factor C and is required for sister chromatid cohesion
**YOR217W**	[RFC1] Subunit of heteropentameric Replication factor C (RF-C), which is a DNA binding protein and ATPase that acts as a clamp loader of the proliferating cell nuclear antigen (PCNA) processivity factor for DNA polymerases delta and epsilon replication factor C subunit 1|similar to human RFC 140 kDa subunit Null mutant is inviable, rfc1 conditional mutants arrest before mitosis
YER173W	[RAD24] Checkpoint protein, involved in the activation of the DNA damage and meiotic pachytene checkpoints; subunit of a clamp loader that loads Rad17p-Mec3p-Ddc1p onto DNA; homolog of human and S. pombe Rad17 protein cell cycle exonuclease (putative) radiation sensitive
YFR027W	[ECO1] Acetyltransferase required for the establishment of sister chromatid cohesion during DNA replication, but not for its maintenance during G2 and M phases; also required for postreplicative double-strand break repair; interacts with Chl1p
YMR078C	[CTF18] Subunit of a complex with Ctf8p that shares some subunits with Replication Factor C and is required for sister chromatid cohesion; may have overlapping functions with Rad24p in the DNA damage replication checkpoint Null mutant is viable, exhibits increased level of spontaneous mitotic recombination, slow growth, and cold sensitivity
**YJR068W**	[RFC2] Subunit of heteropentameric Replication factor C (RF-C), which is a DNA binding protein and ATPase that acts as a clamp loader of the proliferating cell nuclear antigen (PCNA) processivity factor for DNA polymerases delta and epsilon replication factor C subunit 2|similar to human RFC 37 kDa subunit
**YBR087W**	[RFC5] Subunit of heteropentameric Replication factor C (RF-C), which is a DNA binding protein and ATPase that acts as a clamp loader of the proliferating cell nuclear antigen (PCNA) processivity factor for DNA polymerases delta and epsilon replication factor C subunit 5|similar to human RFC 38 kDa subunit
**YNL290W**	[RFC3] Subunit of heteropentameric Replication factor C (RF-C), which is a DNA binding protein and ATPase that acts as a clamp loader of the proliferating cell nuclear antigen (PCNA) processivity factor for DNA polymerases delta and epsilon replication factor C subunit 3|similar to human RFC 36 kDa subunit
**YOL094C**	[RFC4] Subunit of heteropentameric Replication factor C (RF-C), which is a DNA binding protein and ATPase that acts as a clamp loader of the proliferating cell nuclear antigen (PCNA) processivity factor for DNA polymerases delta and epsilon replication factor C subunit 4|similar to human RFC 40 kDa subunit

0207	GO_TERM:[response to DNA damage stimulus] P-Value:1.9e-08 OVERLAP:[DNA polymerase zeta] <410.40.110> SIZE:2
YLR288C	[MEC3] DNA damage and meiotic pachytene checkpoint protein; subunit of a heterotrimeric complex (Rad17p-Mec3p-Ddc1p) that forms a sliding clamp, loaded onto partial duplex DNA by a clamp loader complex; homolog of human and S. pombe Hus1
YOR368W	[RAD17] Checkpoint protein, involved in the activation of the DNA damage and meiotic pachytene checkpoints; with Mec3p and Ddc1p, forms a clamp that is loaded onto partial duplex DNA; homolog of human and S. pombe Rad1 and U. maydis Rec1 proteins 3'-5'exonuclease (putative)
YPL194W	[DDC1] DNA damage checkpoint protein, part of a PCNA-like complex required for DNA damage response, required for pachytene checkpoint to inhibit cell cycle in response to unrepaired recombination intermediates; potential Cdc28p substrate Null mutant is viable, sensitive to DNA damage and defective in delaying G1-S and G2-M transistion and in decreasing rate of DNA synthesis when DNA is damaged during G1, G2 or S phase, respectively
**YIL139C**	[REV7] Subunit of DNA polymerase zeta, which is involved in DNA repair; required for mutagenesis induced by DNA damage DNA polymerase zeta (pol-zeta) subunit
YOR346W	[REV1] Deoxycytidyl transferase, forms a complex with the subunits of DNA polymerase zeta, Rev3p and Rev7p; involved in repair of abasic sites in damaged DNA deoxycytidyl transferase Null mutant is viable, exhibts decreased revertibility
**YPL167C**	[REV3] Subunit of DNA polymerase zeta, which is involved in DNA repair; required for mutagenesis induced by DNA damage DNA polymerase zeta subunit The null mutant is viable and resists ultraviolet (UV) mutagenesis in both haploid and homozygous mutant diploid cells. Other antimutator phenotypes are also observed.

0208	GO_TERM:[catalytic activity] P-Value:1.8e-01
YGR192C	[TDH3] Glyceraldehyde-3-phosphate dehydrogenase, isozyme 3, involved in glycolysis and gluconeogenesis; tetramer that catalyzes the reaction of glyceraldehyde-3-phosphate to 1,3 bis-phosphoglycerate; detected in the cytoplasm and cell-wall glyceraldehyde-3-phosphate dehydrogenase 3
YDR434W	[GPI17] Transmembrane protein subunit of the glycosylphosphatidylinositol transamidase complex that adds GPIs to newly synthesized proteins; human PIG-Sp homolog GPI transamidase component, human PIG-S homologue
YKL039W	[PTM1] Protein of unknown function, copurifies with late Golgi vesicles containing the v-SNARE Tlg2p membrane protein (putative) Null mutant is viable, no observable phenotype

0209	GO_TERM:[lipid particle] P-Value:3.2e-06

YBR041W	[FAT1] Fatty acid transporter and very long-chain fatty acyl-CoA synthetase, may form a complex with Faa1p or Faa4p that imports and activates exogenous fatty acids fatty acid transporter Null mutant is viable, but is Ole- in presence of cerulenin (i.e., unable to grow on YPD supplemented with oleic acid and cerulenin)
YJL052W	[TDH1] Glyceraldehyde-3-phosphate dehydrogenase, isozyme 1, involved in glycolysis and gluconeogenesis; tetramer that catalyzes the reaction of glyceraldehyde-3-phosphate to 1,3 bis-phosphoglycerate; detected in the cytoplasm and cell-wall glyceraldehyde-3-phosphate dehydrogenase 1 Null mutant is viable, tdh1 tdh2 and tdh1 tdh3 double mutants grow at wild type rates when ethanol is used as a carbon source
YEL023C	
YJR009C	[TDH2] Glyceraldehyde-3-phosphate dehydrogenase, isozyme 2, involved in glycolysis and gluconeogenesis; tetramer that catalyzes the reaction of glyceraldehyde-3-phosphate to 1,3 bis-phosphoglycerate; detected in the cytoplasm and cell-wall glyceraldehyde 3-phosphate dehydrogenase Null mutant is viable, grow poorly on glucose, grow as well as wild-type on ethanol media, tdh2 tdh3 double deletion mutants are inviable

0210	GO_TERM:[protein phosphatase type 1 regulator activity] P-Value:6.6e-04 OVERLAP:[Serine/threonine phoshpoprotein phosphatase] <450> SIZE:6
YIL045W	[PIG2] Putative type-1 protein phosphatase targeting subunit that tethers Glc7p type-1 protein phosphatase to Gsy2p glycogen synthase type-1 protein phosphatase regulatory subunit
**YOR178C**	[GAC1] Regulatory subunit for Glc7p type-1 protein phosphatase (PP1), tethers Glc7p to Gsy2p glycogen synthase, binds Hsf1p heat shock transcription factor, required for induction of some HSF-regulated genes under heat shock Glc7p regulatory subunit
YPR169W	[JIP5] Nucleolar protein of unknown function, exhibits a physical interaction with Bre1p
YER033C	[ZRG8] Cytoplasmic protein of unknown function, transcription is induced under conditions of zinc deficiency
YDR475C	[JIP4] Protein of unknown function; previously annotated as two separate ORFs, YDR474C and YDR475C, which were merged as a result of corrections to the systematic reference sequence
YIR033W	[MGA2] ER membrane protein involved, with its homolog Spt23p, in regulation of OLE1 transcription; inactive ER form dimerizes and one subunit is then activated by ubiquitin/proteasome-dependent processing followed by nuclear targeting Null mutant is viable, shows subtle effects on growth, UV sensitivity, and galactose utilization; mga2 spt23 double deletion mutants are inviable
YOR355W	[GDS1] Protein of unknown function, required for growth on glycerol as a carbon source Null mutant is viable, shows partial impairment of growth on medium containing glycerol as the carbon source. Overexpxression suppresses NAM9-1 glycerol deficient phenotype
YMR219W	[ESC1] Protein localized to the nuclear periphery, involved in telomeric silencing; interacts with PAD4-domain of Sir4p
YDR096W	[GIS1] Transcriptional factor, involved in the expression of genes during nutrient limitation; also involved in the negative regulation of DPP1 and PHR1 zinc finger protein (putative) Null mutant is viable and shows enhanced basal level expression of PHR1
YFL023W	[BUD27] Protein involved in bud-site selection, nutrient signaling, and gene expression controlled by the TOR kinase; diploid mutants display a random budding pattern instead of the wild-type bipolar pattern

0211	GO_TERM:[biological_process] P-Value:9.6e-02
YDR128W	
YHR033W	

0212	GO_TERM:[response to acid] P-Value:8.8e-05
YHR030C	[SLT2] Serine/threonine MAP kinase involved in regulating the maintenance of cell wall integrity and progression through the cell cycle; regulated by the PKC1-mediated signaling pathway Null mutant is viable but temperature sensitive. At elevated temperatures or in the presence of caffeine, mull mutants exhibit cell wall defects that result in cell lysis. Lysis is prevented by addition of 1M sorbitol.
YGR229C	[SMI1] Protein involved in the regulation of cell wall synthesis; proposed to be involved in coordinating cell cycle progression with cell wall integrity 57 kDa nuclear protein Null mutant is viable, shows osmotic sensitivity, sensitivity to cercosporamide, resistance to zymolase; temperature sensitive mutant arrests at S phase with small buds
YPL089C	[RLM1] MADS-box transcription factor, component of the protein kinase C-mediated MAP kinase pathway involved in the maintenance of cell integrity; phosphorylated and activated by the MAP-kinase Slt2p Null mutant is viable but shows caffeine sensitivity

0213	GO_TERM:[catalytic activity] P-Value:1.0e-01 OVERLAP:[Ku complex] <510.180.30.10> SIZE:2

**YMR106C**	[YKU80] Subunit of the telomeric Ku complex (Yku70p-Yku80p), involved in telomere length maintenance, structure and telomere position effect; relocates to sites of double-strand cleavage to promote nonhomologous end joining during DSB repair Ku80 homolog Null mutant is viable, methylmethane sulfonate sensitive, exhibits DNA joining defects; temperature sensitive, bleomycin sensitive
YJR064W	[CCT5] Subunit of the cytosolic chaperonin Cct ring complex, related to Tcp1p, required for the assembly of actin and tubulins in vivo chaperonin subunit epsilon subunit
YNR016C	[ACC1] Acetyl-CoA carboxylase, biotin containing enzyme that catalyzes the carboxylation of acetyl-CoA to form malonyl-CoA; required for de novo biosynthesis of long-chain fatty acids acetyl CoA carboxylase acc1 spores fail to enter vegetative growth
YJR072C	[NPA3] Essential, conserved, cytoplasmic ATPase; phosphorylated by the Pcl1p-Pho85p kinase complex
YNL037C	[IDH1] Subunit of mitochondrial NAD(+)-dependent isocitrate dehydrogenase, which catalyzes the oxidation of isocitrate to alpha-ketoglutarate in the TCA cycle isocitrate dehydrogenase 1 alpha-4-beta-4 subunit Null mutant is viable, grows at a reduced rate on glycerol, lactate, and acetate
YPL140C	[MKK2] Mitogen-activated kinase kinase involved in protein kinase C signaling pathway that controls cell integrity; upon activation by Bck1p phosphorylates downstream target, Slt2p; functionally redundant with Mkk1p protein kinase Null mutant is viable and shows no obvious phenotypes; mkk1 mkk2 double mutant is caffeine-sensitive and shows a temperature-sensitive cell lysis defect remediated by osmotic stabilizers

0214	
YDR161W	
YHR174W	[ENO2] Enolase II, a phosphopyruvate hydratase that catalyzes the conversion of 2-phosphoglycerate to phosphoenolpyruvate during glycolysis and the reverse reaction during gluconeogenesis; expression is induced in response to glucose enolase

0215	GO_TERM:[transferase activity] P-Value:2.2e-01 OVERLAP:[Pho85p complexes] <133.20> SIZE:6
YDL186W	
YJL167W	[ERG20] Farnesyl pyrophosphate synthetase, has both dimethylallyltranstransferase and geranyltranstransferase activities; catalyzes the formation of C15 farnesyl pyrophosphate units for isoprenoid and sterol biosynthesis farnesyl diphosphate synthetase (FPP synthetase)
**YOL001W**	[PHO80] Cyclin, negatively regulates phosphate metabolism; Pho80p-Pho85p (cyclin-CDK complex) phosphorylates Pho4p and Swi5p; deletion of PHO80 leads to aminoglycoside supersensitivity; truncated form of PHO80 affects vacuole inheritance Pho80p cyclin The null mutant is viable but constitutively derepresses PHO5 (acid phosphatase) transcription and is postive for deoxythymidine monophosphate uptake. The null mutant is supersensitive to aminoglycoside.
YFL030W	[AGX1] Alanine : glyoxylate aminotransferase, catalyzes the synthesis of glycine from glyoxylate, which is one of three pathways for glycine biosynthesis in yeast; has similarity to mammalian and plant alanine : glyoxylate aminotransferases
YIL077C	

0216	GO_TERM:[cytoskeletal protein binding] P-Value:5.0e-03 OVERLAP:[Actin-associated proteins] <140.20.20> SIZE:25
YDR314C	[RAD34] Protein involved in nucleotide excision repair (NRE); homologous to RAD4
YML011C	[RAD33] Putative protein of unknown function; green fluorescent protein (GFP)-fusion protein localizes to the nucleus
YLR218C	
YLR144C	[ACF2] Intracellular beta-1,3-endoglucanase, expression is induced during sporulation; may have a role in cortical actin cytoskeleton assembly Null mutant shows defect in in vitro actin assembly in the permeabilized cell assay
YCR008W	[SAT4] Ser/Thr protein kinase involved in salt tolerance; funtions in regulation of Trk1p-Trk2p potassium transporter; partially redundant with Hal5p; has similarity to Npr1p
YCR009C	[RVS161] Amphiphysin-like lipid raft protein; subunit of a complex (Rvs161p-Rvs167p) that regulates polarization of the actin cytoskeleton, endocytosis, cell polarity, cell fusion and viability following starvation or osmotic stress Null mutant is viable, rvs161 mutations result in a delocalization of the actin cytoskeleton, high salt sensitivity, random budding pattern in diploid cells, defects in endocytosis, and reduced viability upon starvation; rvs161 mutants exhibit synthetic lethality with sst2 mutants
**YLR429W**	[CRN1] Coronin, cortical actin cytoskeletal component that associates with the Arp2p/Arp3p complex to regulate its activity Dictyostelium and human actin-binding protein coronin homolog Overexpression of CRN1 causes growth arrest and redistribution of Arp2p and Crn1p into aberrant actin loops.

0217	GO_TERM:[double-strand break repair via single-strand annealing, removal of nonhomologous ends] P-Value:9.7e-06 OVERLAP:[NEF1 complex] <510.180.10.10> SIZE:3

YAL027W	
YER049W	[TPA1] Hypothetical protein
**YML095C**	[RAD10] Single-stranded DNA endonuclease (with Rad1p), cleaves single-stranded DNA during nucleotide excision repair and double-strand break repair; subunit of Nucleotide Excision Repair Factor 1 (NEF1); homolog of human ERCC1 protein ssDNA endonuclease radiation sensitive|Deletion of this nucleotide excision repair (NER) gene results in lower levels of cross-link-induced recombination but higher mutation frequencies than wild-type cells.
**YPL022W**	[RAD1] Single-stranded DNA endonuclease (with Rad10p), cleaves single-stranded DNA during nucleotide excision repair and double-strand break repair; subunit of Nucleotide Excision Repair Factor 1 (NEF1); homolog of human XPF protein UV endonuclease radiation sensitive|Deletion of this nucleotide excision repair (NER) gene results in lower levels of cross-link-induced recombination but higher mutation frequencies than wild-type cells.

0218	GO_TERM:[biological_process] P-Value:9.6e-02
YMR031C	
YMR086W	

0219	GO_TERM:[eisosome] P-Value:7.4e-06 OVERLAP:[cytoplasmic ribosomal large subunit] <500.40.10> SIZE:81
YKL142W	[MRP8] Putative mitochondrial ribosomal protein, has similarity to E. coli ribosomal protein S2 ribosomal protein
**YNL067W**	[RPL9B] Protein component of the large (60S) ribosomal subunit, nearly identical to Rpl9Ap and has similarity to E. coli L6 and rat L9 ribosomal proteins ribosomal protein L9B (L8B) (rp24) (YL11)
YGR086C	[PIL1] Integral membrane protein that along with Lsp1p is a primary component of eisosomes, large immobile patch structures at the cell cortex associated with endocytosis; null mutants show activation of Pkc1p/Ypk1p stress resistance pathways
YPL004C	[LSP1] Integral membrane protein that along with Pil1p is a primary component of eisosomes, large immobile patch structures at the cell cortex associated with endocytosis; null mutants show activation of Pkc1p/Ypk1p stress resistance pathways

0220	GO_TERM:[binding] P-Value:2.7e-01
YDR032C	[PST2] Protein of unknown function with similarity to members of a family of flavodoxin-like proteins; induced by oxidative stress in a Yap1p dependent manner; GFP-fusion protein localizes to the cytoplasm in a punctate pattern
YHL024W	[RIM4] Putative RNA-binding protein required for the expression of early and middle sporulation genes RNA-binding protein of the RRM class (putative) Null mutant is viable. Homozygous null diploid fails to sporulate, does not form meiosis I or II spindles, and exhibits reduced expression of early and middle sporulation-specific genes. Null mutant is suppressed by hyperactive Ime2p derivative, but not by overexpression IME1
YBR185C	[MBA1] Protein involved in assembly of mitochondrial respiratory complexes; may act as a receptor for proteins destined for export from the mitochondrial matrix to the inner membrane Null mutant is viable, conditionally defective in the assembly of mitochondrial respiratory complexes
YCL032W	[STE50] Protein involved in mating response, invasive/filamentous growth, and osmotolerance, acts as an adaptor that links G protein-associated Cdc42p-Ste20p complex to the effector Ste11p to modulate signal transduction contains SAM (sterile alpha motif) Null mutant is viable, sterile, has a modulated sensitivity to alpha-pheromone

0221	GO_TERM:[DNA strand elongation] P-Value:1.9e-13 OVERLAP:[DNA polymerase epsilon (II)] <410.40.100> SIZE:3
YJL090C	[DPB11] Essential BRCT repeat protein, required on the prereplicative complex at replication origins for loading DNA polymerases to initiate DNA synthesis, also required for S/M checkpoint control DNA polymerase II complex Null mutant is inviable; conditional allele demonstrates defective S-phase progression
YKL108W	[SLD2] Protein required for DNA replication, phosphorylated in S phase by S-phase cyclin-dependent kinases (Cdks), phosphorylation is essential for DNA replication and for complex formation with Dpb11p; potential Cdc28p substrate Null mutant is inviable; conditional mutant is defective in DNA replication and DNA replication checkpoint
**YNL262W**	[POL2] Catalytic subunit of DNA polymerase epsilon, one of the major chromosomal DNA replication polymerases characterized by processivity and proofreading exonuclease activity; also involved in DNA synthesis during DNA repair DNA polymerase II
**YPR175W**	[DPB2] Second largest subunit of DNA polymerase II (DNA polymerase epsilon), required for normal yeast chromosomal replication; expression peaks at the G1/S phase boundary; potential Cdc28p substrate DNA polymerase epsilon subunit B Null mutant is inviable; conditional mutant shows defects in DNA replication
**YBR278W**	[DPB3] Third-largest subunit of DNA polymerase II (DNA polymerase epsilon), required to maintain fidelity of chromosomal replication and also for inheritance of telomeric silencing; mRNA abundance peaks at the G1/S boundary of the cell cycle DNA polymerase II C and C' subunits Null mutant is viable, shows increased spontaneous mutation rate

YDR121W	[DPB4] Shared subunit of DNA polymerase epsilon and of ISW2/yCHRAC chromatin accessibility complex; involved in both chromosomal DNA replication and in inheritance of telomeric silencing DNA polymerase II (epsilon) 4th subunit

0222	GO_TERM:[DNA replication] P-Value:4.4e-30 OVERLAP:[Pre-replication complex (pre-RC)] <410.30> SIZE:16
YOL146W	[PSF3] Subunit of the GINS complex (Sld5p, Psf1p, Psf2p, Psf3p), which is localized to DNA replication origins and implicated in assembly of the DNA replication machinery subunit of the GINS complex
YDR013W	[PSF1] Subunit of the GINS complex (Sld5p, Psf1p, Psf2p, Psf3p), which is localized to DNA replication origins and implicated in assembly of the DNA replication machinery subunit of the GINS complex
YJL072C	[PSF2] Subunit of the GINS complex (Sld5p, Psf1p, Psf2p, Psf3p), which is localized to DNA replication origins and implicated in assembly of the DNA replication machinery subunit of the GINS complex
YCR086W	[CSM1] Nucleolar protein that forms a complex with Lrs4p which binds Mam1p at kinetochores during meiosis I to mediate accurate chromosome segregation, may be involved in premeiotic DNA replication; possible role in telomere maintenance
YMR048W	[CSM3] Protein required for accurate chromosome segregation during meiosis Null: missegregates chromosomes in meiosis
YCL061C	[MRC1] S-phase checkpoint protein found at replication forks, required for DNA replication; also required for Rad53p activation during DNA replication stress, where it forms a replication-pausing complex with Tof1p and is phosphorylated by Mec1p; protein involved in replication checkpoint Null: sensitive to hydroxyurea; replication checkpoint defective; slower DNA replication than wild type; partial loss of silencing at telomeres and HM loci; synthetic lethal with rad9 null, rad53-21, and mec1-21.
YDL017W	[CDC7] DDK (Dbf4-dependent kinase) catalytic subunit required for firing origins and replication fork progression in mitosis through phosphorylation of Mcm2-7p complexes and Cdc45p; kinase activity correlates with cyclical DBF4 expression Cdc7p-Dbf4p kinase complex catalytic subunit Null mutant is inviable. cdc7 mutant arrests at G(sub)1/S phase with duplicated spindle pole bodies and no spindles; the spindle pole bodies eventually enlarge, invaginate from the nuclear envelope into the center of the nucleus, sometimes fragmenting into three or four smaller spindle pole bodies. In heterozygotes, cdc7 spores fail to germinate.
**YLR274W**	[CDC46] Component of the hexameric MCM complex, which is important for priming origins of DNA replication in G1 and becomes an active ATP-dependent helicase that promotes DNA melting and elongation when activated by Cdc7p-Dbf4p in S-phase Null mutant is inviable; at nonpermissive temperature cdc46(ts) mutants arrest with a large bud and a single nucleus and exhibit a high rate of recombination
YNL273W	[TOF1] Subunit of a replication-pausing checkpoint complex (Tof1p-Mrc1p-Csm3p) that acts at the stalled replication fork to promote sister chromatid cohesion after DNA damage, facilitating gap repair of damaged DNA; interacts with the MCM helicase
**YBR202W**	[CDC47] Component of the hexameric MCM complex, which is important for priming origins of DNA replication in G1 and becomes an active ATP-dependent helicase that promotes DNA melting and elongation when activated by Cdc7p-Dbf4p in S-phase Null mutant is inviable, at nonpermissive temperature cdc47(ts) mutants arrest with a large bud and a single nucleus
YIL150C	[MCM10] Essential, chromatin-associated protein involved in the initiation of DNA replication; required for the association of the MCM2-7 complex with replication origins
YDR489W	[SLD5] Subunit of the GINS complex (Sld5p, Psf1p, Psf2p, Psf3p), which is localized to DNA replication origins and implicated in assembly of the DNA replication machinery subunit of the GINS complex
**YBL023C**	[MCM2] Protein involved in DNA replication; component of the Mcm2-7 hexameric complex that binds chromatin as a part of the pre-replicative complex Null mutant is inviable, at nonpermissive temperature mcm2(ts) mutants arrest with a large bud and a single nucleus, with < 2N DNA content, and exhibit a high rate of recombination; mcm2 mutants are defective in minichromosome maintenance; mcm2-1 cdc45-1 mutants are synthetically lethal
**YPR019W**	[CDC54] Essential helicase component of heterohexameric MCM2-7 complexes which bind pre-replication complexes on DNA and melt the DNA prior to replication; accumulates in the nucleus in G1; homolog of S. pombe Cdc21p Null mutant is inviable; at nonpermissive temperature cdc54(ts) mutants arrest with a large bud and a single nucleus and exhibit a high rate of recombination
**YLR103C**	[CDC45] DNA replication initiation factor; recruited to MCM pre-RC complexes at replication origins; promotes release of MCM from Mcm10p, recruits elongation machinery; mutants in human homolog may cause velocardiofacial and DiGeorge syndromes chromosomal DNA replication initiation protein required for minichromosome maintenance and chromosomal DNA replication
**YEL032W**	[MCM3] Protein involved in DNA replication; component of the Mcm2-7 hexameric complex that binds chromatin as a part of the pre-replicative complex Null mutant is inviable, at nonpermissive temperature mcm3(ts) mutants arrest with a large bud and a single nucleus and exhibit a high rate of recombination; mcm3 mutants are defective in minichromosome maintenance; mcm3-1 cdc45-1 mutants are synthetically lethal
**YGL201C**	[MCM6] Protein involved in DNA replication; component of the Mcm2-7 hexameric complex that binds chromatin as a part of the pre-replicative complex

0223	GO_TERM:[catalytic activity] P-Value:7.3e-02 OVERLAP:[Actin-associated proteins] <140.20.20> SIZE:25
**YHR179W**	[OYE2] Widely conserved NADPH oxidoreductase containing flavin mononucleotide (FMN), homologous to Oye3p with slight differences in ligand binding and catalytic properties; may be involved in sterol metabolism NAPDH dehydrogenase (old yellow enzyme), isoform 2
YLL060C	[GTT2] Glutathione S-transferase capable of homodimerization; functional overlap with Gtt2p, Grx1p, and Grx2p glutathione transferase

0224	GO_TERM:[macromolecule metabolism] P-Value:7.6e-01 OVERLAP:[Actin-associated proteins] <140.20.20> SIZE:25
YBR218C	[PYC2] Pyruvate carboxylase isoform, cytoplasmic enzyme that converts pyruvate to oxaloacetate; highly similar to isoform Pyc1p but differentially regulated; mutations in the human homolog are associated with lactic acidosis pyruvate carboxylase Null mutant is viable; pyc1 pyc2 double mutant is unable to grow on glucose as sole carbon source unless aspartate is added to the medium
**YNL079C**	[TPM1] Major isoform of tropomyosin; binds to and stabilizes actin cables and filaments, which direct polarized cell growth and the distribution of several organelles; acetylated by the NatB complex and acetylated form binds actin most efficiently tropomyosin I Null mutant is viable, grows slowly, exhibits cell size heterogeneity, has delocalized deposition of chitin, mates poorly; exhibits loss of actin cables

0225	GO_TERM:[microfilament motor activity] P-Value:3.4e-10 OVERLAP:[Actin-associated motorproteins] <140.20.30> SIZE:7
**YAL029C**	[MYO4] One of two type V myosins; required for mother-specific HO expression, for the bud tip localization of ASH1 and IST2 mRNA; facilitates growth and orientation of ER tubules along with She3p myosin V heavy chain Null mutant is viable, has no detectable phenotype, either alone or in conjunction with mutations in other myosin genes. Overexpression of MYO4 results in several morphological abnormalities, including the formation of short strings of unseparated cells in diploid strains, or clusters of cells in haploid strains
YBR130C	[SHE3] Protein that acts as an adaptor between Myo4p and the She2p-mRNA complex; part of the mRNA localization machinery that restricts accumulation of certain proteins to the bud; also required for cortical ER inheritance
YKL130C	[SHE2] RNA-binding protein that binds specific mRNAs and interacts with She3p; part of the mRNA localization machinery that restricts accumulation of certain proteins to the bud
YKL124W	[SSH4] Protein whose overexpression confers resistance to the growth inhibitor leflunomide; suppressor of shr3 mutation
YMR071C	[TVP18] Integral membrane protein localized to late Golgi vesicles along with the v-SNARE Tlg2p integral membrane protein Null: no notable phenotype
YBR109C	[CMD1] Calmodulin; Ca++ binding protein that regulates Ca++ independent processes (mitosis, bud growth, actin organization, endocytosis, etc.) and Ca++ dependent processes (stress-activated pathways), targets include Nuf1p, Myo2p and calcineurin calmodulin
YOR035C	[SHE4] Protein containing a UCS (UNC-45/CRO1/SHE4) domain, binds to myosin motor domains to regulate myosin function; involved in endocytosis, polarization of the actin cytoskeleton, and asymmetric mRNA localization
**YHR023W**	[MYO1] Type II myosin heavy chain, required for wild-type cytokinesis and cell separation; localizes to the actomyosin ring; binds to myosin light chains Mlc1p and Mlc2p through its IQ1 and IQ2 motifs respectively class II myosin Null mutant is viable, exhibts abnormal chitin distribution and cell wall organization at the mother-bud neck in a high proportion of dividing cells; exhibits abberant nuclear migration and cytokinesis; bem2 myo1 double mutants are inviable
YGL106W	[MLC1] Essential light chain for myosin Myo2p; may stabilize Myo2p by binding to the neck region; may interact with Myo1p, Iqg1p, and Myo2p to coordinate formation and contraction of the actomyosin ring with targeted membrane deposition myosin Myo2p light chain Null mutant is inviable; MLC1 is halploinsufficient, the haploinsufficiency exhibited by MLC1 is suppressed by reduced copies of MYO2; a diploid strain hemizygous for both MYO2 and MLC1 is viable
**YOR326W**	[MYO2] One of two type V myosins, involved in polarized distribution of mitochondria; required for mitochondrion and vacuole inheritance and nuclear spindle orientation; moves multiple cargo; reversibly phosphorylated in vivo class V myosin Null mutant is inviable. myo2-66 (E511K), a temperature-sensitive allele, accumulates secretory vesicles and exhibits defects in initiation of new buds and delocalized chitin.

0226	GO_TERM:[autophagy] P-Value:8.2e-05
YLR423C	[ATG17] Protein that interacts with and is required for activation of Apg1p protein kinase; involved in autophagy but not in the Cvt (cytoplasm to vacuole targeting) pathway required for activation of Apg1 protein kinase|Null mutant is viable and has defect in autophagy
YPL166W	[ATG29] Protein of unknown function; green fluorescent protein (GFP)-fusion protein localizes to the cytoplasm in a punctate pattern

YPR049C	[ATG11] Peripheral membrane protein required for delivery of aminopeptidase I (Lap4p) to the vacuole in the cytoplasm-to-vacuole targeting pathway; also required for peroxisomal degradation (pexophagy) Oligomeric, coiled-coil, peripheral membrane protein required for stable binding of precursor API to its target membrane. cvt9 is defective in maturation of the vacuolar protein, aminopeptidase I and exhibits minor defects in autophagy|cvt9 is defective in vacuolar delivery of aminopeptidase I and peroxisome degradation but is not needed for macroautophagy. The null mutant is viable and is relatively starvation-insensitive.
YGR035C	
YBR197C	
YKL002W	[DID4] Class E Vps protein of the ESCRT-III complex, required for sorting of integral membrane proteins into lumenal vesicles of multivesicular bodies, and for delivery of newly synthesized vacuolar enzymes to the vacuole, involved in endocytosis class E vacuolar-protein sorting and endocytosis factor
YLR108C	

0227	GO_TERM:[dynactin complex] P-Value:1.3e-11 OVERLAP:[Dynactin complex] <140.30.30.30> SIZE:3
YNL090W	[RHO2] Non-essential small GTPase of the Rho/Rac subfamily of Ras-like proteins, involved in the establishment of cell polarity and in microtubule assembly GTP-binding protein|rho subfamily
YPR165W	[RHO1] GTP-binding protein of the rho subfamily of Ras-like proteins, involved in establishment of cell polarity; regulates protein kinase C (Pkc1p) and the cell wall synthesizing enzyme 1,3-beta-glucan synthase (Fks1p and Gsc2p) GTP-binding protein|rho subfamily null is inviable; synthetic lethal with bem2
YBL106C	[SRO77] Protein with roles in exocytosis and cation homeostasis; functions in docking and fusion of post-Golgi vesicles with plasma membrane; homolog of Sro7p and Drosophila lethal giant larvae tumor suppressor; interacts with SNARE protein Sec9p yeast homolog of the Drosophila tumor suppressor, lethal giant larvae Null mutant is viable and shows no phenotypes, but is cs- in combination with sro7/sni1 (YPR032W) null; sro7/sni1 sro77/sni2 double mutants have an exocytic defect, accumulate post-Golgi vesicles, have partially delocalized actin, and suppress the growth and cell separation defects of myo1 cells.
YPR083W	[MDM36] Protein required for normal mitochondrial morphology and inheritance
**YPL174C**	[NIP100] Large subunit of the dynactin complex, which is involved in partitioning the mitotic spindle between mother and daughter cells; putative ortholog of mammalian p150(glued) large subunit of dynactin complex (putative) Null mutant is viable but exhibits slow growth and defects in partitioning into daughter cells.
**YMR294W**	[JNM1] Component of the yeast dynactin complex, consisting of Nip100p, Jnm1p, and Arp1p; required for proper nuclear migration and spindle partitioning during mitotic anaphase B
YDR106W	[ARP10] Component of the dynactin complex, localized to the pointed end of the Arp1p filament; may regulate membrane association of the complex
**YHR129C**	[ARP1] Actin-related protein of the dynactin complex; required for spindle orientation and nuclear migration; putative ortholog of mammalian centractin Null mutant is viable, but both null mutations and overexpression lead to defects in spindle orientation and nuclear migration (during mitosis in arp1 mutants the nucleus fails to move into the neck).

0228	GO_TERM:[pyruvate dehydrogenase complex (sensu Eukaryota)] P-Value:2.0e-14 OVERLAP:[Pyruvate dehydrogenase] <390> SIZE:5
YDR148C	[KGD2] Dihydrolipoyl transsuccinylase, a component of the mitochondrial alpha-ketoglutarate dehydrogenase complex, which catalyzes a step in the tricarboxylic acid (TCA) cycle, the oxidative decarboxylation of alpha-ketoglutarate to succinyl-CoA alpha-ketoglutarate dehydrogenase complex dihydrolipoyl transsuccinylase component Null mutant is viable but is respiratory deficient (pet-), and its mitochondria are unable to catalyze the reduction of NAD+ by alpha-ketoglutarate
**YFL018C**	[LPD1] Dihydrolipoamide dehydrogenase, the lipoamide dehydrogenase component (E3) of the pyruvate dehydrogenase and 2-oxoglutarate dehydrogenase multi-enzyme complexes dihydrolipoamide dehydrogenase precursor (mature protein is the E3 component of alpha-ketoacid dehydrogenase complexes) unable to utilize glycine as sole nitrogen source
YFR049W	[YMR31] Mitochondrial ribosomal protein of the small subunit, has similarity to human mitochondrial ribosomal protein MRP-S36 mitochondrial ribosomal protein
YIL125W	[KGD1] Component of the mitochondrial alpha-ketoglutarate dehydrogenase complex, which catalyzes a key step in the tricarboxylic acid (TCA) cycle, the oxidative decarboxylation of alpha-ketoglutarate to form succinyl-CoA alpha-ketoglutarate dehydrogenase Null mutant is viable but is deficient in alpha-ketoglutarate dehydrogenase, is therefore respiratory deficient, cannot grow on glycerol, and produces increased amount of organic acids during growth on glucose
**YGR193C**	[PDX1] Dihydrolipoamide dehydrogenase (E3)-binding protein (E3BP) of the mitochondrial pyruvate dehydrogenase (PDH) complex, plays a structural role in the complex by binding and positioning E3 to the dihydrolipoamide acetyltransferase (E2) core pyruvate dehydrogenase complex protein X component
YDR430C	[CYM1] Lysine-specific metalloprotease of the mitochondrial intermembrane space, member of the pitrilysin family; degrades proteins and presequence peptides cleaved from imported proteins; required for normal mitochondrial morphology Metalloprotease

**YBR221C**	[PDB1] E1 beta subunit of the pyruvate dehydrogenase (PDH) complex, which is an evolutionarily-conserved multi-protein complex found in mitochondria pyruvate dehydrogenase beta subunit (E1 beta)
**YER178W**	[PDA1] E1 alpha subunit of the pyruvate dehydrogenase (PDH) complex, catalyzes the direct oxidative decarboxylation of pyruvate to acetyl-CoA, regulated by glucose pyruvate dehydrogenase alpha subunit (E1 alpha) Null mutant is viable, exhibits reduced growth on glucose and increased formation of petites
**YNL071W**	[LAT1] Dihydrolipoamide acetyltransferase component (E2) of pyruvate dehydrogenase complex, which catalyzes the oxidative decarboxylation of pyruvate to acetyl-CoA pyruvate dehydrogenase complex dihydrolipoamide acetyltransferase component (E2)

0229	GO_TERM:[Ras protein signal transduction] P-Value:1.1e-09
YLR310C	[CDC25] Membrane bound guanine nucleotide exchange factor (GEF or GDP-release factor); indirectly regulates adenylate cyclase through activation of Ras1p and Ras2p by stimulating the exchange of GDP for GTP; required for progression through G1 adenylate cyclase regulatory protein Null mutant is inviable; arrests at G(sub)1; low levels cAMP and decreased levels of Mg2+-dependent cyclase activity
YBR225W	
YGL197W	[MDS3] Protein with an N-terminal kelch-like domain, putative negative regulator of early meiotic gene expression; required, with Pmd1p, for growth under alkaline conditions Null mutant is viable; mds3 pmd1 double deletion mutants exhibit starvation-independent expression of early sporulation-specific genes; mds3 is a suppressor of mck1 sporulation defects; amino terminal truncation generates a dominant negative allele
YOR101W	[RAS1] GTPase involved in G-protein signaling in the adenylate cyclase activating pathway, plays a role in cell proliferation; localized to the plasma membrane; homolog of mammalian RAS proto-oncogenes ras homolog
YBR140C	[IRA1] GTPase-activating protein that negatively regulates RAS by converting it from the GTP- to the GDP-bound inactive form, required for reducing cAMP levels under nutrient limiting conditions, mediates membrane association of adenylate cyclase GTPase activating protein (GAP) Null mutant is viable, exhibits constitutive activation of the Ras/cyclic AMP (cAMP) pathway, heat shock sensitivity, nitrogen starvation sensitivity, sporulation deficiency, suppresses lethality of cdc25, but not cyr1, ras1, or ras2 mutants
YLL016W	
YNL098C	[RAS2] GTP-binding protein that regulates the nitrogen starvation response, sporulation, and filamentous growth; farnesylation and palmitoylation required for activity and localization to plasma membrane; homolog of mammalian Ras proto-oncogenes small GTP-binding protein Loss of function mutants grow poorly on nonfermentable carbon sources, sporulate in rich media and are unable to differentiate into a pseudohyphal form

0230	GO_TERM:[actin cortical patch] P-Value:1.2e-04 OVERLAP:[Actin-associated proteins] <140.20.20> SIZE:25
YGR233C	[PHO81] Cyclin-dependent kinase (CDK) inhibitor, regulates Pho80p-Pho85p and Pcl7p-Pho85p cyclin-CDK complexes in response to phosphate levels; required for derepression of PHO5; transcriptionally regulated by Pho4p and Pho2p phosphatase deficient
YHR071W	[PCL5] Cyclin, interacts with Pho85p cyclin-dependent kinase (Cdk), induced by Gcn4p at level of transcription, specifically required for Gcn4p degradation, may be sensor of cellular protein biosynthetic capacity
YGL063W	[PUS2] Putative pseudouridine synthase pseudouridine synthase
YJL005W	[CYR1] Adenylate cyclase, required for cAMP production and cAMP-dependent protein kinase signaling; involved in cell cycle control and glucose and nitrogen repression of sporulation adenylate cyclase Null mutant is inviable. cyr1 transiently arrests in G1 and sporulates precociously. N-terminal domain is dispensable for mitotic G1 arrest after nitrogen starvation, but required for sporulation. When altered, cAMP levels remain high and cells continue to bud with abnormal spindles
**YNL138W**	[SRV2] CAP (cyclase-associated protein) subunit of adenylyl cyclase complex; N-terminus binds adenylyl cyclase and facilitates activation by RAS; C-terminus binds ADP-actin monomers, facilitating regulation of actin dynamics and cell morphogenesis 70 kDa adenylyl cyclase-associated protein
**YLL050C**	[COF1] Cofilin, promotes actin filament depolarization in a pH-dependent manner; binds both actin monomers and filaments and severs filaments , thought to be regulated by phosphorylation at SER4, ubiquitous and essential in eukaryotes actin binding and severing protein|cofilin
**YMR092C**	[AIP1] Actin cortical patch component, interacts with the actin depolymerizing factor cofilin; required to restrict cofilin localization to cortical patches; contains WD repeats actin cortical patch component

0231	GO_TERM:[coated pit] P-Value:6.0e-12 OVERLAP:[AP-2 complex] <260.20.20> SIZE:4

YBR058C	[UBP14] Ubiquitin-specific protease that specifically disassembles unanchored ubiquitin chains; involved in fructose-1,6-bisphosphatase (Fbp1p) degradation; similar to human isopeptidase T ubiquitin-specific protease Null mutant is viable but show accumulation of free ubiquitin chains, which correlates with defects in ubiquitin-dependent proteolysis; overexpression of mutant or wild-type Ubp14p can inhibit protein degration too
YOL113W	[SKM1] Member of the PAK family of serine/threonine protein kinases with similarity to Ste20p and Cla4p; proposed to be a downstream effector of Cdc42p during polarized growth
**YBL037W**	[APL3] Alpha-adaptin, large subunit of the clathrin associated protein complex (AP-2); involved in vesicle mediated transport clathrin associated protein complex large subunit
**YJR005W**	[APL1] Beta-adaptin, large subunit of the clathrin associated protein complex (AP-2); involved in vesicle mediated transport; similar to mammalian beta-chain of the clathrin associated protein complex beta-adaptin|clathrin associated protein complex large subunit
**YJR058C**	[APS2] Small subunit of the clathrin-associated adaptor complex AP-2, which is involved in protein sorting at the plasma membrane; related to the sigma subunit of the mammalian plasma membrane clathrin-associated protein (AP-2) complex clathrin associated protein complex small subunit null mutant is viable; slight effect on chc1-ts cell growth
**YOL062C**	[APM4] Mu2-like subunit of the clathrin associated protein complex (AP-2); involved in vesicle transport clathrin associated protein complex medium subunit

0232	GO_TERM:[meiotic recombination] P-Value:7.2e-06
YMR133W	[REC114] Protein involved in early stages of meiotic recombination; possibly involved in the coordination of recombination and meiotic division; mutations lead to premature initiation of the first meiotic division early sporulation protein reduced meiotic recombination, rec114 mutants execute meiosis I early, are rescued by spo13 and are epistatic to rad52 spo13
YER044C-A	[MEI4] Meiosis-specific protein involved in recombination; required for chromosome synapsis; required for production of viable spores 88 bp intron at 5' end spliced independently of MER1|meiosis-specific protein Loss of full chromosome pairing, heteroduplex DNA, synaptonemal complexes, meiotic intra- and interchromosomal gene conversion, reciprocal recombination and viable spores. mei4 executes both divisions with a delay in meiosis II, is rescued by spo13 and is epiststic to rad52
YJR021C	[REC107] Protein involved in early stages of meiotic recombination; involved in altering chromatin structure at DNA double-stranded break sites and in coordination between the initiation of recombination and the first division of meiosis ds break formation complex subunit reduced meiotic recombination

0233	GO_TERM:[commitment complex] P-Value:4.3e-04 OVERLAP:[Actin-associated motorproteins] <140.20.30> SIZE:7
YNL210W	[MER1] Protein with RNA-binding motifs required for meiosis-specific mRNA splicing; required for chromosome pairing and meiotic recombination RNA-binding motif protein required for MRE2-dependent mRNA splicing Null mutant is viable, associated with decreased levels of inter-and intrachromosomal meiotic recombination; production of inviable spores, multicopy REC107 restores gene conversion and syntaptonemal complexes to mer1 mutants, but not reciprocal recombination of viability
**YBR172C**	[SMY2] Protein of unknown function that interacts with Myo2p; has similarity to S. pombe Mpd2
YKL074C	[MUD2] Protein involved in early pre-mRNA splicing; component of the pre-mRNA-U1 snRNP complex, the commitment complex; interacts with Msl5p/BBP splicing factor and Sub2p; similar to metazoan splicing factor U2AF65
YLR116W	[MSL5] Component of the commitment complex, which defines the first step in the splicing pathway; essential protein that interacts with Mud2p and Prp40p, forming a bridge between the intron ends; also involved in nuclear retention of pre-mRNA
YPL105C	

0234	
YGR061C	[ADE6] Formylglycinamidine-ribonucleotide (FGAM)-synthetase, catalyzes a step in the 'de novo' purine nucleotide biosynthetic pathway 5'-phosphoribosylformyl glycinamidine synthetase Adenine requiring
YLR386W	[VAC14] Protein involved in regulated synthesis of PtdIns(3,5)P(2), in control of trafficking of some proteins to the vacuole lumen via the MVB, and in maintenance of vacuole size and acidity; activator of Fab1p Activator of Fab1p Null mutant has extremely enlarged vacuoles, is defective in vacuole membrane scission, has a vacuole acidification defect, and is defective in phosphatidylinositol 3,5 bisphosphate synthesis

0235	GO_TERM:[mRNA export from nucleus] P-Value:2.3e-05

YIR005W	[IST3] Component of the U2 snRNP, required for the first catalytic step of splicing and for spliceosomal assembly; interacts with Rds3p and is required for Mer1p-activated splicing Null mutant is viable but exhibits slow growth and a pre-mRNA splicing defect in vivo and in vitro. Deletion caused an immediate and exclusive accumulation of a particle consistent with a pre-mRNA/penta-snRNP complex.
YGL174W	[BUD13] Subunit of the RES complex, which is required for nuclear pre-mRNA retention and splicing; involved in bud-site selection; diploid mutants display a unipolar budding pattern instead of the wild-type bipolar pattern Null mutant is viable; diploid null mutants exhibit unipolar budding and elongate phenotype.
YLR016C	[PML1] Subunit of the RES complex, which is required for nuclear retention of unspliced pre-mRNAs; acts in the same pathway as Pml39p and Mlp1p

0236	GO_TERM:[cytosolic small ribosomal subunit (sensu Eukaryota)] P-Value:1.0e-02 OVERLAP:[cytoplasmic ribosomal small subunit] <500.40.20> SIZE:57
**YOR167C**	[RPS28A] Protein component of the small (40S) ribosomal subunit; nearly identical to Rps28Ap and has similarity to rat S28 ribosomal protein ribosomal protein S28A (S33A) (YS27)
YEL015W	[EDC3] Non-essential conserved protein of unknown function, plays a role in mRNA decapping by specifically affecting the function of the decapping enzyme Dcp1p; localizes to cytoplasmic mRNA processing bodies
YBR094W	
**YLR264W**	[RPS28B] Protein component of the small (40S) ribosomal subunit; nearly identical to Rps28Bp and has similarity to rat S28 ribosomal protein ribosomal protein S28B (S33B) (YS27)

0237	GO_TERM:[cytoplasmic mRNA processing body] P-Value:2.4e-11
YGL173C	[KEM1] Evolutionarily-conserved 5'-3' exonuclease component of cytoplasmic processing (P) bodies involved in mRNA decay; plays a role in microtubule-mediated processes, filamentous growth, ribosomal RNA maturation, and telomere maintenance 5'-3' exonuclease <u>K</u>ar1-1 nuclear-fusion-defect <u>E</u>nhancing <u>M</u>utation. Null mutant grows poorly. mutants exhibit aberrant mRNA turnover, are thought to be pleiotropic as a result; elongated morphology, defective in spindle-pole-body duplication/separation and telomere maintenance, benomyl hypersensitive, 10-20-fold elevation in chromosome loss, decreased mitotic recombination, inviable upon N starvation.
YOL149W	[DCP1] Subunit of the Dcp1p-Dcp2p decapping enzyme complex, which removes the 5' cap structure from mRNAs prior to their degradation; enhances the activity of catalytic subunit Dcp2p; regulated by DEAD box protein Dhh1p Null mutant is inviable in the FY1679 background, but viable, though grows slowly, in the CEN.PK141 background.
YJL124C	[LSM1] Lsm (Like Sm) protein; forms heteroheptameric complex (with Lsm2p, Lsm3p, Lsm4p, Lsm5p, Lsm6p, and Lsm7p) involved in degradation of cytoplasmic mRNAs absence of LSM1p leads to the accumulation of deadenylated capped mRNAs and also suppresses a PAB1 deletion.
YNL118C	[DCP2] Catalytic subunit of the Dcp1p-Dcp2p decapping enzyme complex, which removes the 5' cap structure from mRNAs prior to their degradation; member of the Nudix hydrolase family

0238	GO_TERM:[snRNP U6] P-Value:3.9e-20
YCR077C	[PAT1] Topoisomerase II-associated deadenylation-dependent mRNA-decapping factor; also required for faithful chromosome transmission, maintenance of rDNA locus stability, and protection of mRNA 3'-UTRs from trimming; functionally linked to Pab1p Null mutant is viable; slow growth rate, reduced fidelity of chromosome segregation during both mitosis and meiosis; slower rate of deadenylation-dependent decapping of mRNAs and transcript-specific effects on mRNA decay rates.
YMR268C	[PRP24] Splicing factor that reanneals U4 and U6 snRNPs during spliceosome recycling U4/U6 snRNP-associated protein defective in splicing
YER146W	[LSM5] Lsm (Like Sm) protein; part of heteroheptameric complexes (Lsm2p-7p and either Lsm1p or 8p): cytoplasmic Lsm1p complex involved in mRNA decay; nuclear Lsm8p complex part of U6 snRNP and possibly involved in processing tRNA, snoRNA, and rRNA snRNP protein
YJR022W	[LSM8] Lsm (Like Sm) protein; forms heteroheptameric complex (with Lsm2p, Lsm3p, Lsm4p, Lsm5p, Lsm6p, and Lsm7p) that is part of spliceosomal U6 snRNP and is also implicated in processing of pre-tRNA, pre-snoRNA, and pre-rRNA snRNP protein
YDR378C	[LSM6] Lsm (Like Sm) protein; part of heteroheptameric complexes (Lsm2p-7p and either Lsm1p or 8p): cytoplasmic Lsm1p complex involved in mRNA decay; nuclear Lsm8p complex part of U6 snRNP and possibly involved in processing tRNA, snoRNA, and rRNA snRNP protein
YNL147W	[LSM7] Lsm (Like Sm) protein; part of heteroheptameric complexes (Lsm2p-7p and either Lsm1p or 8p): cytoplasmic Lsm1p complex involved in mRNA decay; nuclear Lsm8p complex part of U6 snRNP and possibly involved in processing tRNA, snoRNA, and rRNA snRNP protein
YBL026W	[LSM2] Lsm (Like Sm) protein; part of heteroheptameric complexes (Lsm2p-7p and either Lsm1p or 8p): cytoplasmic Lsm1p complex involved in mRNA decay; nuclear Lsm8p complex part of U6 snRNP and possibly involved in processing tRNA, snoRNA, and rRNA snRNA-associated protein, Sm class
YLR438C-A	[LSM3] Lsm (Like Sm) protein; part of heteroheptameric complexes (Lsm2p-7p and either Lsm1p or 8p): cytoplasmic Lsm1p complex involved in mRNA decay; nuclear Lsm8p complex part of U6 snRNP and possibly involved in processing tRNA, snoRNA, and rRNA snRNP protein

0239	GO_TERM:[spliceosome complex] P-Value:2.6e-07
YKR022C	[NTR2] Essential protein that forms a dimer with Ntr1p; also forms a trimer, with Ntr2p and the DExD/H-box RNA helicase Prp43p, that is involved in spliceosome disassembly
YGR129W	[SYF2] Component of the spliceosome complex involved in pre-mRNA splicing; involved in regulation of cell cycle progression
YBR188C	[NTC20] Member of a complex, including Prp19p, that binds to the spliceosome; required for pre-mRNA splicing splicing factor Null mutant is viable. ntc20 ntc30 double mutant is very sick and accumulates pre-mRNA. Null mutant is synthetically lethal with prp19.
YJR050W	[ISY1] Component of the spliceosome complex involved in pre-mRNA splicing, auxiliary splicing factor that may modulate Syf1p activity and help optimize splicing; isy1 syf2 double mutation activates the spindle checkpoint, causing cell cycle arrest

0240	GO_TERM:[nuclear mRNA splicing, via spliceosome] P-Value:6.9e-63 OVERLAP:[Prp19p-associated complex] <440.30.10.10> SIZE:3
YMR125W	[STO1] Large subunit of the nuclear mRNA cap-binding protein complex, interacts with Npl3p to carry nuclear poly(A)+ mRNA to cytoplasm; also involved in nuclear mRNA degradation and telomere maintenance; orthologous to mammalian CBP80 Large subunit of the nuclear cap-binding protein complex defective growth on fermentable carbon sources and supression of top1-hpr1
YDR235W	[PRP42] U1 snRNP protein involved in splicing, required for U1 snRNP biogenesis; contains multiple tetriatricopeptide repeats U1 snRNP protein|shares 50% sequence similarity with Prp39p U1 snRNP protein and has multiple copies of the crn-like TPR motif Null mutant is inviable; prp39-1 is a point mutant that is temperature-sensitive for pre-mRNA splicing
YML046W	[PRP39] U1 snRNP protein involved in splicing, contains multiple tetriatricopeptide repeats RNA splicing factor|U1 snRNP protein Temperature-sensitive mutant arrests at the nonpermissive temperature and shows block in pre-mRNA splicing
YDL087C	[LUC7] Essential protein associated with the U1 snRNP complex; splicing factor involved in recognition of 5' splice site Null mutant is inviable; luc7 mutants exhibit synthetic lethality with the Cap-Binding Complex
YHR086W	[NAM8] RNA binding protein, component of the U1 snRNP protein; mutants are defective in meiotic recombination and in formation of viable spores, involved in the formation of DSBs through meiosis-specific splicing of MER2 pre-mRNA RNA-binding protein|U1 snRNP protein Null mutant is viable; defective in meiotic recombination, formation of viable spores, and formation of meiosis-specific double-strand breaks and crossover and noncrossover recombinants; overexpression suppresses mitochondrial splicing defects; impaired association of yeast-specific U1 snRNP proteins but hyperstabilized association of Snu65p/Prp42p with the U1 snRNP; affects in vivo splicing of introns with non-canonical 5'-splice sites; mutant contains a U1 snRNP with aberrant migration behaviour on native gels
YIL061C	[SNP1] Component of U1 snRNP required for mRNA splicing via spliceosome; may interact with poly(A) polymerase to regulate polyadenylation; homolog of human U1 70K protein U1snRNP 70K protein homolog Null mutant is inviable in some strain backgrounds and in other strain backgrounds, null mutant is viable, exhibits greatly increased doubling rates, severe temperature sensitivities, and defects in nuclear pre-mRNA splicing
YGR013W	[SNU71] Component of U1 snRNP required for mRNA splicing via spliceosome; yeast specific, no metazoan counterpart U1 snRNP protein
YDR240C	[SNU56] Component of U1 snRNP required for mRNA splicing via spliceosome; yeast specific, no metazoan counterpart; interacts with mRNA in commitment complex U1 snRNP protein Null mutant is inviable; mutation affects the in vitro formation of commitment complexes and spliceosomes and the in vivo splicing efficiency of certain introns.
YBR119W	[MUD1] U1 snRNP A protein, homolog of human U1-A; involved in nuclear mRNA splicing U1 snRNP A protein
YKL012W	[PRP40] U1 snRNP protein involved in splicing, interacts with the branchpoint-binding protein during the formation of the second commitment complex U1 snRNP protein Null mutant is inviable; temperature-sensitive mutants show a splicing defect
YLR298C	[YHC1] Component of the U1 snRNP complex required for pre-mRNA splicing; putative ortholog of human U1C protein, which is involved in formation of a complex between U1 snRNP and the pre-mRNA 5' splice site
YDR473C	[PRP3] Splicing factor, component of the U4/U6-U5 snRNP complex snRNP from U4/U6 and U5 snRNPs RNA synthesis defective
**YJL203W**	[PRP21] Subunit of the SF3a splicing factor complex, required for spliceosome assembly RNA splicing factor Null mutant is inviable, certain prp21 mutations are allele-specific suppressors of prp9 mutations
YDL209C	[CWC2] Protein involved in pre-mRNA splicing, component of a complex containing Cef1p; interacts with Prp19p; contains an RNA recognition motif; has similarity to S. pombe Cwf2p Null: required for pre-mRNA splicing
YPL151C	[PRP46] Splicing factor that is found in the Cef1p subcomplex of the spliceosome pre-mRNA splicing factor
**YPR101W**	[SNT309] Component of NineTeen complex (NTC) containing Prp19p involved in mRNA splicing, interacts physically and genetically with Prp19p Null mutant is viable, temperature sensitive, exhibits defects in splicing at elevated temperature; snt309 prp19 mutants are synthetically lethal
YML049C	[RSE1] Protein involved in pre-mRNA splicing; component of the pre-spliceosome; associates with U2 snRNA; involved in ER to Golgi transport An uncharacterized mutant allele grows slowly and exhibits defects in ER-to-Golgi transport and mRNA splicing.
YDL030W	[PRP9] Subunit of the SF3a splicing factor complex, required for spliceosome assembly; acts after the formation of the U1 snRNP-pre-mRNA complex RNA splicing factor

YMR240C	[CUS1] Protein required for assembly of U2 snRNP into the spliceosome, forms a complex with Hsh49p and Hsh155p U2 snRNP protein suppresses cold sensitivity of a U2 G53A cs mutant
YMR213W	[CEF1] Essential splicing factor; associated with Prp19p and the spliceosome, contains an N-terminal c-Myb DNA binding motif necessary for cell viability but not for Prp19p association, evolutionarily conserved and homologous to S. pombe Cdc5p protein complex component associated with the splicing factor Prp19p Null mutant is inviable, arrests in G2/M, exhibits abnormal nuclear morphologies. Essential for mRNA splicing.
YLR117C	[CLF1] Essential splicesome assembly factor; contains multiple tetratricopeptide repeat (TPR) protein-binding motifs and interacts specifically with many spliceosome components, may serve as a scaffold during splicesome assembly pre-mRNA splicing factor Null mutant is inviable; clf1 alleles show synthetic lethality with cdc40/prp17 and are defective in 5' splice site cleavage
YOR159C	[SME1] Core Sm protein Sm E; part of heteroheptameric complex (with Smb1p, Smd1p, Smd2p, Smd3p, Smx3p, and Smx2p) that is part of the spliceosomal U1, U2, U4, and U5 snRNPs; homolog of human Sm E human E core protein homolog
YKL173W	[SNU114] GTPase component of U5 snRNP involved in mRNA splicing via spliceosome; binds directly to U5 snRNA; proposed to be involved in conformational changes of the spliceosome; similarity to ribosomal translocation factor EF-2 U5 snRNP-specific protein related to EF-2 Null mutant is inviable; growth inhibitory when over-expressed; required for pre-mRNA splicing in vivo
YFL017W-A	[SMX2] Core Sm protein Sm G; part of heteroheptameric complex (with Smb1p, Smd1p, Smd2p, Smd3p, Sme1p, and Smx3p) that is part of the spliceosomal U1, U2, U4, and U5 snRNPs; homolog of human Sm G snRNP G protein (human Sm-G homolog)
YER172C	[BRR2] RNA-dependent ATPase RNA helicase involved in the facilitation and disruption of snRNA interactions, required for disruption of U4/U6 base-pairing in native snRNPs to activate the spliceosome for catalysis DEIH-box ATPase Null mutant is inviable; stabilized splicing intermediates which contain a mutant hammerhead cis-targeted ribozyme, decreased steady-state levels of endogneous mRNAs, increased ratio of pre-mRNA to mRNA of specific message(s); synthetic lethal with U2 mutants
YHR165C	[PRP8] Component of the U4/U6-U5 snRNP complex, involved in the second catalytic step of splicing U5 snRNP and spliceosome component Null mutant is inviable; synthetic lethal with U2 snRNA (LSR1); blocks pre-mRNA splicing in vivo and in vitro
**YLL036C**	[PRP19] Splicing factor associated with the spliceosome; contains a U-box, a motif found in a class of ubiquitin ligases RNA splicing factor Null mutant is inviable; pso4-1 mutants are sensitive to 8-methoxypsoralen, UV, MMS, and X-rays. prp19 ntc20 double mutants are inviable; prp19 isy1 double mutants are inviable.
YLR275W	[SMD2] Core Sm protein Sm D2; part of heteroheptameric complex (with Smb1p, Smd1p, Smd3p, Sme1p, Smx3p, and Smx2p) that is part of the spliceosomal U1, U2, U4, and U5 snRNPs; homolog of human Sm D2
YGR074W	[SMD1] Core Sm protein Sm D1; part of heteroheptameric complex (with Smb1p, Smd2p, Smd3p, Sme1p, Smx3p, and Smx2p) that is part of the spliceosomal U1, U2, U4, and U5 snRNPs; homolog of human Sm D1
YPR182W	[SMX3] Core Sm protein Sm F; part of heteroheptameric complex (with Smb1p, Smd1p, Smd2p, Smd3p, Sme1p, and Smx2p) that is part of the spliceosomal U1, U2, U4, and U5 snRNPs; homolog of human Sm F snRNP protein
YER029C	[SMB1] Core Sm protein Sm B; part of heteroheptameric complex (with Smd1p, Smd2p, Smd3p, Sme1p, Smx3p, and Smx2p) that is part of the spliceosomal U1, U2, U4, and U5 snRNPs; homolog of human Sm B and Sm B'
YLR147C	[SMD3] Core Sm protein Sm D3; part of heteroheptameric complex (with Smb1p, Smd1p, Smd2p, Sme1p, Smx3p, and Smx2p) that is part of the spliceosomal U1, U2, U4, and U5 snRNPs; homolog of human Sm D3 core snRNP protein Null mutant is inviable; depletion of Smd3p affects levels of U snRNAs and their cap modification; synthetic lethal with U2 snRNA (LSR1); blocks pre-mRNA splicing in vivo and in vitro
YPL213W	[LEA1] Component of U2 snRNP; disruption causes reduced U2 snRNP levels; physically interacts with Msl1p; invovled in telomere maintenance; putative homolog of human U2A' snRNP protein Null mutant is viable but grows slowly and is temperature sensitive. Null mutant also exhibits defects in spliceosome formation.

0241	GO_TERM:[deoxyribonucleoside metabolism] P-Value:5.2e-04
YJR056C	
YNR012W	[URK1] Uridine/cytidine kinase, component of the pyrimidine ribonucleotide salvage pathway that converts uridine into UMP and cytidine into CMP; involved in the pyrimidine deoxyribonucleotide salvage pathway, converting deoxycytidine into dCMP uridine kinase
YHL018W	
YER023W	[PRO3] Delta 1-pyrroline-5-carboxylate reductase, catalyzes the last step in proline biosynthesis delta 1-pyrroline-5-carboxylate reductase proline requiring
YBR176W	[ECM31] Ketopantoate hydroxymethyltransferase, required for pantothenic acid biosynthesis, converts 2-oxoisovalerate into 2-dehydropantoate
YLR245C	[CDD1] Cytidine deaminase; catalyzes the modification of cytidine to uridine in vitro but native RNA substrates have not been identified, localizes to both the nucleus and cytoplasm cytidine deaminase

YPR193C	[HPA2] Tetrameric histone acetyltransferase with similarity to Gcn5p, Hat1p, Elp3p, and Hpa3p; acetylates histones H3 and H4 in vitro and exhibits autoacetylation activity histone acetyltransferase Null mutant is viable and does not show any detectable phenotype
YJL218W	
YDR321W	[ASP1] Cytosolic L-asparaginase, involved in asparagine catabolism asparaginase I Aspartic acid requiring
YEL066W	[HPA3] D-Amino acid N-acetyltransferase, catalyzes N-acetylation of D-amino acids through ordered bi-bi mechanism in which acetyl-CoA is first substrate bound and CoA is last product liberated; similar to Hpa2p, acetylates histones weakly in vitro D-Amino acid N-acetyltransferase Null mutant is viable and does not show any detectable phenotype
YNL331C	[AAD14] Putative aryl-alcohol dehydrogenase with similarity to P. chrysosporium aryl-alcohol dehydrogenase; mutational analysis has not yet revealed a physiological role aryl-alcohol dehydrogenase (putative)
YBR252W	[DUT1] dUTPase, catalyzes the hydrolysis of dUTP to dUMP and PPi and thereby prevents the incorporation of uracil into DNA during replication dUTP pyrophosphatase
YML064C	[TEM1] GTP-binding protein of the ras superfamily involved in termination of M-phase; controls actomyosin and septin dynamics during cytokinesis GTP-binding protein|ras family Null mutant is inviable; net1-1 can suppress the lethality of a tem1 deletion by enabling Clb2p degradation and Sic1p accumulation; tem1-3 temperature sensitive mutants arrest in late anaphase with large buds, an elongated spindle and separated DNA; overexpression of CDC15, CDC5, SIC1, SPO12, and CDC14 can suppress the ts growth defects of tem1-3; overexpression of CLB2 is toxic to tem1-3 mutants at permissive temperature; deletion of cfi1 suppresss the temperature sensitivity of tem1-1 mutants
YPL070W	[MUK1] Protein of unknown function, localized to the cytoplasm; computational analysis of large-scale protein-protein interaction data suggests a possible role in transcriptional regulation

0242	GO_TERM:[Rho guanyl-nucleotide exchange factor activity] P-Value:1.6e-05
YDL203C	[YDL203C] Hypothetical protein a TRP/SEL-1 domain containing protein
YLR371W	[ROM2] GDP/GTP exchange protein (GEP) for Rho1p and Rho2p; mutations are synthetically lethal with mutations in rom1, which also encodes a GEP Null mutant is viable but shows temperature- and cold-sensitive growth defects at 37 and 11 degrees, increased sensitivity to benomyl, and elongated buds and abnormal mating projections at the permissive temperature; synthetically lethal with rom1
YBR011C	[IPP1] Cytoplasmic inorganic pyrophosphatase (PPase), catalyzes the rapid exchange of oxygens from Pi with water, highly expressed and essential for viability, active-site residues show identity to those from E. coli PPase inorganic pyrophosphatase
YLR425W	[TUS1] Guanine nucleotide exchange factor (GEF) that functions to modulate Rhop1 activity as part of the cell integrity signaling pathway; multicopy suppressor of tor2 mutation and ypk1 ypk2 double mutation; potential Cdc28p substrate Null mutant is viable; shows temperature sensitive growth above 37 degrees C, but no detectable secretory or endocytosis defect.
YPL066W	

0243	GO_TERM:[nucleus] P-Value:1.1e-01
YFL001W	[DEG1] Non-essential tRNA:pseudouridine synthase, introduces pseudouridines at position 38 or 39 in tRNA, important for maintenance of translation efficiency and normal cell growth, localizes to both the nucleus and cytoplasm Null mutant is viable, but demonstrates depressed growth rate
YDL153C	[SAS10] Component of the small (ribosomal) subunit (SSU) processosome required for pre-18S rRNa processing; essential nucleolar protein that, when overproduced, disrupts silencing U3 snoRNP protein Null mutant is inviable; derepresses HMR, HML and telomeres when overexpressed
YIL091C	
YJR119C	

0244	GO_TERM:[actin cortical patch] P-Value:5.7e-04
YBL047C	[EDE1] Key endocytic protein involved in a network of interactions with other endocytic proteins, binds membranes in a ubiquitin-dependent manner, may also bind ubiquitinated membrane-associated proteins
YDL161W	[ENT1] Epsin-like protein involved in endocytosis and actin patch assembly and functionally redundant with Ent2p; binds clathrin via a clathrin-binding domain motif at C-terminus Null mutant is viable, synthetically lethal with ent2 (YLR206w). ent1/2 double mutants have endocytosis and actin cytoskeleton defects.

0245	GO_TERM:[nucleus] P-Value:2.1e-01

YDR091C	[RLI1] Essential iron-sulfur protein required for ribosome biogenesis and translation initiation; facilitates binding of a multifactor complex (MFC) of translation initiation factors to the small ribosomal subunit; predicted ABC family ATPase Null mutant is inviable; overexpression of RLI1 from a galactose-inducible promoter has a moderate inhibitory effect on growth.
YJR067C	[YAE1] Essential protein of unknown function
YNL260C	
0246	GO_TERM:[translation initiation factor activity] P-Value:1.3e-36 OVERLAP:[eIF3] <500.10.40> SIZE:7
YIL131C	[FKH1] Transcription factor of the forkhead family that regulates the cell cycle and pseudohyphal growth; also involved in chromatin silencing at HML and HMR forkhead protein
YER025W	[GCD11] Gamma subunit of the translation initiation factor eIF2, involved in the identification of the start codon; binds GTP when forming the ternary complex with GTP and tRNAi-Met translational initiation factor eIF-2 gamma subunit Null mutant is inviable, gcd11 mutants have slower growth rate under nonstarvation conditions
YNL265C	[IST1] Putative translation initiation factor, as suggested by computational analysis of large-scale protein-protein interaction data
YJR007W	[SUI2] Alpha subunit of the translation initiation factor eIF2, involved in the identification of the start codon; phosphorylation of Ser51 is required for regulation of translation by inhibiting the exchange of GDP for GTP Translation initiation factor eIF-2 alpha subunit suppression of initiator codon mutations
YPL237W	[SUI3] Beta subunit of the translation initiation factor eIF2, involved in the identification of the start codon; proposed to be involved in mRNA binding translation initiation factor eIF-2 beta subunit suppression of initiator codon mutations
YLR291C	[GCD7] Beta subunit of the translation initiation factor eIF2B, the guanine-nucleotide exchange factor for eIF2; activity subsequently regulated by phosphorylated eIF2; first identified as a negative regulator of GCN4 expression negative regulator of GCN4 expression|translation initiation factor eIF2B subunit Null mutant is inviable; non-null mutants exhibit an increase in GCN4 translation
YDR211W	[GCD6] Catalytic epsilon subunit of the translation initiation factor eIF2B, the guanine-nucleotide exchange factor for eIF2; activity subsequently regulated by phosphorylated eIF2; first identified as a negative regulator of GCN4 expression translation initiation factor eIF-2B epsilon subunit Null mutant is inviable; non-null mutations increase GCN4 translation
YGR083C	[GCD2] Delta subunit of the translation initiation factor eIF2B, the guanine-nucleotide exchange factor for eIF2; activity subsequently regulated by phosphorylated eIF2; first identified as a negative regulator of GCN4 expression 71 kDa subunit (delta)|translation initiation factor eIF2B subunit|translational repressor of GCN4 protein Null mutant is inviable; resistance to 5-methyltryptophan, 5-fluorotryptophan and canavanine; override requirement for GCN2 and GCN3 gene products for derepression of GCN4 constitutive derepression and slow growth; temperature sensitive for growth
YKR026C	[GCN3] Alpha subunit of the translation initiation factor eIF2B, the guanine-nucleotide exchange factor for eIF2; activity subsequently regulated by phosphorylated eIF2; first identified as a positive regulator of GCN4 expression eIF2B 34 kDa alpha subunit null mutants fail to derepress amino acid-regulated genes under conditions of amino acid starvation
YOR260W	[GCD1] Gamma subunit of the translation initiation factor eIF2B, the guanine-nucleotide exchange factor for eIF2; activity subsequently regulated by phosphorylated eIF2; first identified as a negative regulator of GCN4 expression gamma subunit|negative regulator in the general control of amino acid biosynthesis|translation initiation factor eIF2B subunit affect growth rate under nonstarvation conditions
**YNL062C**	[GCD10] Subunit of tRNA (1-methyladenosine) methyltransferase with Gcd14p, required for the modification of the adenine at position 58 in tRNAs, especially tRNAi-Met; first identified as a negative regulator of GCN4 expression RNA-binding protein|subunit of tRNA(1-methyladenosine) methyltransferase, along with Gcd14p Null mutant is inviable. There are mutants available that show constitutive HIS4 transcription and slow growth
YOL087C	
**YNL244C**	[SUI1] Translation initiation factor eIF1; component of a complex involved in recognition of the initiator codon; modulates translation accuracy at the initiation phase translation initiation factor eIF1
YPR041W	[TIF5] Translation initiation factor eIF-5; N-terminal domain functions as a GTPase-activating protein to mediate hydrolysis of ribosome-bound GTP; C-terminal domain is the core of ribosomal preinitiation complex formation Translation initiation factor eIF5
**YOR361C**	[PRT1] Subunit of the core complex of translation initiation factor 3(eIF3), essential for translation; part of a subcomplex (Prt1p-Rpg1p-Nip1p) that stimulates binding of mRNA and tRNA(i)Met to ribosomes translation initiation factor eIF3 subunit
YLR192C	[HCR1] Dual function protein involved in translation initiation as a substoichiometric component of eukaryotic translation initiation factor 3 (eIF3) and required for processing of 20S pre-rRNA; binds to eIF3 subunits Rpg1p and Prt1p and 18S rRNA Substoichiometric component of eukaryotic translation initiation factor 3 (eIF3)
**YBR079C**	[RPG1] Subunit of the core complex of translation initiation factor 3(eIF3), essential for translation; part of a subcomplex (Prt1p-Rpg1p-Nip1p) that stimulates binding of mRNA and tRNA(i)Met to ribosomes translation initiation factor eIF3 subunit Null mutant is inviable; temperature sensitive mutant arrests in G1 phase
**YMR146C**	[TIF34] Subunit of the core complex of translation initiation factor 3(eIF3), which is essential for translation translation initiation factor eIF3 subunit
**YDR429C**	[TIF35] Subunit of the core complex of translation initiation factor 3(eIF3), which is essential for translation translation initiation factor eIF3 subunit

**YMR309C**	[NIP1] Subunit of the eukaryotic translation initiation factor 3 (eIF3), involved in the assembly of preinitiation complex and start codon selection translation initiation factor eIF3 subunit Null mutant is inviable; nip1-1 is a temperature-sensitive mutant defective in nuclear transport

0247	GO_TERM:[polysome] P-Value:1.1e-07 OVERLAP:[Nonsense-mediated mRNA decay pathway complex] <300> SIZE:3
YDR172W	[SUP35] Translation termination factor eRF3; altered protein conformation creates the [PSI(+)] prion, a dominant cytoplasmically inherited protein aggregate that alters translational fidelity and creates a nonsense suppressor phenotype translation termination factor eRF3 accumulation of large budded cells and substantial arrest of DNA synthesis at the nonpermissive temperature (arrests at G(sub)1/S transition); omnipotent suppressor of nonsense mutations
**YMR080C**	[NAM7] ATP-dependent RNA helicase of the SFI superfamily, required for nonsense mediated mRNA decay and for efficient translation termination at nonsense codons; involved in telomere maintenance helicase (putative) Null mutant is viable, exhibits impairment in respiratory growth that is exacerbated by low temperatures; exhibits stabilization of nonsense-containing mRNAs which leads to a nonsense suppression phenotype
**YGR072W**	[UPF3] Component of the nonsense-mediated mRNA decay (NMD) pathway, along with Nam7p and Nmd2p; involved in decay of mRNA containing nonsense codons; involved in telomere maintenance Null mutant is viable but shows increased accumulation of mRNA containing a premature stop codon due to mRNA stabilization
**YHR077C**	[NMD2] Protein involved in the nonsense-mediated mRNA decay (NMD) pathway; interacts with Nam7p and Upf3p; involved in telomere maintenance Null mutant is viable, exhibits stabilization of nonsense-containing mRNAs

0248	GO_TERM:[steroid binding] P-Value:6.5e-05
YBR143C	[SUP45] Polypeptide release factor involved in translation termination; mutant form acts as a recessive omnipotent suppressor eRF1 (eukaryotic Release Factor 1) homolog The null mutant is inviable. Other mutant alleles produce a variety of phenotypes which can include: omnipotent nonsense suppression, osmotic sensitivity, benomyl sensitivity, paromomycin sensitivity, and novobiocin resistance.
YML068W	[ITT1] Protein that modulates the efficiency of translation termination, interacts with translation release factors eRF1 (Sup45p) and eRF3 (Sup35p) in vitro, contains a zinc finger domain characteristic of the TRIAD class of proteins
YAR042W	[SWH1] Protein similar to mammalian oxysterol-binding protein; contains ankyrin repeats; localizes to the Golgi and the nucleus-vacuole junction
YDL019C	[OSH2] Member of an oxysterol-binding protein family with seven members in S. cerevisiae; family members have overlapping, redundant functions in sterol metabolism and collectively perform a function essential for viability
YER120W	[SCS2] Integral ER membrane protein that regulates phospholipid metabolism via an interaction with the FFAT motif of Opi1p, also involved in telomeric silencing, disruption causes inositol auxotrophy above 34 degrees C, VAP homolog

0249	GO_TERM:[binding] P-Value:7.5e-02 OVERLAP:[eIF4A] <500.10.50> SIZE:2
YDR129C	[SAC6] Fimbrin, actin-bundling protein; cooperates with Scp1p (calponin/transgelin) in the organization and maintenance of the actin cytoskeleton actin filament bundling protein|fimbrin homolog suppressor of actin mutations, abnormal actin structures, defective morphogenesis
**YJL138C**	[TIF2] Translation initiation factor eIF4A, identical to Tif1p; DEA(D/H)-box RNA helicase that couples ATPase activity to RNA binding and unwinding; forms a dumbbell structure of two compact domains connected by a linker; interacts with eIF4G translation initiation factor eIF4A subunit viable, tif1tif2 double mutant is lethal
YLR380W	[CSR1] Phosphatidylinositol transfer protein with a potential role in lipid turnover; interacts specifically with thioredoxin peroxidase (Tsa2p) and may have a role in oxidative stress resistance

0250	GO_TERM:[exosome (RNase complex)] P-Value:1.2e-36 OVERLAP:[Exosome complex] <440.12.10> SIZE:7
YDR083W	[RRP8] Nucleolar protein involved in rRNA processing, pre-rRNA cleavage at site A2; also involved in telomere maintenance; mutation is synthetically lethal with a gar1 mutation nucleolar protein required for efficient processing of pre-rRNA at site A2; methyltransferase homolog
YNR024W	
YHR081W	[LRP1] Substrate-specific nuclear cofactor for exosome activity in the processing of stable RNAs; required for telomere length maintenance; homolog of mammalian nuclear matrix protein C1D involved in regulation of DNA repair and recombination
**YDR280W**	[RRP45] Protein involved in rRNA processing; component of the exosome 3->5 exonuclease complex 3'->5' exoribonuclease Null mutant is inviable; mutant is defective in 3' processing of 5.8S rRNA

**YOL021C**	[DIS3] Nucleolar exosome component, involved in rRNA processing and RNA degradation, binds Gsp1p/Ran and enhances the GEF activity of Srm1p, implicated in mitotic control, homologous to the E. coli RNase R of the RNase II family 3'-5' exoribonuclease complex subunit
YNL232W	[CSL4] Subunit of the exosome, which is an essential complex present in both nucleus and cytoplasm that mediates RNA processing and degradation Null mutant is inviable, csl4-1 exhibits double mutant inviability in combination with cbf1(cep1) deletion mutants
YOR001W	[RRP6] Exonuclease component of the nuclear exosome; contributes to the quality-control system that retains and degrades aberrant mRNAs in the nucleus Null mutant is viable, heat sensitive; other mutants show a 5.8S rRNA 3' end formation defect
**YGR095C**	[RRP46] Protein involved in rRNA processing; component of the exosome 3->5 exonuclease complex 3'->5' exoribonuclease Null mutant is inviable; mutant is defective in 3' processing of 5.8S rRNA
**YCR035C**	[RRP43] Protein involved in rRNA processing; component of the exosome 3->5 exonuclease complex with Rrp41p, Rrp42p, Rrp4p and Dis3p; required for efficient maturation of 5.8S, 18S and 25S rRNA exosome 3->5 exoribonuclease complex component with Rrp4p, Rrp41p, Rrp42p and Dis3p (Rrp44p) Null mutant is inviable in some strain backgrounds; rrp43 mutants are defective in 3' processing of 5.8S RNA
**YHR069C**	[RRP4] Protein involved in rRNA processing; component of the exosome 3->5 exonuclease complex with Rrp41p, Rrp42p, Rrp43p and Dis3p 3'-5' exoribonuclease|3'-5' exoribonuclease complex component with Rrp4p, Rrp41p, Rrp42p and Dis3p (Rrp44p) Null is inviable; Defective in 3' processing of 5.8S rRNA
**YDL111C**	[RRP42] Protein involved in rRNA processing; component of the exosome 3->5 exonuclease complex with Rrp4p, Rrp41p, Rrp43p and Dis3p exosome 3->5 exoribonuclease complex component with Rrp4p, Rrp41p, Rrp43p and Dis3p (Rrp44p) Null mutant is inviable, rrp42 mutants are defective in 3' processing of 5.8S RNA
**YGR195W**	[SKI6] 3'-to-5' phosphorolytic exoribonuclease that is a subunit of the exosome; required for 3' processing of the 5.8S rRNA; involved in 3' to 5' mRNA degradation and translation inhibition of non-poly(A) mRNAs RNAse PH homolog Null mutant is inviable; mutants show superkiller phenotype, improved translation of non-poly(A) mRNA, abnormal 60S ribosomal subunits and defective 3' processing of 5.8S rRNA; a Tn3 insertion into this gene causes hypersensitivity to the cell surface polymer perturbing agent calcofluor white
YOL142W	[RRP40] Protein involved in rRNA processing; component of the exosome 3->5 exonuclease complex 3' -> 5' exoribonuclease The null mutant is inviable and defective in 3' processing of 5.8S rRNA
YGR158C	[MTR3] 3'5' exoribonuclease, exosome subunit; nucleolar protein involved in export of mRNA and ribosomal subunits; homologous to the E. coli exonuclease RNase PH null is inviable; mutant with mtr3-1 allele has defects in both mRNA transport and in rRNA synthesis/processing, with polyA+ mRNA accumulated in the nucleolus
YOR076C	[SKI7] Antiviral adaptor protein that mediates interactions via its N-terminus between the exosome and Ski complex (Ski2p, Ski3p, Ski8p) which operate in the 3'-to-5' mRNA-decay pathway; cytoplasmic protein required for degrading nonstop mRNAs GTPase (putative)

0251	GO_TERM:[mRNA binding] P-Value:1.3e-05 OVERLAP:[pre mRNA3'-end processing factor CFI] <440.10.10> SIZE:5
YGR054W	
YNL016W	[PUB1] Poly(A)+ RNA-binding protein, abundant mRNP-component protein hypothesized to bind a pool of non-translatable mRNAs; not reported to associate with polyribosomes poly(A) binding protein
YDR432W	[NPL3] RNA-binding protein that carries poly(A)+ mRNA from the nucleus into the cytoplasm; phosphorylation by Sky1p in the cytoplasm may promote release of mRNAs contains RNA recognition motif|nuclear shuttling protein Null mutant is inviable, npl3 mutants are temperature-sensitive for growth, but do not exhibit a defect in localization of nuclear proteins
**YOL123W**	[HRP1] Subunit of cleavage factor I, a five-subunit complex required for the cleavage and polyadenylation of pre-mRNA 3' ends; RRM-containing heteronuclear RNA binding protein and hnRNPA/B family member that binds to poly (A) signal sequences cleavage and polyadenylation factor CF I component involved in pre-mRNA 3'-end processing Null mutant is inviable; mutants can suppress temperature-sensitive alleles of npl3 (but not npl3 null mutants)
YBR034C	[HMT1] Nuclear SAM-dependent mono- and asymmetric arginine dimethylating methyltransferase that modifies hnRNPs, including Npl3p and Hrp1p, thus facilitating nuclear export of these proteins; required for viability of npl3 mutants arginine methyltransferase|mono- and asymmetrically dimethylating enzyme Null mutant is viable, hmt1 npl3-1 mutants are inviable
YGL122C	[NAB2] Nuclear polyadenylated RNA-binding protein; autoregulates mRNA levels; related to human hnRNPs; has nuclear localization signal sequence that binds to Kap104p; required for poly(A) tail length control and nuclear mRNA export polyadenylated RNA binding protein

0252	GO_TERM:[rRNA catabolism] P-Value:1.2e-13 OVERLAP:[rRNA splicing] <440.30.20> SIZE:24
YJL050W	[MTR4] Dead-box family ATP dependent helicase required for mRNA export from the nucleus; co-factor of the exosome complex, required for 3' end formation of 5.8S rRNA RNA helicase

YNL299W	[TRF5] Poly (A) polymerase involved in nuclear RNA quality control based on: homology with Trf4p, genetic interactions with TRF4 mutants, physical interaction with Mtr4p (TRAMP subunit), and by direct assay; disputed role as a sigma DNA polymerase DNA polymerase sigma
YOL115W	[PAP2] Catalytic subunit of TRAMP (Trf4/Pap2p-Mtr4p-Air1p/2p), a nuclear poly (A) polymerase complex involved in RNA quality control; catalyzes polyadenylation of unmodified tRNAs, and snoRNA and rRNA precursors; disputed role as a DNA polymerase DNA polymerase sigma|poly(A) polymerase
YDL175C	[AIR2] RING finger protein that interacts with the arginine methyltransferase Hmt1p; may regulate methylation of Npl3p, which modulates Npl3p function in mRNA processing and export; has similarity to Air1p
YIL079C	[AIR1] RING finger protein that interacts with the arginine methyltransferase Hmt1p to regulate methylation of Npl3p, which modulates Npl3p function in mRNA processing and export; has similarity to Air2p
YML117W	[NAB6] Putative RNA-binding protein, based on computational analysis of large-scale protein-protein interaction data
**YMR239C**	[RNT1] RNAase III; cleaves a stem-loop structure at the 3' end of U2 snRNA to ensure formation of the correct U2 3' end ribonuclease III
YLR430W	[SEN1] Nuclear protein, putative helicase required for processing of tRNAs, rRNAs, and small nuclear RNAs; potential Cdc28p substrate nuclear-localized tRNA splicing complex component
YNL251C	[NRD1] RNA-binding protein that interacts with the C-terminal domain of the RNA polymerase II large subunit (Rpo21p), required for transcription termination and 3' end maturation of nonpolyadenylated RNAs
YPL190C	[NAB3] Single stranded DNA binding protein; acidic ribonucleoprotein; required for termination of non-poly(A) transcripts and efficient splicing; interacts with Nrd1p polyadenylated RNA binding protein|polyadenylated single strand DNA-binding protein null is inviable; overexpression reduces the expression of the G1 cyclin CLN3

0253	GO_TERM:[phosphatase regulator activity] P-Value:2.9e-04 OVERLAP:[Serine/threonine phoshpoprotein phosphatase] <450> SIZE:6
**YER054C**	[GIP2] Putative regulatory subunit of the protein phosphatase Glc7p, involved in glycogen metabolism; contains a conserved motif (GVNK motif) that is also found in Gac1p, Pig1p, and Pig2p
YER158C	
YAR014C	[BUD14] Protein involved in bud-site selection, Bud14p-Glc7p complex functions as a cortical regulator of dynein; diploid mutants display a random budding pattern instead of the wild-type bipolar pattern
YML131W	
YOL154W	[ZPS1] Putative GPI-anchored protein; transcription is induced under low-zinc conditions, as mediated by the Zap1p transcription factor, and at alkaline pH

0254	GO_TERM:[protein deubiquitination] P-Value:2.9e-03
YHR039C	[MSC7] Protein of unknown function, green fluorescent protein (GFP)-fusion protein localizes to the endoplasmic reticulum; msc7 mutants are defective in directing meiotic recombination events to homologous chromatids
YAR010C	
YNR051C	[BRE5] Ubiquitin protease cofactor, forms deubiquitination complex with Ubp3p that coregulates anterograde and retrograde transport between the endoplasmic reticulum and Golgi compartments; null is sensitive to brefeldin A
YDL066W	[IDP1] Mitochondrial NADP-specific isocitrate dehydrogenase, catalyzes the oxidation of isocitrate to alpha-ketoglutarate; not required for mitochondrial respiration and may function to divert alpha-ketoglutarate to biosynthetic processes NADP-dependent isocitrate dehydrogenase
YER151C	[UBP3] Ubiquitin-specific protease that interacts with Bre5p to co-regulate anterograde and retrograde transport between endoplasmic reticulum and Golgi compartments; inhibitor of gene silencing; cleaves ubiquitin fusions but not polyubiquitin ubiquitin-specific protease Null mutant is viable. Null yuh1 ubp1 ubp2 ubp3 quadruple mutants are viable and retain the ability to deubiquitinate ubiquitin fusions. Deletion of the UBP3 gene results in markedly improved silencing of genes inserted either near a telomere or at one of the silent mating type loci.
YMR173W	[DDR48] DNA damage-responsive protein, expression is increased in response to heat-shock stress or treatments that produce DNA lesions; contains multiple repeats of the amino acid sequence NNNDSYGS flocculent specific protein Null mutant is viable, displays reduced spontaneous mutation rate
YOL109W	[ZEO1] Peripheral membrane protein of the plasma membrane that interacts with Mid2p; regulates the cell integrity pathway mediated by Pkc1p and Slt2p Null mutant is viable and exhibits slow growth in galactose

0255	GO_TERM:[cellular response to phosphate starvation] P-Value:4.2e-05 OVERLAP:[cytoplasmic ribosomal large subunit] <500.40.10> SIZE:81
**YDL191W**	[RPL35A] Protein component of the large (60S) ribosomal subunit, identical to Rpl35Bp and has similarity to rat L35 ribosomal protein ribosomal protein L35A
YFR034C	[PHO4] Basic helix-loop-helix (bHLH) transcription factor; binds cooperatively with Pho2p to the PHO5 promoter; function is regulated by phosphorylation at multiple sites and by phosphate availability myc-family transcription factor
**YDL136W**	[RPL35B] Protein component of the large (60S) ribosomal subunit, identical to Rpl35Ap and has similarity to rat L35 ribosomal protein ribosomal protein L35B
YDL106C	[PHO2] Homeobox transcription factor; regulatory targets include genes involved in phosphate metabolism; binds cooperatively with Pho4p to the PHO5 promoter; phosphorylation of Pho2p facilitates interaction with Pho4p homeobox transcription factor|positive regulator of PHO5 and other genes The null mutant is viable but unable to sporulate. Many genes regulated by GRF10 are expressed at non-wild type levels in GRF10 null mutants.
YMR045C	

0256	OVERLAP:[cytoplasmic ribosomal large subunit] <500.40.10> SIZE:81
**YER117W**	[RPL23B] Protein component of the large (60S) ribosomal subunit, identical to Rpl23Ap and has similarity to E. coli L14 and rat L23 ribosomal proteins ribosomal protein L23B (L17aB) (YL32)
YDR361C	[BCP1] Essential protein involved in nuclear export of Mss4p, which is a lipid kinase that generates phosphatidylinositol 4,5-biphosphate and plays a role in actin cytoskeleton organization and vesicular transport
YPL208W	[RKM1] SET-domain lysine-N-methyltransferase, catalyzes the formation of dimethyllysine residues on the large ribsomal subunit protein L23a (RPL23A and RPL23B) methyltransferase

0257	GO_TERM:[2-isopropylmalate synthase activity] P-Value:4.9e-06
YJR041C	[URB2] Nucleolar protein required for normal metabolism of the rRNA primary transcript, proposed to be involved in ribosome biogenesis
YKL214C	[YRA2] Member of the REF (RNA and export factor binding proteins) family; when overexpressed, can substitute for the function of Yra1p in export of poly(A)+ mRNA from the nucleus
YOL144W	[NOP8] Nucleolar protein required for 60S ribosomal subunit biogenesis
YNR038W	[DBP6] Essential protein involved in ribosome biogenesis; putative ATP-dependent RNA helicase of the DEAD-box protein family RNA helicase (putative) Null mutant is inviable; Dbp6p depletion leads to decreased production of the 27S and 7S precursors, resulting in a depletion of the mature 25S and 5.8S rRNAs
YLR221C	[RSA3] Protein with a likely role in ribosomal maturation, required for accumulation of wild-type levels of large (60S) ribosomal subunits; binds to the helicase Dbp6p in pre-60S ribosomal particles in the nucleolus
YNL104C	[LEU4] Alpha-isopropylmalate synthase (2-isopropylmalate synthase); the main isozyme responsible for the first step in the leucine biosynthesis pathway alpha-isopropylmalate synthase (2-isopropylmalate synthase) Null mutant is viable, Leu+
YOR108W	[LEU9] Alpha-isopropylmalate synthase II (2-isopropylmalate synthase), catalyzes the first step in the leucine biosynthesis pathway; the minor isozyme, responsible for the residual alpha-IPMS activity detected in a leu4 null mutant alpha-isopropylmalate synthase (2-isopropylmalate synthase)

0258	GO_TERM:[transcription export complex] P-Value:4.0e-15 OVERLAP:[Ctk1p complex] <133.50> SIZE:3
**YKL139W**	[CTK1] Catalytic (alpha) subunit of C-terminal domain kinase I (CTDK-I), which phosphorylates the C-terminal repeated domain of the RNA polymerase II large subunit (Rpo21p) to affect both transcription and pre-mRNA 3' end processing kinase subunit of RNA polymerase II carboxy-terminal domain kinase I Null mutations in each of the CTK1, CTK2, and CTK3 genes cause slow growth, cold-sensitivity, flocculence, and enlarged cell size.
**YJL006C**	[CTK2] Beta subunit of C-terminal domain kinase I (CTDK-I), which phosphorylates the C-terminal repeated domain of the RNA polymerase II large subunit (Rpo21p) to affect both transcription and pre-mRNA 3' end processing; has similarity to cyclins RNA polymerase II C-terminal domain kinase beta subunit, similar to cyclin Null mutations in each of the CTK1, CTK2, and CTK3 genes cause slow growth, cold-sensitivity, flocculence, and enlarged cell size.
**YML112W**	[CTK3] Gamma subunit of C-terminal domain kinase I (CTDK-I), which phosphorylates the C-terminal repeated domain of the RNA polymerase II large subunit (Rpo21p) to affect both transcription and pre-mRNA 3' end processing RNA polymerase II C-terminal domain kinase gamma subunit, similar to cyclin-dependent kinase Null mutations in each of the CTK1, CTK2, and CTK3 genes cause slow growth, cold-sensitivity, flocculence, and enlarged cell size.
YPL127C	[HHO1] Histone H1, a linker histone required for nucleosome packaging at restricted sites; suppresses DNA repair involving homologous recombination; not required for telomeric silencing, basal transcriptional repression, or efficient sporulation histone H1 Null mutant is viable; other phenotype: Increased basal expression of a CYC1-lacz reporter gene; nuclear localization of a Hho1-GFP fusion protein

YBR020W	[GAL1] Galactokinase, phosphorylates alpha-D-galactose to alpha-D-galactose-1-phosphate in the first step of galactose catabolism; expression regulated by Gal4p galactokinase Null mutant is viable and cannot utilize galactose.
YJR032W	[CPR7] Peptidyl-prolyl cis-trans isomerase (cyclophilin), catalyzes the cis-trans isomerization of peptide bonds N-terminal to proline residues; binds to Hsp82p and contributes to chaperone activity cyclophilin 40|peptidyl-prolyl cis-trans isomerase (PPIase)
YOR191W	[RIS1] Member of the SWI/SNF family of DNA-dependent ATPases, plays a role in antagonizing silencing during mating-type switching, contains an N-terminal domain that interacts with Sir4p and a C-terminal SNF2 domain SWI2/SNF2 DNA-dependent ATPase family member (putative) Null mutant is viable but shows slower mating type switching; interferes with silencing when overexpressed
YDR138W	[HPR1] Subunit of THO/TREX complexes that couple transcription elongation with mitotic recombination and with mRNA metabolism and export, subunit of an RNA Pol II complex; regulates lifespan; involved in telomere maintenance; similar to Top1p Increased intrachromosomal recombination
YCL011C	[GBP2] Poly(A+) RNA-binding protein, involved in the export of mRNAs from the nucleus to the cytoplasm; similar to Hrb1p and Npl3p; also binds single-stranded telomeric repeat sequence in vitro contains RNA recognition motifs Mutation alters the distribution of Rap1p, a telomere-associated protein, but has no effect on telomere length or telomere position
YNL004W	[HRB1] Poly(A+) RNA-binding protein, involved in the export of mRNAs from the nucleus to the cytoplasm; similar to Gbp2p and Npl3p hypothetical RNA-binding protein
YDL084W	[SUB2] Component of the TREX complex required for nuclear mRNA export; DEAD-box RNA helicase involved in early and late steps of spliceosome assembly; homolog of the human splicing factor hUAP56 ATP-dependent RNA helicase Null mutant is inviable; sub2 allele suppresses cold-sensitive snRNP phenotype of brr1-1
YNL139C	[RLR1] Subunit of the THO complex, which is required for efficient transcription elongation and involved in transcriptional elongation-associated recombination; required for LacZ RNA expression from certain plasmids Null mutant is viable but shows poor growth and a temperature-sensitive phenotype.Increased frequencies of recombination between direct repeats (>1000-fold above wild-type levels) that is linked to transcriptional elongation defects. General defects in RNA polII transcription. Incapacity to transcribe lacZ. Overexpression of RLR1 suppresses the ts phenotype and the incapacity to transcribe lacZ sequences of hpr1-delta mutants
YNL253W	[TEX1] Protein involved in mRNA export, component of the transcription export (TREX) complex
YHR167W	[THP2] Subunit of the THO complex, which connects transcription elongation and mitotic recombination, and of the TREX complex, which is recruited to activated genes and couples transcription to mRNA export; involved in telomere maintenance null mutant is viable, hyper-recombination between direct repeats dependent on transcription elongation, transcription elongation impairment, inability to properly transcribe lacZ sequences
YML062C	[MFT1] Subunit of the THO complex, which is a nuclear complex comprised of Hpr1p, Mft1p, Rlr1p, and Thp2p, that is involved in transcription elongation and mitotic recombination; involved in telomere maintenance mitochondrial targeting protein

0259	GO_TERM:[molecular_function] P-Value:1.7e-01
YGR272C	
YNL255C	[GIS2] Protein with seven cysteine-rich CCHC zinc-finger motifs, similar to human CNBP, proposed to be involved in the RAS/cAMP signaling pathway

0260	GO_TERM:[NatA complex] P-Value:2.2e-06 OVERLAP:[Protein N-acetyltransferase] <370> SIZE:2
**YHR013C**	[ARD1] Subunit of the N-terminal acetyltransferase NatA (Nat1p, Ard1p, Nat5p); N-terminally acetylates many proteins, which influences multiple processes such as the cell cycle, heat-shock resistance, mating, sporulation, and telomeric silencing N alpha-acetyltransferase major subunit|complexes with Nat1p
YOR253W	[NAT5] Subunit of the N-terminal acetyltransferase NatA (Nat1p, Ard1p, Nat5p); N-terminally acetylates many proteins, which influences multiple processes such as the cell cycle, heat-shock resistance, mating, sporulation, and telomeric silencing N-acetyltransferase

0261	GO_TERM:[catalytic activity] P-Value:7.3e-02
YFR053C	[HXK1] Hexokinase isoenzyme 1, a cytosolic protein that catalyzes phosphorylation of glucose during glucose metabolism; expression is highest during growth on non-glucose carbon sources; glucose-induced repression involves the hexokinase Hxk2p hexokinase I (PI) (also called hexokinase A) Null mutant is viable, is able to ferment fructose, and has little or no effect on glucose repression; hxk1, hxk2 double null mutant cannot ferment fructose and fails to show glucose repression at SUC2, CYC1, GAL10
YOR081C	[TGL5] Triacylglycerol lipase involved in TAG mobilization; localizes to lipid particles; potential Cdc28p substrate triacylglycerol lipase

0262	GO_TERM:[transcription termination from Pol II promoter, RNA polymerase(A) coupled] P-Value:4.8e-08 OVERLAP:[rRNA splicing] <440.30.20> SIZE:24
YGL246C	[RAI1] Nuclear protein that binds to and stabilizes the exoribonuclease Rat1p, required for pre-rRNA processing

YDR289C	[RTT103] Protein that interacts with exonuclease Rat1p and Rai1p and plays a role in transcription termination by RNA polymerase II, has an RPR domain (carboxy-terminal domain interacting domain); also involved in regulation of Ty1 transposition Gene disruption causes Ty1 hypertransposition phenotype
**YOR048C**	[RAT1] Nuclear 5' to 3' single-stranded RNA exonuclease, involved in RNA metabolism, including rRNA and snRNA processing as well as mRNA transcription termination 5'-3' exoribonuclease

0263	GO_TERM:[translation repressor activity] P-Value:1.3e-10
YPR189W	[SKI3] Protein involved in exosome mediated 3' to 5' mRNA degradation and translation inhibition of non-poly(A) mRNAs; forms complex with Ski2p and Ski8p; required for repressing propagation of dsRNA viruses antiviral protein that blocks translation of un-polyadenylated mRNAs
YLR398C	[SKI2] Putative RNA helicase, involved in exosome mediated 3' to 5' mRNA degradation and translation inhibition of non-poly(A) mRNAs; forms complex with Ski3p and Ski8p; required for repressing propagation of dsRNA viruses antiviral protein|helicase (putative) Null mutant is viable; SKI2 is essential in cells carrying M dsRNA
YGL213C	[SKI8] Protein involved in exosome mediated 3' to 5' mRNA degradation and translation inhibition of non-poly(A) mRNAs as well as double-strand break formation during meiotic recombination; required for repressing propagation of dsRNA viruses antiviral protein|mRNA is induced early in meiosis Null mutant is viable; rec103 is rescued by spo13 and is episatic to rad52 spo13, suggesting it is an early recombination gene
YKL023W	

0264	GO_TERM:[cytosolic ribosome (sensu Eukaryota)] P-Value:3.3e-03 OVERLAP:[cytoplasmic ribosomal small subunit] <500.40.20> SIZE:57
YIL043C	[CBR1] Microsomal cytochrome b reductase, not essential for viability; also detected in mitochondria cytochrome b reductase
**YER131W**	[RPS26B] Protein component of the small (40S) ribosomal subunit; nearly identical to Rps26Ap and has similarity to rat S26 ribosomal protein ribosomal protein S26B
YLR150W	[STM1] Protein that binds G4 quadruplex and purine motif triplex nucleic acid; acts with Cdc13p to maintain telomere structure; interacts with ribosomes and subtelomeric Y' DNA; multicopy suppressor of tom1 and pop2 mutations purine motif triplex-binding protein Null mutant is viable; overexpression of STM1 suppresses some phenotypes of pop2 null mutations and the temperature sensitivity of tom1 and htr1 mutants. Cells lacking Stm1 display deficiency in the apoptosis-like cell death process induced by treatment with low concentrations of H2O2. Survival is increased when Stm1 is completely missing from the cells or when inhibition of Stm1 synthesis permits proteasomal degradation to decrease its amount in the cell. Stm1 accumulation induces cell death.
**YGL189C**	[RPS26A] Protein component of the small (40S) ribosomal subunit; nearly identical to Rps26Bp and has similarity to rat S26 ribosomal protein ribosomal protein S26A Null mutant is viable and grows slowly
YLR435W	[TSR2] Protein with a potential role in pre-rRNA processing

0265	GO_TERM:[molecular_function] P-Value:1.7e-01
YBL051C	[PIN4] Protein involved in G2/M phase progression and response to DNA damage, interacts with Rad53p; contains an RNA recognition motif, a nuclear localization signal, and several SQ/TQ cluster domains; hyperphosphorylated in response to DNA damage Other phenotypes: overexpression of PIN4 allows for the induction of the [PSI+] prion by Sup35p overproduction in the strains cured of [PIN+].
YIL151C	

0266	GO_TERM:[catalytic activity] P-Value:5.0e-02 OVERLAP:[cytoplasmic ribosomal small subunit] <500.40.20> SIZE:57
**YLR333C**	[RPS25B] Protein component of the small (40S) ribosomal subunit; nearly identical to Rps25Ap and has similarity to rat S25 ribosomal protein ribosomal protein S25B (S31B) (rp45) (YS23)
YOR360C	[PDE2] High-affinity cyclic AMP phosphodiesterase, component of the cAMP-dependent protein kinase signaling system, protects the cell from extracellular cAMP, contains readthrough motif surrounding termination codon high affinity cAMP phosphodiesterase null suppresses the heat-shock and starvation phenotypes of ras2Val119 mutation and of the ras1 ras2 double mutant; null by itself shows slow growth in presence of external cAMP, and is thermo-sensitive when nitrogen-starved in presence of cAMP
YJL101C	[GSH1] Gamma glutamylcysteine synthetase, catalyzes the first step in the gamma-glutamyl cycle for glutathione (GSH) biosynthesis; expression induced by oxidants, cadmium, and mercury gamma-glutamylcysteine synthetase Null mutant is viable, exhibits alteration of glutathione content and reduction in growth rate

YGR237C	
YML056C	[IMD4] Inosine monophosphate dehydrogenase, catalyzes the first step of GMP biosynthesis, member of a four-gene family in S. cerevisiae, constitutively expressed IMP dehydrogenase homolog
YOR243C	[PUS7] Pseudouridine synthase, catalyzes pseudouridylation at position 35 in U2 snRNA, position 13 in cytoplasmic tRNAs, and position 35 in pre-tRNA(Tyr); Asp(256) mutation abolishes activity; conserved in archaea, some bacteria, and vertebrates pseudouridine synthase

0267	GO_TERM:[regulation of translational fidelity] P-Value:2.1e-03 OVERLAP:[cytoplasmic ribosomal small subunit] <500.40.20> SIZE:57
YNL224C	
**YKR057W**	[RPS21A] Protein component of the small (40S) ribosomal subunit; nearly identical to Rps21Bp and has similarity to rat S21 ribosomal protein ribosomal protein S21A (S26A) (YS25)
YBR158W	[AMN1] Protein required for daughter cell separation, multiple mitotic checkpoints, and chromosome stability; contains 12 degenerate leucine-rich repeat motifs; expression is induced by the Mitotic Exit Network (MEN)
**YMR116C**	[ASC1] WD repeat protein (G-beta like protein) involved in translation regulation; required for repression of Gcn4p activity in the absence of amino-acid starvation; core component of the ribosome; ortholog of mammalian RACK1 G-beta like protein Null mutant is viable. Null mutation suppresses the absence of growth of a cyp1- strain in anaerobiosis and also causes delayed growth in aerobic or heme sufficient conditions; trp auxotrophs of the asc1 null allele are cold sensitive for growth; other mutants have increased cell size
YAR018C	[KIN3] Nonessential protein kinase with unknown cellular role protein kinase
YJL080C	[SCP160] Essential RNA-binding G protein effector of mating response pathway, predominantly associated with nuclear envelope and ER, interacts in mRNA-dependent manner with translating ribosomes via multiple KH domains, similar to vertebrate vigilins
YGR285C	[ZUO1] Cytosolic ribosome-associated chaperone that acts, together with Ssz1p and the Ssb proteins, as a chaperone for nascent polypeptide chains; contains a DnaJ domain and functions as a J-protein partner for Ssb1p and Ssb2p zuotin, Z-DNA binding protein (putative)
YHR064C	[SSZ1] Hsp70 protein that interacts with Zuo1p (a DnaJ homolog) to form a ribosome-associated complex that binds the ribosome via the Zuo1p subunit; also involved in pleiotropic drug resistance via sequential activation of PDR1 and PDR5; binds ATP HSP70 family Null mutant is viable, cold sensitive; SSZ1 overexpression causes increased expression of some PDR genes

0268	OVERLAP:[cytoplasmic ribosomal large subunit] <500.40.10> SIZE:81
**YDL130W**	[RPP1B] Ribosomal protein P1 beta, component of the ribosomal stalk, which is involved in interaction of translational elongation factors with ribosome; accumulation is regulated by phosphorylation and interaction with the P2 stalk component ribosomal protein P1B (L44') (YP1beta) (Ax)
YLR287C	

0269	GO_TERM:[structural constituent of ribosome] P-Value:6.5e-05 OVERLAP:[cytoplasmic ribosomal large subunit] <500.40.10> SIZE:81
YOR293W	[RPS10A] Protein component of the small (40S) ribosomal subunit; nearly identical to Rps10Bp and has similarity to rat ribosomal protein S10 ribosomal protein S10A
**YEL054C**	[RPL12A] Protein component of the large (60S) ribosomal subunit, nearly identical to Rpl12Bp; rpl12a rpl12b double mutant exhibits slow growth and slow translation; has similarity to E. coli L11 and rat L12 ribosomal proteins ribosomal protein L12A (L15A) (YL23)
**YLR075W**	[RPL10] Protein component of the large (60S) ribosomal subunit, responsible for joining the 40S and 60S subunits; regulates translation initiation; has similarity to rat L10 ribosomal protein and to members of the QM gene family ribosomal protein L10 Null mutant is inviable; temperature-sensitive mutant, at restrictive temperature (on rich medium), arrests after 1-3 cell divisions as large budded cells with aberrent septum and cytoskeleton

0270	GO_TERM:[catalytic activity] P-Value:7.3e-02
YER142C	[MAG1] 3-methyl-adenine DNA glycosylase involved in protecting DNA against alkylating agents; initiates base excision repair by removing damaged bases to create abasic sites that are subsequently repaired 3-methyladenine DNA glycosylase Null mutant is viable, deficient in 3-methyladenine DNA glycosylase activity and shows enhanced sensitivity to several monofunctional alkylating agents
YHR216W	[IMD2] Inosine monophosphate dehydrogenase, catalyzes the first step of GMP biosynthesis, expression is induced by mycophenolic acid resulting in resistance to the drug, expression is repressed by nutrient limitation IMP dehydrogenase homolog

0271	GO_TERM:[nucleic acid binding] P-Value:1.7e-02
YDR312W	[SSF2] Protein required for ribosomal large subunit maturation, functionally redundant with Ssf1p; member of the Brix family Null mutant is viable; displays double mutant lethality with ssf1 null mutations. Ssfp depletion is associated with arrest of cell division and decreased mating
YOR080W	[DIA2] Origin-binding F-box protein that forms an SCF ubiquitin ligase complex with Skp1p and Cdc53p; plays a role in DNA replication, involved in invasive and pseudohyphal growth Enhanced invasive growth in haploids; haploid budding pattern becomes polar.

0272	GO_TERM:[cytoplasm organization and biogenesis] P-Value:1.9e-66 OVERLAP:[cytoplasmic ribosomal large subunit] <500.40.10> SIZE:81
YJL122W	[ALB1] Shuttling pre-60S factor; involved in the biogenesis of ribosomal large subunit; interacts directly with Arx1p; responsible for Tif6p recycling defects in absence of Rei1p.
YPR017C	[DSS4] Nucleotide release factor functioning in the post-Golgi secretory pathway, required for ER-to-Golgi transport, binds zinc, found both on membranes and in the cytosol; guanine nucleotide dissociation stimulator GDP dissociation factor for Sec4p
YPR045C	
**YLR325C**	[RPL38] Protein component of the large (60S) ribosomal subunit, has similarity to rat L38 ribosomal protein ribosomal protein L38
YAL051W	[OAF1] Oleate-activated transcription factor, acts alone and as a heterodimer with Pip2p; activates genes involved in beta-oxidation of fatty acids and peroxisome organization and biogenesis transcription factor
YDL171C	[GLT1] NAD(+)-dependent glutamate synthase (GOGAT), synthesizes glutamate from glutamine and alpha-ketoglutarate; with Gln1p, forms the secondary pathway for glutamate biosynthesis from ammonia; expression regulated by nitrogen source glutamate synthase (NADH)
YLR233C	[EST1] TLC1 RNA-associated factor involved in telomere length regulation as the recruitment subunit of the telomerase holoenzyme, has a possible role in activating Est2p-TLC1-RNA bound to the telomere Telomere elongation protein
YBR267W	[REI1] Cytoplasmic pre-60S factor; required for the correct recycling of shuttling factors Alb1, Arx1 and Tif6 at the end of the ribosomal large subunit biogenesis; involved in bud growth in the mitotic signaling network
**YPL143W**	[RPL33A] N-terminally acetylated ribosomal protein L37 of the large (60S) ribosomal subunit, nearly identical to Rpl33Bp and has similarity to rat L35a; rpl33a null mutant exhibits slow growth while rpl33a rpl33b double null mutant is inviable ribosomal protein L33A (L37A) (YL37) (rp47) Null mutant is viable, severely impaired in growth. rpl33a rpl33b double deletion mutants are inviable
YLR455W	
YLR196W	[PWP1] Protein with WD-40 repeats involved in rRNA processing; associates with trans-acting ribosome biogenesis factors; similar to beta-transducin superfamily Null mutants are viable but show severely retarded growth
YER036C	[ARB1] ATPase of the ATP-binding cassette (ABC) family involved in 40S and 60S ribosome biogenesis, has similarity to Gcn20p; shuttles from nucleus to cytoplasm, physically interacts with Tif6p, Lsg1p Shuttling protein, ATP binding cassette protein
YDR386W	[MUS81] Helix-hairpin-helix protein, involved in DNA repair and replication fork stability; functions as an endonuclease in complex with Mms4p; interacts with Rad54p Null mutant is viable but is MMS and UV sensitive and meiosis defective, null is synthetically lethal with sgs1 null
**YDL082W**	[RPL13A] Protein component of the large (60S) ribosomal subunit, nearly identical to Rpl13Bp; not essential for viability; has similarity to rat L13 ribosomal protein ribosomal protein L13A
**YMR142C**	[RPL13B] Protein component of the large (60S) ribosomal subunit, nearly identical to Rpl13Ap; not essential for viability; has similarity to rat L13 ribosomal protein ribosomal protein L13B
**YGR085C**	[RPL11B] Protein component of the large (60S) ribosomal subunit, nearly identical to Rpl11Ap; involved in ribosomal assembly; depletion causes degradation of proteins and RNA of the 60S subunit; has similarity to E. coli L5 and rat L11 ribosomal protein L11B (L16B) (rp39B) (YL22) Null mutant is viable, rp111b rp111a mutants are inviable. rpl11 mutants are deficient in 60S ribosomal subunits relative to 40S subunits. 43S preinitiation complexes accumulate in half-mer polyribosomes in the absence of sufficient 60S subunits.
**YLR340W**	[RPP0] Conserved ribosomal protein P0 similar to rat P0, human P0, and E. coli L10e; shown to be phosphorylated on serine 302 ribosomal protein P0 (A0) (L10E)
**YBL092W**	[RPL32] Protein component of the large (60S) ribosomal subunit, has similarity to rat L32 ribosomal protein; overexpression disrupts telomeric silencing ribosomal protein L32 overexpression disrupts telomeric silencing
YBR084W	[MIS1] Mitochondrial C1-tetrahydrofolate synthase, involved in interconversion between different oxidation states of tetrahydrofolate (THF); provides activities of formyl-THF synthetase, methenyl-THF cyclohydrolase, and methylene-THF dehydrogenase C1-tetrahydrofolate synthase Null mutant is viable, exhibits no apparent defects in cell growth

YAL025C	[MAK16] Essential nuclear protein, constituent of 66S pre-ribosomal particles; required for normal concentration of free 60S ribosomal subunits; required for maintenance of M1 satellite double-stranded RNA of the L-A virus nuclear protein (putative) Null mutant is inviable, conditional mutants arrest at G(sub)1, are deficient in maintenance of killer M1 double-stranded RNA
**YLR029C**	[RPL15A] Protein component of the large (60S) ribosomal subunit, nearly identical to Rpl15Bp and has similarity to rat L15 ribosomal protein; binds to 5.8 S rRNA ribosomal protein L15A (YL10) (rp15R) (L13A)
**YPL249C-A**	[RPL36B] Protein component of the large (60S) ribosomal subunit, nearly identical to Rpl36Ap and has similarity to rat L36 ribosomal protein; binds to 5.8 S rRNA ribosomal protein L36B (L39) (YL39)
**YDL075W**	[RPL31A] Protein component of the large (60S) ribosomal subunit, nearly identical to Rpl31Bp and has similarity to rat L31 ribosomal protein; associates with the karyopherin Sxm1p ribosomal protein L31A (L34A) (YL28)
**YKL180W**	[RPL17A] Protein component of the large (60S) ribosomal subunit, nearly identical to Rpl17Bp and has similarity to E. coli L22 and rat L17 ribosomal proteins; copurifies with the components of the outer kinetochore DASH complex ribosomal protein L17A (L20A) (YL17)
YDL168W	[SFA1] Bifunctional enzyme containing both alcohol dehydrogenase and glutathione-dependent formaldehyde dehydrogenase activities, functions in formaldehyde detoxification and formation of long chain and complex alcohols, regulated by Hog1p-Sko1p glutathione-dependent formaldehyde dehydrogenase
YKL021C	[MAK11] Protein essential for cell growth and replication of M dsRNA virus; contains four beta-transducin repeats Null mutant is inviable, mak11-1 mutants result in specific loss of M1 double stranded RNA
**YPR102C**	[RPL11A] Protein component of the large (60S) ribosomal subunit, nearly identical to Rpl11Bp; involved in ribosomal assembly; depletion causes degradation of proteins and RNA of the 60S subunit; has similarity to E. coli L5 and rat L11 ribosomal protein L11A (L16A) (rp39A) (YL22)
YHR170W	[NMD3] Protein involved in nuclear export of the large ribosomal subunit; acts as a Crm1p-dependent adapter protein for export of nascent ribosomal subunits through the nuclear pore complex factor required for a late assembly step of the 60S subunit Null mutant is inviable, at nonpermissive temperature, nmd3 ts mutants exhibit decreased levels of 60S subunits resulting in formation of half-mer polysomes; nmd3 xrn1(kem1) double mutants are inviable
YPL259C	[APM1] Mu1-like medium subunit of the clathrin-associated protein complex (AP-1); binds clathrin; involved in clathrin-dependent Golgi protein sorting clathrin associated protein complex medium subunit Null mutant is viable, enhances the slow growth and late Golgi sorting defects of a chc1-ts mutant
**YGL103W**	[RPL28] Ribosomal protein L29 of the large (60S) ribosomal subunit, has similarity to E. coli L15 and rat L27a ribosomal proteins; may have peptidyl transferase activity; can mutate to cycloheximide resistance ribosomal protein L28 (L29) (rp44) (YL24)
YAL035W	[FUN12] GTPase, required for general translation initiation by promoting Met-tRNAiMet binding to ribosomes and ribosomal subunit joining; homolog of bacterial IF2 97 kDa protein
YLR276C	[DBP9] ATP-dependent RNA helicase of the DEAD-box family involved in biogenesis of the 60S ribosomal subunit
**YBL087C**	[RPL23A] Protein component of the large (60S) ribosomal subunit, identical to Rpl23Bp and has similarity to E. coli L14 and rat L23 ribosomal proteins ribosomal protein L23A (L17aA) (YL32)
**YIL133C**	[RPL16A] N-terminally acetylated protein component of the large (60S) ribosomal subunit, binds to 5.8 S rRNA; has similarity to Rpl16Bp, E. coli L13 and rat L13a ribosomal proteins; transcriptionally regulated by Rap1p ribosomal protein L16A (L21A) (rp22) (YL15)
YKR024C	[DBP7] Putative ATP-dependent RNA helicase of the DEAD-box family involved in ribosomal biogenesis RNA helicase (putative)
YER002W	[NOP16] Constituent of 66S pre-ribosomal particles, involved in 60S ribosomal subunit biogenesis ribosome biogenesis
YCL054W	[SPB1] AdoMet-dependent methyltransferase involved in rRNA processing and 60S ribosomal subunit maturation; methylates G2922 in the tRNA docking site of the large subunit rRNA and in the absence of snR52, U2921; suppressor of PAB1 mutants AdoMet-dependent rRNA methyltransferase
YNL230C	[ELA1] Elongin A, F-box protein that forms a heterodimer with Elc1p and participates in transcription elongation elongin A transcription elongation factor
YIL035C	[CKA1] Alpha catalytic subunit of casein kinase 2, a Ser/Thr protein kinase with roles in cell growth and proliferation; the holoenzyme also contains CKA2, CKB1 and CKB2, the many substrates include transcription factors and all RNA polymerases protein kinase CK2 alpha subunit Null mutant is viable; however, strains lacking both cka1 and cka2 (the alpha and alpha' subunits of protein kinase CK2, respectively) are inviable.
YPL146C	[NOP53] Nucleolar protein; involved in biogenesis of the 60S subunit of the ribosome; interacts with rRNA processing factors Cbf5p and Nop2p; null mutant is viable but growth is severely impaired
**YPL220W**	[RPL1A] N-terminally acetylated protein component of the large (60S) ribosomal subunit, nearly identical to Rpl1Bp and has similarity to E. coli L1 and rat L10a ribosomal proteins; rpl1a rpl1b double null mutation is lethal ribosomal protein L1A, forms part of the 60S ribosomal subunit Null mutant is viable; shows double mutant lethality with rpl1b (ssm2b) null mutants
YLR106C	[MDN1] Huge dynein-related AAA-type ATPase (midasin), forms extended pre-60S particle with the Rix1 complex (Rix1p-Ipi1p-Ipi3p), may mediate ATP-dependent remodeling of 60S subunits and subsequent export from nucleoplasm to cytoplasm midasin

YOR005C	[DNL4] DNA ligase required for nonhomologous end-joining (NHEJ), forms stable heterodimer with required cofactor Lif1p, catalyzes DNA ligation as part of a complex with Lif1p and Nej1p; involved in meiosis, not essential for vegetative growth ATP dependent DNA ligase Null mutant is viable, deficient in non-homologous double-strand end joining
YGL099W	[LSG1] Putative GTPase involved in 60S ribosomal subunit biogenesis; required for the release of Nmd3p from 60S subunits in the cytoplasm
YOL041C	[NOP12] Nucleolar protein, required for pre-25S rRNA processing; contains an RNA recognition motif (RRM) and has similarity to Nop13p, Nsr1p, and putative orthologs in Drosophila and S. pombe Null mutant is viable and shows slow growth and cold sensitivity
YOR294W	[RRS1] Essential protein that binds ribosomal protein L11 and is required for nuclear export of the 60S pre-ribosomal subunit during ribosome biogenesis; mouse homolog shows altered expression in Huntington's disease model mice Null mutant is inviable. Rsr1p depletion causes defects in pre-rRNA processing and ribosome assembly. The rrs1-1 mutant exhibits reduced transcriptional repression of both rRNA and ribosomal protein genes.
**YOL127W**	[RPL25] Primary rRNA-binding ribosomal protein component of the large (60S) ribosomal subunit, has similarity to E. coli L23 and rat L23a ribosomal proteins; binds to 26S rRNA via a conserved C-terminal motif ribosomal protein L25 (rpl6L) (YL25)
YNL182C	[IPI3] Protein required for cell viability; computational analysis of large-scale protein-protein interaction data suggests a possible role in assembly of the ribosomal large subunit
YHR085W	[IPI1] Protein of unknown function, essential for viability, may be involved in rRNA processing
YKL172W	[EBP2] Essential protein required for the maturation of 25S rRNA and 60S ribosomal subunit assembly, localizes to the nucleolus; constituent of 66S pre-ribosomal particles nucleolar protein
**YGL030W**	[RPL30] Protein component of the large (60S) ribosomal subunit, has similarity to rat L30 ribosomal protein; involved in pre-rRNA processing in the nucleolus; autoregulates splicing of its transcript ribosomal protein L30 (L32) (rp73) (YL38) large subunit
YCR072C	[RSA4] WD-repeat protein involved in ribosome biogenesis; required for maturation and efficient intra-nuclear transport or pre-60S ribosomal subunits, localizes to the nucleolus
**YOL120C**	[RPL18A] Protein component of the large (60S) ribosomal subunit, identical to Rpl18Bp and has similarity to rat L18 ribosomal protein; intron of RPL18A pre-mRNA forms stem-loop structures that are a target for Rnt1p cleavage leading to degradation ribosomal protein L18A (rp28A)
**YPL198W**	[RPL7B] Protein component of the large (60S) ribosomal subunit, nearly identical to Rpl7Ap and has similarity to E. coli L30 and rat L7 ribosomal proteins; contains a conserved C-terminal Nucleic acid Binding Domain (NDB2) ribosomal protein L7B (L6B) (rp11) (YL8) Null mutant is viable; disruption of both RPL7A and RPL7B is lethal
**YFR031C-A**	[RPL2A] Protein component of the large (60S) ribosomal subunit, identical to Rpl2Bp and has similarity to E. coli L2 and rat L8 ribosomal proteins ribosomal protein L2A (L5A) (rp8) (YL6)
YLL034C	[RIX7] Putative ATPase of the AAA family, required for export of pre-ribosomal large subunits from the nucleus; distributed between the nucleolus, nucleoplasm, and nuclear periphery depending on growth conditions
**YDR012W**	[RPL4B] Protein component of the large (60S) ribosomal subunit, nearly identical to Rpl4Ap and has similarity to E. coli L4 and rat L4 ribosomal proteins ribosomal protein L4B (L2B) (rp2) (YL2)
YLR449W	[FPR4] Nuclear protein, putative peptidyl-prolyl cis-trans isomerase (PPIase) with similarity to Fpr3p; overproduction suppresses the growth defect resulting from the absence of E3 ubiquitin-protein ligase Tom1p peptidyl-prolyl cis-trans isomerase (PPIase)
**YGL076C**	[RPL7A] Protein component of the large (60S) ribosomal subunit, nearly identical to Rpl7Bp and has similarity to E. coli L30 and rat L7 ribosomal proteins; contains a conserved C-terminal Nucleic acid Binding Domain (NDB2) ribosomal protein L7A (L6A) (rp11) (YL8) Null mutant is viable; grows more slowly than wild-type
**YMR242C**	[RPL20A] Protein component of the large (60S) ribosomal subunit, nearly identical to Rpl20Bp and has similarity to rat L18a ribosomal protein ribosomal protein L20A (L18A)
YLR002C	[NOC3] Protein that forms a nuclear complex with Noc2p that binds to 66S ribosomal precursors to mediate their intranuclear transport; also binds to chromatin to promote the association of DNA replication factors and replication initiation
YNR053C	[NOG2] Putative GTPase that associates with pre-60S ribosomal subunits in the nucleolus and is required for their nuclear export and maturation part of a pre-60S complex
YPR143W	[RRP15] Nucleolar protein, constituent of pre-60S ribosomal particles; required for processing of the 27S pre-rRNA at the A2 site to yield 5.8S and 25S rRNA
YGR245C	[SDA1] Highly conserved nuclear protein required for actin cytoskeleton organization and passage through Start, plays a critical role in G1 events, binds Nap1p, also involved in 60S ribosome biogenesis
YLR074C	[BUD20] Protein involved in bud-site selection; diploid mutants display a random budding pattern instead of the wild-type bipolar pattern
YHR197W	[RIX1] Essential protein involved in the processing of the ITS2 region of the rRNA locus; required for the maturation and nuclear export of the 60S ribosomal subunit
YKL014C	[URB1] Nucleolar protein required for the normal accumulation of 25S and 5.8S rRNAs, associated with the 27SA2 pre-ribosomal particle; proposed to be involved in the biogenesis of the 60S ribosomal subunit
YKL009W	[MRT4] Protein involved in mRNA turnover and ribosome assembly, localizes to the nucleolus Null mutant exhibits slow growth. ts mutation results in decreased decay rates of mRNAs
**YHR010W**	[RPL27A] Protein component of the large (60S) ribosomal subunit, nearly identical to Rpl27Bp and has similarity to rat L27 ribosomal protein ribosomal protein L27A

YDR101C	[ARX1] Shuttling pre-60S factor; involved in the biogenesis of ribosomal large subunit biogenesis; interacts directly with Alb1; responsible for Tif6 recycling defects in absence of Rei1; associated with the ribosomal export complex
**YLL045C**	[RPL8B] Ribosomal protein L4 of the large (60S) ribosomal subunit, nearly identical to Rpl8Ap and has similarity to rat L7a ribosomal protein; mutation results in decreased amounts of free 60S subunits ribosomal protein L8B (L4B) (rp6) (YL5) Null mutant is viable. rpl8a rpl8b mutants are inviable. The rpl8b allele, krb1, can suppress some mak mutations and represents a chromosomal rearrangement involving chromosome XII
YBR142W	[MAK5] Essential nucleolar protein, putative DEAD-box RNA helicase required for maintenance of M1 dsRNA virus; involved in biogenesis of large (60S) ribosomal subunits deficient in maintenance of killer
YER126C	[NSA2] Constituent of 66S pre-ribosomal particles, involved in 60S ribosomal subunit biogenesis ribosome biogenesis Heterozygous diploid mutant exhibit haploinsufficiency K1 killer toxin resistance
**YOR063W**	[RPL3] Protein component of the large (60S) ribosomal subunit, has similarity to E. coli L3 and rat L3 ribosomal proteins; involved in the replication and maintenance of killer double stranded RNA virus ribosomal protein L3 (rp1) (YL1) Tricodermin resistance
YNL175C	[NOP13] Protein of unknown function, localizes to the nucleolus and nucleoplasm; contains an RNA recognition motif (RRM) and has similarity to Nop12p, which is required for processing of pre-18S rRNA
YLL008W	[DRS1] Nucleolar DEAD-box protein required for ribosome assembly and function, including synthesis of 60S ribosomal subunits; constituent of 66S pre-ribosomal particles ATP dependent RNA helicase (putative)
YDR060W	[MAK21] Constituent of 66S pre-ribosomal particles, required for large (60S) ribosomal subunit biogenesis; involved in nuclear export of pre-ribosomes; required for maintenance of dsRNA virus; homolog of human CAATT-binding protein deficient in maintenance of killer
YDL031W	[DBP10] Putative ATP-dependent RNA helicase of the DEAD-box protein family, constituent of 66S pre-ribosomal particles; essential protein involved in ribosome biogenesis
YPL211W	[NIP7] Nucleolar protein required for 60S ribosome subunit biogenesis, constituent of 66S pre-ribosomal particles; physically interacts with Nop8p and the exosome subunit Rrp43p Null mutant is inviable; in the temperature-sensitive mutant nip7-1, glycine 71 is replaced by aspartic acid
**YPL131W**	[RPL5] Protein component of the large (60S) ribosomal subunit with similarity to E. coli L18 and rat L5 ribosomal proteins; binds 5S rRNA and is required for 60S subunit assembly ribosomal protein L5 (L1a)(YL3)
YPL043W	[NOP4] Nucleolar protein, essential for processing and maturation of 27S pre-rRNA and large ribosomal subunit biogenesis; constituent of 66S pre-ribosomal particles; contains four RNA recognition motifs (RRMs) RNA binding protein (putative) Null mutant is inviable; conditional mutant shows diminished accumulation of 60S ribosomal subunits due to a lack of production of mature 25S rRNA from 27S precursor rRNA
YHR088W	[RPF1] Nucleolar protein involved in the assembly of the large ribosomal subunit; constituent of 66S pre-ribosomal particles; contains a sigma(70)-like motif, which is thought to bind RNA
YDR496C	[PUF6] Pumilio-homology domain protein that binds ASH1 mRNA at PUF consensus sequences in the 3' UTR and represses its translation, resulting in proper asymmetric localization of ASH1 mRNA Deletion reduces the asymmetric localization of both Ash1p and ASH1 mRNA and affects the HO promoter activity.
YFL002C	[SPB4] Putative ATP-dependent RNA helicase, nucleolar protein required for synthesis of 60S ribosomal subunits at a late step in the pathway; sediments with 66S pre-ribosomes in sucrose gradients ATP dependent RNA helicase suppression of pab1 null mutant
YFR001W	[LOC1] Nuclear protein involved in asymmetric localization of ASH1 mRNA; binds double-stranded RNA in vitro; constituent of 66S pre-ribosomal particles Mutant exhibits slow growth at 30C
YNL002C	[RLP7] Nucleolar protein with similarity to large ribosomal subunit L7 proteins; constituent of 66S pre-ribosomal particles; plays an essential role in processing of precursors to the large ribosomal subunit RNAs Null mutant is inviable<br>required for an early step in large ribosomal subunit biogenesis
YNL110C	[NOP15] Constituent of 66S pre-ribosomal particles, involved in 60S ribosomal subunit biogenesis; localizes to both nucleolus and cytoplasm ribosome biogenesis
YER006W	[NUG1] GTPase that associates with nuclear 60S pre-ribosomes, required for export of 60S ribosomal subunits from the nucleus Nuclear GTPase involved in Ribosome biogenesis
YMR290C	[HAS1] ATP-dependent RNA helicase; localizes to both the nuclear periphery and nucleolus; highly enriched in nuclear pore complex fractions; constituent of 66S pre-ribosomal particles RNA-dependent helicase (putative)
YGL111W	[NSA1] Constituent of 66S pre-ribosomal particles, involved in 60S ribosomal subunit biogenesis ribosome biogenesis
YLR009W	[RLP24] Ribosomal Like Protein 24 part of a pre-60S complex
YHR066W	[SSF1] Constituent of 66S pre-ribosomal particles, required for ribosomal large subunit maturation; functionally redundant with Ssf2p; member of the Brix family Ssf2p homolog Null mutant is viable, ssf1 ssf2 double deletion mutants are inviable. SSF1 is a high copy suppressor of the mating defect caused by a temperature sensitive G beta subunit mutation. Depletion of SSF gene products from growing cultures caused both an arrest of cell division and a significant decrease in the ability of cells to mate. Mating efficiency was increased by extra copies of the SSF genes and decreased by elimination of the gene products
YPL093W	[NOG1] Putative GTPase that associates with free 60S ribosomal subunits in the nucleolus and is required for 60S ribosomal subunit biogenesis; constituent of 66S pre-ribosomal particles; member of the ODN family of nucleolar G-proteins homologs identified in human and Trypanosoma brucei|nucleolar G-protein (putative)

YDR087C	[RRP1] Essential evolutionarily conserved nucleolar protein necessary for biogenesis of 60S ribosomal subunits and processing of pre-rRNAs to mature rRNAs, associated with several distinct 66S pre-ribosomal particles Null mutant is inviable, cannot be suppressed by srd1 mutations. rrp1-1 mutations are associated with temperature-sensitive growth, a conditional defect in processing of 27S pre-rRNA to mature 25S rRNA, and a nonconditional increase in sensitivity to several aminoglycoside antibiotics. srd1 is an allele-specific suppressor of rrp1-1.
YOL077C	[BRX1] Nucleolar protein, constituent of 66S pre-ribosomal particles; depletion leads to defects in rRNA processing and a block in the assembly of large ribosomal subunits; possesses a sigma(70)-like RNA-binding motif
YOR206W	[NOC2] Protein that forms a nucleolar complex with Mak21p that binds to 90S and 66S pre-ribosomes, as well as a nuclear complex with Noc3p that binds to 66S pre-ribosomes; both complexes mediate intranuclear transport of ribosomal precursors
YMR049C	[ERB1] Protein required for maturation of the 25S and 5.8S ribosomal RNAs; constituent of 66S pre-ribosomal particles; homologous to mammalian Bop1
YKR081C	[RPF2] Essential protein involved in the processing of pre-rRNA and the assembly of the 60S ribosomal subunit; interacts with ribosomal protein L11; localizes predominantly to the nucleolus; constituent of 66S pre-ribosomal particles
YNL061W	[NOP2] Probable RNA m(5)C methyltransferase, essential for processing and maturation of 27S pre-rRNA and large ribosomal subunit biogenesis; localized to the nucleolus; constituent of 66S pre-ribosomal particles 90 kDa protein homologous to a human proliferation-associated nucleolar protein, p120 Null mutant is inviable; overexpression leads to changes in nucleolar morphology
YOR272W	[YTM1] Constituent of 66S pre-ribosomal particles, required for maturation of the large ribosomal subunit microtubule-associated protein
YPR016C	[TIF6] Constituent of 66S pre-ribosomal particles, has similarity to human translation initiation factor 6 (eIF6); may be involved in the biogenesis and or stability of 60S ribosomal subunits Null mutant is inviable; cells are depleted of 60S ribosomal subunits, translation initiation is inhibited, and cells arrest in G1
YGR103W	[NOP7] Nucleolar protein involved in rRNA processing and 60S ribosomal subunit biogenesis; constituent of several different pre-ribosomal particles
YHR052W	[CIC1] Essential protein that interacts with proteasome components and has a potential role in proteasome substrate specificity; also copurifies with 66S pre-ribosomal particles Null: lethal. Other phenotypes: cic1-2 ts mutant stabilizes F-box proteins.

0273	GO_TERM:[eukaryotic translation initiation factor 4F complex] P-Value:1.5e-07 OVERLAP:[eIF4F] <500.10.80> SIZE:3
YLR363W-A	
YML017W	[PSP2] Asn rich cytoplasmic protein that contains RGG motifs; high-copy suppressor of group II intron-splicing defects of a mutation in MRS2 and of a conditional mutation in POL1 (DNA polymerase alpha); possible role in mitochondrial mRNA splicing Null mutant is viable, exhibits no apparent defects; psp1 psp2 double deletion mutants are viable
YIR001C	[SGN1] Cytoplasmic RNA-binding protein, contains an RNA recognition motif (RRM); may have a role in mRNA translation, as suggested by genetic interactions with genes encoding proteins involved in translational initiation
YLR432W	[IMD3] Inosine monophosphate dehydrogenase, catalyzes the first step of GMP biosynthesis, member of a four-gene family in S. cerevisiae, constitutively expressed IMP dehydrogenase homolog
YBL032W	[HEK2] RNA binding protein with similarity to hnRNP-K that localizes to the cytoplasm and to subtelomeric DNA; required for the proper localization of ASH1 mRNA; involved in the regulation of telomere position effect and telomere length Null: ASH1 mRNA is partially delocalized
YOL139C	[CDC33] Cytoplasmic mRNA cap binding protein; the eIF4E-cap complex is responsible for mediating cap-dependent mRNA translation via interactions with the translation initiation factor eIF4G (Tif4631p or Tif4632p) mRNA cap binding protein eIF-4E Null mutant is inviable. cdc33 mutants arrest at G(sub)1. cdc33 has normal cAMP pools and is not suppressed by cAPK mutants, suggesting sporulation is independent of the cAMP pathway
**YGL049C**	[TIF4632] Translation initiation factor eIF4G, subunit of the mRNA cap-binding protein complex (eIF4F) that also contains eIF4E (Cdc33p); associates with the poly(A)-binding protein Pab1p, also interacts with eIF4A (Tif1p); homologous to Tif4631p 150 kDa|eIF-4F mRNA cap-binding complex subunit|eIF-4G homolog Null mutant is viable; tif4631 tif4632 double disruption mutants are inviable
**YGR162W**	[TIF4631] Translation initiation factor eIF4G, subunit of the mRNA cap-binding protein complex (eIF4F) that also contains eIF4E (Cdc33p); associates with the poly(A)-binding protein Pab1p, also interacts with eIF4A (Tif1p); homologous to Tif4632p 150 kDa subunit|Tif4632p and mammalian p220 homolog|mRNA cap binding protein eIF-4F Null mutant is viable, grows slowly and is cold-sensitive. tif4631 tif4632 double deletion mutants are inviable

0274	GO_TERM:[rRNA processing] P-Value:9.5e-04 OVERLAP:[rRNA splicing] <440.30.20> SIZE:24
YEL026W	[SNU13] RNA binding protein, part of U3 snoRNP involved in rRNA processing, part of U4/U6-U5 tri-snRNP involved in mRNA splicing, similar to human 15.5K protein U3 snoRNP protein|U4/U6.U5 snRNP component
YJL010C	[YJL010C] Essential nucleolar protein required for 18S rRNA synthesis

**YGR159C**	[NSR1] Nucleolar protein that binds nuclear localization sequences, required for pre-rRNA processing and ribosome biogenesis nuclear localization sequence binding protein Null mutant is viable, shows severe growth defect.
YLR410W	[VIP1] Protein of unknown function probably involved in the function of the cortical actin cytoskeleton; putative ortholog of S. pombe asp1+

0275	GO_TERM:[nucleolus] P-Value:3.2e-02
YCR087C-A	
YKR092C	[SRP40] Nucleolar, serine-rich protein with a role in preribosome assembly or transport; may function as a chaperone of small nucleolar ribonucleoprotein particles (snoRNPs); immunologically and structurally to rat Nopp140 Nopp140 homolog, a nonribosomal protein of the nucleolus and coiled bodies|nucleolar protein

0276	GO_TERM:[nucleolar preribosome, small subunit precursor] P-Value:3.0e-05 OVERLAP:[cytoplasmic ribosomal small subunit] <500.40.20> SIZE:57
**YNL178W**	[RPS3] Protein component of the small (40S) ribosomal subunit, has apurinic/apyrimidinic (AP) endonuclease activity; essential for viability; has similarity to E. coli S3 and rat S3 ribosomal proteins ribosomal protein S3 (rp13) (YS3)
YKL143W	[LTV1] Protein required for growth at low temperature
YPL239W	[YAR1] Cytoplasmic ankyrin-repeat containing protein of unknown function, proposed to link the processes of 40S ribosomal subunit biogenesis and adaptation to osmotic and oxidative stress; expression repressed by heat shock 200-amino-acid protein with two ANK repeat motifs and an acidic C terminus rich in PEST-like sequences Null mutant is viable, grow slowly at low temperature. YAR1 overexpression has no phenotype

0277	GO_TERM:[unannotated] P-Value:1.6e-02
YGR283C	
YMR310C	

0278	GO_TERM:[35S primary transcript processing] P-Value:3.4e-07 OVERLAP:[rRNA splicing] <440.30.20> SIZE:24
YGL078C	[DBP3] Putative ATP-dependent RNA helicase of the DEAD-box family involved in ribosomal biogenesis ATP dependent RNA helicase|dead/deah box protein CA3
YDL208W	[NHP2] Nuclear protein related to mammalian high mobility group (HMG) proteins, essential for function of H/ACA-type snoRNPs, which are involved in 18S rRNA processing HMG-like protein
YHR072W-A	[NOP10] Constituent of small nucleolar ribonucleoprotein particles containing H/ACA-type snoRNAs, which are required for pseudouridylation and processing of pre-18S rRNA H/ACA-box snoRNPs component
**YHR089C**	[GAR1] Protein component of the H/ACA snoRNP pseudouridylase complex, involved in the modification and cleavage of the 18S pre-rRNA small nucleolar RNP protein

0279	GO_TERM:[regulation of translational fidelity] P-Value:5.7e-05 OVERLAP:[cytoplasmic ribosomal small subunit] <500.40.20> SIZE:57
**YBR189W**	[RPS9B] Protein component of the small (40S) ribosomal subunit; nearly identical to Rps9Bp and has similarity to E. coli S4 and rat S9 ribosomal proteins ribosomal protein S9B (S13) (rp21) (YS11)
**YPL081W**	[RPS9A] Protein component of the small (40S) ribosomal subunit; nearly identical to Rps9Ap and has similarity to E. coli S4 and rat S9 ribosomal proteins ribosomal protein S9A (S13) (rp21) (YS11)

0280	GO_TERM:[non-membrane-bound organelle] P-Value:1.8e-01 OVERLAP:[cytoplasmic ribosomal small subunit] <500.40.20> SIZE:57
**YML063W**	[RPS1B] Ribosomal protein 10 (rp10) of the small (40S) subunit; nearly identical to Rps1Ap and has similarity to rat S3a ribosomal protein ribosomal protein S1B (rp10B)
YPR112C	[MRD1] Essential conserved protein that associates with 35S precursor rRNA and is required for its initial processing at the A(0)-A(2) cleavage sites, shows partial nucleolar localization, contains five consensus RNA-binding domains Null mutant is inviable (haploid divides 2-3 times)

0281	GO_TERM:[ribosome biogenesis] P-Value:4.2e-07 OVERLAP:[Casein kinase I] <120.10> SIZE:4
YOR145C	[PNO1] Essential nucleolar protein required for pre-18S rRNA processing, interacts with Dim1p, an 18S rRNA dimethyltransferase, and also with Nob1p, which is involved in proteasome biogenesis; contains a KH domain Associated with Nob1 Essential for growth. Other phenotypes: temperature sensitive phenotype of pno1-1.
YPL266W	[DIM1] Essential 18S rRNA dimethylase, responsible for conserved m6(2)Am6(2)A dimethylation in 3'-terminal loop of 18 S rRNA, part of 90S and 40S pre-particles in nucleolus, involved in pre-ribosomal RNA processing dimethyladenosine transferase
**YPL204W**	[HRR25] Protein kinase involved in regulating diverse events including vesicular trafficking, gene expression, DNA repair, and chromosome segregation; binds the CTD of RNA pol II; homolog of mammalian casein kinase 1delta (CK1delta) casein kinase I isoform Null mutant is viable but shows slow growth; hrr25-1 mutation results in sensitivity to continuous expression of HO endonuclease, to methylmethanesulfonate, and to x-irradiation; homozygous hrr25-1 mutants are unable to sporulate
YNL207W	[RIO2] Essential serine kinase involved in the processing of the 20S pre-rRNA into mature 18S rRNA; has similarity to Rio1p
YOR056C	[NOB1] Essential nuclear protein involved in proteasome maturation and synthesis of 40S ribosomal subunits; required for cleavage of the 20S pre-rRNA to generate the mature 18S rRNA Associated with the 26S proteasome

0282	GO_TERM:[rRNA processing] P-Value:2.3e-63 OVERLAP:[rRNA splicing] <440.30.20> SIZE:24
**YHL034C**	[SBP1] Nucleolar single-strand nucleic acid binding protein; associates with small nuclear RNAs
YGR081C	[SLX9] Protein of unknown function; deletion mutant has synthetic fitness defect with an sgs1 deletion mutant
YJR123W	[RPS5] Protein component of the small (40S) ribosomal subunit, the least basic of the non-acidic ribosomal proteins; phosphorylated in vivo; essential for viability; has similarity to E. coli S7 and rat S5 ribosomal proteins ribosomal protein S5 (S2) (rp14) (YS8) null is inviable; transcription of RPS5 is sensitive to heat-shock and carbon source shift.
YGL171W	[ROK1] ATP-dependent RNA helicase of the DEAD box family; required for 18S rRNA synthesis Null mutant is inviable. Depletion of Rok1p inhibits pre-rRNA processing at sites A0, A1, and A2, thereby blocking 18S rRNA synthesis.
YIL069C	[RPS24B] Protein component of the small (40S) ribosomal subunit; identical to Rps24Ap and has similarity to rat S24 ribosomal protein ribosomal protein S24B
YER102W	[RPS8B] Protein component of the small (40S) ribosomal subunit; identical to Rps8Bp and has similarity to rat S8 ribosomal protein ribosomal protein S8B (S14B) (rp19) (YS9)
YBR031W	[RPL4A] N-terminally acetylated protein component of the large (60S) ribosomal subunit, nearly identical to Rpl4Bp and has similarity to E. coli L4 and rat L4 ribosomal proteins ribosomal protein L4A (L2A) (rp2) (YL2)
YKR060W	[UTP30] Possible U3 snoRNP protein involved in maturation of pre-18S rRNA, based on computational analysis of large-scale protein-protein interaction data
YJL033W	[HCA4] Putative nucleolar DEAD box RNA helicase; high-copy number suppression of a U14 snoRNA processing mutant suggests an involvement in 18S rRNA synthesis RNA helicase (putative)
YML024W	[RPS17A] Ribosomal protein 51 (rp51) of the small (40s) subunit; nearly identical to Rps17Bp and has similarity to rat S17 ribosomal protein ribosomal protein S17A (rp51A) Null mutant is viable and grows slowly; rps17A rps17B double null mutant is inviable
YDR025W	[RPS11A] Protein component of the small (40S) ribosomal subunit; identical to Rps11Bp and has similarity to E. coli S17 and rat S11 ribosomal proteins ribosomal protein S11A (S18A) (rp41A) (YS12)
YEL055C	[POL5] Protein with sequence similarity to the human MybBP1A and weak sequence similar to B-type DNA polymerases, not required for chromosomal DNA replication; required for the synthesis of rRNA DNA polymerase phi
YDR299W	[BFR2] Essential protein possibly involved in secretion; multicopy suppressor of sensitivity to Brefeldin A Null mutant is inviable; BFR2 overexpression can suppress the growth defect of mutants blocked at the step of budding or docking of small vessicles en route to the Golgi
YDL060W	[TSR1] Protein required for processing of 20S pre-rRNA in the cytoplasm, associates with pre-40S ribosomal particles essential
YGR145W	[ENP2] Essential nucleolar protein of unknown function; contains WD repeats, interacts with Mpp10p and Bfr2p, and has homology to Spb1p
YLR441C	[RPS1A] Ribosomal protein 10 (rp10) of the small (40S) subunit; nearly identical to Rps1Bp and has similarity to rat S3a ribosomal protein ribosomal protein S1A (rp10A)
YDR447C	[RPS17B] Ribosomal protein 51 (rp51) of the small (40s) subunit; nearly identical to Rps17Ap and has similarity to rat S17 ribosomal protein ribosomal protein S17B (rp51B) Null mutant is viable, rp51a (rps17a) rp51b (rps17b) deletion mutants are inviable
YDR365C	[ESF1] Nucleolar protein involved in pre-rRNA processing; depletion causes severely decreased 18S rRNA levels
**YDR398W**	[UTP5] Nucleolar protein, component of the small subunit (SSU) processome containing the U3 snoRNA that is involved in processing of pre-18S rRNA U3 snoRNP protein
YLR175W	[CBF5] Component of box H/ACA small nucleolar ribonucleoprotein particles (snoRNPs), probable rRNA pseudouridine synthase, binds to snoRNP Nop10p and also interacts with ribosomal biogenesis protein Nop53p major low affinity 55 kDa centromere/microtubule binding protein
YPL012W	[RRP12] Protein required for export of the ribosomal subunits; associates with the RNA components of the pre-ribosomes; contains HEAT-repeats

YNL075W	[IMP4] Component of the SSU processome, which is required for pre-18S rRNA processing; interacts with Mpp10p; member of a superfamily of proteins that contain a sigma(70)-like motif and associate with RNAs U3 snoRNP protein
YLR197W	[SIK1] Essential evolutionarily-conserved nucleolar protein component of the box C/D snoRNP complexes that direct 2'-O-methylation of pre-rRNA during its maturation; overexpression causes spindle orientation defects U3 snoRNP protein wild-type gene suppresses toxicity of GAL4-I-Kappa-B alpha in yeast|Other phenotypes: Shortens the G1 phase of the cell cycle when present in high-copy
YNL308C	[KRI1] Essential nucleolar protein required for 40S ribosome biogenesis; physically and functionally interacts with Krr1p Krr1p binding protein
YDR064W	[RPS13] Protein component of the small (40S) ribosomal subunit; has similarity to E. coli S15 and rat S13 ribosomal proteins ribosomal protein S13 (S27a) (YS15)
YDL213C	[NOP6] Protein with similarity to hydrophilins, which are involved in the adaptive response to hyperosmotic conditions; computational analysis of large-scale protein-protein interaction data suggests a possible role in rRNA processing
YBL072C	[RPS8A] Protein component of the small (40S) ribosomal subunit; identical to Rps8Ap and has similarity to rat S8 ribosomal protein ribosomal protein S8A (S14A) (rp19) (YS9)
YJR145C	[RPS4A] Protein component of the small (40S) ribosomal subunit; mutation affects 20S pre-rRNA processing; identical to Rps4Bp and has similarity to rat S4 ribosomal protein ribosomal protein S4A (YS6) (rp5) (S7A) Null mutant is viable; rps4a rps4b double deletion is inviable
YHR203C	[RPS4B] Protein component of the small (40S) ribosomal subunit; identical to Rps4Bp and has similarity to rat S4 ribosomal protein ribosomal protein S4B (YS6) (rp5) (S7B)
YOR096W	[RPS7A] Protein component of the small (40S) ribosomal subunit, nearly identical to Rps7Bp; interacts with Kti11p; deletion causes hypersensitivity to zymocin; has similarity to rat S7 and Xenopus S8 ribosomal proteins ribosomal protein S7A (rp30)
YOL010W	[RCL1] RNA terminal phosphate cyclase-like protein involved in rRNA processing at sites A0, A1, and A2; does not possess detectable RNA cyclase activity
YPL217C	[BMS1] Essential conserved nucleolar GTP-binding protein required for synthesis of 40S ribosomal subunits and for processing of the 35S pre-rRNA at sites A0, A1, and A2; interacts with Rcl1p, has similarity to Tsr1p Null mutant is inviable; a temperature-sensitive allele exhibits a synthetic growth defect with bmh1-delta; the temperature-sensitive allele also exhibits diploid specific bud site randomization at the semi-permissive temperature
YMR128W	[ECM16] Essential DEAH-box ATP-dependent RNA helicase specific to the U3 snoRNP, predominantly nucleolar in distribution, required for 18S rRNA synthesis U3 snoRNP protein A Tn3 insertion into this gene causes hypersensitivity to the cell surface polymer perturbing agent calcofluor white.
YNR054C	[ESF2] Essential nucleolar protein involved in pre-18S rRNA processing; component of the small subunit (SSU) processome; has sequence similarity to mABT1, a mouse transcription activator
YNL132W	[KRE33] Essential protein of unknown function; heterozygous mutant shows haploinsufficiency in K1 killer toxin resistance Heterozygous diploid mutant exhibit haploinsufficiency K1 killer toxin resistance
YLL011W	[SOF1] Essential subunit of the U3 (box C+D) snRNP complex required for 2' O-methylation of pre-RNA; has similarity to the beta subunit of trimeric G-proteins and the splicing factor Prp4p U3 snoRNP protein Null mutant is inviable. sof1-56, a dominant suppressor of nop1 mutants can restore growth and pre-RNA processing at 35 degrees C. In vivo depletion of SOF1 leads to impaired pre-rRNA processing and inhibition of 18S rRNA production.
**YDL014W**	[NOP1] Nucleolar protein, component of the small subunit processome complex, which is required for processing of pre-18S rRNA; has similarity to mammalian fibrillarin U3 snoRNP protein|similar to mammalian fibrillarin Null mutant is inviable. Temperature-sensitive alleles exhibit various defects in rRNA processing.|
YLR186W	[EMG1] Protein required for the maturation of the 18S rRNA and for 40S ribosome production; associated with spindle/microtubules; nuclear localization depends on physical interaction with Nop14p; may bind snoRNAs ribosome biogenesis
YLR129W	[DIP2] Nucleolar protein, specifically associated with the U3 snoRNA, part of the large ribonucleoprotein complex known as the small subunit (SSU) processome, required for 18S rRNA biogenesis, part of the active pre-rRNA processing complex U3 snoRNP protein
YLR409C	[UTP21] Possible U3 snoRNP protein involved in maturation of pre-18S rRNA, based on computational analysis of large-scale protein-protein interaction data U3 snoRNP protein
**YLR222C**	[UTP13] Nucleolar protein, component of the small subunit (SSU) processome containing the U3 snoRNA that is involved in processing of pre-18S rRNA U3 snoRNP protein
YHR148W	[IMP3] Component of the SSU processome, which is required for pre-18S rRNA processing, essential protein that interacts with Mpp10p and mediates interactions of Imp4p and Mpp10p with U3 snoRNA U3 snoRNP protein Null mutant is inviable. Depletion of Imp3p prevents the synthesis of mature 18S rRNA.
YJL069C	[UTP18] Possible U3 snoRNP protein involved in maturation of pre-18S rRNA, based on computational analysis of large-scale protein-protein interaction data U3 snoRNA associated protein|U3 snoRNP protein Null: lethal. Other phenotypes: required for 18S RNA production
YMR229C	[RRP5] Protein required for the synthesis of both 18S and 5.8S rRNA; C-terminal region is crucial for the formation of 18S rRNA and N-terminal region is required for the 5.8S rRNA; component of small ribosomal subunit (SSU) processosome U3 snoRNP protein Overexpression of RRP5 facilitates mitochondrial import of hydrophobic proteins; overexpression of an RRP5 mutant complements the rRNA processing defect of the null alllele, but does not facilitate mitochondrial import; required for processing of pre-rRNA
YBL004W	[UTP20] Component of the small-subunit (SSU) processome, which is involved in the biogenesis of the 18S rRNA U3 snoRNP protein

YBR247C	[ENP1] Protein associated with U3 and U14 snoRNAs, required for pre-rRNA processing and 40S ribosomal subunit synthesis; localized in the nucleus and concentrated in the nucleolus 57 kDa protein with an apparent MW of 70 kDa by SDS-PAGE (putative)
YDL148C	[NOP14] Nucleolar protein, forms a complex with Noc4p that mediates maturation and nuclear export of 40S ribosomal subunits; also present in the small subunit processome complex, which is required for processing of pre-18S rRNA U3 snoRNP protein
YOR310C	[NOP58] Protein involved in pre-rRNA processing, 18S rRNA synthesis, and snoRNA synthesis; component of the small subunit processome complex, which is required for processing of pre-18S rRNA U3 snoRNP protein Null mutant is inviable; in vivo depletion impairs synthesis of the 40S ribosomal subunit
**YGR128C**	[UTP8] Nucleolar protein required for export of tRNAs from the nucleus; also copurifies with the small subunit (SSU) processome containing the U3 snoRNA that is involved in processing of pre-18S rRNA U3 snoRNP protein
YGR090W	[UTP22] Possible U3 snoRNP protein involved in maturation of pre-18S rRNA, based on computational analysis of large-scale protein-protein interaction data
YPR144C	[NOC4] Nucleolar protein, forms a complex with Nop14p that mediates maturation and nuclear export of 40S ribosomal subunits U3 snoRNP protein Null: lethal. Other phenotypes: required for 18S RNA production
YCL059C	[KRR1] Essential nucleolar protein required for the synthesis of 18S rRNA and for the assembly of 40S ribosomal subunit
YOR078W	[BUD21] Component of small ribosomal subunit (SSU) processosome that contains U3 snoRNA; originally isolated as bud-site selection mutant that displays a random budding pattern U3 snoRNP protein Null mutant is viable; random budding in diploid null mutants; null has both reduced growth and reduced protein synthesis rates
**YER082C**	[UTP7] Nucleolar protein, component of the small subunit (SSU) processome containing the U3 snoRNA that is involved in processing of pre-18S rRNA U3 snoRNP protein Heterozygous diploid mutant exhibit haploinsufficiency K1 killer toxin resistance
YMR093W	[UTP15] Nucleolar protein, component of the small subunit (SSU) processome containing the U3 snoRNA that is involved in processing of pre-18S rRNA U3 snoRNP protein
**YDR449C**	[UTP6] Nucleolar protein, component of the small subunit (SSU) processome containing the U3 snoRNA that is involved in processing of pre-18S rRNA U3 snoRNP protein
**YDR324C**	[UTP4] Nucleolar protein, component of the small subunit (SSU) processome containing the U3 snoRNA that is involved in processing of pre-18S rRNA U3 snoRNP protein
YPL126W	[NAN1] U3 snoRNP protein, component of the small (ribosomal) subunit (SSU) processosome containing U3 snoRNA; required for the biogenesis of18S rRNA U3 snoRNP protein
YJR002W	[MPP10] Component of the SSU processome, which is required for pre-18S rRNA processing, interacts with and controls the stability of Imp3p and Imp4p, essential for viability; similar to human Mpp10p U3 snoRNP protein
**YJL109C**	[UTP10] Nucleolar protein, component of the small subunit (SSU) processome containing the U3 snoRNA that is involved in processing of pre-18S rRNA U3 snoRNP protein
YPR137W	[RRP9] Protein involved in pre-rRNA processing, associated with U3 snRNP; component of small ribosomal subunit (SSU) processosome; ortholog of the human U3-55k protein U3 snoRNP protein null mutant is inviable; genetic depletion inhibits pre-rRNA processing at sites A0, A1 and A2, and thereby inhibits synthesis of 18S rRNA
YIL019W	[FAF1] Protein required for pre-rRNA processing and 40S ribosomal subunit assembly
YCR057C	[PWP2] Conserved 90S pre-ribosomal component essential for proper endonucleolytic cleavage of the 35 S rRNA precursor at A0, A1, and A2 sites; contains eight WD-repeats; PWP2 deletion leads to defects in cell cycle and bud morphogenesis U3 snoRNP protein
**YHR196W**	[UTP9] Nucleolar protein, component of the small subunit (SSU) processome containing the U3 snoRNA that is involved in processing of pre-18S rRNA U3 snoRNP protein

0283	GO_TERM:[NAD-independent histone deacetylase activity] P-Value:1.2e-17
YHR149C	[SKG6] Protein of unknown function; found in the bud tip and bud neck, potential Cdc28p substrate; Skg6p interacts with Zds1p and Zds2p
YML109W	[ZDS2] Protein that interacts with silencing proteins at the telomere, involved in transcriptional silencing; paralog of Zds1p Null mutant is viable; zds1 zds2 double deletion causes slow growth and defects in bud morphology and cell cycle progression
YMR273C	[ZDS1] Protein that interacts with silencing proteins at the telomere, involved in transcriptional silencing; has a role in localization of Bcy1p, a regulatory subunit of protein kinase A; implicated in mRNA nuclear export High-copy suppressor of ceg-ts mutations
YBR103W	[SIF2] WD40 repeat-containing subunit of the Set3C histone deacetylase complex, which represses early/middle sporulation genes; antagonizes telomeric silencing; binds specifically to the Sir4p N-terminus Null mutant is viable, exhibits increased telomeric silencing
YOL068C	[HST1] NAD(+)-dependent histone deacetylase; essential subunit of the Sum1p/Rfm1p/Hst1p complex required for ORC-dependent silencing and mitotic repression; non-essential subunit of the Set3C deacetylase complex; involved in telomere maintenance Overexpression restores transcriptional silencing in a sir2 mutant
YIL112W	[HOS4] Subunit of the Set3 complex, which is a meiotic-specific repressor of sporulation specific genes that contains deacetylase activity; potential Cdc28p substrate
YDR155C	[CPR1] Cytoplasmic peptidyl-prolyl cis-trans isomerase (cyclophilin), catalyzes the cis-trans isomerization of peptide bonds N-terminal to proline residues; binds the drug cyclosporin A cyclophilin|peptidyl-prolyl cis-trans isomerase (PPIase)

YKR029C	[SET3] Defining member of the SET3 histone deacetylase complex which is a meiosis-specific repressor of sporulation genes; necessary for efficient transcription by RNAPII; one of two yeast proteins that contains both SET and PHD domains
YCR033W	[SNT1] Subunit of the Set3C deacetylase complex; putative DNA-binding protein
YGL194C	[HOS2] Histone deacetylase required for gene activation via specific deacetylation of lysines in H3 and H4 histone tails; subunit of the Set3 complex, a meiotic-specific repressor of sporulation specific genes that contains deacetylase activity

0284	GO_TERM:[protein phosphatase type 2A complex] P-Value:7.2e-15
YOR014W	[RTS1] B-type regulatory subunit of protein phosphatase 2A (PP2A); homolog of the mammalian B' subunit of PP2A protein phosphatase 2A (PP2A) B-type regulatory subunit Null mutant is viable but is temperature-sensitive, hypersensitive to ethanol, and unable to grow with glycerol as the sole carbon source
YOR073W	[SGO1] Component of the spindle checkpoint, involved in sensing lack of tension on mitotic chromosomes; protects centromeric Rec8p at meiosis I; required for accurate chromosomal segregation at meiosis II and for mitotic chromosome stability
YGR161C	[RTS3] Putative component of the protein phosphatase type 2A complex
YGL190C	[CDC55] Non-essential regulatory subunit B of protein phosphatase 2A; has multiple roles in mitosis and protein biosynthesis; found in the nucleus of most cells but also at the bud neck (large-budded cells) and at the bud tip (small-budded cells) protein phosphatase 2A regulatory subunit B abnormally elongated buds, delay or partial block of septation and/or cell separation; deletion mutant is cold-sensitive
YAL016W	[TPD3] Regulatory subunit A of the heterotrimeric protein phosphatase 2A, which also contains regulatory subunit Cdc55p and either catalytic subunit Pph21p or Pph22p; required for cell morphogenesis and for transcription by RNA polymerase III protein phosphatase 2A regulatory subunit A Null mutant is viable, defective in cytokinesis at reduced temperatures, defective in transcription by RNA polymerase III at elevated temperatures; nocodazole sensitive and exhibits phenotypes of previously identified kinetochore/spindle checkpoint mutants
YDL134C	[PPH21] Catalytic subunit of protein phosphatase 2A, functionally redundant with Pph22p; methylated at C terminus; forms alternate complexes with several regulatory subunits; involved in signal transduction and regulation of mitosis Null mutant is viable, pph21 pph22 mutants produce very small spores in some strain backgrounds and are inviable in others, pph21 pph22 pph3 mutants are inviable

0285	GO_TERM:[organelle organization and biogenesis] P-Value:8.5e-01 OVERLAP:[cytoplasmic ribosomal large subunit] <500.40.10> SIZE:81
**YIL148W**	[RPL40A] Fusion protein, identical to Rpl40Bp, that is cleaved to yield ubiquitin and a ribosomal protein of the large (60S) ribosomal subunit with similarity to rat L40; ubiquitin may facilitate assembly of the ribosomal protein into ribosomes also encodes a ubiquitin protein|ribosomal protein L40A
YMR276W	[DSK2] Nuclear-enriched ubiquitin-like polyubiquitin-binding protein, required for spindle pole body (SPB) duplication and for transit through the G2/M phase of the cell cycle, involved in proteolysis, interacts with the proteasome ubiquitin-like protein
YOR042W	[CUE5] Protein containing a CUE domain that binds ubiquitin, which may facilitate intramolecular monoubiquitination; green fluorescent protein (GFP)-fusion protein localizes to the cytoplasm in a punctate pattern

0286	GO_TERM:[signalosome complex] P-Value:7.3e-20
YJR084W	[CSN12] Subunit of the Cop9 signalosome, which is required for deneddylation, or removal of the ubiquitin-like protein Rub1p from Cdc53p (cullin); involved in adaptation to pheromone signaling COP9 signalosome (CSN) subunit
YMR025W	[CSI1] Subunit of the Cop9 signalosome, which is required for deneddylation, or removal of the ubiquitin-like protein Rub1p from Cdc53p (cullin); involved in adaptation to pheromone signaling Interactor with COP9 signalosome (CSN) complex
YIL071C	[PCI8] Possible shared subunit of Cop9 signalosome (CSN) and eIF3, binds eIF3b subunit Prt1p, has possible dual functions in transcriptional and translational control, contains a PCI (Proteasome-COP9 signalosome (CSN)-eIF3) domain COP9 signalosome (CSN) subunit|translational regulator (putative)
YOL117W	[RRI2] Subunit of the COP9 signalosome (CSN) complex that cleaves the ubiquitin-like protein Nedd8 from SCF ubiquitin ligases; plays a role in the mating pheromone response COP9 signalosome (CSN) subunit Null: viable. Other phenotypes: Cdc53 accumulates exclusively in the 'rubinylated' form in an rri2-null
YDL216C	[RRI1] Catalytic subunit of the COP9 signalosome (CSN) complex that acts as an isopeptidase in cleaving the ubiquitin-like protein Nedd8 from SCF ubiquitin ligases; metalloendopeptidase involved in the adaptation to pheromone signaling COP9 signalosome (CSN) subunit Null mutant is viable; accumulates cdc53 in rub1 conjugated form
YDR179C	[CSN9] Subunit of the Cop9 signalosome, which is required for deneddylation, or removal of the ubiquitin-like protein Rub1p from Cdc53p (cullin); involved in adaptation to pheromone signaling COP9 signalosome (CSN) subunit

0287	GO_TERM:[EKC/KEOPS protein complex] P-Value:1.0e-12
YGR262C	[BUD32] Protein involved in bud-site selection; diploid mutants display a random budding pattern instead of the wild-type bipolar pattern Diploid mutants exhibit random budding
YJL184W	[GON7] Protein of unknown function, proposed to be involved in the transfer of mannosylphosphate groups onto N-linked oligosaccharides; also proposed to be involved in responding to osmotic stress
YKR038C	[KAE1] Putative glycoprotease that interacts with Bud32p, which is a member of the novel protein kinase piD261 family Putative O-sialo-glycoprotein-endopeptidase A1 Null: gene disruption is lethal
YML036W	[CGI121] Component of conserved KEOPS protein complex, which promotes telomere uncapping and elongation, and also contains protein kinase Bud32p, putative peptidase Kae1p, and Gon7p; deletion suppresses cdc13-1

0288	GO_TERM:[proteasome complex (sensu Eukaryota)] P-Value:1.7e-03
YIL007C	[NAS2] Protein with similarity to the p27 subunit of mammalian proteasome modulator; not essential; interacts with Rpn4p
YGL058W	[RAD6] Ubiquitin-conjugating enzyme (E2), involved in postreplication repair (with Rad18p), sporulation, telomere silencing, and ubiquitin-mediated N-end rule protein degradation (with Ubr1p) ubiquitin-conjugating enzyme Radiation sensitive. Defective for postreplication repair, repression of retrotransposition, meiotic gene conversion and sporulation. Mutations in srs2 suppress rad6 radiation-sensitivity but not the sporulation defect. rad6 forms recombination intermediates. mgs1 is synthetic lethal with rad6.|Deletion mutants of this post-replication repair (PRR) gene do not have any cross-link-induced mutations but show increased levels of recombination.
YDL020C	[RPN4] Transcription factor that stimulates expression of proteasome genes; Rpn4p levels are in turn regulated by the 26S proteasome in a negative feedback control mechanism; RPN4 is transcriptionally regulated by various stress responses Null mutant is viable, exhibits synthetic interactions with sen3, sun1, and cdc28-1N
YLR024C	[UBR2] Cytoplasmic ubiquitin-protein ligase (E3) ubiquitin-protein ligase (E3)

0289	GO_TERM:[ribonucleoprotein complex] P-Value:2.2e-01 OVERLAP:[eIF5A] <500.10.100> SIZE:2
YNL055C	[POR1] Mitochondrial porin (voltage-dependent anion channel), outer membrane protein required for the maintenance of mitochondrial osmotic stability and mitochondrial membrane permeability porin|voltage-dependent anion channel (VDAC) Null mutant is viable, shows strain-dependent delayed growth on glycerol
**YEL034W**	[HYP2] Translation initiation factor eIF-5A, promotes formation of the first peptide bond; similar to and functionally redundant with Anb1p; undergoes an essential hypusination modification; expressed under aerobic conditions translation initiation factor eIF-5A Null mutant is viable; a double mutant containing disruptions of both HYP2 and and the highly homologous ANB1 is inviable
YNL085W	[MKT1] Protein that forms a complex with Pbp1p that may mediate posttranscriptional regulation of HO endonuclease; involved in propagation of M2 dsRNA satellite of L-A virus retroviral protease signature protein

0290	GO_TERM:[pre-autophagosomal structure] P-Value:5.7e-06
YMR159C	[ATG16] Protein that interacts with the Atg12p-Atg5p conjugate during formation of the pre-autophagosomal structure; essential for autophagy Null mutant is viable, defective in autophagy
YPL149W	[ATG5] Conserved autophagy-related protein that undergoes conjugation with Atg12p and then associates with Atg16p to form a cytosolic complex essential for autophagosome formation reduced viability upon nutrient starvation; defective in autophagy

0291	GO_TERM:[catalytic activity] P-Value:2.0e-02
YOR209C	[NPT1] Nicotinate phosphoribosyltransferase, acts in the salvage pathway of NAD+ biosynthesis; required for silencing at rDNA and telomeres and has a role in silencing at mating-type loci; localized to the nucleus nicotinate phosphoribosyltransferase Mutations weaken silencing and also cause a reduction in the intracellular NAD(+) level.
YCR053W	[THR4] Threonine synthase, conserved protein that catalyzes formation of threonine from 0-phosphohomoserine; expression is regulated by the GCN4-mediated general amino acid control pathway threonine synthase threonine requiring
YOL151W	[GRE2] NADPH-dependent methylglyoxal reductase (D-lactaldehyde dehydrogenase); stress induced (osmotic, ionic, oxidative, heat shock and heavy metals); regulated by the HOG pathway

0292	GO_TERM:[molecular_function] P-Value:1.7e-01
YKL206C	[ADD66] Protein of unknown function involved in ER-associated protein degradation; green fluorescent protein (GFP)-fusion protein localizes to the cytoplasm
YLR199C	

0293	GO_TERM:[proteasome complex (sensu Eukaryota)] P-Value:7.6e-91 OVERLAP:[19/22S regulator] <360.10.20> SIZE:18
YFL007W	[BLM10] Proteosome activator subunit; found in association with core particles, with and without the 19S regulatory particle; required for resistance to bleomycin, may be involved in protecting against oxidative damage; similar to mammalian PA200
YDL188C	[PPH22] Catalytic subunit of protein phosphatase 2A, functionally redundant with Pph21p; methylated at C terminus; forms alternate complexes with several regulatory subunits; involved in signal transduction and regulation of mitosis protein phosphatase type 2A Null mutant is viable, pph21 pph22 mutants produce very small spores in some strain backgrounds and are inviable in others, pph21 pph22 pph3 mutants are inviable
YBR173C	[UMP1] Short-lived chaperone required for correct maturation of the 20S proteasome; degraded by proteasome upon completion of its assembly; involved in ubiquitin-mediated proteolysis; mutant defective in degradation of short-lived proteins 20S proteasome maturation factor Null mutant is viable, grows slower than wild-type, is hypersensitive to cadmium ions and canavanine, accumulates Ub-protein conjugates; ump1 null homozygous diploids fail to sporulate
YER094C	[PUP3] Beta subunit of the 20S proteasome involved in ubiquitin-dependent catabolism; human homolog is subunit C10 20S proteasome subunit beta3_sc
YJL001W	[PRE3] 20S proteasome beta-type subunit, responsible for cleavage after acidic residues in peptides 20S proteasome subunit
YBL041W	[PRE7] 20S proteasome beta-type subunit proteasome subunit
YPR103W	[PRE2] 20S proteasome beta-type subunit, responsible for the chymotryptic activity of the proteasome proteasome subunit Null mutant is inviable, pre2 mutants exhibit defects in chymotrypsin-like proteolysis, stress response and ubiquitin signaled protein degradation
YGR135W	[PRE9] 20S proteasome beta-type subunit; the only nonessential 20S subunit proteasome component Y13
YOL038W	[PRE6] 20S proteasome alpha-type subunit 20S proteasome alpha-type subunit
YMR314W	[PRE5] 20S proteasome alpha-type subunit 20S proteasome alpha-type subunit
YML092C	[PRE8] 20S proteasome beta-type subunit proteasome component Y7
YOR362C	[PRE10] 20S proteasome alpha-type subunit proteasome component YC1 (protease yscE subunit 1)
YGL011C	[SCL1] Alpha subunit of the 20S core complex of the 26S proteasome involved in the degradation of ubiquitinated substrates; essential for growth; detected in the mitochondria proteasome subunit YC7alpha/Y8 (protease yscE subunit 7) Null mutant is inviable, SCL1 is a dominant suppressor of the ts lethality of crl3
YFR050C	[PRE4] 20S proteasome beta-type subunit necessary for peptidyl glutamyl peptide hydrolyzing activity|proteasome subunit
YGR253C	[PUP2] Alpha subunit of the 20S proteasome involved in ubiquitin-dependent catabolism; human homolog is subunit zeta proteasome subunit
YOR157C	[PUP1] Endopeptidase with trypsin-like activity that cleaves after basic residues; beta-type subunit of 20S proteasome synthesized as a proprotein before being proteolytically processed for assembly into 20S particle; human homolog is subunit Z proteasome subunit (putative)
YGR184C	[UBR1] Ubiquitin-protein ligase (E3) that interacts with Rad6p/Ubc2p to ubiquitinate substrates of the N-end rule pathway; binds to the Rpn2p, Rpt1p, and Rpt6p proteins of the 19S particle of the 26S proteasome ubiquitin-protein ligase Null mutant is viable, unable to degrade substrates of the N-end rule pathway, partially defective in sporulation
YBL039C	[URA7] Major CTP synthase isozyme (see also URA8), catalyzes the ATP-dependent transfer of the amide nitrogen from glutamine to UTP, forming CTP, the final step in de novo biosynthesis of pyrimidines; involved in phospholipid biosynthesis CTP synthase Null mutant is viable; ura7 ura8 double deletion mutants are inviable
YDL040C	[NAT1] Subunit of the N-terminal acetyltransferase NatA (Nat1p, Ard1p, Nat5p); N-terminally acetylates many proteins, which influences multiple processes such as the cell cycle, heat-shock resistance, mating, sporulation, and telomeric silencing N-terminal acetyltransferase Null mutant is viable, has reduced acetyltransferase activity, derepressed silent mating type locus (HML) and fails to enter G0
YBR217W	[ATG12] Ubiquitin-like modifier, conjugated via an isopeptide bond to a lysine residue of Atg5p by the E1 enzyme, Atg7p, and the E2 enzyme, Atg10p, a step that is essential for autophagy Null mutant is viable, defective in autophagy
YBR272C	[HSM3] Protein of unknown function, involved in DNA mismatch repair during slow growth; has weak similarity to Msh1p Null mutant is viable, hsm3 null mutants exhibit enhanced rates of spontaneous mutation to canavanine resistance and reversions of lys1-1 and his1-7, increased rate of intragenic mitotic recombination at the ADE2 gene, and reduced ability to correct DNA heteroduplexes
YEL037C	[RAD23] Protein with ubiquitin-like N terminus, recognizes and binds damaged DNA (with Rad4p) during nucleotide excision repair; regulates Rad4p levels, subunit of Nuclear Excision Repair Factor 2 (NEF2); homolog of human HR23A and HR23B proteins ubiquitin-like protein radiation sensitive

**YGR232W**	[NAS6] Regulatory, non-ATPase subunit of the 26S proteasome; homolog of the human oncoprotein gankyrin, which interacts with the retinoblastoma tumor suppressor (Rb) and cyclin-dependent kinase 4/6 26S proteasome interacting protein
YGL004C	[RPN14] Putative non-ATPase subunit of the 19S regulatory particle of the 26S proteasome; localized to the cytoplasm
YER012W	[PRE1] 20S proteasome beta-type subunit; localizes to the nucleus throughout the cell cycle 22.6 kDa proteasome subunit Null mutant is inviable, pre1 mutants accumulate ubiquitin-protein conjugates
YHL030W	[ECM29] Major component of the proteasome; tethers the proteasome core particle to the regulatory particle, and enhances the stability of the proteasome
**YDL147W**	[RPN5] Essential, non-ATPase regulatory subunit of the 26S proteasome lid, similar to mammalian p55 subunit and to another S. cerevisiae regulatory subunit, Rpn7p proteasome regulatory particle subunit
YFR010W	[UBP6] Ubiquitin-specific protease situated in the base subcomplex of the 26S proteasome, releases free ubiquitin from branched polyubiquitin chains; deletion causes hypersensitivity to cycloheximide and other toxic compounds
**YHR027C**	[RPN1] Non-ATPase base subunit of the 19S regulatory particle of the 26S proteasome; may participate in the recognition of several ligands of the proteasome; contains a leucine-rich repeat (LRR) domain, a site for protein?protein interactions 26S proteasome PA700 subunit Null mutant is inviable; hrd2-1 mutation slows degradation of Hmg2p. hrd2-1 strains are sensitive to canavanine and show a global accumulation of ubiquitin-conjugated proteins, but are not temperature-sensitive
**YFR004W**	[RPN11] Metalloprotease subunit of the 19S regulatory particle of the 26S proteasome lid; couples the deubiquitination and degradation of proteasome substrates
**YER021W**	[RPN3] Essential, non-ATPase regulatory subunit of the 26S proteasome lid, similar to the p58 subunit of the human 26S proteasome; temperature-sensitive alleles cause metaphase arrest, suggesting a role for the proteasome in cell cycle control 26S proteasome regulatory module component Null mutant is inviable. RPN3 is a high copy suppressor of the nin1-1 temperature sensitive phenotype
**YKL145W**	[RPT1] One of six ATPases of the 19S regulatory particle of the 26S proteasome involved in the degradation of ubiquitinated substrates; required for optimal CDC20 transcription; interacts with Rpn12p and the E3 ubiquitin-protein ligase Ubr1p 26S protease subunit component (putative)|ATPase
**YDR394W**	[RPT3] One of six ATPases of the 19S regulatory particle of the 26S proteasome involved in the degradation of ubiquitinated substrates; substrate of N-acetyltransferase B Null mutant is inviable; yta2 is an extragenic suppressor of yme1 mutations
YDR363W-A	[SEM1] Component of the lid subcomplex of the regulatory subunit of the 26S proteasome; ortholog of human DSS1 Null mutant is viable but is temperature-sensitive in a sigma1278b background (but not in a S288C background). The null mutation suppresses the temperature sensitivity of sec3-2, sec8-9, sec10-2 and sec15-1.
**YHR200W**	[RPN10] Non-ATPase base subunit of the 19S regulatory particle (RP) of the 26S proteasome; N-terminus plays a role in maintaining the structural integrity of the RP; binds selectively to polyubiquitin chains; homolog of the mammalian S5a protein 26S proteasome component|mammalian S5a protein homolog Null mutant is viable, exhibits a modest sensitivity to amino acid analogs and has increased steady-state levels of ubiquitin-protein conjugates
**YFR052W**	[RPN12] Subunit of the 19S regulatory particle of the 26S proteasome lid; synthetically lethal with RPT1, which is an ATPase component of the 19S regulatory particle; physically interacts with Nob1p and Rpn3p 32-34 kDa protein Null mutant is inviable; nin1-1 mutant is temperature-sensitive mutant that shows i) higher rates of recombination and chromosome and plasmid loss; ii) greater sensitivity to UV irradiation; iii) at restrictive temperature, arrest in G2, failure to activate histone H1 kinase, and accumulation of polyubiquinated proteins
**YGL048C**	[RPT6] One of six ATPases of the 19S regulatory particle of the 26S proteasome involved in the degradation of ubiquitinated substrates; bound by ubiquitin-protein ligases Ubr1p and Ufd4p; localized mainly to the nucleus throughout the cell cycle ATPase
**YIL075C**	[RPN2] Subunit of the 26S proteasome, substrate of the N-acetyltransferase Nat1p Null mutant is inviable/null mutant is viable, but shows temperature sensitivity (conflicting reports)
**YDR427W**	[RPN9] Non-ATPase regulatory subunit of the 26S proteasome, has similarity to putative proteasomal subunits in other species; null mutant is temperature sensitive and exhibits cell cycle and proteasome assembly defects proteasome regulatory particle subunit Null mutant is viable, temperature sensitive; rpn9 rpn10 double deletion mutants are viable
**YOR261C**	[RPN8] Essential, non-ATPase regulatory subunit of the 26S proteasome; has similarity to the human p40 proteasomal subunit and to another S. cerevisiae regulatory subunit, Rpn11p proteasome regulatory particle subunit
**YOR117W**	[RPT5] One of six ATPases of the 19S regulatory particle of the 26S proteasome involved in the degradation of ubiquitinated substrates; recruited to the GAL1-10 promoter region upon induction of transcription
**YDL097C**	[RPN6] Essential, non-ATPase regulatory subunit of the 26S proteasome lid required for the assembly and activity of the 26S proteasome; the human homolog (S9 protein) partially rescues Rpn6p depletion proteasome regulatory particle subunit
**YOR259C**	[RPT4] One of six ATPases of the 19S regulatory particle of the 26S proteasome involved in the degradation of ubiquitinated substrates; required for spindle pole body duplication; localized mainly to the nucleus throughout the cell cycle 26S proteasome cap subunit component|ATPase Null mutant is inviable; ts mutant strain arrests as large-budded cells after 1, 2, 3 divisions with a G2 content of DNA and a monopolar spindle; unduplicated spindle pole body is enlarged as in other monopolar mutants; they also fail to arrest at G1 when starved for a single amino acid (but do arrest at G1 when deprived of all nitrogen), are resistant to cyclohexamide, and are hypersensitive to amino acid analogs, hygromycin B and 3-aminotriazole

**YDL007W**	[RPT2] One of six ATPases of the 19S regulatory particle of the 26S proteasome involved in the degradation of ubiquitinated substrates; required for normal peptide hydrolysis by the core 20S particle one of the ATPase subunits of the proteasome
YLR421C	[RPN13] Subunit of the 19S regulatory particle of the 26S proteasome lid Null mutant is viable but defective in degradation of ubiquitinated substrates.
**YPR108W**	[RPN7] Essential, non-ATPase regulatory subunit of the 26S proteasome, similar to another S. cerevisiae regulatory subunit, Rpn5p, as well as to mammalian proteasome subunits proteasome regulatory particle subunit

0294	GO_TERM:[response to abiotic stimulus] P-Value:2.7e-03
YBL061C	[SKT5] Activator of Chs3p (chitin synthase III), recruits Chs3p to the bud neck via interaction with Bni4p; has similarity to Shc1p, which activates Chs3p during sporulation Null mutant is viable, resistant to Calcofluor white, exhibits a reduction in cell wall chitin and chitin synthase III activity
YGL073W	[HSF1] Trimeric heat shock transcription factor, activates multiple genes in response to hyperthermia; recognizes variable heat shock elements (HSEs) consisting of inverted NGAAN repeats; constitutively DNA-bound; posttranslationally regulated heat shock transcription factor

0295	GO_TERM:[biopolymer metabolism] P-Value:5.3e-01
YLR256W	[HAP1] Zinc finger transcription factor involved in the complex regulation of gene expression in response to levels of heme and oxygen; the S288C sequence differs from other strain backgrounds due to a Ty1 insertion in the carboxy terminus zinc finger transcription factor of the Zn(2)-Cys(6) binuclear cluster domain type Essential for anaerobic or heme deficient growth; Null mutant is viable, deficient in expression of CYC1 and CYC7
YNL064C	[YDJ1] Protein chaperone involved in regulation of the HSP90 and HSP70 functions; involved in protein translocation across membranes; member of the DnaJ family heat shock protein|yeast dnaJ homolog (nuclear envelope protein) slow growth at 23 degrees, inviable at 37 degrees; modest mitochondrial import defect at 23 degrees, substantial import defect at 37 degrees

0296	GO_TERM:[protein folding] P-Value:4.2e-14
YDL229W	[SSB1] Cytoplasmic ATPase that is a ribosome-associated molecular chaperone, functions with J-protein partner Zuo1p; may be involved in folding of newly-made polypeptide chains; member of the HSP70 family; interacts with phosphatase subunit Reg1p HSP70 family
YML028W	[TSA1] Ubiquitous housekeeping thioredoxin peroxidase, reduces reactive oxygen, nitrogen and sulfur species using thioredoxin as hydrogen donor; mediates redox regulation of the nuclear localization of Yap1p; deletion results in mutator phenotype thioredoxin peroxidase Null mutant is viable, grows slower than wild-type under aerobic conditions
YPL106C	[SSE1] ATPase that is a component of the heat shock protein Hsp90 chaperone complex; binds unfolded proteins; member of the heat shock protein 70 (HSP70) family; localized to the cytoplasm HSP70 family|SSA1 SSE2 homolog Null mutant is viable, slow growing, shows no additive effects with sse2 null mutation; temperature sensitive in some strain backgrounds
YBL075C	[SSA3] ATPase involved in protein folding and the response to stress; plays a role in SRP-dependent cotranslational protein-membrane targeting and translocation; member of the heat shock protein 70 (HSP70) family; localized to the cytoplasm heat shock protein of HSP70 family Null mutant is viable; an intact copy of SSA3 regulated by the constitutive SSA2 promoter is capable of rescuing the inviability of an ssa1 ssa2 ssa4 strain; an intact copy of SSA3 regulated by the constitutive SSA2 promoter is capable of rescuing the inviability of an ssa1 ssa2 ssa4 strain
YLL024C	[SSA2] ATP binding protein involved in protein folding and vacuolar import of proteins; member of heat shock protein 70 (HSP70) family; associated with the chaperonin-containing T-complex; present in the cytoplasm, vacuolar membrane and cell wall HSP70 family Null mutant is viable, temperature sensitive; ssa1 ssa2 ssa4 strains are inviable; an intact copy of SSA3 regulated by the constitutive SSA2 promoter is capable of rescuing the inviability of an ssa1 ssa2 ssa4 strain
YAL005C	[SSA1] ATPase involved in protein folding and nuclear localization signal (NLS)-directed nuclear transport; member of heat shock protein 70 (HSP70) family; forms a chaperone complex with Ydj1p; localized to the nucleus, cytoplasm, and cell wall heat shock protein of HSP70 family Null mutant is viable, temperature sensitive; ssa1 ssa2 ssa4 strains are inviable; an intact copy of SSA3 regulated by the constitutive SSA2 promoter is capable of rescuing the inviability of an ssa1 ssa2 ssa4 strain
YOR027W	[STI1] Hsp90 cochaperone, interacts with the Ssa group of the cytosolic Hsp70 chaperones; activates the ATPase activity of Ssa1p; homolog of mammalian Hop protein heat shock protein also induced by canavanine and entry into stationary phase Null mutant is viable but shows slow growth at high or low temperatures; shows synthetic interactions with hsp82, cpr7, kin28 and sba1
YMR186W	[HSC82] Cytoplasmic chaperone of the Hsp90 family, redundant in function and nearly identical with Hsp82p, and together they are essential; expressed constitutively at 10-fold higher basal levels that HSP82 and induced 2-3 fold by heat shock chaperonin Null mutant is viable at 25 degrees C; ability to grow at higher temperatures varies with gene copy number

YPL240C	[HSP82] Cytoplasmic chaperone (Hsp90 family) required for pheromone signaling and negative regulation of Hsf1p; docks with the mitochondrial import receptor Tom70p for preprotein delivery; interacts with co-chaperones Cns1p, Cpr6p, Cpr7p, and Sti1p heat shock protein 90|mammalian Hsp90 homolog Null mutant is viable at 25 degrees C; ability to grow at higher temperatures varies with gene copy number

0297	
YJL057C	[IKS1] Putative serine/threonine kinase; expression is induced during mild heat stress; deletion mutants are hypersensitive to copper sulphate and resistant to sorbate; interacts with an N-terminal fragment of Sst2p Null mutant is heat shock sensitive
YMR129W	[POM152] Nuclear pore membrane glycoprotein; may be involved in duplication of nuclear pores and nuclear pore complexes during S-phase; membrane glycoprotein|nuclear pore complex subunit Null mutant is viable; overproduction inhibits cell growth; synthetically lethal with NUP170 and NUP188

0298	GO_TERM:[catalytic activity] P-Value:7.3e-02 OVERLAP:[Phenylalaninine-tRNA-ligase] <330> SIZE:3
YDL193W	[NUS1] Prenyltransferase, required for cell viability prenyltransferase
**YFL022C**	[FRS2] Alpha subunit of cytoplasmic phenylalanyl-tRNA synthetase, forms a tetramer with Frs1p to form active enzyme; evolutionarily distant from mitochondrial phenylalanyl-tRNA synthetase based on protein sequence, but substrate binding is similar phenylalanine-tRNA ligase subunit

0299	GO_TERM:[nucleus] P-Value:6.9e-01
YGL164C	[YRB30] RanGTP-binding protein, inhibits RanGAP1 (Rna1p)-mediated GTP hydrolysis of RanGTP (Gsp1p); shares similarity to proteins in other fungi but not in higher eukaryotes Overproduction of the full-length protein and complete deletion of the open reading frame reveal no obvious phenotype. Overproduction of C-term truncated forms of the protein inhibits yeast vegetative growth.
YOR185C	[GSP2] GTP binding protein (mammalian Ranp homolog) involved in the maintenance of nuclear organization, RNA processing and transport; interacts with Kap121p, Kap123p and Pdr6p (karyophilin betas); Gsp1p homolog that is not required for viability GTP-binding protein|Gsp1p homolog

0300	GO_TERM:[oxidoreductase activity, acting on the CH-OH group of donors, NAD or NADP as acceptor] P-Value:3.4e-04
YDL246C	[SOR2] Protein of unknown function, computational analysis of large-scale protein-protein interaction data suggests a possible role in fructose or mannose metabolism
YJR159W	[SOR1] Sorbitol dehydrogenase; expression is induced in the presence of sorbitol sorbitol dehydrogenase, sorbitol-induced

0301	GO_TERM:[nuclear pore] P-Value:1.3e-03
YDL207W	[GLE1] Cytoplasmic nucleoporin required for polyadenylated RNA export but not for protein import; component of Nup82p nuclear pore subcomplex; contains a nuclear export signal nuclear pore complex subunit|nuclear-export-signal (NES)-containing protein
YMR255W	[GFD1] Coiled-coiled protein of unknown function, identified as a high-copy suppressor of a dbp5 mutation Null mutant is viable; high copy suppressor of rat8-2

0302	GO_TERM:[structural molecule activity] P-Value:3.0e-07
YGL170C	[SPO74] Component of the meiotic outer plaque of the spindle pole body, involved in modifying the meiotic outer plaque that is required prior to prospore membrane formation
YJL039C	[NUP192] Essential structural subunit of the nuclear pore complex (NPC), localizes to the nuclear periphery of nuclear pores, homologous to human p205 nuclear pore complex subunit
YMR153W	[NUP53] Subunit of the nuclear pore complex (NPC), interacts with karyopherin Kap121p or with Nup170p via overlapping regions of Nup53p, involved in activation of the spindle checkpoint mediated by the Mad1p-Mad2p complex karyopherin docking complex component of the nuclear pore complex|nuclear pore complex subunit Null mutant is viable but disrupts Kap121 localization to the nuclear envelope.

YDL088C	[ASM4] Nuclear pore complex subunit, part of a subcomplex also containing Nup53p, Nup170p, and Pse1p nuclear pore complex subunit Null mutant is viable in some strain backgrounds (including CEN.PK2); however, in the FY1679 genetic background, it is inviable.
YML031W	[NDC1] Nuclear envelope protein with multiple putative transmembrane domains, required for nuclear pore complex assembly and spindle pole body duplication; required for meiosis II multiple transmembrane domains (putative)|nuclear envelope protein|nuclear pore complex subunit Null mutant is inviable. Conditional lethal mutants are available that show asymmetric chromosomal segregation during mitosis and meiosis II due to a defect in spindle pole body duplication

0303	GO_TERM:[protein import into nucleus] P-Value:1.3e-03
YDR002W	[YRB1] Ran GTpase binding protein; involved in nuclear protein import and RNA export, ubiquitin-mediated protein degradation during the cell cycle; shuttles between the nucleus and cytoplasm; is essential; homolog of human RanBP1 yrb1 (ts) mutants are defective in protein import and nuclear export
YMR235C	[RNA1] GTPase activating protein (GAP) for Gsp1p, involved in nuclear transport GTPase activating protein (GAP) for Gsp1p inviable, RNA synthesis defective

0304	GO_TERM:[nuclear pore] P-Value:1.2e-03
YKL186C	[MTR2] mRNA transport regulator, essential nuclear protein; Mex67p and Mtr2p form a mRNA export complex which binds to RNA mRNA transport regulator Null mutant is inviable; mtr2 mutants exhibit nuclear mRNA accumulation and nucleolar fragmentation
YPL169C	[MEX67] Poly(A)RNA binding protein involved in nuclear mRNA export, component of the nuclear pore; ortholog of human TAP a poly(A)+RNA binding protein

0305	GO_TERM:[protein import into nucleus] P-Value:8.6e-52 OVERLAP:[NUP84 complex] <310.40> SIZE:6
YIL115C	[NUP159] Subunit of the nuclear pore complex that is found exclusively on the cytoplasmic side, forms a subcomplex with Nup82p and Nsp1p, required for mRNA export nucleoporin Null mutant is inviable; at nonpermissive temperature, a temperature-sensitive mutant shows cessation of mRNA export without cytoplasmic accumulation of NLS-containing reporter protein, while at permissive temperature, the nuclear pore complexes are clustered; temperature-sensitive allele is synthetically lethal with nup120 and is suppressed by high copy GLE1
YJL061W	[NUP82] Subunit of the nuclear pore complex (NPC), forms a subcomplex with Nup159p and Nsp1p, interacts with Nup116p and is required for proper localization of Nup116p in the NPC 82 kDa protein, with putative coiled-coil domain, has carboxy-terminal domain, containing heptad repeats, that binds Nsp1p|nuclear pore complex subunit|nucleoporin Null mutant is inviable; cells depleted of Nup82p, or cells with temperature-sensitive Nup82p at nonpermissive temperature, show defect in poly(A)+RNA export but no major alterations in nuclear envelope structure or nuclear pore density
YNL189W	[SRP1] Karyopherin alpha homolog, forms a dimer with karyopherin beta Kap95p to mediate import of nuclear proteins, binds the nuclear localization signal of the substrate during import; may also play a role in regulation of protein degradation karyopherin alpha supressor of rpb1, cold-sensitive
YBR017C	[KAP104] Transportin, cytosolic karyopherin beta 2 involved in delivery of heterogeneous nuclear ribonucleoproteins to the nucleoplasm, binds rg-nuclear localization signals on Nab2p and Hrp1p, plays a role in cell-cycle progression karyopherin beta 2 Null mutant is viable at 23 degrees C, but fails to germinate and dies at 30 C, shows severe nuclear envelope defects
YAR002W	[NUP60] Subunit of the nuclear pore complex (NPC), functions to anchor Nup2p to the NPC in a process controlled by the nucleoplasmic concentration of Gsp1p-GTP; potential Cdc28p substrate; involved in telomere maintenance nuclear pore complex subunit
YER110C	[KAP123] Karyopherin beta, mediates nuclear import of ribosomal proteins prior to assembly into ribosomes and import of histones H3 and H4; localizes to the nuclear pore, nucleus, and cytoplasm; exhibits genetic interactions with RAI1 karyopherin beta 4
YLR293C	[GSP1] GTP binding protein (mammalian Ranp homolog) involved in the maintenance of nuclear organization, RNA processing and transport; regulated by Prp20p, Rna1p, Yrb1p, Yrb2p, Yrp4p, Yrb30p, Cse1p and Kap95p; yeast Gsp2p homolog GTP-binding protein
YOR098C	[NUP1] Nuclear pore complex (NPC) subunit, involved in protein import/export and in export of RNAs, possible karyopherin release factor that accelerates release of karyopherin-cargo complexes after transport across NPC; potential Cdc28p substrate nuclear pore complex subunit Davis and Fink (Cell 61:965-978) report that a NUP1 deletion is inviable, whereas Schlaich and Hurt (Eur J Cell Biol 127:319-332) report that NUP1 deletion is viable.
YOR160W	[MTR10] Nuclear import receptor, mediates the nuclear localization of proteins involved in mRNA-nucleus export
YLR347C	[KAP95] Karyopherin beta, forms a dimeric complex with Srp1p (Kap60p) that mediates nuclear import of cargo proteins via a nuclear localization signal (NLS), interacts with nucleoporins to guide transport across the nuclear pore complex karyopherin beta (importin 90) homolog (95 kDa) essential, ts mutant shows nuclear import defect

YJL041W	[NSP1] Essential component of the nuclear pore complex, which mediates nuclear import and export nuclear pore complex subunit
YBL079W	[NUP170] Abundant subunit of the nuclear pore complex (NPC), required for proper localization of specific nucleoporins within the NPC, involved in nuclear envelope permeability and in chromosome segregation, has similarity to Nup157p nuclear pore complex subunit Null mutant is viable; synthetically lethal with nup157, nup188, and pom152; changing NUP170 expression causes morphological abnormalities in nuclear envelope
YMR308C	[PSE1] Karyopherin/importin that interacts with the nuclear pore complex; acts as the nuclear import receptor for specific proteins, including Pdr1p, Yap1p, Ste12p, and Aft1p karyopherin Null mutant is viable but grows very slowly; overexpression of PSE1 results in enhanced protein secretion
YLR335W	[NUP2] Protein involved in nucleocytoplasmic transport, binds to either the nucleoplasmic or cytoplasmic faces of the nuclear pore complex depending on Ran-GTP levels; also has a role in chromatin organization nucleoporin Null mutant is viable; some combinations of alleles of nup1, nsp1 and nup2 are synthetically lethal
YML103C	[NUP188] Subunit of the nuclear pore complex (NPC), involved in the structural organization of the complex and of the nuclear envelope, also involved in nuclear envelope permeability, interacts with Pom152p and Nic96p nuclear pore complex subunit Null mutant is viable but exhibits abnormalities in nuclear envelope and nuclear pore morphology; dominant mutants of nup188 are temperature-sensitive and show nuclear envelope herniations; synthetically lethal with pom152, nup157, and nup170
YFR002W	[NIC96] Component of the nuclear pore complex, required for nuclear pore formation; forms a subcomplex with Nsp1p, Nup57p, and Nup49p 96 kDa nucleoporin-interacting component|nuclear pore complex subunit
YDR192C	[NUP42] Subunit of the nuclear pore complex (NPC) that localizes exclusively to the cytoplasmic side; involved in RNA export, most likely at a terminal step; interacts with Gle1p 42 kDa protein associated with nuclear pore complexes; structurally related to the FG-nucleoporin family of pore proteins|nuclear pore complex subunit Null mutant is viable, NUP42 is essential for the export of heat shock mRNAs following stress
**YLR208W**	[SEC13] Component of both the Nup84 nuclear pore sub-complex and of the COPII complex (Sar1p, Sec13p, Sec16p, Sec23p, Sec24p, Sec31p, Sfb2p, and Sfb3p) which is important for the formation of ER to Golgi transport vesicles nuclear pore complex subunit|protein involved in release of transport vesicles from the ER Null mutant is inviable; ts mutants exhibit defects in secretion.
YDR335W	[MSN5] Karyopherin involved in nuclear import and export; shown to be responsible for nuclear import of replication protein A and for export of several proteins including Swi6p, Far1p, and Pho4p; cargo dissociation involves binding to RanGTP Disruptants are not completely sterile
YKL068W	[NUP100] Subunit of the nuclear pore complex (NPC) that is localized to both sides of the pore; contains a repetitive GLFG motif that interacts with mRNA export factor Mex67p and with karyopherin Kap95p; homologous to Nup116p nuclear pore complex subunit Null mutant is viable with no obvious phenotypes; synthetically lethal with nup116 and gle2 mutants
YGR119C	[NUP57] Essential subunit of the nuclear pore complex (NPC), functions as the organizing center of an NPC subcomplex containing Nsp1p, Nup49p, Nup57p, and Nic96p nucleoporin
YGL172W	[NUP49] Subunit of the Nsp1p-Nup57p-Nup49p-Nic96p subcomplex of the nuclear pore complex (NPC), required for nuclear export of ribosomes nuclear pore complex subunit Null mutant is inviable; some nsp1 nsp49 alleles exhibit synthetic lethality
YMR047C	[NUP116] Subunit of the nuclear pore complex (NPC) that is localized to both sides of the pore; contains a repetitive GLFG motif that interacts with mRNA export factor Mex67p and with karyopherin Kap95p; homologous to Nup100p nuclear pore complex subunit Null mutant grows slowly, accumulates unspliced pre-tRNAs, acumulates poly(A)+ RNA in the nucleus, and is temperature-sensitive; at nonpermissive temperature, null mutants show membrane seals covering cytoplasmic face of nuclear pore complexes; synthetically lethal with nsp1, nup100, and nup145
**YGL100W**	[SEH1] Nuclear pore protein that is part of the evolutionarily conserved Nup84p complex (Nup84p, Nup85p, Nup120p, Nup145p, and Seh1p); homologous to Sec13p nuclear pore complex subunit
YER105C	[NUP157] Abundant subunit of the nuclear pore complex (NPC), present on both sides of the NPC, has similarity to Nup170p nuclear pore complex subunit Null mutant is viable; synthetically lethal with nup170 and nup188
**YGL092W**	[NUP145] Essential nucleoporin, catalyzes its own cleavage in vivo to generate a C-terminal fragment that assembles into the Nup84p subcomplex of the nuclear pore complex, and an N-terminal fragment of unknown function that is homologous to Nup100p nuclear pore complex subunit Null mutant is inviable, depletion of Nup145p in vivo leads rapidly to nuclear retention of polyadenylated RNAs and more slowly to cytoplasmic accumulation of a nuclear reporter protein
YKR082W	[NUP133] Subunit of the Nup84p subcomplex of the nuclear pore complex (NPC), localizes to both sides of the NPC, required to establish a normal nucleocytoplasmic concentration gradient of the GTPase Gsp1p nuclear pore complex subunit Null mutant is viable but grows slowly and is temperature-sensitive; at nonpermissive temperature, poly(A)+ RNA accumulates in nucleus (although nuclear import of karyophilic proteins is not blocked) and nuclear pores cluster; synthetically lethal with nup120
**YJR042W**	[NUP85] Subunit of the Nup84p subcomplex of the nuclear pore complex (NPC), required for assembly of the subcomplex and also for formation of the nucleocytoplasmic Gsp1p concentration gradient that plays a role in nuclear trafficking nuclear pore complex subunit Null mutant is viable but is temperature-sensitive; at nonpermissive temperature, null mutant accumulates poly(A)+ RNA and has fragmented nucleolus; at permissive temperature, nuclear envelope of null mutant detaches from nucleus

YDL116W	[NUP84] Subunit of the nuclear pore complex (NPC), forms a subcomplex with Nup85p, Nup120p, Nup145p-C, Sec13p, and Seh1p that plays a role in nuclear mRNA export and NPC biogenesis nuclear pore complex subunit|similar to mammalian Nup107p Null mutant is viable but has defects in nuclear membrane and nuclear pore complex organization and in poly(A)+ RNA transport
YKL057C	[NUP120] Subunit of the Nup84p subcomplex of the nuclear pore complex (NPC), required for even distribution of NPCs around the nuclear envelope, involved in establishment of a normal nucleocytoplasmic concentration gradient of the GTPase Gsp1p 100 kDa protein (predicted molecular weight is 120 kDa) with two leucine zipper motifs, coiled-coil region, and some homology to Nup133p|nuclear pore complex subunit Null mutant is viable but grows slower, is temperature-sensitive, and shows nucleolar fragmentation and clustering of nuclear pore complexes; at nonpermissive temperature, null mutant accumulates poly(A)+ mRNA in nucleus and shows nucleolar fragmentation and spindle defects; temperature sensitivity can be suppressed by growth in high osmolarity media; synthetically lethal with nup133 and nup159

0306	GO_TERM:[catalytic activity] P-Value:1.3e-01
YIL161W	
YNL023C	[FAP1] Protein that binds to Fpr1p (FKBP12), conferring rapamycin resistance by competing with rapamycin for Fpr1p binding; has similarity to putative transcription factors, including D. melanogaster shuttle craft and human NFX1 transcription factor homolog; similarity to Drosophila melanogaster shuttle craft protein; similarity to human NFX1 protein; similarity to human DNA-binding protein tenascin Null mutant is viable and shows no phenotype; overexpression confers rapamycin resistance
YJR104C	[SOD1] Cu, Zn superoxide dismutase; some mutations are analogous to those that cause ALS (amyotrophic lateral sclerosis) in humans Cu, Zn superoxide dismutase Null mutant is viable; dioxygen and paraquat sensitive; fails to grow on lactate as a carbon source; exhibits increased copper sensitivity; exhibits slower proliferation time due to increased length of G1; methionine auxotroph and oxygen sensitive; SOD1 is required for sporulation
YIL078W	[THS1] Threonyl-tRNA synthetase, essential cytoplasmic protein threonine-tRNA ligase
YNL135C	[FPR1] Peptidyl-prolyl cis-trans isomerase (PPIase), binds to the drugs FK506 and rapamycin; also binds to the nonhistone chromatin binding protein Hmo1p and may regulate its assembly or function peptidyl-prolyl cis-trans isomerase (PPIase)

0307	GO_TERM:[tRNA-intron endonuclease activity] P-Value:3.4e-08 OVERLAP:[tRNA splicing] <440.30.30> SIZE:11
YMR059W	[SEN15] Subunit of the tRNA splicing endonuclease, which is composed of Sen2p, Sen15p, Sen34p, and Sen54p tetrameric tRNA splicing endonuclease 15kDa subunit
YAR008W	[SEN34] Subunit of the tRNA splicing endonuclease, which is composed of Sen2p, Sen15p, Sen34p, and Sen54p; Sen34p contains the active site for tRNA 3' splice site cleavage and has similarity to Sen2p and to Archaeal tRNA splicing endonuclease tetrameric tRNA splicing endonuclease 34 kDa subunit Null mutant is inviable and shows H242A impaired 3'splice site cleavage
YLR105C	[SEN2] Subunit of the tRNA splicing endonuclease, which is composed of Sen2p, Sen15p, Sen34p, and Sen54p; Sen2p contains the active site for tRNA 5' splice site cleavage and has similarity to Sen34p and to Archaeal tRNA splicing endonuclease tRNA splicing endonuclease subunit
YGR256W	[GND2] 6-phosphogluconate dehydrogenase (decarboxylating), catalyzes an NADPH regenerating reaction in the pentose phosphate pathway; required for growth on D-glucono-delta-lactone 6-phosphogluconate dehydrogenase
YDL200C	[MGT1] DNA repair methyltransferase (6-O-methylguanine-DNA methylase) involved in protection against DNA alkylation damage 6-O-methylguanine-DNA methylase Null mutant is viable, sensitive to alkylation induced killing and mutation
YHR183W	[GND1] 6-phosphogluconate dehydrogenase (decarboxylating), catalyzes an NADPH regenerating reaction in the pentose phosphate pathway; required for growth on D-glucono-delta-lactone and adaptation to oxidative stress 6-phosphogluconate dehydrogenase
YNL014W	[HEF3] Translational elongation factor EF-3; paralog of YEF3 and member of the ABC superfamily; stimulates EF-1 alpha-dependent binding of aminoacyl-tRNA by the ribosome; normally expressed in zinc deficient cells Translation elongation factor 3 (EF-3)

0308	OVERLAP:[Phosphofructokinase] <340> SIZE:2
YIR007W	
YMR205C	[PFK2] Beta subunit of heterooctameric phosphofructokinase involved in glycolysis, indispensable for anaerobic growth, activated by fructose-2,6-bisphosphate and AMP, mutation inhibits glucose induction of cell cycle-related genes phosphofructokinase beta subunit Null mutant is viable but exhibits slow growth and decreased efficiency of glucose utilization.

0309	GO_TERM:[peptidyl-diphthamide metabolism] P-Value:3.1e-07 OVERLAP:[eEF2] <500.20.20> SIZE:2
YJL062W-A	
**YDR385W**	[EFT2] Elongation factor 2 (EF-2), also encoded by EFT1; catalyzes ribosomal translocation during protein synthesis; contains diphthamide, the unique posttranslationally modified histidine residue specifically ADP-ribosylated by diphtheria toxin translation elongation factor 2 (EF-2)
YHL014C	[YLF2] Protein of unknown function; shares weak similarity to E. coli GTP-binding protein gtp1
YMR083W	[ADH3] Mitochondrial alcohol dehydrogenase isozyme III; involved in the shuttling of mitochondrial NADH to the cytosol under anaerobic conditions and ethanol production alcohol dehydrogenase isoenzyme III
YLR043C	[TRX1] Cytoplasmic thioredoxin isoenzyme of the thioredoxin system which protects cells against both oxidative and reductive stress, forms LMA1 complex with Pbi2p, acts as a cofactor for Tsa1p, required for ER-Golgi transport and vacuole inheritance EC 1.8.4.8|thioredoxin Null mutant is viable; trx1-trx2 double mutant shows prolonged S phase, shortened G(sub)1 and methionine auxotrophy
YBL071W-A	[KTI11] Protein required for synthesis of diphthamide, which is a modified histidine residue of translation elongation factor 2 (Eft1p or Eft2p); functions with Dph1p, Dph2p, Jjj3p, and Dph5p; may act in a complex with Dph1p and Dph2p
YER001W	[MNN1] Alpha-1,3-mannosyltransferase, integral membrane glycoprotein of the Golgi complex, required for addition of alpha1,3-mannose linkages to N-linked and O-linked oligosaccharides, one of five S. cerevisiae proteins of the MNN1 family alpha-1,3-mannosyltransferase
YIL103W	[DPH1] Protein required, along with Dph2p, Kti11p, Jjj3p, and Dph5p, for synthesis of diphthamide, which is a modified histidine residue of translation elongation factor 2 (Eft1p or Eft2p); may act in a complex with Dph2p and Kti11p
YKL191W	[DPH2] Protein required, along with Dph1p, Kti11p, Jjj3p, and Dph5p, for synthesis of diphthamide, which is a modified histidine residue of translation elongation factor 2 (Eft1p or Eft2p); may act in a complex with Dph1p and Kti11p

0310	GO_TERM:[cortical cytoskeleton organization and biogenesis] P-Value:1.2e-03
YIL095W	[PRK1] Protein serine/threonine kinase; regulates the organization and function of the actin cytoskeleton through the phosphorylation of the Pan1p-Sla1p-End3p protein complex serine/threonine protein kinase Null mutant is viable. Strains that overexpress Prk1 are inviable.
YOR329C	[SCD5] Protein required for normal cortical actin organization and endocytosis; multicopy suppressor of clathrin deficiency; acts as a targeting subunit for protein phosphatase type 1
YHR020W	
YGR175C	[ERG1] Squalene epoxidase, catalyzes the epoxidation of squalene to 2,3-oxidosqualene; plays an essential role in the ergosterol-biosynthesis pathway and is the specific target of the antifungal drug terbinafine squalene monooxygenase Null mutant is inviable when cells are grown under aerobic conditions; erg1 null mutants are viable under anaerobic conditions during which ergosterol is taken up by the cells
YNL272C	[SEC2] Guanyl-nucleotide exchange factor for the small G-protein Sec4p, located on cytoplasmic vesicles; essential for post-Golgi vesicle transport GDP/GTP exchange factor accumulates secretory vesicles

0311	GO_TERM:[response to DNA damage stimulus] P-Value:8.5e-07 OVERLAP:[other DNA repair complexes] <510.180.20> SIZE:5
**YDR004W**	[RAD57] Protein that stimulates strand exchange by stabilizing the binding of Rad51p to single-stranded DNA; involved in the recombinational repair of double-strand breaks in DNA during vegetative growth and meiosis; forms heterodimer with Rad55p RecA homolog|interacts with Rad 55p by two-hybrid analysis|similar to DMC1, RAD51, and RAD55 Null mutant is viable, radiation sensitive|Deletion of this homologous recombination (HR) gene decreases psoralen-induced recombination and increases mutation frequencies.
**YDR076W**	[RAD55] Protein that stimulates strand exchange by stabilizing the binding of Rad51p to single-stranded DNA; involved in the recombinational repair of double-strand breaks in DNA during vegetative growth and meiosis; forms heterodimer with Rad57p RecA homolog|interacts with Rad51p and Rad57p by two-hybrid analysis|similar to DMC1, RAD51, RAD57 Null mutant is viable, radiation sensitive, x-ray sensitive|Deletion of this homologous recombination (HR) gene decreases psoralen-induced recombination and increases mutation frequencies.

YHR154W	[RTT107] Protein that interacts with Mms22p and is implicated in Mms22-dependent DNA repair during S phase, damage induces phosphorylation by Mec1p at one or more SQ/TQ motifs; has four BRCT domains; has a role in regulation of Ty1 transposition Null: increases transposition of Ty1
YBR228W	[SLX1] Subunit of a complex, with Slx4p, that hydrolyzes 5' branches from duplex DNA in response to stalled or converging replication forks; function overlaps with that of Sgs1p-Top3p
YLR135W	[SLX4] Subunit of a complex, with Slx1p, that hydrolyzes 5' branches from duplex DNA in response to stalled or converging replication forks; function overlaps with that of Sgs1p-Top3p

0312	GO_TERM:[translation elongation factor activity] P-Value:6.0e-15 OVERLAP:[eEF1] <500.20.10> SIZE:6
YOR091W	[TMA46] Protein of unknown function that associates with ribosomes; interacts with GTPase Rbg1p
YDR169C	[STB3] Protein that binds Sin3p in a two-hybrid assay
YLR249W	[YEF3] Translational elongation factor, stimulates the binding of aminoacyl-tRNA (AA-tRNA) to ribosomes by releasing EF-1 alpha from the ribosomal complex; contains two ABC cassettes; binds and hydrolyses ATP Translation elongation factor 3 (EF-3)
YDR234W	[LYS4] Homoaconitase, catalyzes the conversion of homocitrate to homoisocitrate, which is a step in the lysine biosynthesis pathway homoaconitase Lysine requiring
**YPR080W**	[TEF1] Translational elongation factor EF-1 alpha; also encoded by TEF2; functions in the binding reaction of aminoacyl-tRNA (AA-tRNA) to ribosomes translational elongation factor EF-1 alpha
YKL056C	[TMA19] Protein of unknown function that associates with ribosomes; homolog of translationally controlled tumor protein; green fluorescent protein (GFP)-fusion protein localizes to the cytoplasm; YKL056C is not an essential gene
**YBR118W**	[TEF2] Translational elongation factor EF-1 alpha; also encoded by TEF1; functions in the binding reaction of aminoacyl-tRNA (AA-tRNA) to ribosomes translational elongation factor EF-1 alpha
**YKL081W**	[TEF4] Translation elongation factor EF-1 gamma translation elongation factor EF-1gamma
**YAL003W**	[EFB1] Translation elongation factor 1 beta; stimulates nucleotide exchange to regenerate EF-1 alpha-GTP for the next elongation cycle; part of the EF-1 complex, which facilitates binding of aminoacyl-tRNA to the ribosomal A site translation elongation factor EF-1beta
**YPL048W**	[CAM1] Translational cofactor elongation factor-1 gamma, participates in the regulation of GTP-binding protein EF-1 alpha, may play a redundant role in the regulation of protein synthesis or another GTP-dependent process calcium and phospholipid binding protein homologous to translation elongation factor 1-gamma (EF-1gamma)

0313	GO_TERM:[sphingolipid biosynthesis] P-Value:5.3e-06
YEL022W	[GEA2] Guanine nucleotide exchange factor for ADP ribosylation factors (ARFs), involved in vesicular transport between the Golgi and ER, Golgi organization, and actin cytoskeleton organization; similar to but not functionally redundant with Gea1p ARF GTP/GDP exchange factor Null mutant is viable, synthetically lethal with gea1 null mutant
YER100W	[UBC6] Ubiquitin-conjugating enzyme involved in ER-associated protein degradation; located at the cytosolic side of the ER membrane; tail region contains a transmembrane segment at the C-terminus; substrate of the ubiquitin-proteasome pathway ubiquitin-conjugating enzyme
YJR077C	[MIR1] Mitochondrial phosphate carrier, imports inorganic phosphate into mitochondria; functionally redundant with Pic2p but more abundant than Pic2 under normal conditions Null mutant is viable on glucose containing media, but is unable to grow on a non-fermentable carbon source, shows reduced levels of mitochondrial proteins
YDR062W	[LCB2] Component of serine palmitoyltransferase, responsible along with Lcb1p for the first committed step in sphingolipid synthesis, which is the condensation of serine with palmitoyl-CoA to form 3-ketosphinganine serine palmitoyltransferase component Auxotrophic for long-chain component of sphingolipids; some mutations can suppress the Ca2+-sensitive mutant csg2
YBR058C-A	[TSC3] Protein that stimulates the activity of serine palmitoyltransferase (Lcb1p, Lcb2p) several-fold; involved in sphingolipid biosynthesis Essential for cell viability only at elevated temperatures. Dominant mutations in Lcb2p subunit of serine palmitoyltransferase suppress temp-sensitive growth phenotype of tsc3 delta null mutant.
YMR296C	[LCB1] Component of serine palmitoyltransferase, responsible along with Lcb2p for the first committed step in sphingolipid synthesis, which is the condensation of serine with palmitoyl-CoA to form 3-ketosphinganine serine palmitoyltransferase component Null mutant is auxotrophic for long-chain component of sphingolipids; homozygous lcb1 diploids fail to sporulate

0314	GO_TERM:[branched-chain-amino-acid transaminase activity] P-Value:1.2e-06

YDR171W	[HSP42] Small cytosolic stress-induced chaperone that forms barrel-shaped oligomers and suppresses the aggregation of non-native proteins; oligomer dissociation is not required for function; involved in cytoskeleton reorganization after heat shock Null mutant is viable; hsp42 hsp26 double deletion mutants are viable; hsp42 null mutants subjected to moderate thermal stress reorganize the actin cytoskeleton more slowly than wild-type
YHR152W	[SPO12] Nucleolar protein of unknown function, positive regulator of exit from mitosis; involved in regulating the release of Cdc14p from the nucleolus in early anaphase; proposed to play similar role in meiosis 20 kDa protein with negatively charged C-terminus required for function|positive regulator of exit from M-phase in mitosis and meiosis (putative) sporulation defective; loss of function in mitosis results in delay in G2; loss of function in meiosis results in a prolonged pachytene stage and presence of synaptonemal complexes, a single meiosis II-like equational division at the time of meiosis II, and dyad asci containing two diploid spores. Gain of function in mitosis suppresses M-phase anaphase arrest caused by overexpression of CLB2 deg- and mutants (e.g. dbf2-ts). mRNA is cell cycle regulated (with DBF2) in mitosis and increases 5-10x in meiosis.
YHR208W	[BAT1] Mitochondrial branched-chain amino acid aminotransferase, homolog of murine ECA39; highly expressed during logarithmic phase and repressed during stationary phase branched-chain amino acid transaminase|highly similar to mammalian ECA39, which is regulated by the oncogene myc Null mutant is viable; ILV auxotrophy in bat1 bat2 double mutant
YJR148W	[BAT2] Cytosolic branched-chain amino acid aminotransferase, homolog of murine ECA39; highly expressed during stationary phase and repressed during logarithmic phase branched-chain amino acid transaminase

0315	GO_TERM:[catalytic activity] P-Value:7.3e-02
YKL157W	[APE2] Zinc-dependent metallopeptidase yscII, may have a role in obtaining leucine from dipeptide substrates; sequence coordinates have changed since RT-PCR analysis showed that the adjacent ORF YKL158W comprises the 5' exon of APE2/YKL157W aminopeptidase yscII
YLR304C	[ACO1] Aconitase, required for the tricarboxylic acid (TCA) cycle and also independently required for mitochondrial genome maintenance; component of the mitochondrial nucleoid; mutation leads to glutamate auxotrophy aconitase

0316	GO_TERM:[transporter activity] P-Value:3.2e-03
YJR132W	[NMD5] Karyopherin, a carrier protein involved in nuclear import of proteins; importin beta homolog Upf1p interacting protein|importin beta homolog Kap119p Null mutant is viable, exhibits mislocalization of TFIIS and Hog1p
YNL323W	[LEM3] Membrane protein of the plasma membrane and ER, involved in translocation of phospholipids and alkylphosphocholine drugs across the plasma membrane membrane glycoprotein Null mutant sensitive to brefeldin A, shows increased glucocorticoid receptor activity in response to dexamethasone. Disruption showed marked decrease in internalization of phosphatidylethanolamine and phosphatidylcholine.

0317	GO_TERM:[cytoskeleton] P-Value:8.7e-03 OVERLAP:[Chaperonine containing T-complex TRiC (TCP RING Complex)] <130> SIZE:8
YDR510W	[SMT3] Ubiquitin-like protein of the SUMO family, conjugated to lysine residues of target proteins; regulates chromatid cohesion, chromosome segregation, APC-mediated proteolysis, DNA replication and septin ring dynamics isolated as suppressor of mif2 (centromeric protein) mutation
**YJL008C**	[CCT8] Subunit of the cytosolic chaperonin Cct ring complex, related to Tcp1p, required for the assembly of actin and tubulins in vivo chaperonin containing T-complex subunit eight component

0318	GO_TERM:[catalytic activity] P-Value:2.0e-02
YKL152C	[GPM1] Tetrameric phosphoglycerate mutase, mediates the conversion of 3-phosphoglycerate to 2-phosphoglycerate during glycolysis and the reverse reaction during gluconeogenesis phosphoglycerate mutase Required for sporulation
YPL061W	[ALD6] Cytosolic aldehyde dehydrogenase that is activated by Mg2+ and utilizes NADP+ as the preferred coenzyme; required for the conversion of acetaldehyde to acetate; constitutively expressed aldehyde dehydrogenase Null mutant is viable, grows at approximately one-third the rate of wild-type, unable to grow on ethanol as a carbon source
YPL226W	[NEW1] ATP binding cassette family member; Asn/Gln-rich rich region supports [NU+] prion formation, susceptibility to [PSI+] prion induction and aggregation of a fragment of the human Machado-Joseph Disease protein

0319	GO_TERM:[cytoplasmic part] P-Value:7.9e-01

YDL052C	[SLC1] 1-acyl-sn-gylcerol-3-phosphate acyltransferase, catalyzes the acylation of lysophosphatidic acid to form phosphatidic acid, a key intermediate in lipid metabolism; located in lipid particles and endoplasmic reticulum 1-acyl-sn-gylcerol-3-phosphate acyl transferase (putative) slc1-1 mutant suppresses sphingolipid long chain biosynthetic defect; the mutant also makes novel phosphatidylinositol derivatives and lacks sphingolipids
YDR170C	[SEC7] Guanine nucleotide exchange factor (GEF) for ADP ribosylation factors involved in proliferation of the Golgi, intra-Golgi transport and ER-to-Golgi transport; found in the cytoplasm and on Golgi-associated coated vesicles guanine nucleotide exchange protein for ARF

0320	GO_TERM:[amino acid biosynthesis] P-Value:3.4e-03 OVERLAP:[Arginine-specific carbamoylphosphate synthase] <80> SIZE:2
YER086W	[ILV1] Threonine deaminase, catalyzes the first step in isoleucine biosynthesis; expression is under general amino acid control; ILV1 locus exhibits highly positioned nucleosomes whose organization is independent of known ILV1 regulation threonine deaminase
**YJR109C**	[CPA2] Large subunit of carbamoyl phosphate synthetase, which catalyzes a step in the synthesis of citrulline, an arginine precursor carbamyl phosphate synthetase

0321	GO_TERM:[cell organization and biogenesis] P-Value:4.1e-01
YHL011C	[PRS3] 5-phospho-ribosyl-1(alpha)-pyrophosphate synthetase, involved in nucleotide, histidine, and tryptophan biosynthesis; one of a five related enzymes, which are active as heteromultimeric complexes ribose-phosphate pyrophosphokinase
YLR259C	[HSP60] Tetradecameric mitochondrial chaperonin required for ATP-dependent folding of precursor polypeptides and complex assembly; prevents aggregation and mediates protein refolding after heat shock; role in mtDNA transmission; similarity to groEL chaperonin|groEL homolog

0322	GO_TERM:[polar microtubule] P-Value:6.8e-07 OVERLAP:[Tubulins] <140.30.10> SIZE:4
YKL010C	[UFD4] Ubiquitin-protein ligase (E3) that interacts with Rpt4p and Rpt6p, two subunits of the 19S particle of the 26S proteasome; cytoplasmic E3 involved in the degradation of ubiquitin fusion proteins ubiquitin ligase e3 Null is viable; defective in proteolysis of fusion proteins containing a 'nonremovable' N-terminal ubiquitin moiety
YDR214W	[AHA1] Co-chaperone that binds to Hsp82p and activates its ATPase activity; similar to Hch1p; expression is regulated by stresses such as heat shock Hsp90 system cochaperone; Aha1 binds to the middle domain of Hsp90 and improves client protein activation in vivo
YIL094C	[LYS12] Homo-isocitrate dehydrogenase, an NAD-linked mitochondrial enzyme required for the fourth step in the biosynthesis of lysine, in which homo-isocitrate is oxidatively decarboxylated to alpha-ketoadipate homo-isocitrate dehydrogenase Null mutant is viable but shows decreased growth in the absence of lysine
YGL195W	[GCN1] Positive regulator of the Gcn2p kinase activity, forms a complex with Gcn20p; proposed to stimulate Gcn2p activation by an uncharged tRNA translational activator of GCN4 through activation of GCN2 in response to starvation Null mutant is viable and sensitive to 3-aminotriazole
**YFL037W**	[TUB2] Beta-tubulin; associates with alpha-tubulin (Tub1p and Tub3p) to form tubulin dimer, which polymerizes to form microtubules beta-tubulin null is inviable; conditional mutants show block of mitotic nuclear migration and chromosome segregation and defects in spindle and/or cytoplasmic microtubules at non-permissive conditions; some mutants are benomyl-hypersensitive
**YML085C**	[TUB1] Alpha-tubulin; associates with beta-tubulin (Tub2p) to form tubulin dimer, which polymerizes to form microtubules alpha-tubulin Null mutant is inviable; heterozygous tub1 null diploids are slow growing and sporulate poorly
YMR012W	[CLU1] eIF3 component of unknown function; deletion causes defects in mitochondrial organization but not in growth or translation initiation, can rescue cytokinesis and mitochondrial organization defects of the Dictyostelium cluA- mutant Sometimes copurifies with translation initiation factor eIF3, but apparently not required for translation initiation Null mutant is viable, growth is normal, mitochondrial network is collapsed to one side of the cell
YLL040C	[VPS13] Protein of unknown function; heterooligomeric or homooligomeric complex; peripherally associated with membranes; homologous to human COH1; involved in sporulation, vacuolar protein sorting and protein-Golgi retention
YFR009W	[GCN20] Positive regulator of the Gcn2p kinase activity, forms a complex with Gcn1p; proposed to stimulate Gcn2p activation by an uncharged tRNA ATP-binding cassette (ABC) family Null mutant is viable and shows impaired derepression of GCN4 translation and reduced levels of eIF-2 alpha phosphorylation
YLR153C	[ACS2] Acetyl-coA synthetase isoform, required for growth on glucose; expressed under anaerobic conditions acetyl CoA synthetase Null mutant is viable, and grows on ethanol or acetate as sole carbon source, but is unable to grow on glucose as sole carbon source; acs1 acs2 double null mutant is inviable

YKL211C	[TRP3] Bifunctional enzyme exhibiting both indole-3-glycerol-phosphate synthase and anthranilate synthase activities, forms multifunctional hetero-oligomeric anthranilate synthase:indole-3-glycerol phosphate synthase enzyme complex with Trp2p anthranilate synthase component II|indole-3-phosphate Null mutant is viable, tryptophan auxotroph
**YML124C**	[TUB3] Alpha-tubulin; associates with beta-tubulin (Tub2p) to form tubulin dimer, which polymerizes to form microtubules; expressed at lower level than Tub1p alpha-tubulin Null mutant is viable, hypersensitive to benomyl, exhibits poor spore viability
YOR133W	[EFT1] Elongation factor 2 (EF-2), also encoded by EFT2; catalyzes ribosomal translocation during protein synthesis; contains diphthamide, the unique posttranslationally modified histidine residue specifically ADP-ribosylated by diphtheria toxin translation elongation factor 2 (EF-2)
YER095W	[RAD51] Strand exchange protein, forms a helical filament with DNA that searches for homology; involved in the recombinational repair of double-strand breaks in DNA during vegetative growth and meiosis; homolog of Dmc1p and bacterial RecA protein Rad51p colocalizes to ~ 65 spots with Dmc1p prior to synapsis (independently of ZIP1 and DMC1), and interacts with Rad52p and Rad55p; human Rad51p homolog interacts with Brca2 protein which has been implicated in causing breast cancer|RecA homolog Null mutant is viable; accumulates meiosis-specific double strand breaks at a recombination hotspot and reduces the formation of physical recombinants and processed double strand breaks with long heterogeneous tails; rad51 mutants are also defective for X-ray damage repair and gene conversions; rad51 rad27 mutants are inviable.|Deletion of this homologous recombination (HR) gene decreases psoralen-induced recombination and increases mutation frequencies.
YGR234W	[YHB1] Nitric oxide oxidoreductase, flavohemoglobin involved in nitric oxide detoxification; plays a role in the oxidative and nitrosative stress responses flavohemoglobin Null mutant is viable. A rho+ strain carrying a yhb1(-) deletion has normal levels of both cyanide-sensitive and cyanide-insensitive respiration. Cells that carry a yhb1(-) deletion are sensitive to conditions that promote oxidative stress.
YDR127W	[ARO1] Pentafunctional arom protein, catalyzes steps 2 through 6 in the biosynthesis of chorismate, which is a precursor to aromatic amino acids 3-dehydroquinate dehydratase (3-dehydroquinase)|3-dehydroquinate synthase|epsp synthase|pentafunctional arom polypeptide|shikimate 5-dehydrogenase|shikimate kinase aromatic amino acid requiring; lack of premeiotic DNA synthesis; blocked sporulation in homozygous mutant
YKL104C	[GFA1] Glutamine-fructose-6-phosphate amidotransferase, catalyzes the formation of glucosamine-6-P and glutamate from fructose-6-P and glutamine in the first step of chitin biosynthesis glucoseamine-6-phosphate synthase|glutamine_fructose-6-phosphate amidotransferase Null mutant is viable, glucosamine auxotroph
YDL055C	[PSA1] GDP-mannose pyrophosphorylase (mannose-1-phosphate guanyltransferase), synthesizes GDP-mannose from GTP and mannose-1-phosphate in cell wall biosynthesis; required for normal cell wall structure GDP-mannose pyrophosphorylase|mannose-1-phosphate guanyltransferase
YKL029C	[MAE1] Mitochondrial malic enzyme, catalyzes the oxidative decarboxylation of malate to pyruvate, which is a key intermediate in sugar metabolism and a precursor for synthesis of several amino acids malic enzyme null mutant exhibits no malic enzyme activity and synthetic phenotypes with pyk1 and pyk2 mutations
YLR180W	[SAM1] S-adenosylmethionine synthetase, catalyzes transfer of the adenosyl group of ATP to the sulfur atom of methionine; one of two differentially regulated isozymes (Sam1p and Sam2p)

0323	GO_TERM:[transferase activity] P-Value:9.6e-02
YDR186C	
YBR125C	[PTC4] Cytoplasmic type 2C protein phosphatase; identified as a high-copy number suppressor of the synthetic lethality of a cnb1 mpk1 double deletion; overexpression decreases high-osmolarity induced Hog1p phosphorylation and kinase activity type 2C protein phosphatase
YDR071C	[PAA1] Polyamine acetyltransferase; acetylates polyamines such as putrescine, spermidine and spermine; may be involved in transcription and/or DNA replication via regulation of levels of polyamines bound to chromosomal DNA polyamine acetyltransferase Null: Mutant is viable. It is somewhat HU sensitive. It shows genetic interactions with gcn5, spt8 and spt15
YDR247W	[VHS1] Cytoplasmic serine/threonine protein kinase; identified as a high-copy suppressor of the synthetic lethality of a sis2 sit4 double mutant, suggesting a role in G1/S phase progression; homolog of Sks1p

0324	GO_TERM:[regulation of transcription] P-Value:4.0e-02
YER064C	
YPL026C	[SKS1] Putative serine/threonine protein kinase; involved in the adaptation to low concentrations of glucose independent of the SNF3 regulated pathway Null mutant is viable; Sks1 lacking the consensus ATP binding site cannot suppress snf3 mutants when overexpressed

0325	GO_TERM:[IMP cyclohydrolase activity] P-Value:5.0e-06
YCR073W-A	[SOL2] Protein with a possible role in tRNA export; shows similarity to 6-phosphogluconolactonase non-catalytic domains but does not exhibit this enzymatic activity; homologous to Sol1p, Sol3p, and Sol4p
YLR257W	
YMR120C	[ADE17] Enzyme of 'de novo' purine biosynthesis containing both 5-aminoimidazole-4-carboxamide ribonucleotide transformylase and inosine monophosphate cyclohydrolase activities, isozyme of Ade16p; ade16 ade17 mutants require adenine and histidine 5-aminoimidazole-4-carboxamide ribonucleotide (AICAR) transformylase/IMP cyclohydrolase Null mutant is viable; ade16 ade17 double mutants require adenine
YLR028C	[ADE16] Enzyme of 'de novo' purine biosynthesis containing both 5-aminoimidazole-4-carboxamide ribonucleotide transformylase and inosine monophosphate cyclohydrolase activities, isozyme of Ade17p; ade16 ade17 mutants require adenine and histidine 5-aminoimidazole-4-carboxamide ribonucleotide (AICAR) transformylase/IMP cyclohydrolase Null mutant is viable; ade16 ade17 double mutant requires adenine
YNR034W	[SOL1] Protein with a possible role in tRNA export; shows similarity to 6-phosphogluconolactonase non-catalytic domains but does not exhibit this enzymatic activity; homologous to Sol2p, Sol3p, and Sol4p

0326	GO_TERM:[phosphopantothenoylcysteine decarboxylase activity] P-Value:2.0e-05 OVERLAP:[Serine/threonine phoshpoprotein phosphatase] <450> SIZE:6
YKR072C	[SIS2] Negative regulatory subunit of the protein phosphatase 1 Ppz1p; involved in ion homeostasis and cell cycle progression
YMR311C	[GLC8] Regulatory subunit of protein phosphatase 1 (Glc7p), involved in glycogen metabolism and chromosome segregation; proposed to regulate Glc7p activity via conformational alteration; ortholog of the mammalian protein phosphatase inhibitor 2 protein phosphatase 1 (Glc7p) regulator
YOR054C	[VHS3] Functionally redundant (see also SIS2) inhibitory subunit of Ppz1p, a PP1c-related ser/thr protein phosphatase Z isoform; synthetically lethal with sis2; putative phosphopantothenoylcysteine decarboxylase involved in coenzyme A biosynthesis
YDR436W	[PPZ2] Serine/threonine protein phosphatase Z, isoform of Ppz1p; involved in regulation of potassium transport, which affects osmotic stability, cell cycle progression, and halotolerance Null mutant is viable but shows increase in cell size and cell lysis (remediated by 1 M sorbitol); ppz1 ppz2 double mutant shows increased expression of ENA1, resistance to sodium and lithium, and sensitivity to 5 mM caffeine (which is suppressed by 1 M sorbitol)
YML016C	[PPZ1] Serine/threonine protein phosphatase Z, isoform of Ppz2p; involved in regulation of potassium transport, which affects osmotic stability, cell cycle progression, and halotolerance Null mutant is viable, exhibits increased tolerance to Na+ and Li+ cations, increased cell size and lysis; ppz1 ppz2 double deletion mutants exhibit a temperature sensitive cell lysis defect and fail to grow in the presence of 5 mM caffeine

0327	GO_TERM:[negative regulation of ligase activity] P-Value:2.6e-04
YBR050C	[REG2] Regulatory subunit of the Glc7p type-1 protein phosphatase; involved with Reg1p, Glc7p, and Snf1p in regulation of glucose-repressible genes, also involved in glucose-induced proteolysis of maltose permease Glc7p regulatory subunit
YKR098C	[UBP11] Ubiquitin-specific protease that cleaves ubiquitin from ubiquitinated proteins ubiquitin-specific protease
YGR097W	[ASK10] Component of the RNA polymerase II holoenzyme, phosphorylated in response to oxidative stress; has a role in destruction of Ssn8p, which relieves repression of stress-response genes transcriptional activator of the SKN7 mediated 'two-component' regulatory system
YPR030W	[CSR2] Nuclear protein with a potential regulatory role in utilization of galactose and nonfermentable carbon sources; overproduction suppresses the lethality at high temperature of a chs5 spa2 double null mutation; potential Cdc28p substrate
YDR017C	[KCS1] Inositol hexaphosphate kinase, phosphorylates inositol hexakisphosphate (InsP6) to diphosphoinositol polyphosphates, required for proper vacuole morphology and involved in salt stress response, contains two leucine heptad repeats Inositol polyphosphate kinase Null mutant is viable; kcs1 ptc1 double mutant is inviable; isolated as a suppressor of a hyper-recombination mutant of PKC1
YDR099W	[BMH2] 14-3-3 protein, minor isoform; binds proteins and DNA, involved in regulation of many processes including exocytosis and vesicle transport, Ras/MAPK signaling during pseudohyphal development, rapamycin-sensitive signaling, and others member of conserved eukaryotic 14-3-3 gene family Null mutant is viable; bmh1 bmh2 double mutant is inviable; (in strain Sigma-1278b, required for pseudohyphal development but not for viability)

YDR130C	[FIN1] Spindle pole body-related intermediate filament protein, forms cell cycle-specific filaments between spindle pole bodies in mother and daughter cells, able to self-assemble, expression induced during S/G2, localization cell-cycle dependent
YER177W	[BMH1] 14-3-3 protein, major isoform; binds proteins and DNA, involved in regulation of many processes including exocytosis and vesicle transport, Ras/MAPK signaling during pseudohyphal development, rapamycin-sensitive signaling, and others member of conserved eukaryotic 14-3-3 gene family Null mutant is viable; bmh1 bmh2 double mutant is inviable; (in strain Sigma-1278b, required for pseudohyphal development but not for viability)

0328	
YMR102C	
YNL218W	[MGS1] Protein with DNA-dependent ATPase and ssDNA annealing activities involved in maintenance of genome; interacts functionally with DNA polymerase delta; homolog of human Werner helicase interacting protein (WHIP) mgs1 is synthetic lethal with rad6 and exhibits a synergistic growth defect with rad18 and rad5. mgs1 mutant is not sensitive to DNA-damaging agents, but mgs1 rad5 double mutant has increased sensitivity to hydroxyurea and a greatly increased spontaneous mutation rate. Growth defects of mgs1 rad18 double mutants are suppressed by a mutation in SRS2 or by overexpression of Rad52. mgs1 mutation suppresses temperature sensitivity of POL3 mutants.

0329	GO_TERM:[transferase activity] P-Value:1.8e-02
YOR267C	[HRK1] Protein kinase implicated in activation of the plasma membrane H(+)-ATPase Pma1p in response to glucose metabolism; plays a role in ion homeostasis protein kinase similar to Npr1
YPR160W	[GPH1] Non-essential glycogen phosphorylase required for the mobilization of glycogen, activity is regulated by cyclic AMP-mediated phosphorylation, expression is regulated by stress-response elements and by the HOG MAP kinase pathway glycogen phosphorylase Null mutant is viable; haploid cells contain higher levels of intracellular glycogen

0330	GO_TERM:[phosphatase regulator activity] P-Value:2.2e-05 OVERLAP:[Serine/threonine phoshpoprotein phosphatase] <450> SIZE:6
YFR003C	[YPI1] Inhibitor of the type I protein phosphatase Glc7p, which is involved in regulation of a variety of metabolic processes; overproduction causes decreased cellular content of glycogen PP1 phosphatase inhibitor
**YKL193C**	[SDS22] Conserved nuclear regulatory subunit of Glc7p type 1 protein serine-threonine phosphatase (PP1), functions positively with Glc7p to promote dephosphorylation of nuclear substrates required for chromosome transmission during mitosis Glc7p regulatory subunit

0331	GO_TERM:[AMP-activated protein kinase activity] P-Value:5.6e-12 OVERLAP:[SNF1 complex] <470.10> SIZE:6
**YDR422C**	[SIP1] Alternate beta-subunit of the Snf1p kinase complex, may confer substrate specificity; vacuolar protein containing KIS (Kinase-Interacting Sequence) and ASC (Association with Snf1 kinase Complex) domains involved in protein interactions protein kinase complex component Null mutant is viable, exhibits a slight increase in GAL gene expression
YER129W	[SAK1] Upstream kinase for the SNF1 complex; partially redundant function with Elm1p and Tos3p; members of this family of kinases have functional orthology with LKB1, a mammalian kinase associated with Peutz-Jeghers cancer-susceptibility syndrome
YDR419W	[RAD30] DNA polymerase eta, involved in the predominantly error-free bypass replication of DNA lesions, catalyzes the efficient and accurate synthesis of DNA opposite cyclobutane pyrimidine dimers; homolog of human POLH and bacterial DinB proteins DNA polymerase eta Null mutant shows increased sensitivity to UV (254 nm). Deletion of RAD30 did not affect spontaneous mutagenesis. Overproduction of Rad30p slightly mutagenic in wild-type yeast strain, moderately mutagenic in strains with inactive 3'->5'-exonuclease of DNA polymerase epsilon or DNA mismatch repair.
**YGL208W**	[SIP2] One of three beta subunits of the Snf1 serine/threonine protein kinase complex involved in the response to glucose starvation; null mutants exhibit accelerated aging; N-myristoylprotein localized to the cytoplasm and the plasma membrane
**YDR477W**	[SNF1] AMP-activated serine/threonine protein kinase found in a complex containing Snf4p and members of the Sip1p/Sip2p/Gal83p family; required for transcription of glucose-repressed genes, thermotolerance, sporulation, and peroxisome biogenesis serine/threonine kinase Null mutant is viable, sensitive to heat stress and starvation and fails to accumulate glycogen during growth in rich medium; sucrose nonfermenting, high copy MSI1 and PDE2 partially suppress snf1 sporulation defects

**YGL115W**	[SNF4] Protein kinase activator found in a complex containing Snf1p and members of the Sip1p/Sip2p/Gal83p family; activates the Snf1p protein kinase; involved in expression of glucose-repressed genes, sporulation, and peroxisome biogenesis associates with Snf1p Null mutant is viable, sucrose nonfermenting; high copy MSI1 and PDE2 partially suppress sporulation defect
YDR028C	[REG1] Regulatory subunit of type 1 protein phosphatase Glc7p, involved in negative regulation of glucose-repressible genes Glc7p regulatory subunit
**YER027C**	[GAL83] One of three possible beta-subunits of the Snf1 kinase complex, allows nuclear localization of the Snf1 kinase complex in the presence of a nonfermentable carbon source; contains glycogen-binding domain

0332	GO_TERM:[regulation of transcription from RNA polymerase II promoter] P-Value:4.5e-06 OVERLAP:[ARG complex] <510.190.120> SIZE:4
YCL066W	[HMLALPHA1] Silenced copy of ALPHA1 at HML, encoding a transcriptional coactivator involved in the regulation of mating-type alpha-specific gene expression involved in the regulation of alpha-specific genes|transcription factor
YCL067C	[HMLALPHA2] Silenced copy of ALPHA2 at HML; homeobox-domain protein that associates with Mcm1p in haploid cells to repress a-specific gene expression and interacts with a1p in diploid cells to repress haploid-specific gene expression
**YML099C**	[ARG81] Zinc-finger transcription factor of the Zn(2)-Cys(6) binuclear cluster domain type, involved in the regulation of arginine-responsive genes; acts with Arg80p and Arg82p zinc finger transcription factor of the Zn(2)-Cys(6) binuclear cluster domain type
**YMR043W**	[MCM1] Transcription factor involved in cell-type-specific transcription and pheromone response; plays a central role in the formation of both repressor and activator complexes contains the 56 amino-acid MADS (MCM1, AG, DEFAm SRF)-box motif within its DNA binding domain, plays a central role in the formation of both repressor and activator complexes|transcription factor Null mutant is inviable, Pro97Leu mutant is sterile, exhibits defects in minichromosome maintenance
**YDR173C**	[ARG82] Protein involved in regulation of arginine-responsive and Mcm1p-dependent genes; has a dual-specificity inositol polyphosphate kinase activity required for regulation of phosphate- and nitrogen-responsive genes dual specificity inositol 1,4,5-trisphosphate 6-kinase/inositol 1,4,5,6-tetrakisphosphate 3-kinase (IP3 6-/IP4 3-kinase) Null mutant is viable but requires arginine at 23C; growth defect at 30C; inviable at 37C; null is defective in sporulation, mating, amino acid metabolism (fails to grow on medium in which arginine or ornithine is the sole nitrogen source); null mutants accumulate IP3, I(4,5)P2 and have drastically reduced levels of IP4, IP5 and IP6.
**YMR042W**	[ARG80] Transcription factor involved in regulation of arginine-responsive genes; acts with Arg81p and Arg82p transcription factor Arginine requiring

0333	GO_TERM:[histone deacetylase complex] P-Value:3.5e-16 OVERLAP:[HDB complex] <240.20> SIZE:3
YMR019W	[STB4] Protein that binds Sin3p in a two-hybrid assay; contains a Zn(II)2Cys6 zinc finger domain characteristic of DNA-binding proteins Null mutant is viable, hypersensitive to caffeine
YFL031W	[HAC1] bZIP transcription factor (ATF/CREB1 homolog) that regulates the unfolded protein response, via UPRE binding, and membrane biogenesis; ER stress-induced splicing pathway utilizing Ire1p, Trl1p and Ada5p facilitates efficient Hac1p synthesis bZIP (basic-leucine zipper) protein Null mutant is viable but is sensitive to caffeine (suppressed by high-copy SRA5) and stresses that produce unfolded proteins. High-copy HAC1 suppresses S. pombe cdc10-129
YMR075W	[RCO1] Essential subunit of the histone deacetylase Rpd3S complex; interacts with Eaf3p
**YOL004W**	[SIN3] DNA binding subunit of Sin3p-Rpd3p histone deacetylase complex, involved in transcriptional repression of meiosis-specific genes during vegetative growth and silencing; involved in telomere maintenance DNA binding protein|involved in transcriptional regulation inviable, reduced potassium dependency
YPL181W	[CTI6] Protein that relieves transcriptional repression by binding to the Cyc8p-Tup1p corepressor and recruiting the SAGA complex to the repressed promoter; contains a PHD finger domain
YNL097C	[PHO23] Probable component of the Rpd3 histone deacetylase complex, involved in transcriptional regulation of PHO5; C-terminus has similarity to human candidate tumor suppressor p33(ING1) Null mutant is viable but shows constitutive PHO5 expression
YKL185W	[ASH1] Zinc-finger inhibitor of HO transcription; mRNA is localized and translated in the distal tip of anaphase cells, resulting in accumulation of Ash1p in daughter cell nuclei and inhibition of HO expression; potential Cdc28p substrate zinc finger transcription factor Mutant ash1 daughters can transcribe HO and switch mating type
YDR207C	[UME6] Key transcriptional regulator of early meiotic genes, binds URS1 upstream regulatory sequence, couples metabolic responses to nutritional cues with initiation and progression of meiosis, forms complex with Ime1p, and also with Sin3p-Rpd3p C6 zinc finger URS1-binding protein Null mutant is viable. Exhibits defects in IME1-dependent activation and repression through URS1 sites. ume6 does not require Mata/Matalpha, starvation, IME1, or IME2 for derepressed expression in mitosis and is epistatic to lethality of IME1 overexpression in haploids.
**YNL330C**	[RPD3] Histone deacetylase; regulates transcription and silencing histone deacetylase Null mutant is viable and shows reduced potassium dependency, mating defects, hypersensitivity to cycloheximide, and constitutive derepression of acid phosphatase; mutant epistasis analysis indicates that RPD3 acts in the same pathway as UME4/SIN3; homozygous mutant diploid is defective in sporulation and recombination

YPL139C	[UME1] Negative regulator of meiosis, required for repression of a subset of meiotic genes during vegetative growth, binding of histone deacetylase Rpd3p required for activity, contains a NEE box and a WD repeat motif; homologous with Wtm1p, Wtm2p transcriptional modulator Null mutant is viable, expression of the meiotic gene IME2 in null haploid
YIL084C	[SDS3] Component of the Rpd3p/Sin3p deacetylase complex required for its structural integrity and catalytic activity, involved in transcriptional silencing and required for sporulation; cells defective in SDS3 display pleiotropic phenotypes extragenic suppressor of a silencing defective rap 1s hmr delta A strain, sporulation defects
YDL076C	[RXT3] Protein of unknown function, may be involved in chromatin silencing
**YMR263W**	[SAP30] Subunit of a histone deacetylase complex, along with Rpd3p and Sin3p, that is involved in silencing at telomeres, rDNA, and silent mating-type loci; involved in telomere maintenance
YAL013W	[DEP1] Transcriptional modulator involved in regulation of structural phospholipid biosynthesis genes and metabolically unrelated genes, as well as maintenance of telomeres, mating efficiency, and sporulation
YBR095C	[RXT2] Subunit of the histone deacetylase Rpd3L complex; possibly involved in cell fusion and invasive growth

0334	GO_TERM:[aminopeptidase activity] P-Value:1.1e-04
YHR113W	
YOR302W	
YGL156W	[AMS1] Vacuolar alpha mannosidase, involved in free oligosaccharide (fOS) degradation; delivered to the vacuole in a novel pathway separate from the secretory pathway alpha mannosidase
YKL103C	[LAP4] Vacuolar aminopeptidase, often used as a marker protein in studies of autophagy and cytosol to vacuole targeting (CVT) pathway vacuolar aminopeptidase ysc1 Leucine aminopeptidase deficient
YOL082W	[ATG19] Protein involved in the cytoplasm-to-vacuole targeting pathway and in autophagy, recognizes cargo proteins and delivers them to the preautophagosomal structure for eventual engulfment by the autophagosome and degradation Receptor for biosynthetic cytoplasm to vacuole targeting Null: viable, unable to target vacuolar aminopeptidase I and to vacuoles, both under growing and nitrogen starvation conditions.

0335	GO_TERM:[plasma membrane] P-Value:5.0e-03
YDR508C	[GNP1] High-affinity glutamine permease, also transports Leu, Ser, Thr, Cys, Met and Asn; expression is fully dependent on Grr1p and modulated by the Ssy1p-Ptr3p-Ssy5p (SPS) sensor of extracellular amino acids high affinity glutamine permease Null mutant is viable but shows reduced glutamine transport and is therefore resistant to the glutamine analog L-glutamic acid gamma-monohydroxamate; overexpression induces sensitivity to heat shock
YLR373C	[VID22] Glycosylated integral membrane protein localized to the plasma membrane; plays a role in fructose-1,6-bisphosphatase (FBPase) degradation; involved in FBPase transport from the cytosol to Vid (vacuole import and degradation) vesicles Null mutant is viable but exhibits vacuole degradation of cytosolic proteins

0336	GO_TERM:[energy derivation by oxidation of organic compounds] P-Value:2.4e-02 OVERLAP:[other respiration chain complexes] <420.60> SIZE:14
**YKL085W**	[MDH1] Mitochondrial malate dehydrogenase, catalyzes interconversion of malate and oxaloacetate; involved in the tricarboxylic acid (TCA) cycle malate dehydrogenase
YIL053W	[RHR2] Constitutively expressed isoform of DL-glycerol-3-phosphatase; involved in glycerol biosynthesis, induced in response to both anaerobic and, along with the Hor2p/Gpp2p isoform, osmotic stress DL-glycerol-3-phosphatase Mutants lacking RHR2 show poor anaerobic growth. Mutants lacking RHR2 and HOR2 lack glycerol 3-phosphatase activity, produce only a small amount of glycerol. Overproduction of both genes does not significantly enhance glycerol production.
YJR035W	[RAD26] Protein involved in transcription-coupled repair nucleotide excision repair of UV-induced DNA lesions; homolog of human CSB protein DNA dependent ATPase|human Cockayne syndrome B gene ERCC6 homolog Null mutant is viable, defective in transcription-coupled repair, and hypermutable following exposure to UV light and shows delayed recovered of growth after UV exposure; rad7 rad26 and rad16 rad26 double mutants show enhanced sensitivity to UV light

0337	GO_TERM:[nucleotide-excision repair factor 3 complex] P-Value:1.2e-05 OVERLAP:[NEF3 complex] <510.180.10.30> SIZE:9
**YGR258C**	[RAD2] Single-stranded DNA endonuclease, cleaves single-stranded DNA during nucleotide excision repair to excise damaged DNA; subunit of Nucleotide Excision Repair Factor 3 (NEF3); homolog of human XPG protein xeroderma pigmentosum group G (XPG) protein homolog Null mutant is viable, radiation sensitive
**YIL143C**	[SSL2] Component of the holoenzyme form of RNA polymerase transcription factor TFIIH, has DNA-dependent ATPase/helicase activity and is required, with Rad3p, for unwinding promoter DNA; involved in DNA repair; homolog of human ERCC3 DNA helicase|human XPBC, ERCC3 homolog

0338	GO_TERM:[transcription factor TFIIH complex] P-Value:1.6e-27 OVERLAP:[TFIIH] <510.100> SIZE:9
YDR079C-A	[TFB5] Component of the RNA polymerase II general transcription and DNA repair factor TFIIH; involved in transcription initiation; homolog of the Chlamydomonas reinhardtii REX1-S protein which is involved in DNA repair transcription initiation/DNA repair factor TFIIH subunit
**YPL122C**	[TFB2] Subunit of TFIIH and nucleotide excision repair factor 3 complexes, involved in transcription initiation, required for nucleotide excision repair, similar to 52 kDa subunit of human TFIIH TFIIH subunit Null mutant is inviable; a c-terminal deletion mutant is associated with defects in nucleotide excision repair (as demonstrated by UV sensitivity
**YER171W**	[RAD3] 5' to 3' DNA helicase, involved in nucleotide excision repair and transcription; subunit of RNA polymerase II transcription initiation factor TFIIH; subunit of Nucleotide Excision Repair Factor 3 (NEF3); homolog of human XPD protein Null mutant is inviable; rad3 mutants are radiation sensitive|Deletion of this nucleotide excision repair (NER) gene results in lower levels of cross-link-induced recombination but higher mutation frequencies than wild-type cells.
**YDR311W**	[TFB1] Subunit of TFIIH and nucleotide excision repair factor 3 complexes, required for nucleotide excision repair, target for transcriptional activators transcription initiation factor IIb, 75 kDa subunit component
**YPR056W**	[TFB4] Subunit of TFIIH complex, involved in transcription initiation, similar to 34 kDa subunit of human TFIIH; interacts with Ssl1p transcription initiation factor TFIIH subunit
**YDL108W**	[KIN28] Serine/threonine protein kinase, subunit of the transcription factor TFIIH; involved in transcription initiation at RNA polymerase II promoters
**YLR005W**	[SSL1] Component of the core form of RNA polymerase transcription factor TFIIH, which has both protein kinase and DNA-dependent ATPase/helicase activities and is essential for transcription and nucleotide excision repair; interacts with Tfb4p RNA polymerase transcription factor TFIIH component Null mutant is inviable; temperature-sensitive mutants are UV-sensitive and deficient in nucleotide excision repair.
**YDR460W**	[TFB3] Subunit of TFIIH and nucleotide excision repair factor 3 complexes, involved in transcription initiation, required for nucleotide excision repair; ring finger protein similar to mammalian CAK and TFIIH subunit TFIIH subunit
**YPR025C**	[CCL1] Cyclin associated with protein kinase Kin28p, which is the TFIIH-associated carboxy-terminal domain (CTD) kinase involved in transcription initiation at RNA polymerase II promoters TFIIK subunit, a subcomplex of transcription factor TFIIH|cyclin

0339	GO_TERM:[telomere cap complex] P-Value:2.2e-06
YBR275C	[RIF1] Protein that binds to the Rap1p C-terminus and acts synergistically with Rif2p to help control telomere length and establish telomeric silencing; deletion results in telomere elongation RAP1-interacting factor defective in telomeric silencing and telomere length regulation
YNL216W	[RAP1] DNA-binding protein involved in either activation or repression of transcription, depending on binding site context; also binds telomere sequences and plays a role in telomeric position effect (silencing) and telomere structure repressor activator protein null is inviable; some mutations abolish silencing (at telomeres and at the silent mating-type loci), other mutations or overproduction alter telomere length
YPL098C	[MGR2] Protein required for growth of cells lacking the mitochondrial genome
YNR071C	
YLR453C	[RIF2] Protein that binds to the Rap1p C-terminus and acts synergistically with Rif1p to help control telomere length and establish telomeric silencing; deletion results in telomere elongation nuclear protein
YML038C	[YMD8] Putative nucleotide sugar transporter, has similarity to Vrg4p
YNR066C	

0340	GO_TERM:[transcription factor TFIIIB complex] P-Value:2.3e-06 OVERLAP:[TFIIIB] <510.140> SIZE:3
**YGR246C**	[BRF1] TFIIIB B-related factor, one of three subunits of RNA polymerase III transcription initiation factor TFIIIB, binds TFIIIC and TBP and recruits RNA pol III to promoters, amino-terminal half is homologous to TFIIB RNA polymerase III transcription factor|similar to TFIIB
**YNL039W**	[BDP1] Essential subunit of RNA polymerase III transcription factor (TFIIIB), which is involved in transcription of genes encoding tRNAs, 5S rRNA, U6 snRNA, and other small RNAs TFIIIB 90 kDa subunit Null mutant is inviable; tfc5 mutant suppresses mutations in the class III transcription system

0341	GO_TERM:[transcription factor TFIIIC complex] P-Value:7.5e-16 OVERLAP:[TFIIIC] <510.150> SIZE:5
**YAL001C**	[TFC3] Largest of six subunits of the RNA polymerase III transcription initiation factor complex (TFIIIC); part of the TauB domain of TFIIIC that binds DNA at the BoxB promoter sites of tRNA and similar genes; cooperates with Tfc6p in DNA binding transcription factor tau (TFIIIC) subunit
**YBR123C**	[TFC1] One of six subunits of the RNA polymerase III transcription initiation factor complex (TFIIIC); part of the TauA globular domain of TFIIIC that binds DNA at the BoxA promoter sites of tRNA and similar genes; human homolog is TFIIIC-63 95 kDa|transcription factor tau (TFIIIC) subunit lethal

**YDR362C**	[TFC6] One of six subunits of RNA polymerase III transcription initiation factor complex (TFIIIC); part of TFIIIC TauB domain that binds BoxB promoter sites of tRNA and other genes; cooperates with Tfc3p in DNA binding; human homolog is TFIIIC-110 91 kDa tau91 subunit of transcription factor IIIC (TFIIIC)
**YOR110W**	[TFC7] One of six subunits of the RNA polymerase III transcription initiation factor complex (TFIIIC); part of the TauA globular domain of TFIIIC that binds DNA at the BoxA promoter sites of tRNA and similar genes TFIIIC (tau55) 55 kDa subunit
YPL007C	[TFC8] One of six subunits of RNA polymerase III transcription initiation factor complex (TFIIIC); part of TFIIIC TauB domain that binds BoxB promoter sites of tRNA and other genes; linker between TauB and TauA domains; human homolog is TFIIIC-90 TFIIIC (tau60) 60 kDa subunit

0342	GO_TERM:[mRNA cleavage factor complex] P-Value:6.3e-46 OVERLAP:[pre mRNA3'-end processing factor CFII] <440.10.20> SIZE:4
YHR029C	[YHI9] Protein of unknown function that is a member of the PhzF superfamily, although unlike its bacterial homolog, is most likely not involved in phenazine production; possibly involved in a membrane regulation metabolic pathway
YPL138C	[SPP1] Subunit of COMPASS (Set1C), a complex which methylates histone H3 on lysine 4 and is required in telomeric transcriptional silencing; PHD finger domain protein similar to human CGBP, an unmethylated CpG binding protein compass (complex proteins associated with Set1p) component Null: defective in silencing of expression of genes located near telomeres; hydroxyurea sensitive.
YBR258C	[SHG1] Subunit of the COMPASS (Set1C) complex, which methylates histone H3 on lysine 4 and is required in transcriptional silencing near telomeres compass (complex proteins associated with Set1p) component
YHR119W	[SET1] Histone methyltransferase, subunit of the COMPASS (Set1C) complex which methylates histone H3 on lysine 4; required in transcriptional silencing near telomeres and at the silent mating type loci; contains a SET domain Null mutant is viable, exhibits derepression of silenced genes at telomeres and the HML silent mating-type locus
YBR175W	[SWD3] Essential subunit of the COMPASS (Set1C) complex, which methylates histone H3 on lysine 4 and is required in transcriptional silencing near telomeres; WD40 beta propeller superfamily member and ortholog of mammalian WDR5 compass (complex proteins associated with Set1p) component Null: defective in silencing of expression of genes located near telomeres; hydroxyurea sensitive.
YLR015W	[BRE2] Subunit of the COMPASS (Set1C) complex, which methylates histone H3 on lysine 4 and is required in transcriptional silencing near telomeres; involved in telomere maintenance; similar to trithorax-group protein ASH2L compass (complex proteins associated with Set1p) component Null: null mutant is sensitive to brefeldin A
YAR003W	[SWD1] Subunit of the COMPASS (Set1C) complex, which methylates histone H3 on lysine 4 and is required in transcriptional silencing near telomeres; WD40 beta propeller superfamily member with similarity to mammalian Rbbp7 compass (complex proteins associated with Set1p) component Null: defective in silencing of expression of genes located near telomeres; hydroxyurea sensitive.
YDR469W	[SDC1] Subunit of the COMPASS (Set1C) complex, which methylates histone H3 on lysine 4 and is required in transcriptional silencing near telomeres; has similarity to C. elegans Dpy-30 compass (complex proteins associated with Set1p) component Null: defective in silencing of expression of genes located near telomeres; hydroxyurea sensitive.
YHL033C	[RPL8A] Ribosomal protein L4 of the large (60S) ribosomal subunit, nearly identical to Rpl8Bp and has similarity to rat L7a ribosomal protein; mutation results in decreased amounts of free 60S subunits ribosomal protein L8A (rp6) (YL5) (L4A) Null mutant is viable, rpl4A rpl4B double mutant is inviable; deficient in maintenance of killer
YLR448W	[RPL6B] Protein component of the large (60S) ribosomal subunit, has similarity to Rpl6Bp and to rat L6 ribosomal protein; binds to 5.8S rRNA ribosomal protein L6B (L17B) (rp18) (YL16) Null mutant is viable, grows slower than wild-type. rpl6a rpl6b double mutants are inviable; rpl6 mutants are deficient in 60S ribosomal subunits relative to 40S subunits; 43S preinitiation complexes accumulate in half-mer polyribosomes in the absence of sufficient 60S subunits.
YOR250C	[CLP1] Subunit of cleavage factor I (CFI), involved in both the endonucleoiytc cleavage and polyadenylation steps of mRNA 3'-end maturation cleavage and polyadenylation factor CF I component involved in pre-mRNA 3'-end processing
YGR047C	[TFC4] One of six subunits of the RNA polymerase III transcription initiation factor complex (TFIIIC); part of the TauA domain of TFIIIC that binds BoxA DNA promoter sites of tRNA and similar genes; has TPR motifs; human homolog is TFIIIC-102 131 kDa|transcription factor tau (TFIIIC) subunit
YGL044C	[RNA15] Cleavage and polyadenylation factor I (CF I) component involved in cleavage and polyadenylation of mRNA 3' ends; interacts with the A-rich polyadenylation signal in complex with Rna14p and Hrp1p cleavage and polyadenylation factor CF I component involved in pre-mRNA 3'-end processing
YOR179C	[SYC1] Subunit of the APT subcomplex of cleavage and polyadenylation factor, may have a role in 3' end formation of both polyadenylated and non-polyadenylated RNAs
YPL008W	[CHL1] Conserved nuclear protein required to establish sister-chromatid pairing during S-phase, probable DNA helicase with similarity to human BACH1, which associates with tumor suppressor BRCA1; associates with acetyltransferase Ctf7p deah box protein|kinetochore protein Null mutant is viable, ts mutants mis-segregate chromosomes at permissive temperature leading to increased rate of mitotic chromosome loss, at non-permissive temperature ts mutants transiently arrest as large-budded cells with G(sub)2 DNA content and short spindle
YDR228C	[PCF11] mRNA 3' end processing factor, essential component of cleavage and polyadenylation factor IA (CF IA), involved in pre-mRNA 3' end processing and in transcription termination; binds C-terminal domain of largest subunit of RNA pol II (Rpo21p) cleavage and polyadenylation factor CF I component involved in pre-mRNA 3'-end processing Null mutant is inviable; pcf11 (ts) mutations are synthetically lethal with rna14 (ts) and rna15 (ts) mutations

YER133W	[GLC7] Catalytic subunit of type 1 serine/threonine protein phosphatase, involved in many processes including glycogen metabolism, sporulation, and mitosis; interacts with multiple regulatory subunits; predominantly isolated with Sds22p protein phosphatase type I
YMR061W	[RNA14] Cleavage and polyadenylation factor I (CF I) component involved in cleavage and polyadenylation of mRNA 3' ends; bridges interaction between Rna15p and Hrp1p in the CF I complex cleavage and polyadenylation factor CF I component involved in pre-mRNA 3'-end processing
**YDR301W**	[CFT1] RNA-binding subunit of the mRNA cleavage and polyadenylation factor; involved in poly(A) site recognition and required for both pre-mRNA cleavage and polyadenylation, 51% sequence similarity with mammalian AAUAA-binding subunit of CPSF 150 kDa protein associated with polyadenylation factor 1 (PF I)|cleavage factor II (CF II) component
YKL018W	[SWD2] Subunit of the COMPASS (Set1C) complex, which methylates histone H3 on lys 4 and is involved in telomeric silencing; subunit of CPF (cleavage and polyadenylation factor), a complex involved in RNAP II transcription termination compass (complex proteins associated with Set1p) component
YNL222W	[SSU72] Transcription/RNA-processing factor that associates with TFIIB and cleavage/polyadenylation factor Pta1p; exhibits phosphatase activity on serine-5 of the RNA polymerase II C-terminal domain; affects start site selection in vivo
**YAL043C**	[PTA1] Subunit of holo-CPF, a multiprotein complex and functional homolog of mammalian CPSF, required for the cleavage and polyadenylation of mRNA and snoRNA 3' ends; involved in pre-tRNA processing; binds to the phosphorylated CTD of RNAPII cleavage factor II (CF II) component|polyadenylation factor I (PF I) Null mutant is inviable; temperature-sensitive mutant shows defects in pre-tRNA processing
YDR195W	[REF2] RNA-binding protein involved in the cleavage step of mRNA 3'-end formation prior to polyadenylation, and in snoRNA maturation; part of holo-CPF subcomplex APT, which associates with 3'-ends of snoRNA- and mRNA-encoding genes RNA-binding protein
**YLR115W**	[CFT2] Subunit of the mRNA cleavage and polyadenlylation factor (CPF); required for pre-mRNA cleavage, polyadenylation and poly(A) site recognition, 43% similarity with the mammalian CPSF-100 protein. 105 kDa protein associated with polyadenylation factor 1 (PF I)|cleavage factor II (CF II) component
YPR107C	[YTH1] Essential RNA-binding component of cleavage and polyadenylation factor, contains five zinc fingers; required for pre-mRNA 3'-end processing and polyadenylation polyadenylation factor subunit null is inviable; other mutations result in polyadenylation deficiency
YJR093C	[FIP1] Subunit of cleavage polyadenylation factor (CPF), interacts directly with poly(A) polymerase (Pap1p) to regulate its activity polyadenylation factor I (PF I) Null mutant is inviable. At restrictive temperature, a temperature-sensitive mutant shows shortening of poly(A) tails
YNL317W	[PFS2] Integral subunit of the pre-mRNA cleavage and polyadenylation factor (CPF) complex; plays an essential role in mRNA 3'-end formation by bridging different processing factors and thereby promoting the assembly of the processing complex polyadenylation factor I (PF I) Null mutant is inviable; conditionally lethal mutations exhibit defects in 3'-end processing in vitro
YGR156W	[PTI1] Pta1p Interacting protein
**YLR277C**	[YSH1] Putative endonuclease, subunit of the mRNA cleavage and polyadenylation specificity complex required for 3' processing of mRNAs cleavage factor II (CF II) component|polyadenylation factor I (PF I)
YKL059C	[MPE1] Essential conserved subunit of CPF (cleavage and polyadenylation factor), plays a role in 3' end formation of mRNA via the specific cleavage and polyadenylation of pre-mRNA, contains a putative RNA-binding zinc knuckle motif
YKR002W	[PAP1] Poly(A) polymerase, one of three factors required for mRNA 3'-end polyadenylation, forms multiprotein complex with polyadenylation factor I (PF I), also required for mRNA nuclear export; may also polyadenylate rRNAs poly(A) polymerase lethal

0343	GO_TERM:[nucleotide-excision repair, DNA damage recognition] P-Value:2.2e-15 OVERLAP:[NEF4 complex] <510.180.10.40> SIZE:2
YER162C	[RAD4] Protein that recognizes and binds damaged DNA (with Rad23p) during nucleotide excision repair; subunit of Nuclear Excision Repair Factor 2 (NEF2); homolog of human XPC protein Null mutant is viable and radiation sensitive|Deletion of this nucleotide excision repair (NER) gene results in lower levels of cross-link-induced recombination but higher mutation frequencies than wild-type cells.
YMR201C	[RAD14] Protein that recognizes and binds damaged DNA during nucleotide excision repair; subunit of Nucleotide Excision Repair Factor 1 (NEF1); contains zinc finger motif; homolog of human XPA protein human xeroderma pigmentosum group A DNA repair gene homolog Null mutant is viable and radiation sensitive
YPL046C	[ELC1] Elongin C, forms heterodimer with Ela1p that participates in transcription elongation; expression dramatically upregulated during sporulation; widely conserved among eukaryotes elongin C transcription elongation factor The deletion mutant is not sensitive to UV damage, however the elc1 rad23 double mutant is more UV sensitive than the rad23 mutant alone.
**YBR114W**	[RAD16] Protein that recognizes and binds damaged DNA in an ATP-dependent manner (with Rad7p) during nucleotide excision repair; subunit of Nucleotide Excision Repair Factor 4 (NEF4); member of the SWI/SNF family radiation sensitive
**YJR052W**	[RAD7] Protein that recognizes and binds damaged DNA in an ATP-dependent manner (with Rad16p) during nucleotide excision repair; subunit of Nucleotide Excision Repair Factor 4 (NEF4) nucleotide excision NEF4 component radiation sensitive

0344	GO_TERM:[transcription regulator activity] P-Value:1.4e-06 OVERLAP:[RTG complex] <510.190.130> SIZE:2
YDR123C	[INO2] Component of the heteromeric Ino2p/Ino4p basic helix-loop-helix transcription activator that binds inositol/choline-responsive elements (ICREs), required for derepression of phospholipid biosynthetic genes in response to inositol depletion helix-loop-helix protein The null mutant is viable but auxotrophic for inositol and choline. The null mutant can also display aberant cell shape and defects in nuclear segregation. Homozygous mutant ino2 delta-1 diploids fail to sporulate. Other mutant alleles show pleiotropic defects in phospholipid metabolism.
YHL020C	[OPI1] Transcriptional regulator of a variety of genes; phosphorylation by protein kinase A stimulates Opi1p function in negative regulation of phospholipid biosynthetic genes; involved in telomere maintenance The null mutant is viable but constitutively accumulates INO1 mRNA.
YOL108C	[INO4] Transcription factor required for derepression of inositol-choline-regulated genes involved in phospholipid synthesis; forms a complex, with Ino2p, that binds the inositol-choline-responsive element through a basic helix-loop-helix domain basic helix-loop-helix (bHLH) protein The null mutant is viable but auxotrophic for inositol and choline. The null mutant expresses repressed levels of inositol-1-phosphate synthase (INO1) mRNA and exhibits reduced phosphatidylcholine biosynthesis.
**YBL103C**	[RTG3] Basic helix-loop-helix-leucine zipper (bHLH/Zip) transcription factor that forms a complex with another bHLH/Zip protein, Rtg1p, to activate the retrograde (RTG) and TOR pathways phosphoprotein
**YOL067C**	[RTG1] Transcription factor (bHLH) involved in interorganelle communication between mitochondria, peroxisomes, and nucleus transcription factor Null mutant is viable but cannot grow on acetate as the sole carbon source, is a glutamate and aspartate auxotroph, and shows decreased citrate synthase, acetyl-CoA synthetase, NAD isocitrate dehydrogenase, and pyruvate carboxylase activities

0345	GO_TERM:[transcription regulator activity] P-Value:3.8e-02
YDR259C	[YAP6] Putative basic leucine zipper (bZIP) transcription factor; overexpression increases sodium and lithium tolerance
YGL181W	[GTS1] Protein containing a zinc-finger in the N-terminus and a long Gln-rich region in the C-terminus; regulates ultradian rhythm, cell size, cell cycle, lifespan, sporulation, heat tolerance, and multidrug transport Null mutant is viable; shows reduced lag phase
YDR034C	[LYS14] Transcriptional activator involved in regulation of genes of the lysine biosynthesis pathway; requires 2-aminoadipate semialdehyde as co-inducer Lysine requiring
YDL138W	[RGT2] Plasma membrane glucose receptor, highly similar to Snf3p; both Rgt2p and Snf3p serve as transmembrane glucose sensors generating an intracellular signal that induces expression of glucose transporter (HXT) genes glucose receptor Dominant mutant suppresses growth defect of snf3 mutants on low concentrations of glucose or fructose
YDR277C	[MTH1] Negative regulator of the glucose-sensing signal transduction pathway, required for repression of transcription by Rgt1p; interacts with Rgt1p and the Snf3p and Rgt2p glucose sensors; phosphorylated by Yck1p, triggering Mth1p degradation Msn3p homolog (61% identical) Null mutant is viable; mth1(htr1) mutants are deficient in glucose update and transcription of glucose transporters; mth1 (htr1) mutation suppresses glucose sensitivity of tpi1 mutant; multicopy expression of HXT genes suppresses some defects of mth1 (htr1) mutants; msn3 mth1 double deletion mutants are impaired in derepression of invertase in response to glucose limitation

0346	GO_TERM:[CCAAT-binding factor complex] P-Value:1.3e-08 OVERLAP:[CCAAT-binding factor complex] <510.160> SIZE:4
YDR022C	[CIS1] Protein of unknown function that may be involved in microtubule organization; high-copy suppressor of CIK1 deletion
YOR047C	[STD1] Protein involved in control of glucose-regulated gene expression; interacts with protein kinase Snf1p, glucose sensors Snf3p and Rgt2p, and TATA-binding protein Spt15p; acts as a regulator of the transcription factor Rgt1p MTH1 homolog Null mutant is viable, no defects in mating or sporulation. Suppressor of TBP deletion; multicopy suppressor of SNF; std1-mth1 has defective glucose derepression and sporulation
**YGL237C**	[HAP2] Subunit of the heme-activated, glucose-repressed Hap2p/3p/4p/5p CCAAT-binding complex, a transcriptional activator and global regulator of respiratory gene expression; contains sequences sufficient for both complex assembly and DNA binding transcriptional activator protein of CYC1 (component of HAP2/HAP3 heteromer)
**YBL021C**	[HAP3] Subunit of the heme-activated, glucose-repressed Hap2p/3p/4p/5p CCAAT-binding complex, a transcriptional activator and global regulator of respiratory gene expression; contains sequences contributing to both complex assembly and DNA binding transcriptional activator protein of CYC1 (component of HAP2/HAP3 heteromer)
**YOR358W**	[HAP5] Subunit of the heme-activated, glucose-repressed Hap2/3/4/5 CCAAT-binding complex, a transcriptional activator and global regulator of respiratory gene expression; required for assembly and DNA binding activity of the complex CCAAT-binding transcription factor component (along with Hap2p and Hap3p)

0347	GO_TERM:[autophagy] P-Value:1.1e-05

YFR019W	[FAB1] 1-phosphatidylinositol-3-phosphate 5-kinase; vacuolar membrane kinase that generates phosphatidylinositol (3,5)P2, which is involved in vacuolar sorting and homeostasis 1-phosphatidylinositol-3-phosphate 5-kinase Null mutant is temperature-sensitive. Mutation causes pleiotropic effects on nuclear migration and orientation, and separation of mitotic chromosomes (forms aploid and binucleate cells); has defects in vacuolar function and morphology.
YFR021W	[ATG18] Phosphatidylinositol 3,5-bisphosphate-binding protein of the vacuolar membrane, predicted to fold as a seven-bladed beta-propeller; required for recycling of Atg9p through the pre-autophagosomal structure (NMR1)Null mutant is viable; arrests with 2C DNA content after shift to sporulation medium.
YDL149W	[ATG9] Transmembrane protein involved in formation of Cvt and autophagic vesicles; cycles between the pre-autophagosomal structure and other cytosolic punctate structures, not found in autophagosomes integral membrane protein Null mutant is viable but blocked in autophagy and aminopeptidase I import into vacuole. Temperature-sensitive mutant accumulates membrane-associated, protease-sensitive API.
YNL242W	[ATG2] Peripheral membrane protein required for the formation of cytosolic sequestering vesicles involved in vacuolar import through both the Cvt pathway and autophagy; interacts with Atg9p and is necessary for its trafficking peripheral membrane protein The null mutant is viable but blocked in autophagy, pexophagy and import of Ape1 by the cytoplasm to vacuole targeting pathway. Diploids homozygous for the null mutation lack premeitoic DNA synthesis and do not sporulate.

0348	GO_TERM:[mitochondrial fission] P-Value:9.8e-14 OVERLAP:[CCR4 complex] <510.190.110> SIZE:13
**YKR036C**	[CAF4] WD40 repeat-containing protein associated with the CCR4-NOT complex, interacts in a Ccr4p-dependent manner with Ssn2p CCR4 transcriptional complex component
YLL001W	[DNM1] Dynamin-related GTPase required for mitochondrial fission and the maintenance of mitochondrial morphology, assembles on the cytoplasmic face of mitochondrial tubules at sites at which division will occur; also participates in endocytosis similar to dynamin GTPase Null mutant is viable, shows mating defects consistent with a delay in receptor-mediated endocytosis
YIL065C	[FIS1] Mitochondrial outer membrane protein involved in membrane fission, required for localization of Dnm1p and Mdv1p during mitochondrial division Null mutant is viable, mitochondrial fission blocked, mitochondrial membranes form nets
YJL112W	[MDV1] Peripheral protein of the cytosolic face of the mitochondrial outer membrane, required for mitochondrial fission; interacts with Fis1p and with the dynamin-related GTPase Dnm1p; contains WD repeats Null mutant is viable, mitochondrial fission blocked, mitochondrial membranes form nets

0349	GO_TERM:[nascent polypeptide-associated complex] P-Value:2.8e-06 OVERLAP:[NAC complex] <510.190.30> SIZE:2
**YHR193C**	[EGD2] Alpha subunit of the heteromeric nascent polypeptide-associated complex (NAC) involved in protein sorting and translocation, associated with cytoplasmic ribosomes GAL4 enhancer protein|nascent-polypeptide-associated complex human alpha NAC subunit homolog
YDR252W	[BTT1] Beta3 subunit of the heterotrimeric nascent polypeptide-associated complex (alpha, beta1, beta3) which binds ribosomes via its beta-subunits in close proximity to nascent polypeptides
YJR011C	

0350	GO_TERM:[CCR4-NOT complex] P-Value:7.8e-24 OVERLAP:[CCR4 complex] <510.190.110> SIZE:13
YBR082C	[UBC4] Ubiquitin-conjugating enzyme that mediates degradation of short-lived and abnormal proteins; interacts with E3-CaM in ubiquitinating calmodulin; interacts with many SCF ubiquitin protein ligases; component of the cellular stress response ubiquitin conjugating enzyme e2 Overexpression confers resistance to methylmercury. The ubc4ubc5 double mutant is temperature sensitive, reduces turnover of short-lived proteins and canavanyl-peptides but not of long-lived proteins.
YDR059C	[UBC5] Ubiquitin-conjugating enzyme that mediates selective degradation of short-lived and abnormal proteins, central component of the cellular stress response; expression is heat inducible ubiquitin-conjugating enzyme viable, ubc4/ubc5 double mutant is temperature sensitive
YGR134W	[CAF130] Part of the evolutionarily-conserved CCR4-NOT transcriptional regulatory complex involved in controlling mRNA initiation, elongation, and degradation
YPL037C	[EGD1] Subunit beta1 of the nascent polypeptide-associated complex (NAC) involved in protein targeting, associated with cytoplasmic ribosomes; enhances DNA binding of the Gal4p activator; homolog of human BTF3b pol II transcribed genes regulator
**YPR072W**	[NOT5] Subunit of the CCR4-NOT complex, which is a global transcriptional regulator with roles in transcription initiation and elongation and in mRNA degradation NOT complex member, a global negative regulator of transcription Null mutant is viable, mutations in not4(mot2) are synthetically lethal with mutations in not5, overexpression of NOT3 or NOT4(MOT2) suppresses not5 mutations
**YIL038C**	[NOT3] Subunit of the CCR4-NOT complex, which is a global transcriptional regulator with roles in transcription initiation and elongation and in mRNA degradation CCR4 transcriptional complex component Null mutant is viable, overexpression of NOT3 suppresses cdc39(not1) and cdc36(not2) mutations

**YNR052C**	[POP2] RNase of the DEDD superfamily, subunit of the Ccr4-Not complex that mediates 3' to 5' mRNA deadenylation transcription factor (putative) Mutant is resistant to glucose derepression, temperature-sensitive, and unable to sporulate and contains reduced amounts of reserve carbohydrates
**YER068W**	[MOT2] Component of the CCR4-NOT transcription regulatory complex, which represses transcription, at least in part, by inhibiting functional TBP-DNA interactions and also aids in transcription elongation; interacts with C-terminal region of Not1p zinc finger protein (putative) Null mutant is viable, exhibits a modest increase in basal transcription of several pheromone-responsive genes; exhibits a conditional cell lysis phenotype
**YCR093W**	[CDC39] Component of the CCR4-NOT complex, which has multiple roles in regulating mRNA levels including regulation of transcription and destabilizing mRNAs by deadenylation; basal transcription factor transcriptional regulator Null mutant is inviable; arrests in G(sub)1 at pachytene at the mononucleate stage with duplicated, unseparated spindle pole bodies and no spindles; temperature sensitive mutation which causes increased basal transcription of many genes
**YAL021C**	[CCR4] Component of the CCR4-NOT transcriptional complex, which is involved in regulation of gene expression; component of the major cytoplasmic deadenylase, which is involved in mRNA poly(A) tail shortening reduced levels of ADH2 expression under both glucose and ethanol growth conditions; temperature sensitive growth on nonfermentative medium
**YDL165W**	[CDC36] Component of the CCR4-NOT complex, which has multiple roles in regulating mRNA levels including regulation of transcription and destabilizing mRNAs by deadenylation; basal transcription factor transcriptional regulator Null mutant is viable, cdc36 mutant arrests in G(sub)1; forms shmoo morphology at restrictive temperature, arrests at pachytene at the mononucleate stage with duplicated spindle pole bodies and no spindles
YNL288W	[CAF40] Evolutionarily conserved subunit of the CCR4-NOT complex involved in controlling mRNA initiation, elongation and degradation; binds Cdc39p

0351	GO_TERM:[catalytic activity] P-Value:7.3e-02
YLL019C	[KNS1] Nonessential putative protein kinase of unknown cellular role; member of the LAMMER family of protein kinases, which are serine/threonine kinases also capable of phosphorylating tyrosine residues protein kinase homolog
YLR044C	[PDC1] Major of three pyruvate decarboxylase isozymes, key enzyme in alcoholic fermentation, decarboxylates pyruvate to acetaldehyde; subject to glucose-, ethanol-, and autoregulation; involved in amino acid catabolism pyruvate decarboxylase undetectable pyruvate decarboxylase activity in pdc1pdc5 double mutants

0352	GO_TERM:[RNA polymerase I upstream activating factor complex] P-Value:1.6e-09 OVERLAP:[Upstream Activation Factor (UAF) complex] <510.30> SIZE:3
**YLR141W**	[RRN5] Protein involved in transcription of rDNA by RNA polymerase I; transcription factor, member of UAF (upstream activation factor) family along with Rrn9p and Rrn10p UAF member (upstream activation factor) along with Rrn9p and Rrn10p|transcription factor
**YBL025W**	[RRN10] Protein involved in promoting high level transcription of rDNA, subunit of UAF (upstream activation factor) for RNA polymerase I upstream activation factor subunit Mutant shows reduction in the transcription of rDNA
**YMR270C**	[RRN9] Protein involved in promoting high level transcription of rDNA, subunit of UAF (upstream activation factor) for RNA polymerase I upstream activation factor subunit

0353	GO_TERM:[generation of precursor metabolites and energy] P-Value:1.9e-01 OVERLAP:[F0/F1 ATP synthase (complex V)] <420.50> SIZE:18
YMR039C	[SUB1] Transcriptional coactivator, facilitates elongation by influencing enzymes that modify RNAP II, acts in a peroxide resistance pathway involving Rad2p; suppressor of TFIIB mutations transcriptional coactivator Null mutant is viable, auxotrophic for inositol; high copy suppressor of SUA7 (TFIIB) mutations. Overexpression of SUB1 stimulates transcription by some types of activators in vivo
YKL060C	[FBA1] Fructose 1,6-bisphosphate aldolase, a cytosolic enzyme required for glycolysis and gluconeogenesis; catalyzes the conversion of fructose 1,6 bisphosphate into two 3-carbon products: glyceraldehyde-3-phosphate and dihydroxyacetone phosphate aldolase Null mutant is viable, lacks aldolase enzymatic activity and fails to grow in media containing as a carbon source metabolites of only one side of the aldolase reaction
YGL250W	
YDL081C	[RPP1A] Ribosomal protein P1 alpha, a component of the ribosomal stalk, which is involved in the interaction between translational elongation factors and the ribosome; accumulation of P1 in the cytoplasm is regulated by phosphorylation and interaction with the P2 stalk component acidic ribosomal protein P1A (YP1alpha) (A1)
YIL005W	[EPS1] Pdi1p (protein disulfide isomerase)-related protein involved in endoplasmic reticulum retention of resident ER proteins
**YLR295C**	[ATP14] Subunit h of the F0 sector of mitochondrial F1F0 ATP synthase, which is a large, evolutionarily conserved enzyme complex required for ATP synthesis ATP synthase subunit h unable to grow on glycerol medium; no detectable oligomycin-sensitive ATPase activity

0354	GO_TERM:[molecular_function] P-Value:3.7e-01

YJL102W	[MEF2] Mitochondrial elongation factor involved in translational elongation mitochondrial elongation factor G-like protein
YJR154W	
YLL022C	[HIF1] Non-essential component of the HAT-B histone acetyltransferase complex (Hat1p-Hat2p-Hif1p), localized to the nucleus; has a role in telomeric silencing Null mutant is viable and does not show any obvious phenotypes
0355	GO_TERM:[chromatin silencing] P-Value:1.8e-18 OVERLAP:[Replication initiation complex] <410.20> SIZE:8
YDR310C	[SUM1] Transcriptional repressor required for mitotic repression of middle sporulation-specific genes; involved in telomere maintenance, regulated by the pachytene checkpoint Restores silencing at HML and HMR in presence of sir2, sir3 and sir4 mutants
YOR279C	[RFM1] DNA-binding protein required for vegetative repression of middle sporulation genes; specificity factor that directs the Hst1p histone deacetylase to some of the promoters regulated by Sum1p; involved in telomere maintenance DNA-binding protein Null mutant is viable, derepression of middle meiosis-specific genes; required for SUM1-1 mediated suppression of sir2 mutants
YKR101W	[SIR1] Protein involved in repression of transcription at the silent mating-type loci HML and HMR; recruitment to silent chromatin requires interactions with Orc1p and with Sir4p, through a common Sir1p domain; binds to centromeric chromatin silent mating loci repressor
YJL194W	[CDC6] Essential ATP-binding protein required for DNA replication, component of the pre-replicative complex (pre-RC) which requires ORC to associate with chromatin and is in turn required for Mcm2-7p DNA association; homologous to S. pombe Cdc18p pre-initiation complex component arrest at initiation of S phase
**YDR052C**	[DBF4] Regulatory subunit of Cdc7p-Dbf4p kinase complex, required for Cdc7p kinase activity and initiation of DNA replication; phosphorylates the Mcm2-7 family of proteins; cell cycle regulated Cdc7p-Dbf4p kinase complex regulatory subunit Null mutant is inviable; conditional alleles cause cell cycle arrest at the G1/S transition; dumbbell shape
YPL001W	[HAT1] Catalytic subunit of the Hat1p-Hat2p histone acetyltransferase complex that uses the cofactor acetyl coenzyme A, to acetylate free nuclear and cytoplasmic histone H4; involved in telomeric silencing and DNA double-strand break repair histone acetyltransferase
YEL056W	[HAT2] Subunit of the Hat1p-Hat2p histone acetyltransferase complex; required for high affinity binding of the complex to free histone H4, thereby enhancing Hat1p activity; similar to human RbAp46 and 48; has a role in telomeric silencing histone acetyltransferase subunit
**YLL004W**	[ORC3] Subunit of the origin recognition complex, which directs DNA replication by binding to replication origins and is also involved in transcriptional silencing origin recognition complex subunit
**YBR060C**	[ORC2] Subunit of the origin recognition complex, which directs DNA replication by binding to replication origins and is also involved in transcriptional silencing; may be phosphorylated by Cdc28p origin recognition complex subunit 2
**YNL261W**	[ORC5] Subunit of the origin recognition complex, which directs DNA replication by binding to replication origins and is also involved in transcriptional silencing ATP-binding site (putative)|origin recognition complex fifth largest subunit orc5-1 mutant is temperature-sensitive, has defects in transcriptional silencing, has elevated rate of plasmid loss and inefficient initiation of DNA replication at the permissive temperature, and arrests at the nonpermissive temperature; CDC6 is multicopy suppressor of orc5-1
**YML065W**	[ORC1] Largest subunit of the origin recognition complex, which directs DNA replication by binding to replication origins and is also involved in transcriptional silencing; may be phosphorylated by Cdc28p origin recognition complex (ORC) 120 kDa (largest) subunit|similar to Cdc6p, Cdc18p, and Sir3p and to proteins from K. lactis, S. pombe, and humans
**YHR118C**	[ORC6] Subunit of the origin recognition complex, which directs DNA replication by binding to replication origins and is also involved in transcriptional silencing; may be phosphorylated by Cdc28p ORC 50 kDa subunit
**YPR162C**	[ORC4] Subunit of the origin recognition complex, which directs DNA replication by binding to replication origins and is also involved in transcriptional silencing origin recognition complex (ORC) 56 kDa subunit
0356	GO_TERM:[homocitrate synthase activity] P-Value:1.9e-07
YDL131W	[LYS21] Homocitrate synthase isozyme, catalyzes the condensation of acetyl-CoA and alpha-ketoglutarate to form homocitrate, which is the first step in the lysine biosynthesis pathway; highly similar to the other isozyme, Lys20p YDL182W (LYS20) homolog|homocitrate synthase
YDL182W	[LYS20] Homocitrate synthase isozyme, catalyzes the condensation of acetyl-CoA and alpha-ketoglutarate to form homocitrate, which is the first step in the lysine biosynthesis pathway; highly similar to the other isozyme, Lys21p YDL131W (LYS21) homolog|homocitrate synthase Null mutant is viable, is able to grow on minimal media, and exhibits reduced but significant homocitrate synthase activity
0357	GO_TERM:[response to osmotic stress] P-Value:5.1e-07
YLR113W	[HOG1] Mitogen-activated protein kinase involved in osmoregulation via three independent osmosensors; mediates the recruitment and activation of RNA Pol II at Hot1p-dependent promoters; localization regulated by Ptp2p and Ptp3p MAP kinase Null mutant is viable and unable to grow in high osmolarity media
YDR162C	[NBP2] Protein involved in the HOG (high osmolarity glycerol) pathway, negatively regulates Hog1p by recruitment of phosphatase Ptc1p the Pbs2p-Hog1p complex, found in the nucleus and cytoplasm, contains an SH3 domain that binds Pbs2p

YDL006W	[PTC1] Type 2C protein phosphatase (PP2C); inactivates the osmosensing MAPK cascade by dephosphorylating Hog1p; mutation delays mitochondrial inheritance; deletion reveals defects in precursor tRNA splicing, sporulation and cell separation Null mutant is viable; exhibits synthetic phenotypes in combination with ptp2, kcs1, and mpk1 (slt2) mutants; ptc1 mutations suppress the hyper-recombination of pkc1 mutants
YJL128C	[PBS2] MAP kinase kinase that plays a pivotal role in the osmosensing signal-transduction pathway, activated under severe osmotic stress MAP kinase kinase (MEK)|may act as a scaffolding protein for Sho1p, Ste11p, and Hog1p Null mutant is viable, sensitive to high osmolarity, sensitive to the antibiotic polymyxin B; shows marked decreased induction of transcription by osmotic stress that is mediated by stress response elements; a deletion in RGA1 and PBS2 activates the pheromone-dependent signal transduction pathway independently of the G protein

0358	
YFL042C	
YMR303C	[ADH2] Glucose-repressible alcohol dehydrogenase II, catalyzes the conversion of ethanol to acetaldehyde; involved in the production of certain carboxylate esters; regulated by ADR1 alcohol dehydrogenase II

0359	GO_TERM:[regulation of transcription by galactose] P-Value:1.9e-05 OVERLAP:[GAL80 complex] <510.190.80> SIZE:3
**YDR009W**	[GAL3] Transcriptional regulator involved in activation of the GAL genes in response to galactose; forms a complex with Gal80p and Gal4p to relieve inhibition by Gal80p; binds galactose and ATP but does not have galactokinase activity Galactose non-utilizer
**YML051W**	[GAL80] Transcriptional regulator involved in the repression of GAL genes in the absence of galactose; inhibits transcriptional activation by Gal4p; inhibition relieved by Gal3p or Gal1p binding transcriptional regulator Null mutant is viable but has constitutive expression of the GAL genes.

0360	GO_TERM:[RNA polymerase II transcription mediator activity] P-Value:2.7e-49 OVERLAP:[Kornberg's mediator (SRB) complex] <510.40.20> SIZE:21
YNL167C	[SKO1] Basic leucine zipper (bZIP) transcription factor of the ATF/CREB family that forms a complex with Tup1p and Ssn6p to both activate and repress transcription; cytosolic and nuclear protein involved in the osmotic and oxidative stress responses Null mutant is viable, associated with partial derepression of the SUC2 gene; associated with increased transcription through ATF/CREB sites. SKO1 is a multicopy suppressor of the lethality caused by overexpressing cAMP-dependent protein kinase and of the toxicity caused by overexpressing Rap1p
YBR112C	[CYC8] General transcriptional co-repressor, acts together with Tup1p; also acts as part of a transcriptional co-activator complex that recruits the SWI/SNF and SAGA complexes to promoters Null mutant is viable; high level constitutivity for invertase, clumpiness, temperature-sensitive growth, alpha-specific mating defects and failure of homozygous diploids to sporulate
YPR068C	[HOS1] Putative class I histone deacetylase (HDAC) with sequence similarity to Hda1p, Rpd3p, Hos2p, and Hos3p; deletion results in increased histone acetylation at rDNA repeats; interacts with the Tup1p-Ssn6p corepressor complex
YOR140W	[SFL1] Transcription repressor involved in regulation of flocculation-related genes, inhibits transcription by recruiting general corepressor Cyc8p-Tup1p to different promoters; negatively regulated by cAMP-dependent protein kinase A subunit Tpk2p transcription factor
YDR146C	[SWI5] Transcription factor that activates transcription of genes expressed in G1 phase and at the G1/M boundary; localization to the nucleus occurs during G1 and appears to be regulated by phosphorylation by Cdc28p kinase transcriptional activator homothallic switching deficient
YPR086W	[SUA7] Transcription factor TFIIB, a general transcription factor required for transcription initiation and start site selection by RNA polymerase II transcription factor TFIIB homolog
YPL248C	[GAL4] DNA-binding transcription factor required for the activation of the GAL genes in response to galactose; repressed by Gal80p and activated by Gal3p zinc finger transcription factor of the Zn(2)-Cys(6) binuclear cluster domain type Null mutant is viable, cannot utilize galactose as sole carbon source
YNL025C	[SSN8] Cyclin-like component of the RNA polymerase II holoenzyme, involved in phosphorylation of the RNA polymerase II C-terminal domain; involved in glucose repression and telomere maintenance C-type cyclin|associates with the Ssn3p cyclin-dependent kinase null is viable, exhibits set of phenotypes common to strains defective in SSN6/TUP1-mediated transcriptional repression. Other mutations show unscheduled meiotic gene expression (derepression of early meiotic genes), suppression of SNF1.
YCR084C	[TUP1] General repressor of transcription, forms complex with Cyc8p, involved in the establishment of repressive chromatin structure through interactions with histones H3 and H4, appears to enhance expression of some genes Null mutant is viable; exhibits flocculent colony morphology
YPL042C	[SSN3] Cyclin-dependent protein kinase, component of RNA polymerase II holoenzyme; involved in phosphorylation of the RNA polymerase II C-terminal domain; involved in glucose repression cyclin (SSN8)-dependent serine/threonine protein kinase null is viable, exhibits set of phenotypes common to strains defective in SSN6/TUP1-mediated transcriptional repression. Other mutations show unscheduled meiotic gene expression (derepression of early meiotic genes), suppression of SNF1.
YDR443C	[SSN2] Protein required for stable association of Srb10p-Srb11p kinase with RNA polymerase holoenzyme; subunit of the RNA polymerase II mediator complex; essential for transcriptional regulation transcription factor Null mutant is viable; ssn2 mutations can suppress CTD truncations or phosphorylation mutants and snf1 mutations

YCR081W	[SRB8] Subunit of the RNA polymerase II mediator complex; associates with core polymerase subunits to form the RNA polymerase II holoenzyme; essential for transcriptional regulation; involved in glucose repression
**YPL129W**	[TAF14] Subunit (30 kDa) of TFIID, TFIIF, and SWI/SNF complexes, involved in RNA polymerase II transcription initiation and in chromatin modification, contains a YEATS domain transcription initiation factor TFIIF small subunit Null mutant is viable but has a depolarized actin cytoskeleton.
**YMR112C**	[MED11] Subunit of the RNA polymerase II mediator complex; associates with core polymerase subunits to form the RNA polymerase II holoenzyme RNA polymerase II holoenzyme/mediator subunit 14 kDa
YGL127C	[SOH1] Subunit of the RNA polymerase II mediator complex; associates with core polymerase subunits to form the RNA polymerase II holoenzyme; involved in telomere maintenance; conserved with other metazoan MED31 subunits Null mutant is viable, shows 10-fold increase in recombination. soh1 mutants were originally identified as suppressors of hyperrecombination hpr1 mutants. Soh1p may functionally interact with components of the RNA polymerase II complex as suggested from the synthetic lethality observed in soh1 rpb delta 104, soh1 rpb2, and soh1 sua7 double mutants.
**YNL236W**	[SIN4] Subunit of the Mediator complex; interacts with the RNA polymerase II holoenzyme to postively or negatively regulate transcription; dispensible for basal transcription RNA polymerase II holoenzyme/mediator subunit Null mutant is viable, temperature sensitive, displays defects in both positive and negative regulation of transcription, suppresses Ty insertion mutations (Spt-), exhibits decreased superhelical density of circular DNA molecules, exhibits expression from promoters lacking UAS elements; associated with a defect in RME1-dependent repression and a methionine or cysteine requirement, exhibits flocculant/lacy colony morphology, suppressor of snf/swi mutations
**YER022W**	[SRB4] Subunit of the RNA polymerase II mediator complex; associates with core polymerase subunits to form the RNA polymerase II holoenzyme; essential for transcriptional regulation RNA polymerase II holoenzyme/mediator subunit Null mutant is inviable, srb4 (ts) mutants display global defects in mRNA synthesis; srb4 mutants are suppressed by mutations in NCB1
**YHR041C**	[SRB2] General transcription factor, subunit of the RNA polymerase II mediator complex; associates with core polymerase subunits to form the RNA polymerase II holoenzyme; involved in telomere maintenance RNA polymerase II holoenzyme/mediator subunit Null mutant is viable, the semi-dominant SRB2-1 mutation suppresses truncation of the C-terminal domain of RNA polymerase II
**YOL051W**	[GAL11] Component of the Mediator complex; interacts with RNA polymerase II and the general transcription factors to form the RNA polymerase II holoenzyme; affects transcription by acting as target of activators and repressors RNA polymerase II holoenzyme complex component|positive and negative transcriptional regulator of genes involved in mating-type specialization Null mutant is viable, exhibits reduced expression of Gal4 regulated genes
**YGR104C**	[SRB5] Subunit of the RNA polymerase II mediator complex; associates with core polymerase subunits to form the RNA polymerase II holoenzyme; essential for transcriptional regulation; involved in telomere maintenance RNA polymerase II holoenzyme/mediator subunit
**YOR174W**	[MED4] Subunit of the RNA polymerase II mediator complex; associates with core polymerase subunits to form the RNA polymerase II holoenzyme; essential for transcriptional regulation RNA polymerase II holoenzyme/mediator subunit
**YHR058C**	[MED6] Subunit of the RNA polymerase II mediator complex; associates with core polymerase subunits to form the RNA polymerase II holoenzyme; essential for transcriptional regulation
**YBR253W**	[SRB6] Subunit of the RNA polymerase II mediator complex; associates with core polymerase subunits to form the RNA polymerase II holoenzyme; essential for transcriptional regulation part of Srb/Mediator complex|transcription factor Null mutant is inviable; temperature sensitive mutants are associated with decreased total polyA+ RNA levels
**YLR071C**	[RGR1] Component of RNA polymerase II holoenzyme/mediator complex; affects chromatin structure and transcriptional regulation of diverse genes; required for glucose repression, HO repression, RME1 repression and sporulation RNA polymerase II holoenzyme/mediator subunit|interacts with Sin4p, Gal11p, and a 50 kDa polypeptide Null mutant is inviable, rgr1 mutants exhibit resistance to glucose represion, temperature sensitivity, sporulation; rgr1-ts allows sporulation of a/a diploids overexpressing RME1
**YDR308C**	[SRB7] Subunit of the RNA polymerase II mediator complex; associates with core polymerase subunits to form the RNA polymerase II holoenzyme; essential for transcriptional regulation; target of the global repressor Tup1p RNA polymerase II holoenzyme/mediator subunit
**YGL151W**	[NUT1] Component of the RNA polymerase II mediator complex, which is required for transcriptional activation and also has a role in basal transcription Null mutant is viable, deletion of NUT1 causes a constitutive, Swi4p-independent phenotype in combination with the nut2-1 allele or an allele of CCR4
**YGL025C**	[PGD1] Subunit of the Mediator global transcriptional cofactor complex, which is part of the RNA polymerase II holoenzyme and plays an essential role in basal and activated transcription; direct target of the Cyc8p-Tup1p transcriptional corepressor Suppresses hyper-deletion phenotype of hpr1 null mutant; reduces frequency of deletions in rad52-1 mutant
YPR070W	[MED1] Subunit of the RNA polymerase II mediator complex; associates with core polymerase subunits to form the RNA polymerase II holoenzyme; essential for transcriptional regulation essential for transcriptional regulation|mediator complex subunit 1 Defects in both repression and induction of GAL genes; supresses loss of the Snf1 kinase
**YOL135C**	[MED7] Subunit of the RNA polymerase II mediator complex; associates with core polymerase subunits to form the RNA polymerase II holoenzyme; essential for transcriptional regulation RNA polymerase II holoenzyme/mediator subunit
**YDL005C**	[MED2] Subunit of the RNA polymerase II mediator complex; associates with core polymerase subunits to form the RNA polymerase II holoenzyme; essential for transcriptional regulation RNA polymerase II holoenzyme/mediator subunit Null mutant is viable, unable to grow on galactose

**YBL093C**	[ROX3] RNA polymerase II holoenzyme component RNA polymerase II holoenzyme/mediator subunit
**YPR168W**	[NUT2] Component of the RNA polymerase II mediator complex, which is required for transcriptional activation and also has a role in basal transcription RNA polymerase II holoenzyme 21 kDa mediator subunit Null mutant is inviable, nut2-1 perturbs repression of URS2
**YBR193C**	[MED8] Subunit of the RNA polymerase II mediator complex; associates with core polymerase subunits to form the RNA polymerase II holoenzyme; essential for transcriptional regulation RNA polymerase II holoenzyme/mediator subunit
**YNR010W**	[CSE2] Component of the Med9/10 module, which is a subcomplex within the RNA polymerase II Mediator complex; required for regulation of RNA polymerase II activity RNA polymerase II mediator subcomplex component Null mutant is viable, accumulates large-budded cells, results in significant increase in chromosome missegregation, slower growth, and defective meiosis

0361	GO_TERM:[transcription factor TFIIE complex] P-Value:8.1e-07 OVERLAP:[TFIIE] <510.80> SIZE:2
**YKL028W**	[TFA1] TFIIE large subunit, involved in recruitment of RNA polymerase II to the promoter, activation of TFIIH, and promoter opening transcription factor tfIIE large subunit
**YKR062W**	[TFA2] TFIIE small subunit, involved in RNA polymerase II transcription initiation transcription factor TFIIE subunit

0362	GO_TERM:[rDNA binding] P-Value:2.9e-06
YDR110W	[FOB1] Nucleolar protein required for DNA replication fork blocking and recombinational hotspot activities; binds to the replication fork barrier site in the rDNA region; related to retroviral integrases DNA replication fork blocking protein Loss of replication fork blocking and recombinational hotspot activities.
YJL076W	[NET1] Core subunit of the RENT complex, which is a complex involved in nucleolar silencing and telophase exit; stimulates transcription by RNA polymerase I and regulates nucleolar structure Null mutant is viable and grows slowly

0363	GO_TERM:[RNA polymerase II transcription elongation factor activity] P-Value:5.3e-05
YPR133C	[SPN1] Protein that interacts with Spt6p and copurifies with Spt5p and RNA polymerase II, probable transcriptional elongation factor; metazoan homologs contain an acidic N terminus; mutations in the gene confer an Spt- phenotype involved in transcriptional elongation
YBR236C	[ABD1] Methyltransferase, catalyzes the transfer of a methyl group from S-adenosylmethionine to the GpppN terminus of capped mRNA RNA (guanine-7-)methyltransferase (cap methyltransferase)
YGR116W	[SPT6] Essential protein that interacts with histones and is involved in nucleosome disassembly and reassembly during transcription elongation interacts with histones, primarily histone H3, possesses nucleosome assembly activity|transcriptional regulator

0364	GO_TERM:[DNA-directed RNA polymerase III complex] P-Value:5.0e-32 OVERLAP:[RNA polymerase III] <510.120> SIZE:13
YDR045C	[RPC11] RNA polymerase III subunit C11; mediates pol III RNA cleavage activity and is important for termination of transcription TFIIS-like small Pol III subunit C11
YDR005C	[MAF1] Negative regulator of RNA polymerase III; component of several signaling pathways that repress polymerase III transcription in response to changes in cellular environment; targets the initiation factor TFIIIB Mislocalizes Mod5p to the nucleus. tRNA levels are elevated in maf1 mutant cells.
**YNL113W**	[RPC19] RNA polymerase subunit, common to RNA polymerases I and III RNA polymerases I (A) and III (C) subunit
**YPR110C**	[RPC40] RNA polymerase subunit, common to RNA polymerase I and III RNA polymerase III subunit
YKR025W	[RPC37] RNA polymerase III subunit C37 RNA polymerase III subunit
YJL011C	[RPC17] RNA polymerase III subunit C17; physically interacts with C31, C11, and TFIIIB70; may be involved in the recruitment of pol III by the preinitiation complex
**YNR003C**	[RPC34] RNA polymerase III subunit C34; interacts with TFIIIB70 and is a key determinant in pol III recruitment by the preinitiation complex RNA polymerase III (C) 34 kDa subunit
**YNL151C**	[RPC31] RNA polymerase III subunit C31; contains HMG-like C-terminal domain HMG1-like protein|RNA polymerase III (C) 31 kDa subunit
**YKL144C**	[RPC25] RNA polymerase III subunit C25 RNA polymerase III subunit

YDL150W	[RPC53] RNA polymerase III subunit C53 RNA polymerase III subunit Null mutant is inviable; temperature sensitive mutants show rapid inhibition of tRNA synthesis after shift to restricitive temperature and arrest in G1
**YOR116C**	[RPO31] RNA polymerase III subunit C160, part of core enzyme; similar to bacterial beta-prime subunit RNA polymerase III subunit
**YOR207C**	[RET1] Second-largest subunit of RNA polymerase III, which is responsible for the transcription of tRNA and 5S RNA genes, and other low molecular weight RNAs
**YPR190C**	[RPC82] RNA polymerase III subunit C82 82 kDa subunit of RNA polymerase III (C)
0365	GO_TERM:[RNA polymerase complex] P-Value:8.1e-39 OVERLAP:[RNA polymerase II] <510.40.10> SIZE:13
YPR010C	[RPA135] RNA polymerase I subunit A135 RNA polymerase I subunit suppression of rpb1, cold sensitive
YOR341W	[RPA190] RNA polymerase I subunit; largest subunit of RNA polymerase I RNA polymerase I subunit
YOR340C	[RPA43] RNA polymerase I subunit A43 DNA dependent RNA polymerase I subunit A43
YJL148W	[RPA34] RNA polymerase I subunit A34.5 Null mutant is viable but its RNA polymerase I lacks subunit A49 (rpa49p); synthetically lethal with RPA14; shows synthetic interactions with DNA topoisomerase I (TPO1)
YNL248C	[RPA49] RNA polymerase I subunit A49 RNA polymerase A 49 kDa alpha subunit Null mutant is viable but grows slowly and is temperature or cold sensitive; synthetically lethal with RPA14
YDR156W	[RPA14] RNA polymerase I subunit A14 RNA polymerase I subunit Null mutant is viable but is temperature sensitive
YJR063W	[RPA12] RNA polymerase I subunit A12.2; contains two zinc binding domains, and the N terminal domain is responsible for anchoring to the RNA pol I complex RNA polymerase I A12.2 subunit Null mutant is viable but is temperature sensitive; synthetically lethal with RPA14
YGL043W	[DST1] General transcription elongation factor TFIIS, enables RNA polymerase II to read through blocks to elongation by stimulating cleavage of nascent transcripts stalled at transcription arrest sites RNA polymerase II elongation factor|Transcription elongation factor S-II (TFIIS)|transcription elongation factor Null mutant is viable; reduced induction of DNA strand transfer; sensitivity to 6-azauracil
YGR063C	[SPT4] Protein that forms a complex with Spt5p and mediates both activation and inhibition of transcription elongation, and plays a role in pre-mRNA processing; in addition, Spt4p is involved in kinetochore function and gene silencing transcriptional regulator|zinc finger protein
YML010W	[SPT5] Protein that forms a complex with Spt4p and mediates both activation and inhibition of transcription elongation; Spt4p-Spt5p complex also plays a role in pre-mRNA processing transcription factor
YJL168C	[SET2] Histone methyltransferase with a role in transcriptional elongation, methylates a lysine residue of histone H3; associates with the C-terminal domain of Rpo21p; histone methylation activity is regulated by phosphorylation status of Rpo21p null is viable; a point mutant suppresses deletion of the UAS in the GAL4 promoter
**YOR224C**	[RPB8] RNA polymerase subunit ABC14.5, common to RNA polymerases I, II, and III 16 kDa RNA polymerase subunit (common to polymerases I, II and III)
**YDL140C**	[RPO21] RNA polymerase II largest subunit B220, part of central core; phosphorylation of C-terminal heptapeptide repeat domain regulates association with transcription and splicing factors; similar to bacterial beta-prime RNA polymerase II core subunit
YGR186W	[TFG1] TFIIF (Transcription Factor II) largest subunit; involved in both transcription initiation and elongation of RNA polymerase II; homologous to human RAP74 transcription factor TFIIF large subunit Mutating the Fcp1p-binding motif KEFGK in Tfg1p to EEFGE led to both synthetic phenotypes in certain fcp1tfg1 double mutants and a reduced ability of Fcp1p to activate transcription when it is artificially tethered to a promoter.
YGR005C	[TFG2] TFIIF (Transcription Factor II) middle subunit; involved in both transcription initiation and elongation of RNA polymerase II; homologous to human RAP30 transcription initiation factor TFIIF middle subunit
**YIL021W**	[RPB3] RNA polymerase II third largest subunit B44, part of central core; similar to prokaryotic alpha subunit RNA polymerase II 45 kDa subunit Null mutant is inviable; rpb3(ts) mutants at restrictive temperature exhibit no assembly of RNA polymerase II
**YOR151C**	[RPB2] RNA polymerase II second largest subunit B150, part of central core; similar to bacterial beta subunit
YDL115C	[IWR1] Protein of unknown function, deletion causes hypersensitivity to the K1 killer toxin
**YOR210W**	[RPB10] RNA polymerase subunit ABC10-beta, common to RNA polymerases I, II, and III RNA polymerase II core subunit
**YPR187W**	[RPO26] RNA polymerase subunit ABC23, common to RNA polymerases I, II, and III; part of central core; similar to bacterial omega subunit RNA polymerases I, II, and III subunit
**YBR154C**	[RPB5] RNA polymerase subunit ABC27, common to RNA polymerases I, II, and III; contacts DNA and affects transactivation 25 kDa RNA polymerase subunit (common to polymerases I, II and III)
**YJL140W**	[RPB4] RNA polymerase II subunit B32; forms two subunit dissociable complex with Rpb7p; dispensable under some environmental conditions; involved in export of mRNA to cytoplasm under stress conditions; involved in telomere maintenance RNA polymerase II fourth largest subunit Null mutant is viable, rbp4 mutants are heat and cold sensitive, exhibit slow growth at intermediate temperatures
**YDR404C**	[RPB7] RNA polymerase II subunit B16; forms two subunit dissociable complex with Rpb4p RNA polymerase II dissociable subunit

**YGL070C**	[RPB9] RNA polymerase II subunit B12.6; contacts DNA; mutations affect transcription start site; involved in telomere maintenance RNA polymerase II core subunit Null mutant is viable, heat and cold sensitive, exhibits altered transcription start sites at various genes
**YOL005C**	[RPB11] RNA polymerase II subunit B12.5; part of central core; similar to Rpc19p and bacterial alpha subunit RNA polymerase II core subunit

0366	GO_TERM:[mRNA capping] P-Value:5.2e-06 OVERLAP:[mRNA guanylyl transferase (capping complex)] <510.170> SIZE:2
YOR375C	[GDH1] NADP(+)-dependent glutamate dehydrogenase, synthesizes glutamate from ammonia and alpha-ketoglutarate; rate of alpha-ketoglutarate utilization differs from Gdh3p; expression regulated by nitrogen and carbon sources NADP-specific glutamate dehydrogenase
**YGL130W**	[CEG1] Alpha (guanylyltransferase) subunit of the mRNA capping enzyme, a heterodimer (the other subunit is CET1, an RNA 5'-triphophatase) involved in adding the 5' cap to mRNA; the mammalian enzyme is a single bifunctional polypeptide mRNA capping enzyme alpha subunit|mRNA guanylyltransferase
**YPL228W**	[CET1] Beta (RNA 5'-triphosphatase) subunit of the mRNA capping enzyme, a heterodimer (the other subunit is CEG1, a guanylyltransferase) involved in adding the 5' cap to mRNA; the mammalian enzyme is a single bifunctional polypeptide RNA 5'-triphosphatase|mRNA capping enzyme beta subunit (80 kDa)

0367	GO_TERM:[molecular_function] P-Value:3.7e-01
YGR187C	[HGH1] Protein of unknown function with similarity to human HMG1 and HMG2; localizes to the cytoplasm
YBR155W	[CNS1] TPR-containing co-chaperone; binds both Hsp82p (Hsp90) and Ssa1p (Hsp70) and stimulates the ATPase activity of SSA1, ts mutants reduce Hsp82p function while over expression suppresses the phenotypes of an HSP82 ts allele and a cpr7 deletion Null mutant is inviable; overexpression of CNS1 restores normal growth and Hsp90 activity in a cpr7 mutant strain.
YCL014W	[BUD3] Protein involved in bud-site selection and required for axial budding pattern; localizes with septins to bud neck in mitosis and may constitute an axial landmark for next round of budding Null mutant is viable; bipolar budding pattern in all cell types

0368	GO_TERM:[protein serine/threonine phosphatase activity] P-Value:1.3e-03
YLR216C	[CPR6] Peptidyl-prolyl cis-trans isomerase (cyclophilin), catalyzes the cis-trans isomerization of peptide bonds N-terminal to proline residues; binds to Hsp82p and contributes to chaperone activity cyclophilin 40|peptidyl-prolyl cis-trans isomerase (PPIase) Null mutant is viable, has normal growth rate
YOR220W	[YOR220W] protein that interacts genetically with the Pat1 mRNA-decapping factor
YNR032W	[PPG1] Putative serine/threonine protein phosphatase, required for glycogen accumulation; interacts with Tap42p, which binds to and regulates other protein phosphatases Null mutant is viable but accumulates less glycogen
YGR123C	[PPT1] Protein serine/threonine phosphatase with similarity to human phosphatase PP5; present in both the nucleus and cytoplasm; expressed during logarithmic growth
YKL117W	[SBA1] Co-chaperone that binds to and regulates Hsp90 family chaperones; important for pp60v-src activity in yeast; homologous to the mammalian p23 proteins HSP90 associated co-chaperone Null mutant is viable, exhibits slow growth at 18 degrees and 37 degrees; synthetic growth defects in SBA1-1/sti1-1 double mutant

0369	GO_TERM:[HIR complex] P-Value:1.9e-13
YJR140C	[HIR3] Transcriptional corepressor involved in the cell cycle-regulated transcription of histone genes HTA1, HTB1, HHT1, and HHT2; involved in position-dependent gene silencing and nucleosome reassembly HTA1-HTB1 transcription is derepressed and is no longer cell-cycle regulated
YBR215W	[HPC2] Highly charged, basic protein required for normal cell-cycle regulation of histone gene transcription; mutants display strong synthetic defects with subunits of FACT, a complex that allows RNA Pol II to elongate through nucleosomes highly charged basic protein altered cell cycle regulation of histone gene transcription; suppresses delta insertion mutations in the HIS4 and LYS2 loci
YBL008W	[HIR1] Non-essential transcriptional corepressor involved in the cell cycle-regulated transcription of histone H2A, H2B, H3 and H4 genes; contributes to nucleosome formation, heterochromatic gene silencing, and formation of functional kinetochores contains nuclear targeting signal|repressor protein (putative)|similar to Tup1p and mammalian retinal transducin Null mutant is viable, but HTA1-HTB1 transcription is derepressed and is no longer cell-cycle regulated; other mutations in this gene give 'spt' gene-class phenotype
YOR038C	[HIR2] Non-essential transcriptional corepressor involved in the cell cycle-regulated transcription of histone H2A, H2B, H3, and H4 genes; recruits Swi-Snf complexes to histone gene promoters; promotes heterochromatic gene silencing with Asf1p contains nuclear targeting signal|repressor protein (putative) Null mutant is viable, but HTA1-HTB1 transcription is derepressed and is no longer cell-cycle regulated; other mutations in this gene give 'spt' gene-class phenotype

0370	GO_TERM:[ISW1 complex] P-Value:4.9e-06

YLR095C	[IOC2] Member of a complex (Isw1b) with Isw1p and Ioc4p that exhibits nucleosome-stimulated ATPase activity and acts within coding regions to coordinate transcription elongation with termination and processing, contains a PHD finger motif
YMR044W	[IOC4] Member of a complex (Isw1b) with Isw1p and Ioc2p that exhibits nucleosome-stimulated ATPase activity and acts within coding regions to coordinate transcription elongation with termination and processing, contains a PWWP motif

0371	GO_TERM:[chromatin accessibility complex] P-Value:1.3e-09
YJL065C	[DLS1] Subunit of ISW2/yCHRAC chromatin accessibility complex along with Itc1p, Isw2p, and Dpb4p; involved in inheritance of telomeric silencing
YGL133W	[ITC1] Component of the ATP-dependent Isw2p-Itc1p chromatin remodeling complex, required for repression of a-specific genes, repression of early meiotic genes during mitotic growth, and repression of INO1 Null mutant is viable, but shows abnormal morphology and reduced mating efficiency when the disruption is in a MATalpha background.
YOR304W	[ISW2] Member of the imitation-switch (ISWI) class of ATP-dependent chromatin remodeling complexes; ATPase component that, with Itc1p, forms a complex required for repression of a-specific genes, INO1, and early meiotic genes during mitotic growth ATPase component of a two subunit chromatin remodeling complex Null mutant is viable, isw1 isw2 chd1 triple deletion mutants are synthetically temperature and formamide sensitive

0372	GO_TERM:[regulation of transcription from RNA polymerase II promoter] P-Value:2.2e-03
YBR049C	[REB1] RNA polymerase I enhancer binding protein; DNA binding protein which binds to genes transcribed by both RNA polymerase I and RNA polymerase II; required for termination of RNA polymerase I transcription RNA polymerase I enhancer binding protein
YLR176C	[RFX1] Protein involved in DNA damage and replication checkpoint pathway; recruits repressors Tup1p and Cyc8p to promoters of DNA damage-inducible genes; similar to a family of mammalian DNA binding RFX1-4 proteins
YKR001C	[VPS1] GTPase required for vacuolar protein sorting, functions in actin cytoskeleton organization via its interaction with Sla1p; required for late Golgi-retention of some proteins including Kex2p; involved in regulating peroxisome biogenesis GTP-binding protein (putative)|dynamin GTPase family member|similar to mammalian Mx proteins Null mutant is viable, but is sporulation defective, fails to grow at high temperature and shows abnormal organization of intracellular membranes
YPL082C	[MOT1] Essential abundant protein involved in regulation of transcription, removes Spt15p (TBP) from DNA via its C-terminal ATPase activity, forms a complex with TBP that binds TATA DNA with high affinity but with altered specificity helicase (putative)

0373	GO_TERM:[chromatin remodeling complex] P-Value:5.7e-49 OVERLAP:[RSC complex (Remodel the structure of chromatin)] <400> SIZE:10
YFL049W	[SWP82] Member of the SWI/SNF chromatin remodeling complex in which it plays an as yet unidentified role; has identifiable counterparts in closely related yeast species; abundantly expressed in many growth conditions; paralog of Npl6p
YOR290C	[SNF2] Catalytic subunit of the SWI/SNF chromatin remodeling complex involved in transcriptional regulation; contains DNA-stimulated ATPase activity; functions interdependently in transcriptional activation with Snf5p and Snf6p transcriptional regulator
YBR289W	[SNF5] Subunit of the SWI/SNF chromatin remodeling complex involved in transcriptional regulation; functions interdependently in transcriptional activation with Snf2p and Snf6p chromatin remodeling Snf/Swi complex subunit Null mutant is viable, sucrose and raffinose nonfermenter
YDR073W	[SNF11] Subunit of the SWI/SNF chromatin remodeling complex involved in transcriptional regulation; interacts with a highly conserved 40-residue sequence of Snf2p SWI/SNF global transcription activator complex component
YJL176C	[SWI3] Subunit of the SWI/SNF chromatin remodeling complex, which regulates transcription by remodeling chromosomes; required for transcription of many genes, including ADH1, ADH2, GAL1, HO, INO1 and SUC2 transcription factor
YHL025W	[SNF6] Subunit of the SWI/SNF chromatin remodeling complex involved in transcriptional regulation; functions interdependently in transcriptional activation with Snf2p and Snf5p chromatin remodeling Snf/Swi complex subunit Null mutant is viable, sucrose and raffinose nonfermenter
YNR023W	[SNF12] 73 kDa subunit of the SWI/SNF chromatin remodeling complex involved in transcriptional regulation; homolog of Rsc6p subunit of the RSC chromatin remodeling complex; deletion mutants are temperature-sensitive RSC chromatin remodeling complex Rsc6p subunit homolog|SWI/SNF transcription activation complex 73 kDa subunit Null mutant is viable but is temperature-sensitive, fails to transcribe SWI/SNF-dependent genes such as SUC2 and INO1, sucrose non-fermenting, defective in transcriptional activation by the glucocorticoid receptor; snf12 mutants are insensitive to expression of Adenovirus E1A protein
YPL016W	[SWI1] Subunit of the SWI/SNF chromatin remodeling complex, which regulates transcription by remodeling chromosomes; required for transcription of many genes, including ADH1, ADH2, GAL1, HO, INO1 and SUC2 zinc finger transcription factor null mutants are deficient in homothallic switching, unable to fully derepress ADH2 expression
YFR013W	[IOC3] Member of a complex (Isw1a) with Isw1p that has nucleosome-stimulated ATPase activity and represses transcription initiation by specific positioning of a promoter proximal dinucleosome; has homology to Esc8p, which is involved in silencing

YLL026W	[HSP104] Heat shock protein that cooperates with Ydj1p (Hsp40) and Ssa1p (Hsp70) to refold and reactivate previously denatured, aggregated proteins; responsive to stresses including: heat, ethanol, and sodium arsenite; involved in [PSI+] propagation heat shock protein 104 Null mutant is viable and defective in induced thermotolerance
YOR156C	[NFI1] SUMO ligase, catalyzes the covalent attachment of SUMO (Smt3p) to proteins; involved in maintenance of proper telomere length chromatin protein Null mutant is viable. SIZ2 is a dosage bypass suppressor of an SMT4 deletion. A siz1 siz2 deletion has a synthetic phenotype (slow growth).
YBR089C-A	[NHP6B] High-mobility group non-histone chromatin protein, functionally redundant with Nhp6Ap; homologous to mammalian high mobility group proteins 1 and 2; acts to recruit transcription factor Rcs1p to certain promoters 11 kDa nonhistone chromosomal protein Deleting both NHP6A and NHP6B gives temperature-sensitive yeast with morphological and cytoskeletal defects at the restrictive temperature; defects are suppressed by 1 M sorbitol in the medium; nhp6a nhp6b double mutant also lacks induction of a subset of genes
YBR245C	[ISW1] Member of the imitation-switch (ISWI) class of ATP-dependent chromatin remodeling complexes; ATPase that forms a complex with Ioc2p and Ioc4p to regulate transcription elongation, and a complex with Ioc3p to repress transcription initiation ATPase component of a four subunit chromatin remodeling complex Null mutant is viable, isw1 isw2 chd1 triple deletion mutants are synthetically temperature and formamide sensitive
YHR056C	[RSC30] One of 15 subunits of the 'Remodel the Structure of Chromatin' (RSC) complex; non-essential gene required for regulation of ribosomal protein genes and the cell wall/stress response; highly similar to Rsc3p; null mutants are osmosensitive
**YMR033W**	[ARP9] Actin-related protein involved in transcriptional regulation; subunit of the chromatin remodeling Snf/Swi complex actin related protein|chromatin remodeling Snf/Swi complex subunit Null mutant is viable, exhibits typical swi/snf phenotypes, including growth defects on media containing galactose, glycerol, or sucrose as sole carbon sources. ARP9 is required for expression of an HO-lacZ fusion gene and for full transcriptional enhancement by the GAL4 activator
YCR020W-B	[HTL1] Subunit of the RSC chromatin remodeling complex, a multisubunit complex that functions in transcriptional regulation, chromosome stability and establishing sister chromatid cohesion; involved in telomere maintenance Null mutant is viable but shows temperature-sensitive lethality
**YFR037C**	[RSC8] One of 15 subunits of the 'Remodel the Structure of Chromatin' (RSC) complex; essential for viability and mitotic growth; homolog of SWI/SNF subunit Swi3p, but unlike Swi3p, does not activate transcription of reporters
YGR275W	[RTT102] Component of both the SWI/SNF and RSC chromatin remodeling complexes, suggested role in chromosome maintenance; possible weak regulator of Ty1 transposition
YDR303C	[RSC3] One of 15 subunits of the 'Remodel the Structure of Chromatin' (RSC) complex; essential gene required for regulation of ribosomal protein genes and the cell wall/stress response; highly similar to Rsc30p inviable; ts mutants display a G2/M arrest
**YIL126W**	[STH1] ATPase component of the ATP-dependent RSC chromatin remodeling complex required for kinetochore function in chromosome segregation; required for expression of early meiotic genes; essential helicase-related protein homologous to Snf2p helicase related protein|snf2 homolog sth1 mutants exhibit altered centromeric and centromere-proximal chromatin structure and increased missegregation of authentic chromosomes; conditional mutants arrest at large bud stage with a single nucleus; null is inviable.
**YPR034W**	[ARP7] Actin-related protein involved in transcriptional regulation; subunit of the chromatin remodeling Snf/Swi complex actin related protein|chromatin remodeling Snf/Swi complex subunit Null mutant is viable, exhibits typical swi/snf phenotypes, including growth defects on media containing galactose, glycerol, or sucrose as sole carbon sources. ARP7 is required for expression of an HO-lacZ fusion gene and for full transcriptional enhancement by the GAL4 activator
YMR091C	[NPL6] Component of the RSC chromatin remodeling complex; interacts with Rsc3p, Rsc30p, Ldb7p, and Htl1p to form a module important for a broad range of RSC functions; involved in nuclear protein import and maintenance of proper telomere length
**YLR357W**	[RSC2] One of 15 subunits of the 'Remodel the Structure of Chromatin' (RSC) complex; required for expression of mid-late sporulation-specific genes; involved in telomere maintenance RSC complex member
YLR033W	[RSC58] Remodels the structure of chromatin complex 58KDa subunit; Chromatin Remodeling Complex subunit 58KDa Subunit of RSC Chromatin Remodeling Complex
**YGR056W**	[RSC1] One of 15 subunits of the 'Remodel the Structure of Chromatin' (RSC) complex; required for expression of mid-late sporulation-specific genes; contains two essential bromodomains, a bromo-adjacent homology (BAH) domain, and an AT hook RSC complex member Null mutant is viable, grows slowly
**YLR321C**	[SFH1] Subunit of the RSC chromatin remodeling complex required for kinetochore function in chromosome segregation; essential gene required for cell cycle progression; phosphorylated in the G1 phase of the cell cycle; Snf5p paralog Snf5p homolog|chromatin remodeling complex member, RSC sfh1 mutants exhibit altered centromeric and centromere-proximal chromatin structure and increased missegregation of authentic chromosomes; null mutant is inviable; sfh1 temp-sensitive mutants arrest in G1.
**YCR052W**	[RSC6] One of 15 subunits of the 'Remodel the Structure of Chromatin' (RSC) complex; essential for mitotic growth; homolog of SWI/SNF subunit Swp73p
**YKR008W**	[RSC4] One of 15 subunits of the 'Remodel the Structure of Chromatin' (RSC) complex; found in close proximity to nucleosomal DNA; displaced from the surface of nucleosomal DNA after chromatin remodeling RSC complex member
YML127W	[RSC9] One of 15 subunits of the 'Remodel the Structure of Chromatin' (RSC) complex; DNA-binding protein involved in the synthesis of rRNA and in transcriptional repression and activation of genes regulated by the Target of Rapamycin (TOR) pathway

0374	GO_TERM:[transcriptional activator activity] P-Value:2.6e-04

YBL005W	[PDR3] Transcriptional activator of the pleiotropic drug resistance network, regulates expression of ATP-binding cassette (ABC) transporters through binding to cis-acting sites known as PDREs (PDR responsive elements) pleiotropic drug resistance
YGL013C	[PDR1] Zinc cluster protein that is a master regulator involved in recruiting other zinc cluster proteins to pleiotropic drug response elements (PDREs) to fine tune the regulation of multidrug resistance genes zinc finger transcription factor of the Zn(2)-Cys(6) binuclear cluster domain type pleiotropic drug resistance, resistant to borrelidin, oligomycin, antimycin, cycloheximide, antibiotic, thioisoleucine, sulfometuron methyl; accumulation of neutral red

0375	GO_TERM:[transcription factor TFIIA complex] P-Value:2.4e-06 OVERLAP:[TFIIA] <510.50> SIZE:2
YGL016W	[KAP122] Karyopherin beta, responsible for import of the Toa1p-Toa2p complex into the nucleus; binds to nucleoporins Nup1p and Nup2p; may play a role in regulation of pleiotropic drug resistance karyopherin beta family member
**YKL058W**	[TOA2] TFIIA small subunit; involved in transcriptional activation, acts as antirepressor or as coactivator; homologous to smallest subunit of human and Drosophila TFIIA beta|transcription factor IIA subunit Null mutant is inviable. Overexpression of TFIIA partially suppresses an spt3 delta mutation.
**YOR194C**	[TOA1] TFIIA large subunit; involved in transcriptional activation, acts as antirepressor or as coactivator; homologous to largest and second largest subunits of human and Drosophila TFIIA transcription factor IIA subunit alpha Null mutant is inviable. Overexpression of TFIIA partially suppresses an spt3 delta mutation. toa1 mutants have Spt-phenotypes. spt3 delta toa1 double mutants are inviable.

0376	GO_TERM:[RNA polymerase II transcription elongation factor activity] P-Value:2.0e-15
YKL110C	[KTI12] Protein associated with the RNA polymerase II Elongator complex; involved in sensitivity to G1 arrest induced by Kluyveromyces lactis toxin, zymocin Elongator associated protein resistant to Kluyveromyces lactis toxin; over expression also results in resistance to Kluyveromyces lactis toxin; zymotoxin resistant; slow growth; thermo-sensitive above 38C; caffeine; Calcofluor White and 6-azauracil sensitive; G1 cell cycle delay
YGR200C	[ELP2] Elongator protein, part of the six-subunit RNA polymerase II Elongator histone acetyltransferase complex; target of Kluyveromyces lactis zymocin RNA polymerase II Elongator subunit
YPL101W	[ELP4] Elongator protein, part of the HAP subcomplex of Elongator, which is a six-subunit component of the RNA polymerase II holoenzyme; required for Elongator structural integrity and histone acetyltransferase activity RNA polymerase II Elongator protein subunit
YMR312W	[ELP6] Elongator protein, part of the HAP subcomplex of Elongator, which is a six-subunit component of the RNA polymerase II holoenzyme; required for Elongator structural integrity and histone acetyltransferase activity RNA polymerase II Elongator protein subunit
YHR187W	[IKI1] Subunit of the Elp4p-Iki1p-Elp6p-subcomplex of RNA polymerase II elongator complex, which is a histone acetyltransferase; iki1 mutations confer resistance to the K. lactis toxin zymocin Null mutant is viable but is insensitive to pGLK killer toxin; zymotoxin resistant; slow growth; thermo-sensitive above 38 0C; caffeine, Calcofluor White and 6-azauracil sensitive; G1 cell cycle delay
YLR384C	[IKI3] Subunit of RNA polymerase II elongator histone acetyltransferase complex, involved in maintaining its structural integrity; negatively regulates exocytosis independent of transcription, homolog of human familial dysautonomia (FD) protein Null mutant is viable; insensitive to pGKL killer toxin; zymotoxin resistant; slow growth; thermo-sensitive above 38 0C; caffeine, Calcofluor White and 6-azauracil sensitive; G1 cell cycle delay
YPL086C	[ELP3] Histone acetyltransferase subunit of the Elongator complex, which is a component of the RNA polymerase II holoenzyme; activity is directed specifically towards histones H3 and H4; disruption confers resistance to K. lactis zymotoxin RNA polymerase II Elongator subunit|histone acetyltransferase

0377	GO_TERM:[nuclear pore] P-Value:8.2e-06
YDR159W	[SAC3] Nuclear pore-associated protein, forms a complex with Thp1p that is involved in transcription and in mRNA export from the nucleus Null mutant is viable, grows more slowly and is larger than wild-type cells; exhibits increased benomyl resistance; in contrast to sac3-1, sac3 null mutants do not suppress the temperature and osmosensitivity of act1-1 mutants
YOL072W	[THP1] Nuclear pore-associated protein, forms a complex with Sac3p that is involved in transcription and in mRNA export from the nucleus; contains a PAM domain implicated in protein-protein binding Null mutant is viable and shows transcription-associated hyper-recombination and transcription elongation impairment, and is unable to transcribe the bacterial lacZ ORF

YOR257W	[CDC31] Component of the spindle pole body (SPB) half-bridge, required for SPB duplication in mitosis and meiosis II; homolog of mammalian centrin; interacts with Kar1p nuclear pore complex subunit|spindle pole body calcium-binding protein component Null mutant is inviable. cdc31 mutants form reductional dyads with unduplicated spindle pole bodies

0378	GO_TERM:[chromosome organization and biogenesis (sensu Eukaryota)] P-Value:2.9e-02
YCR082W	[AHC2] Protein of unknown function, putative transcriptional regulator; proposed to be a Ada Histone acetyltransferase complex component; GFP tagged protein is localized to the cytoplasm and nucleus
YOR023C	[AHC1] Subunit of the Ada histone acetyltransferase complex, required for structural integrity of the complex Ada histone acetyltransferase complex component

0379	GO_TERM:[transcription factor complex] P-Value:7.7e-52 OVERLAP:[SAGA complex] <510.190.10.20.10> SIZE:16
YCR042C	[TAF2] TFIID subunit (150 kDa), involved in RNA polymerase II transcription initiation TATA binding protein-associated factor
YMR005W	[TAF4] TFIID subunit (48 kDa), involved in RNA polymerase II transcription initiation; potential Cdc28p substrate TFIID subunit
YML098W	[TAF13] TFIID subunit (19 kDa), involved in RNA polymerase II transcription initiation, similar to histone H4 with atypical histone fold motif of Spt3-like transcription factors TFIID subunit
YML015C	[TAF11] TFIID subunit (40 kDa), involved in RNA polymerase II transcription initiation, similar to histone H3 with atypical histone fold motif of Spt3-like transcription factors TFIID subunit
YMR227C	[TAF7] TFIID subunit (67 kDa), involved in RNA polymerase II transcription initiation TFIID subunit
YML114C	[TAF8] TFIID subunit (65 kDa), involved in RNA polymerase II transcription initiation TFIID subunit
YPL011C	[TAF3] TFIID subunit (47 kDa), involved in promoter binding and RNA polymerase II transcription initiation TAF(II) complex component
YGR274C	[TAF1] TFIID subunit (145 kDa), involved in RNA polymerase II transcription initiation, has histone acetyltransferase activity, involved in promoter binding and G1/S progression Null mutant is inviable, taf145 (ts) mutants arrest as small unbudded cells with a G0 like morphology at the nonpermissive temperature|Mutations in region 4 (amino acid residues 199 to 303) confer both temperature-conditional (ts) growth phenotypes and transcription defects.
YER148W	[SPT15] TATA-binding protein, general transcription factor that interacts with other factors to form the preinitiation complex at promoters, essential for viability TFIID subunit
YER164W	[CHD1] Nucleosome remodeling factor that functions in regulation of transcription elongation; contains a chromo domain, a helicase domain and a DNA-binding domain; component of both the SAGA and SILK complexes transcriptional regulator Null mutant is viable, resistant to 6-azauracil
**YHR099W**	[TRA1] Subunit of SAGA and NuA4 histone acetyltransferase complexes; interacts with acidic activators (e.g., Gal4p) which leads to transcription activation; similar to human TRRAP, which is a cofactor for c-Myc mediated oncogenic transformation ATM/Mec1/TOR1/TOR2-related|NuA4 complex component
**YGR252W**	[GCN5] Histone acetyltransferase, acetylates N-terminal lysines on histones H2B and H3; catalytic subunit of the ADA and SAGA histone acetyltransferase complexes; founding member of the Gcn5p-related N-acetyltransferase superfamily histone acetyltransferase Null mutant is viable, sensitive to intra-S-phase DNA damage, and grows poorly on minimal media.
YBR111W-A	[SUS1] Protein involved in mRNA export coupled transcription activation; component of the SAGA histone acetylase complex
**YDR167W**	[TAF10] Subunit (145 kDa) of TFIID and SAGA complexes, involved in RNA polymerase II transcription initiation and in chromatin modification TFIID subunit
**YGL112C**	[TAF6] Subunit (60 kDa) of TFIID and SAGA complexes, involved in transcription initiation of RNA polymerase II and in chromatin modification, similar to histone H4 TATA-binding protein-associated-factor
**YDR448W**	[ADA2] Transcription coactivator, component of the ADA and SAGA transcriptional adaptor/HAT (histone acetyltransferase) complexes transcription factor Null mutant is viable, grows poorly on minimal media
**YDR145W**	[TAF12] Subunit (61/68 kDa) of TFIID and SAGA complexes, involved in RNA polymerase II transcription initiation and in chromatin modification, similar to histone H2A TFIID subunit
**YMR236W**	[TAF9] Subunit (17 kDa) of TFIID and SAGA complexes, involved in RNA polymerase II transcription initiation and in chromatin modification, similar to histone H3 TFIID subunit
**YBR198C**	[TAF5] Subunit (90 kDa) of TFIID and SAGA complexes, involved in RNA polymerase II transcription initiation and in chromatin modification

**YDR176W**	[NGG1] Transcriptional regulator involved in glucose repression of Gal4p-regulated genes; component of transcriptional adaptor and histone acetyltransferase complexes, the ADA complex, the SAGA complex, and the SLIK complex genetic and mutant analyses suggest that Ngg1p (Ada3p) is part of two transcriptional adaptor/HAT (histone acetyltransferase complexes, the 0.8 MD ADA complex and the 1.8 MD SAGA complex|transcription factor Null mutant is viable, grows poorly on minimal media
**YBR081C**	[SPT7] Subunit of the SAGA transcriptional regulatory complex, involved in proper assembly of the complex; also present as a C-terminally truncated form in the SLIK/SALSA transcriptional regulatory complex histone acetyltransferase SAGA complex member|transcription factor Null mutant is viable, exhibits growth defects on glucose and galactose, fails to grow on media lacking inositol
**YPL254W**	[HFI1] Adaptor protein required for structural integrity of the SAGA complex, a histone acetyltransferase-coactivator complex that is involved in global regulation of gene expression through acetylation and transcription functions Ada/Gcn5 protein complex member|transcription factor Null mutant phenotypes similar to spt20/ada5 and spt7 mutants. Null mutant is viable, elongated cells, hyperpolarized actin cytoskeleton, heat sensitive, non-respiratory, inositol auxotroph, exhibits suppression of Ty insertion mutations; hfi1 hta1 double mutant is inviable.
**YOL148C**	[SPT20] Subunit of the SAGA transcriptional regulatory complex, involved in maintaining the integrity of the complex histone acetyltransferase SAGA complex member|transcription factor Null mutant is viable, exhibits growth defects on glucose and galactose, fails to grow on media lacking inositol
**YDR392W**	[SPT3] Subunit of the SAGA and SAGA-like transcriptional regulatory complexes, interacts with Spt15p to activate transcription of some RNA polymerase II-dependent genes, also functions to inhibit transcription at some promoters histone acetyltransferase SAGA complex member|transcription factor Null mutant is viable, exhibits defects in mating and sporulation, Ty transcription, and suppression of certain Ty insertion mutations
**YLR055C**	[SPT8] Subunit of the SAGA transcriptional regulatory complex but not present in SAGA-like complex SLIK/SALSA, required for SAGA-mediated inhibition at some promoters probable member of histone acetyltransferase SAGA complex|transcription factor Null mutant is viable, no growth defects, exhibits suppression of Ty insertion mutations, defects in Ty transcription
**YCL010C**	[SGF29] SaGa associated Factor 29kDa; Probable 29kKDa Subunit of SAGA histone acetyltransferase complex Probable 29kKDa Subunit of SAGA histone acetyltransferase complex
YMR223W	[UBP8] Ubiquitin-specific protease that is a component of the SAGA (Spt-Ada-Gcn5-Acetyltransferase) acetylation complex; required for SAGA-mediated deubiquitination of histone H2B
**YGL066W**	[SGF73] Probable 73 kDa subunit of SAGA histone acetyltransferase complex Probable 73KkDa Subunit of SAGA histone acetyltransferase complex
YPL047W	[SGF11] Integral subunit of SAGA histone acetyltransferase complex, regulates transcription of a subset of SAGA-regulated genes, required for the Ubp8p association with SAGA and for H2B deubiquitylation

0380	GO_TERM:[organelle organization and biogenesis] P-Value:1.2e-01 OVERLAP:[Tubulin-associated proteins] <140.30.20> SIZE:14
YER116C	[SLX8] Protein containing a RING finger domain that forms a complex with Hex3p; mutant phenotypes and genetic interactions suggest a possible role in resolving recombination intermediates during DNA replication or repair
**YOR058C**	[ASE1] Member of a family of microtubule-associated proteins (MAPs) that function at the mitotic spindle midzone; required for spindle elongation; undergoes cell cycle-regulated degradation by anaphase promoting complex; potential Cdc28p substrate spindle midzone component Null mutant is viable but temperature sensitive.

0381	GO_TERM:[protein serine/threonine phosphatase activity] P-Value:3.1e-04
YDL047W	[SIT4] Type 2A-related serine-threonine phosphatase that functions in the G1/S transition of the mitotic cycle; cytoplasmic and nuclear protein that modulates functions mediated by Pkc1p including cell wall and actin cytoskeleton organization similar to catalytic subunit of bovine type 2A protein phosphatase sit1-sit4 or sit2-sit4 double mutants are lethal
YJL098W	[SAP185] Protein that forms a complex with the Sit4p protein phosphatase and is required for its function; member of a family of similar proteins including Sap4p, Sap155p, and Sap190p Null mutant is viable; sap185 sap190 double mutants grow slowly; sap155 sap185 sap190 triple mutants are inviable in ssd1-d backgrounds
YOR187W	[TUF1] Mitochondrial translation elongation factor Tu; comprises both GTPase and guanine nucleotide exchange factor activities, while these activities are found in separate proteins in S. pombe and humans translation elongation factor Tu, mitochondrial Null mutant is viable, blocks mitochondrial translation and destabilizes mitochondrial genome.

0382	GO_TERM:[nucleosome] P-Value:2.3e-04
YPR031W	[NTO1] Component of the histone acetyltransferase complex HAT complex component Null: viable. Other phenotypes: require for NuA3 complex integrity
YBL052C	[SAS3] Histone acetyltransferase catalytic subunit of NuA3 complex that acetylates histone H3, involved in transcriptional silencing; homolog of the mammalian MOZ proto-oncogene; sas3 gcn5 double mutation confers lethality
YOR064C	[YNG1] Subunit of the NuA3 histone acetyltransferase complex that acetylates histone H3; has similarity to the human tumor suppressor ING1 histone acetyltransferase complex component carbon source sensitive, heat shock sensitive

0383	GO_TERM:[response to drug] P-Value:1.5e-04
YIL153W	[RRD1] Activator of the phosphotyrosyl phosphatase activity of PP2A; regulates G1 phase progression, the G2/M phase transition, microtubule dynamics, the osmoresponse, bud morphogenesis and DNA repair; subunit of the Tap42p-Sit4p-Rrd1p complex Null mutant shows pleiotropic phenotypes (eg. caffeine and rapamycin resistance, vanadate and calcium sensitivity, etc.); synthetic lethal with RRD2; lethality of rrd1rrd2 suppressed by increased osmolarity and also under oxygen-limited conditions.
YMR028W	[TAP42] Essential protein involved in the TOR signaling pathway; physically associates with the protein phosphatase 2A and the SIT4 protein phosphatase catalytic subunits 42 kDa protein that physically associates with the PP2A and SIT4 protein phosphatase catalytic subunits
YDR293C	[SSD1] Protein with a role in maintenance of cellular integrity, interacts with components of the TOR pathway; ssd1 mutant of a clinical S. cerevisiae strain displays elevated virulence Suppressor of regulatory subunit of protein kinase
YPR040W	[TIP41] Protein that interacts physically and genetically with Tap42p, which regulates protein phosphatase 2A; component of the TOR (target of rapamycin) signaling pathway Homozygous null mutants did not sporulate, showed resistance to benomyl; homozygous and haploid deletants were sensitive to thiabendazol
YBL046W	[PSY4] Putative protein of unknown function; green fluorescent protein (GFP)-fusion protein localizes to the nucleus Regulatory subunit of Pph3p
YDR075W	[PPH3] Catalytic subunit of protein phosphatase; involved in activation of Gln3p, which is a transcription factor with a role in nitrogen utilization protein phosphatase type 2A Null mutant is viable, pph3 pph21 pph22 mutants are inviable
YNL201C	[PSY2] Nuclear protein of unknown function; deletion results in sensitivity to anticancer drugs oxaliplatin and cisplatin, but not mitomycin C; deletion is synthetically lethal with a chitin synthase (CHS1) null mutant

0384	GO_TERM:[protein kinase CK2 complex] P-Value:1.5e-09 OVERLAP:[Casein kinase II] <120.20> SIZE:4
YMR172W	[HOT1] Transcription factor required for the transient induction of glycerol biosynthetic genes GPD1 and GPP2 in response to high osmolarity; targets Hog1p to osmostress responsive promoters; has similarity to Msn1p and Gcr1p nuclear protein osmostress hypersensitivity
**YOR039W**	[CKB2] Beta' regulatory subunit of casein kinase 2, a Ser/Thr protein kinase with roles in cell growth and proliferation; the holoenzyme also contains CKA1, CKA2 and CKB1, the many substrates include transcription factors and all RNA polymerases protein kinase CK2, beta' subunit
**YGL019W**	[CKB1] Beta regulatory subunit of casein kinase 2, a Ser/Thr protein kinase with roles in cell growth and proliferation; the holoenzyme also contains CKA1, CKA2 and CKB2, the many substrates include transcription factors and all RNA polymerases protein kinase CK2 beta subunit Null mutant is viable, exhibits salt sensitivity specific to NaCl and LiCl
**YOR061W**	[CKA2] Alpha' catalytic subunit of casein kinase 2, a Ser/Thr protein kinase with roles in cell growth and proliferation; the holoenzyme also contains CKA1, CKB1 and CKB2, the many substrates include transcription factors and all RNA polymerases protein kinase CK2 alpha' subunit Null mutant is viable, cka1 cka2 double deletion mutants are inviable; Cells in which protein kinase CK2 activity is depleted increase substantially in size prior to growth arrest, and a significant fraction of the arrested cells exhibit a pseudomycelial morphology. Disruption of the activity also results in flocculation. Yeast strains lacking both endogenous catalytic subunit genes can be rescued by expression of the alpha and beta subunits of Drosophila protein kinase CK2 or by expression of the Drosophila alpha subunit alone

0385	GO_TERM:[transcription elongation factor complex] P-Value:3.0e-17 OVERLAP:[RNA polymerase II] <510.40.10> SIZE:13
YGL207W	[SPT16] Subunit of the heterodimeric FACT complex (Spt16p-Pob3p), facilitates RNA Polymerase II transcription elongation through nucleosomes by destabilizing and then reassembling nucleosome structure suppression of Ty insertion mutations
YML069W	[POB3] Subunit of the heterodimeric FACT complex (Spt16p-Pob3p), which facilitates RNA Polymerase II transcription elongation through nucleosomes by destabilizing and then reassembling nucleosome structure DNA polymerase delta binding protein
**YLR418C**	[CDC73] Constituent of Paf1 complex with RNA polymerase II, Paf1p, Hpr1p, Ctr9, Leo1, Rtf1 and Ccr4p, distinct from Srb-containing Pol II complexes; required for expression of certain genes, modification of some histones, and telomere maintenance Mutations affect cell growth and the abundance of transcripts from a subset of genes
YBR279W	[PAF1] RNA polymerase II-associated protein, defines a large complex that is biochemically and functionally distinct from the Srb-Mediator form of Pol II holoenzyme and is required for full expression of a subset of cell cycle-regulated genes
YOR123C	[LEO1] Component of the Paf1 complex, which associates with RNA polymerase II and is involved in histone methylation
YGL244W	[RTF1] Subunit of the RNA polymerase II-associated Paf1 complex; directly or indirectly regulates DNA-binding properties of Spt15p and relative activities of different TATA elements; involved in telomere maintenance nuclear protein Null mutant is viable and can suppress TATA box-binding protein mutants (SPT15) in an allele-specific fashion

YOL145C	[CTR9] Component of the Paf1p complex, which is a large complex that binds to and modulates the activity of RNA polymerase II and is required for expression of a subset of genes, including cyclin genes; contains TPR repeats Null mutant is viable, loses chromosomes and shows temperature sensitivity

0386	GO_TERM:[heterochromatin] P-Value:2.1e-07
YLR278C	
YDR227W	[SIR4] Silent information regulator that, together with SIR2 and SIR3, is involved in assembly of silent chromatin domains at telomeres and the silent mating-type loci; potentially phosphorylated by Cdc28p; some alleles of SIR4 prolong lifespan silencing regulator at HML, HMR, and telomeres
YDR440W	[DOT1] Nucleosomal histone H3-Lys79 methylase, associates with transcriptionally active genes, functions in gene silencing at telomeres, most likely by directly modulating chromatin structure and Sir protein localization Null mutant is viable, bypasses meiotic arrest of zip1 mutant, and shows decreased silencing at telomeres, HML, and HMR. Overexpression causes loss of silencing at telomeres, HML, HMR, and slightly at rDNA
YDL042C	[SIR2] Conserved NAD+ dependent histone deacetylase of the Sirtuin family involved in regulation of lifespan; plays roles in silencing at HML, HMR, telomeres, and the rDNA locus; negatively regulates initiation of DNA replication nuclear NAD-dependent deacetylase Null mutant is viable; sir2 mutations suppress mitotic and meiotic intra- and interchromosomal rDNA recombination (10-15 fold). RAD52 and RAD50 are dispensable for basal level rDNA exchange in SIR2 but are required for increased exchange in sir2
YLR442C	[SIR3] Silencing protein that interacts with Sir2p and Sir4p, and histone H3 and H4 tails, to establish a transcriptionally silent chromatin state; required for spreading of silenced chromatin; recruited to chromatin through interaction with Rap1p silencing regulator at HML, HMR, and telomeres sterile

0387	GO_TERM:[DNA metabolism] P-Value:4.7e-02
YJL047C	[RTT101] Cullin subunit of a Roc1p-dependent E3 ubiquitin ligase complex; deletion phenotype suggests a role in anaphase progression; interacts with Mms22p and implicated in Mms22-dependent DNA repair; modified by the ubiquitin-like protein, Rub1p Null mutant is viable and causes an increase in Ty1 transposition
YLR320W	[MMS22] Protein involved in resistance to ionizing radiation; acts with Mms1p in a repair pathway that may be involved in resolving replication intermediates or preventing the damage caused by blocked replication forks Null: Null phenotype in haploids of either mating type and diploid is extreme sensitivity to MMS or hydroxyurea, moderate sensitivity to gamma or UV irradiation. Diploid is very sensitive to camtothecin. Diploid is also sensitive to bleomycin..

0388	GO_TERM:[protein folding] P-Value:1.2e-03
YCR060W	[TAH1] HSP90 cofactor; interacts with Hsp82p, Pih1p, Rvb1 and Rvb2, contains a single TPR domain with at least two TPR motifs
YHR034C	[PIH1] Protein of unresolved function; may function in protein folding and/or rRNA processing, interacts with a chaperone (Hsp82p), two chromatin remodeling factors (Rvb1p, Rvb2p) and two rRNA processing factors (Rrp43p, Nop58p) null has both reduced growth and reduced protein synthesis rates

0389	GO_TERM:[nucleosome] P-Value:2.0e-19 OVERLAP:[Nucleosomal protein complex] <320> SIZE:8
YBR274W	[CHK1] DNA damage checkpoint effector kinase, mediates mitotic cell cycle arrest via phosphorylation of Pds1p; phosphorylated by checkpoint signal transducer kinase Mec1p; homolog of S. pombe and mammalian Chk1 checkpoint kinase protein kinase Mutants are defective in the DNA damage checkpoint operating at metaphase
YDR174W	[HMO1] Chromatin associated high mobility group (HMG) family member involved in genome maintenance; rDNA-binding component of the Pol I transcription system; associates with a 5'-3' DNA helicase and Fpr1p, a prolyl isomerase high mobility group (HMG) family Null mutant is viable, but grows slowly and shows higher than normal plasmid loss rate
YKR048C	[NAP1] Protein that interacts with mitotic cyclin Clb2p; required for the regulation of microtubule dynamics during mitosis; controls bud morphogenesis; involved in the transport of H2A and H2B histones to the nucleus nucleosome assembly protein I Null mutant is viable but exhibits defects in Clb2 function.
YGL241W	[KAP114] Karyopherin, responsible for nuclear import of Spt15p, histones H2A and H2B, and Nap1p; amino terminus shows similarity to those of other importins, particularly Cse1p; localization is primarily nuclear
YGL097W	[SRM1] Nucleotide exchange factor for Gsp1p, localizes to the nucleus, required for nucleocytoplasmic trafficking of macromolecules; potentially phosphorylated by Cdc28p pheromone response pathway suppressor recessive mutation activates signal transduction pathway required for mating; leads to arrest in G1. mutant phenotype reveals defect in plasmid and chromosome stability, suggesting defect in DNA replication, mitosis, or their coordination.
**YNL030W**	[HHF2] One of two identical histone H4 proteins (see also HHF1); core histone required for chromatin assembly and chromosome function; contributes to telomeric silencing; N-terminal domain involved in maintaining genomic integrity histone H4 (HHF1 and HHF2 code for identical proteins)

**YNL031C**	[HHT2] One of two identical histone H3 proteins (see also HHT1); core histone required for chromatin assembly, involved in heterochromatin-mediated telomeric and HM silencing; regulated by acetylation, methylation, and mitotic phosphorylation histone H3 (HHT1 and HHT2 code for identical proteins)
YOL054W	[YOL054W] Nuclear protein, putative RNA polymerase II elongation factor; isolated as Pob3p/Spt16p-binding protein
**YBR010W**	[HHT1] One of two identical histone H3 proteins (see also HHT2); core histone required for chromatin assembly, involved in heterochromatin-mediated telomeric and HM silencing; regulated by acetylation, methylation, and mitotic phosphorylation histone H3 (HHT1 and HHT2 code for identical proteins)
**YBR009C**	[HHF1] One of two identical histone H4 proteins (see also HHF2); core histone required for chromatin assembly and chromosome function; contributes to telomeric silencing; N-terminal domain involved in maintaining genomic integrity histone H4 (HHF1 and HHF2 code for identical proteins)
**YBL003C**	[HTA2] One of two nearly identical (see also HTA1) histone H2A subtypes; core histone required for chromatin assembly and chromosome function; DNA damage-dependent phosphorylation by Mec1p facilitates DNA repair; acetylated by Nat4p histone H2A (HTA1 and HTA2 code for nearly identical proteins) Null mutant is viable. Deletion of the HTA2-HTB2 (TRT2) locus has no reported observable phenotypes, presumably because HTA1-HTB1 (TRT1) expression is upregulated and can compensate in the absence of TRT2. Overexpression of TRT2 can suppress Ty insertion mutations
**YBL002W**	[HTB2] One of two nearly identical (see HTB1) histone H2B subtypes required for chromatin assembly and chromosome function; Rad6p-Bre1p-Lge1p mediated ubiquitination regulates transcriptional activation, meiotic DSB formation and H3 methylation histone H2B (HTB1 and HTB2 code for nearly identical proteins) Null mutant is viable. Deletion of the HTA2-HTB2 (TRT2) locus has no reported observable phenotypes, presumably because HTA1-HTB1 (TRT1) expression is upregulated and can compensate in the absence of TRT2
**YDR224C**	[HTB1] One of two nearly identical (see HTB2) histone H2B subtypes required for chromatin assembly and chromosome function; Rad6p-Bre1p-Lge1p mediated ubiquitination regulates transcriptional activation, meiotic DSB formation and H3 methylation histone H2B (HTB1 and HTB2 code for nearly identical proteins)
**YDR225W**	[HTA1] One of two nearly identical (see also HTA2) histone H2A subtypes; core histone required for chromatin assembly and chromosome function; DNA damage-dependent phosphorylation by Mec1p facilitates DNA repair; acetylated by Nat4p histone H2A (HTA1 and HTA2 code for nearly identical proteins)

0390	GO_TERM:[H4/H2A histone acetyltransferase complex] P-Value:6.4e-29 OVERLAP:[NuA4 complex] <230.20.40> SIZE:2
YJR082C	[EAF6] Esa1p-associated factor, subunit of the NuA4 acetyltransferase complex
YNL107W	[YAF9] Subunit of both the NuA4 histone H4 acetyltransferase complex and the SWR1 complex, may function to antagonize silencing near telomeres; interacts directly with Swc4p, has homology to human leukemogenic protein AF9, contains a YEATS domain
YGR002C	[SWC4] Protein of unknown function, component of the Swr1p complex that incorporates Htz1p into chromatin; component of the NuA4 histone acetyltransferase complex
YPR023C	[EAF3] Esa1p-associated factor, nonessential component of the NuA4 acetyltransferase complex, homologous to Drosophila dosage compensation protein MSL3
YNL136W	[EAF7] Subunit of the NuA4 histone acetyltransferase complex, which acetylates the N-terminal tails of histones H4 and H2A
YHR090C	[YNG2] Subunit of the NuA4 histone acetyltransferase complex that acetylates histone H4 and H2A; has similarity to the human tumor suppressor ING1 NuA4 histone acetyltransferase complex component carbon source-, heat shock-, temperature-, and caffeine-sensitive, abnormal morphology, reduced histone H4 acetylation; BEM and RAD phenotypes; haploid yng2 mutants do not tolerate mutations in genes important for nonhomologous end joining repair yet remain proficient for homologous recombination.
YFL024C	[EPL1] Component of NuA4, which is an essential histone H4/H2A acetyltransferase complex; homologous to Drosophila Enhancer of Polycomb NuA4 histone acetyltransferase complex component
**YOR244W**	[ESA1] Histone acetyltransferase catalytic subunit of the native multisubunit complex (NuA4) that acetylates four conserved internal lysines of histone H4 N-terminal tail; required for cell cycle progression NuA4 complex component|acetyltransferase in the SAS gene family
YDR359C	[VID21] Component of the NuA4 histone acetyltransferase complex
YEL018W	[EAF5] Esa1p-associated factor, subunit of the NuA4 acetyltransferase complex

0391	GO_TERM:[chromatin remodeling complex] P-Value:9.5e-32
YLR085C	[ARP6] Nuclear actin-related protein involved in chromatin remodeling, component of chromatin-remodeling enzyme complexes
YOL012C	[HTZ1] Histone variant H2AZ, exchanged for histone H2A in nucleosomes by the SWR1 complex; involved in transcriptional regulation through prevention of the spread of silent heterochromatin evolutionarily conserved member of the histone H2A F/Z family of histone variants Null mutant is viable at 28C; high copy suppressor of histone H4 point mutant affecting nucleosome structure
YLR385C	[SWC7] Protein of unknown function, component of the Swr1p complex that incorporates Htz1p into chromatin
YDR334W	[SWR1] Swi2/Snf2-related ATPase, component of the SWR1 complex; required for the incorporation of Htz1p into chromatin Null: Null mutant is viable and shows no growth defects; swr1 rat8-2 and swr1 rsc9-1double mutants has a slow growth phenotype; SWR1 is a partial High copy suppressor of pse1-1 kap123

YDR485C	[VPS72] Protein of unknown function, component of the Swr1p complex that incorporates Htz1p into chromatin; required for vacuolar protein sorting Null mutant secretes CPY.
YAL011W	[SWC3] Protein of unknown function, component of the Swr1p complex that incorporates Htz1p into chromatin; required for formation of nuclear-associated array of smooth endoplasmic reticulum known as karmellae
YBR231C	[SWC5] Protein of unknown function, component of the Swr1p complex that incorporates Htz1p into chromatin Null: Cold-sensitive; Benomyl hypersensitive; Latrunculin-A hypersensitive
YML041C	[VPS71] Protein of unknown function, component of the Swr1p complex that incorporates Htz1p into chromatin; required for vacuolar protein sorting Null mutant secretes CPY.
YFL039C	[ACT1] Actin, structural protein involved in cell polarization, endocytosis, and other cytoskeletal functions actin
YNL215W	[IES2] Protein that associates with the INO80 chromatin remodeling complex under low-salt conditions
YEL044W	[IES6] Protein that associates with the INO80 chromatin remodeling complex under low-salt conditions Null: non essential.
YOR189W	[IES4] Protein that associates with the INO80 chromatin remodeling complex under low-salt conditions Null: non essential.
YPL235W	[RVB2] Essential protein involved in transcription regulation; component of chromatin remodeling complexes; required for assembly and function of the INO80 complex; member of the RUVB-like protein family transcriptional regulator
YDR190C	[RVB1] Essential protein involved in transcription regulation; component of chromatin remodeling complexes; required for assembly and function of the INO80 complex; member of the RUVB-like protein family
YJL081C	[ARP4] Nuclear actin-related protein involved in chromatin remodeling, component of chromatin-remodeling enzyme complexes actin related protein
YDL002C	[NHP10] Protein related to mammalian high mobility group proteins; likely component of the INO80 complex, which is an ATP-dependent chromatin-remodeling complex HMG1-box containing protein null mutant is viable and has normal growth rate
YGL150C	[INO80] ATPase that forms a large complex, containing actin and several actin-related proteins, that has chromatin remodeling activity and 3' to 5' DNA helicase activity in vitro; shows similarity to the Snf2p family of ATPases
YOR141C	[ARP8] Nuclear actin-related protein involved in chromatin remodeling, component of chromatin-remodeling enzyme complexes
YLR052W	[IES3] Subunit of the INO80 chromatin remodeling complex
YER092W	[IES5] Protein that associates with the INO80 chromatin remodeling complex under low-salt conditions Null: non essential.
YFL013C	[IES1] Subunit of the INO80 chromatin remodeling complex
YNL059C	[ARP5] Nuclear actin-related protein involved in chromatin remodeling, component of chromatin-remodeling enzyme complexes actin related protein
